# Common bursting relationships underlie eukaryotic transcription dynamics

**Published:** 2023-04-18

**Authors:** Po-Ta Chen, Benjamin Zoller, Michal Levo, Thomas Gregor

**Affiliations:** 1 Joseph Henry Laboratories of Physics & Lewis-Sigler Institute for Integrative Genomics, Princeton University, Princeton, NJ 08544, USA; 2 Department of Stem Cell and Developmental Biology, CNRS UMR3738 Paris Cité, Institut Pasteur, 25 rue du Docteur Roux, 75015 Paris, France

## Abstract

Transcription commonly occurs in bursts resulting from alternating productive (ON) and quiescent (OFF) periods. Yet how transcriptional bursts are regulated to determine spatiotemporal transcriptional activity remains unclear. Here we perform live transcription imaging of key developmental genes in the fly embryo, with single polymerase sensitivity. Quantification of single allele transcription rates and multi-polymerase bursts reveals shared bursting relationships among all genes, across time and space, as well as *cis*- and *trans*-perturbations. We identify the allele’s ON-probability as the main determinant of the transcription rate, while changes in the transcription initiation rate are limited. Any given ON-probability determines a specific combination of mean ON and OFF times, preserving a constant characteristic bursting time scale. Our findings point to a convergence of various regulatory processes that predominantly affect the ON-probability, thereby controlling mRNA production rather than mechanism-specific modulation of ON and OFF times. Our results thus motivate and guide new investigations into the mechanisms implementing these bursting rules and governing transcriptional regulation.

## INTRODUCTION

Eukaryotic transcriptional regulation is an inherently dynamic and stochastic process. Multiple molecular events orchestrate, in space and time, the initiation of productive transcription by individual RNA polymerases (Pol II complexes), leading to the synthesis of nascent RNA [1, 2]. The amount of transcribed mRNA molecules in turn shapes protein production and thereby dictates cellular behavior. Studies across various systems, from yeast to mammalian cells, have revealed that transcription occurs in bursts, namely the release of multiple Pol IIs in what is often referred to as an ON period, followed by a quiescent OFF period [3–8]. Yet it remains unclear how the kinetic parameters of transcriptional bursting determine mRNA production and govern spatiotemporal transcription dynamics. Is the transcription rate controlled primarily by tuning the durations of ON or OFF periods, the initiation rate (the rate of Pol II release during active periods), or by a combination of these? Furthermore, are distinct bursting parameters controlled by specific regulatory processes, and are distinct bursting strategies underlying temporal versus spatial (tissue-specific) control of transcription?

As a multitude of molecular processes is known to influence transcriptional activity, several studies aimed to uncover links between regulatory determinants and parameters of transcriptional bursting [9–11]. Transcriptional bursting is commonly described by parameters such as burst size, burst frequency, the kinetic rates governing ON and OFF times as well as transcription initiation [3, 12, 13]. Regulatory determinants, such as transcription factor (TF) binding, *cis*-regulatory elements, nucleosome occupancy, histone modification, and enhancer-promoter interactions were suggested to affect distinct bursting parameters [14–22]. Yet, it is difficult to integrate these observations and form a unified understanding of transcriptional control via bursting dynamics.

Much of our quantitative knowledge about transcriptional bursting heavily relies on fixed data [3, 14, 23–26]. Capturing transcriptional bursts in vivo across space and time remains challenging [20, 27–29]. Adding to this challenge, live measurements need to be quantifiable in absolute units (i.e., mRNA count) to facilitate comparisons between different genes and conditions [30–32]. Moreover, to understand the entire spectrum of bursting dynamics, there is a need to probe the full dynamic range of a gene’s activity [25]. Measurements in an endogenous system where tightly regulated spatiotemporal transcriptional dynamics dictate cell-fate determination can further elucidate the functional consequences of bursts and their relation to different regulatory determinants. The early *Drosophila* embryo provides a unique system that meets all these requirements [33].

Here we quantify the endogenous transcription dynamics of key developmental genes in living *Drosophila* embryos. We identify a single control parameter, the instantaneous ON-probability of an allele as the dominant determinant of transcriptional activity, while the initiation rate is mostly conserved. This finding holds across spatial domains, developmental times, genes, and perturbations of *cis*-regulatory elements and *trans*-regulators. Surprisingly, we find a largely constant ON-OFF switching correlation time of roughly one minute. A corollary of the latter is that mean ON and OFF times are tightly coupled, regardless of the *trans*-environment or the *cis*regulatory architecture. While perturbations in the upstream regulatory processes lead to dramatic changes in the ON-probability, i.e., spatiotemporal changes in transcription rate, the underlying changes in ON and OFF periods are predicted from wild-type. Instead of a particular perturbation type dictating changes in specific bursting parameters, we observe that more generally lowly transcribing alleles are tuned to higher expression levels by increasing burst frequency, while highly transcribing alleles are mostly tuned by increasing burst size.These results imply that for the examined genes, the bursting phenomenon can be quantitatively understood by a few simple rules: two of the transcription parameters are quasi-constant, and all others are determined by the ON-probability. Hence, future investigations necessitate a re-examination of our mechanistic understanding of transcription, focusing on how regulatory processes influence a unique control parameter.

## RESULTS

### Instantaneous single allele transcription rate measurements.

**FIG. 1.**
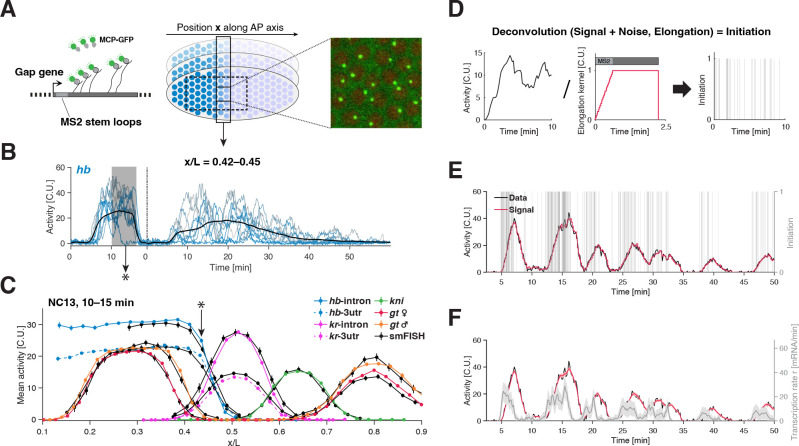
Live single-cell transcription rate measurements of endogenous gap genes. (A) Transcriptional activity measured of a single gap gene allele using a custom-built two-photon microscope in a living fly embryo. An 24xMS2 stemloop cassette is inserted in the first intron of the gap genes. Constitutively expressed MCP-GFP binds stem-loops formed on nascent transcripts, making transcription sites appear bright above background (green hotspots) and enabling quantification of transcriptional activity at single allele resolution along the anterior-posterior (AP) axis of the embryo. (For genomic strategy of all gap gene loci see Fig. S1A). (B) Example single allele transcription time series for the gene *hunchback* across nuclear cycles NC13 and NC14 (sampled every 10s) from a single AP bin (width ~ 2% embryo egg length L) at x/L=0.435±0.010. Intrinsically low embryo-to-embryo variability (compared to the total variance in our data, Fig. S1F) facilitated by the *Drosophila* system allows for pooling alleles from multiple spatially and temporally aligned embryos (n=10-20). Mean transcriptional activity (black line) obtained from pooling 200–350 alleles. (C) Calibration of transcriptional activity in absolute units performed by matching mean activity profiles in a 5-min-interval during NC13 (gray shade in B) from live (color) and previously calibrated fixed smFISH (black) measurements for all examined gap genes [25]. A global conversion factor (one for all genes) leads to a match within 5% error between live and fixed profiles (Fig. S1B), resulting in our activity unit, i.e., cytoplasmic unit (C.U.) equivalent to the intensity of a fully elongated transcript [34]. (D) Reconstruction of transcription initiation events from deconvolution of single allele transcription time series. The signal is modeled as a convolution between transcription initiation events and a kernel accounting for the elongation of a single Pol II through the MS2 cassette and the gene body (using an elongation rate $K_{\text{elo}\;}=1.8\;kb/min$, Fig. S2). Bayesian deconvolution is performed by sampling from the posterior distribution of possible configuration of initiation events given the measured activity and measurement noise (Fig. S1D–E). (E) Example deconvolved initiation configuration (gray bars) and corresponding reconstructed signal (red) from a single allele transcription time series (black). (F) single allele transcription rate (gray) from same allele as in E (black). The rate is estimated by counting the number of initiation events within 10s intervals for a given sampled configuration and averaged over 1’000 of such configurations. The displayed solid line and envelope for transcription rate (gray) and reconstructed signal (red) correspond to the mean and one standard deviation of the posterior distribution.

To study the principles that govern transcription dynamics across space and time, we need access to the endogenous bursting kinetics at a single allele level. We designed an approach to obtain such quantitative measurements in living *Drosophila* embryos [31, 35]. A versatile CRISPR-based scheme is employed to incorporate MS2 cassettes into the gap genes’ introns (or 3’UTR) [36]. These form stem-loops in the transcribed nascent RNA that are subsequently bound by fluorescent coat-proteins (Fig. 1A, S1A and Methods) [37, 38]. A custom-built two-photon microscope generates fluorescence images, capturing RNA synthesis at one tagged allele per nucleus with a 68-fold signal improvement over previous studies [31], approaching single-mRNA sensitivity (Fig. S1D–E). An optimized field-of-view yields 10s interval time-lapses for hundreds of nuclei per embryo, during nuclear cycles (NC) 13 and 14 (Fig. 1A–B; Videos V1-V4), essential for statistical analysis.

To achieve a fully quantitative characterization, we calibrate our measurements to absolute units. We convert the fluorescence signal at the site of transcription into equivalent cytoplasmic mRNA units (C.U.) by matching the mean transcriptional activity to previously calibrated smFISH measurements (Fig. 1B, see Methods) [25, 34]. We find high agreement between smFISH and live measurements, as a single conversion factor adjusts for the difference in fluorescence signal between the two methods, with an average relative error of ~ 5% across all gap genes and MS2 insertion sites (Fig. 1C and S1B). This agreement extends to higher moments (Fig. S1C), despite the fully orthogonal nature of these techniques: one being non-invasive genetically but involving fixation, while the other involves gene editing and stem-loop cassette insertions. This strongly suggests that our live approach captures the endogenous situation and provides means to express our dynamic transcription measurements in terms of absolute mRNA counts.

Our unique combination of absolute calibration and near single transcript sensitivity (Fig. S1D–E) allows us to reconstruct the underlying single allele transcription initiation events by individual Pol II, namely the events in which Pol II complexes are released onto the gene and engage in productive elongation. To infer these initiation events for each time series, we adopt a Baysian deconvolution approach that accounts for measurement noise (Fig. S1D and Methods). The convolution kernel models the fluorescent signal resulting from the Pol II elongation process through the stem-loop cassette (with constant and deterministic elongation, Fig. 1D) [20, 27]. For each time series, the approach generates multiple configurations of transcription initiation events (Fig. 1E). Averaging these configurations gives us a time-dependent instantaneous single allele transcription rate r(t) per time series (Fig. 1F).

We validate this kernel-based deconvolution approach by performing dual-color tagging of the gene body (a 5’ proximal intron and a 3’UTR tag). These measurements support our key assumptions (see Methods) and allow us to extract a Pol II elongation rate of $K_{\text{elo}\;}=1.8\pm 0.1\;kb/min$, which is in line with previous measurements [31, 39] (Fig. S2). Our inferred transcription rates are thus no longer masked by the Pol II elongation dwell time, unlike the directly measured intensities of transcriptional activity. Transcription rates are thus independent of gene length, enabling the direct comparison between different genes and opening a path to identify common principles underlying transcription dynamics.

### Single allele transcription rates hint at a universal bursting regime.

**FIG. 2.**
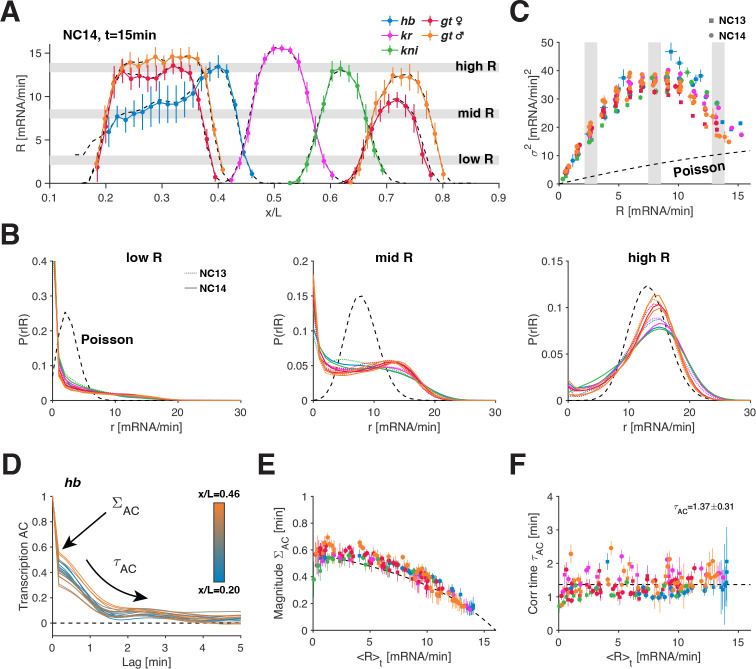
Transcription rates display signatures of a universal bursting regime. (A) Snapshot of mean transcription rates R as a function of AP position in early NC14 (t=15 min after mitosis) for different gap genes (color). AP profiles are obtained by averaging the deconvolved single allele transcription rates over ~ 200 nuclei within each AP bin. The black dashed lines correspond to the mean activity (Fig. 1C) of each gap gene at the same position and time normalized by the effective elongation time (see Methods, Fig. S3A and Fig. S4A). Tight agreement of colored and dashed profiles supports the deconvolution approach (error bars are one standard deviation across the means of 10–20 embryos). (B) Distribution P(r|R) of single allele transcription rates estimated within 1-min-intervals in both NC13 (color dotted lines) and early NC14 (color solid lines). These distributions are computed over all the nuclei from time points and AP bins whose mean transcription rate R is either in a low [2.1,3.2], mid [7.5,8.5] or high regime [12.8,13.9] (as gray shade in A and C). The various gap gene distributions collapse at all regimes and differ from the Poisson distribution (black dashed line), suggestive of a universal bursting regime. (C) Variance of single allele transcription rates as a function of mean transcription rate R in both NC13 (square) and early NC14 (circle) (estimated over 1-min-intervals). Note a strong departure of the variance from a constitutive Poisson regime (dashed line, $\sigma^{2}\sim R$). All gap genes follow the same trend suggestive of a common bursting regime. For higher moments see Fig. S3C. Vertical gray bars correspond to low, mid, and high R, as in A. (D) Auto-correlation (AC) functions of single allele transcription rates estimated within 10s intervals for hb in early NC14 and averaged across time, and within a given AP bin across alleles. Color code stands for position along the AP axis. The AC functions are normalized by the variance and highlight an uncorrelated and a time-correlated component in the single-cell transcription rate fluctuations. The correlated component is characterized by a magnitude $\sigma_{AC}$ and an exponential decay with time scale $\tau_{AC}$. Such correlated fluctuations are expected to arise in a bursting regime due to ON–OFF gene switching (Fig. S3D). (E) Magnitude $\Sigma_{AC}$ of the correlated fluctuations in the single allele transcription rate as a function of mean transcription rate R. All gap data (color) collapses showing a universal trend (dashed line, guide to the eye). The fraction of correlated variability decreases as R increases, as expected when approaching a constitutive regime of uncorrelated Poisson initiation (Fig. S3E). (F) Correlation time of the correlated fluctuations in the single allele transcription rate as a function of mean transcription rate R. The correlation times are obtained by fitting exponentials to the correlated component of the AC function as in D. Strikingly, the correlation time is mostly conserved across genes and transcription levels. Error bars are bootstrapped 68% confidence interval.

Before analyzing the gap genes’ transcription dynamics at the single allele level, we sought to determine whether the averages of the deconvolved single allele transcription rates r recapitulate the well-documented average protein dynamics [40]. We compute a mean transcription rate R=⟨r⟩ per gene along the anterior-posterior (AP) axis for all time points during NC13 and NC14 (Video V5); averaging occurs over 200–300 nuclei (each contributing one allele) in the same AP and time bin from 10–20 embryos (Fig. 1A and 1B). The extracted mean transcription rate profiles (Fig. 2A and S3A) strongly resemble the ones reconstructed from carefully staged gap gene antibody staining, including the well-documented posterior shifts during NC14. Indeed, with simple assumptions on diffusion and lifetime, the mean transcription rates R predict protein patterns with minimal post-transcriptional regulation (Fig. S4, Video V6). Thus, in this system, the rules governing transcription rate modulation will largely determine function, i.e., protein synthesis.

While the examined genes exhibit a common range of mean transcription rates R (Video V5), they display distinct spatiotemporal profiles, giving rise to gene-specific protein patterns. Yet when we examine the distributions of single allele transcription rates, r, underlying a similar mean transcription rate, P(r|R), we find that these distributions collapse across genes (Fig. 2B). Strikingly, for low- to mid-levels of R, the underlying distributions differ starkly from a constitutive regime, which would result in a Poisson distribution. At these levels, the large amounts of non-transcribing or barely transcribing alleles hint at quiescent OFF periods deviating from a constitutive regime. As the mean transcriptional activity increases, the distributions become more Poissonian, suggesting that $P_{ON}$, i.e., the probability of the genes being ON, increases. These observations are consistent with bursting behavior, where the gene alternates between ON and OFF states.

The collapse of our data and the deviation from a constitutive, Poisson regime, are readily observed also when we compute the relationships between R and the higher moments of the P(r|R) distributions (Fig. 2C and S3C). Our data approaches the Poissonian regime only on the extreme ends of the R spectrum, implying that the gap genes transition all the way from fully OFF $\left(P_{ON}=0\right)$ to fully ON $\left(P_{ON}=1\right)$. The gap genes thus provide an opportunity to investigate how an underlying bursting regime can account for a full dynamic range of transcriptional activities.

The dynamic nature of our measurement allows us to examine single allele transcription rates not only via distributions pooled across nuclei but also within individual transcription time series. We find the auto-correlation function of the single allele transcription rates provides further evidence for an underlying common bursting regime (Fig. 2D). An initial sharp drop of magnitude $1-\Sigma_{AC}$ at our sampling time scale (~ 10s) indicates the presence of uncorrelated noise, consistent with independent Pol II initiation events. This drop is followed by a longer decay of correlated noise at time scale $\tau_{AC}$. Such correlated noise is expected in a bursting regime, as the switching between ON and OFF states introduces temporal correlation in transcriptional activity. A theoretical and computational analysis using the two-state model of transcription [12] supports both the interpretation of the auto-correlation functions (Fig. S3D), and our ability to estimate $\Sigma_{AC}$ and $\tau_{AC}$ properly from the deconvolved rates (Fig. S3E–G).

We find both the magnitude of correlated noise $\Sigma_{AC}$ (Fig. 2E) and the correlation time $\tau_{AC}$ (Fig. 2F) collapse across AP bins and different genes. Notably, the magnitude $\Sigma_{AC}$ is highly constrained and drops at high R, consistent with the behavior of the variance (Fig. 2C). Furthermore, the correlation time $\tau_{AC}$ is largely conserved across nuclear cycles, across AP bins, and across genes, confined within the range of 1–2 min and averaging to a value of 1.37 ± 0.31 min (Fig. 2F). This surprising invariance of $\tau_{AC}$ with respect to R suggests that a key temporal characteristic of transcription dynamics is highly conserved. Thus, both the static moment and correlation-based analyses point to a common bursting regime, applicable across genes and space, motivating a time-dependent analysis of transcriptional bursts at the level of individual time series.

### Allele ON-probability is the key regulated transcriptional parameter.

**FIG. 3.**
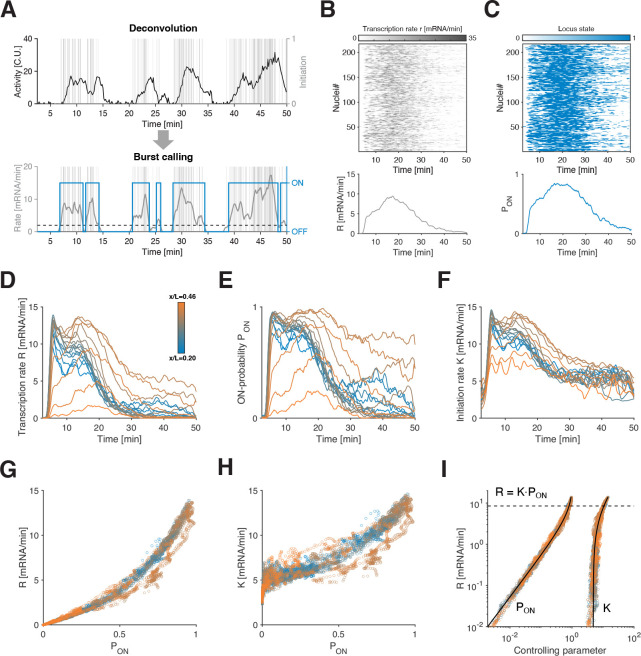
Direct estimation of instantaneous mean transcriptional parameters. (A) A simple clustering procedure on single allele transcription rate determines bursts of transcription. The rate (gray curve) is estimated using a moving window of width ~ 1 min over the deconvolved initiation events (gray vertical bar). A threshold at two mRNA/min (black dashed line) applied on the rate identifies individual bursts (blue curve). (B-C) Heatmaps of deconvolved single allele transcription rates (B) (estimated over 10s intervals) and of corresponding ON-OFF periods (C) (obtained from burst calling) as a function of time during NC14 for N=217 nuclei expressing hb-MS2 at AP position x/L=0.43. Instantaneous mean transcription parameters such as transcription rate R (B, bottom) and ON-probability $P_{ON}$ (C, bottom) are obtained by vertically averaging the heatmaps (top) over all nuclei, respectively. (D-F) hb transcription rate R (D), ON-probability $P_{ON}$ (E), and initiation rate K (F) as a function of time in NC14 for all AP positions (color coded). R and $P_{ON}$ are computed as in B and C, respectively, and K is obtained by averaging the single allele transcription rate (B) conditioned on the locus being ON (C) over all nuclei in each AP bin. (For tests of burst calling procedure on simulated data see Figure S3.) (G-H) Transcription rate R (G) and initiation rate K (H) as a function of $P_{ON}$, for all time points and positions, demonstrating a massive data collapse, suggesting that $P_{ON}$ is the central regulatory parameter for transcriptional bursting. (I) Transcription rate R as a function of both controlling parameter $P_{ON}$ and K in log-space. Since $log(R)=log(K)+log\left(P_{ON}\right)$ by construction, changes in $P_{ON}$ determine changes in R below the dashed line (R~8.5 mRNA/min, corresponding to $P_{ON}=0.75$).

To directly quantify individual transcriptional bursts at the single allele level, we take advantage of our deconvolved initiation events and instantaneous transcription rate time series that are unencumbered by the signal-blurring effect of elongation (Fig. 3A). They allow us to identify distinct periods of active transcription, characterized by consecutive initiation events (i.e., multiple Pol IIs released into productive elongation), interpreted as ON periods, followed by quiescent periods, namely OFF periods (Fig. 3A). We define the switch of an allele from an OFF to an ON state when a moving average of the single allele transcription rate exceeds 2 mRNA/min (see Methods). This threshold is consistent with our detection sensitivity of 1–2 mRNAs, and the size of the moving window for averaging is set based on the correlation time scale from the auto-correlation analysis. The main strength of our burst calling routine is its sole reliance on a minimal clustering model, and as such being devoid of any mechanistic assumptions on the underlying bursts (no explicit mechanistic model is needed, see Methods).

Given a computed bursting profile (demarcated ON and OFF periods) for every single allele, we can now ask how bursting dynamics underlie transcription rates. Specifically, the mean transcription rate at time t,R(t), can be decomposed into two parameters: the instantaneous probability of an allele being in the ON state $P_{ON}(t)$ (i.e., the fraction of ON alleles) and the mean initiation rate in the ON state K(t). Starting with the gene hb, we thus estimate, for a given AP bin, the time-dependent parameters R(t) and $P_{ON}(t)$ by averaging all (~ 250) single allele instantaneous transcription rates and counting the fraction of alleles in the ON state at time t, respectively (Fig. 3B–C). To compute K(t), we average initiation events restricted to the ON state (as opposed to R, which is averaging initiation events regardless of allele state). We repeat this procedure for each position of the AP-axis to obtain the full spatiotemporal dependence (Fig. 3D–F). We validate our approach for burst calling and the recovery of bursting parameters from transcription time series on simulated data (based on a 2-state model) with an overall median error of 10% (see Methods, Fig. S5 and Fig. S6).

All three parameters vary significantly across both space and time (Fig. 3D–F). However, given that these are related by $R=K\cdot P_{ON}$ (Fig. S7A), it is possible that most of the variation in R stems from changes to either K, or $P_{ON}$, or both. When R is plotted against $P_{ON}$, all data points across time and space collapse in a tight monotonically increasing function (Fig. 3G), with $P_{ON}$ spanning from 0 to 1 (fully OFF to fully ON), echoing back to the noise analysis above (Fig. 2B–C). Similarly, the initiation rates K tightly collapse across space and time when plotted against $P_{ON}$. However, K only covers a two-fold change in dynamic range (Fig. 3H), which is marginal compared to R spanning from 0 to 15 (mRNA/min) (Fig. 3I and S7B–C). This two-fold change is largely due to the existence of two optically unresolved sister chromatids and a modest time dependence of K throughout the nuclear cycle (Fig. S7D–I, see Methods). With these considerations we estimate the mean Pol II spacing for a single active chromatid at 303±73 bp, consistent with the classic Miller spreads with average Pol II spacing of 330 ± 180 bp [41].

Overall, we find that R is tightly controlled by $P_{ON}$, while K is only moderately modulated and has significantly less predictive power over R. These results for hb suggest that transcriptional activity is mainly controlled through the probability of an allele being in the ON state. Once in the ON state, transcription initiates at a quasi-constant rate.

### Constant switching correlation time restricts ON and OFF periods.

**FIG. 4.**
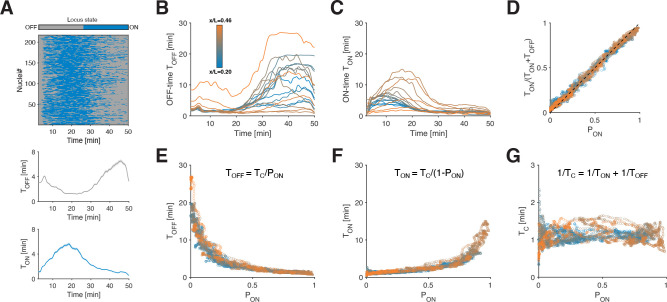
Allele ON-probability controls ON and OFF times. (A) Binarized heatmap from Fig. 3C. Instantaneous mean OFF-time $T_{OFF}$ (bottom, gray) and mean ON-time $T_{ON}$ (bottom, blue) are obtained by the weighted average of the ON and OFF times over all nuclei (see methods). The weights are given by the inverse of the number of time points within each period. (B-C) Mean OFF-time $T_{OFF}$ and mean ON-time $T_{ON}$ as a function of time and position (color coded) for hb in NC14. (D) The ratio of $T_{ON}$ over the sum of $T_{ON}$ and $T_{OFF}$ versus ON-probability $P_{ON}$ for all positions and time points beyond the 7.5 min mark in B and C (near-steady state both quantities are expected to be equal after initial transient, see Fig. S7J–K). Thus, temporal changes in transcriptional parameters must be slow enough to allow relaxation. (E-F) Mean OFF-time $T_{OFF}$ (E) and mean ON-time $T_{ON}$ (F) as a function of $P_{ON}$, for all positions and time points beyond the 7.5 min mark in B and C. (G) Effective switching correlation time $T_{C}$ (defined as: $1/T_{C}=1/T_{ON}+1/T_{OFF}$ as a function of $P_{ON}$, computed using data points in E and F. $T_{C}$ is mostly conserved across time points and position and is $P_{ON}$ independent.

Given the central role of $P_{ON}$ in controlling hb’s transcription rate, we aim to examine how it decomposes into ON and OFF periods in individual alleles. To this end we compute the mean ON and OFF times, averaged across alleles of the same AP bin, and at a given time ($T_{ON}$ and $T_{OFF}$, Fig. 4A–C; see Methods). Near steady state, we expect the ON-probability $P_{ON}$ to be given by the ratio of $T_{ON}$ and $T_{ON}+T_{OFF}$. We verify this relationship (Fig. 4D), showing good agreement (i.e., beyond an initial 7.5 min transient post mitosis, Fig. S7J–K). This is a strong indication of the self-consistency of our general approach for extracting these bursting parameters. Moreover, despite temporal changes in $P_{ON}$ due to developmental regulation, transcriptional bursting in this system seems to operate in a near-steady-state regime.

While $T_{ON}$ and $T_{OFF}$ change over time and in different AP bins (Fig. 4B–C), when we plot $T_{ON}$ and $T_{OFF}$ against $P_{ON}$ all data points collapse again across time and space onto two tight anti-symmetric relationships (Fig. 4E–F). Various combinations of $T_{ON}$ and $T_{OFF}$ could potentially give rise to any given $P_{ON}$, however, here we observe a highly restricted range for these mean durations. Hence a given $P_{ON}$ is unequivocally linked to a specific pair of $T_{ON}$ and $T_{OFF}$, regardless of space and time.

An allele switching dynamically between ON and OFF states will have a correlation time $T_{C}$, which determines, on average, the time needed for the single allele transcription rate to become uncorrelated. For such a system, $T_{C}$ can be computed directly from the mean ON and OFF times and is defined by $1/T_{C}=1/T_{ON}+1/T_{OFF}$ (Fig. 4G, see Methods). Surprisingly, for all AP and time bins, $T_{C}$ is confined between 1–1.5 min and thus largely constant and independent of $P_{ON}$. Moreover, $T_{C}$ matches quantitatively the correlation time $\tau_{AC}$ from the autocorrelation analysis (Fig. 2F), which we found to be independent of the transcription rate. Given that $T_{C}$ characterizes allele switching correlations by construction, this match suggests that the nature of $\tau_{AC}$ could indeed be related to bursting.

The fact that $T_{C}$ seems to remain conserved across space and time restricts the mean ON and OFF times. Indeed, $T_{ON}$ and $T_{OFF}$ can be expressed as a function of $P_{ON}$ and $T_{C}$ (via $T_{ON}=T_{C}/1-P_{ON}$ and $T_{OFF}=T_{C}/P_{ON}$, since $P_{ON}=T_{ON}/\left(T_{ON}+T_{OFF}\right)$ near the steady state, cf. Fig. 4D). Thus, the constancy of $T_{C}$ mathematically explains the tight anti-symmetric relationships of $T_{ON}$ and $T_{OFF}$ with respect to $P_{ON}$ (Fig. 4E–F), so that $P_{ON}$ not only governs the mean transcription rate R but also the entire transcriptional bursting dynamics.

### Common bursting relationships underlie the regulation of all gap genes.

Should we expect these bursting parameter relationships found for hb to generalize to other gap genes or are they gene-specific? The gap genes differ in their *cis*-regulatory elements, namely different numbers and arrangement of enhancers and promoters (Fig. S1A), and different compositions of transcription factors binding sites within each enhancer. Correspondingly, and as discussed above, the gap genes display distinct transcriptional activities along the body axis (Fig. 1C) and across time (Video V8), which, in the case of hb we found to be largely governed by $P_{ON}$.

**FIG. 5.**
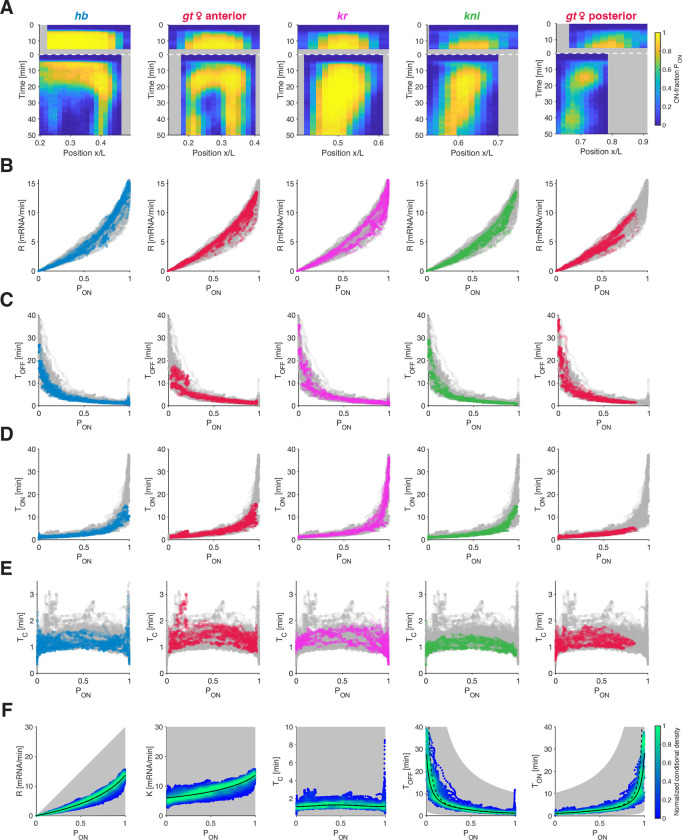
Transcriptional parameters collapse for all gap genes. (A) Kymographs of ON-probability $P_{ON}$ for all gap genes as a function of position and time for NC13 and NC14. The spatiotemporal transcriptional pattern of the gap genes arises from a complex regulation of $P_{ON}$ (color map). (B-E) Transcriptional parameters collapse for all gap genes across time and position. Transcription rate R (B), Mean OFF- (C) and ON-time (D) ($T_{OFF}$ and $T_{ON}$, respectively), and switching correlation time $T_{C}$ (E) as a function of the ON-probability $P_{ON}$. Colored data points represent individual gap gene (same color code as in A, see Fig. S8A–B for gt male data); underlying is the remaining data of all other genes (gray). (F) Density of all data points across space and time (NC13 and NC14) of transcriptional parameters for all gap genes. Color code represents the kernel density estimate of the parameters conditioned on $P_{ON}$ and normalized by the maximum density. Putative accessible space (gray shade) for plausible ranges of K (0.1–30 mRNA/min) and $T_{C}$ (0.1–10 min). Due to the constancy of $T_{C}$ and the small changes in K (2-fold at most), $P_{ON}$ almost fully determines R and dictates the combinations of $T_{OFF}$ and $T_{ON}$. For $T_{OFF}$ and $T_{ON}$, the dashed lines stand for the 2-state model prediction based on $T_{C}$, and the solid lines take the finite recording-length into account (see Figure S8D).

When we apply our deconvolution and burst calling procedure (Fig. 3A) to the measured single allele transcription time series of other gap genes (gt,Kr, and kni), we observe that all genes differ substantially in their spatiotemporal $P_{ON}$ profiles (Fig. 5A). These differences are expected and reflective of the above-mentioned distinct underlying *cis* architectures and *trans* regulators of these genes. Strikingly, despite these differences, they show an almost identical mean transcriptional rate R to $P_{ON}$ relationship (and K to $P_{ON}$ relationship, Fig. S8C), substantiating $P_{ON}$ as the governing factor for the transcriptional activity not only across time and space but also across genes (Fig. 5B).

Computing the various bursting parameters for all gap genes and plotting these as a function of $P_{ON}$, we find the genes display the same $P_{ON}$-dependent relationships: all genes share common $T_{OFF}$ to $P_{ON}$ (Fig. 5C) and $T_{ON}$ to $P_{ON}$ (Fig. 5D) relationships. Thus, when different genes display a specific $P_{ON}$ value, possibly at different spatiotemporal coordinates, the underlying $T_{ON}$ and $T_{OFF}$ periods employed are nonetheless largely similar. This finding can be related to the switching correlation time $T_{C}$, which we find to be conserved across genes (Fig. 5E). The average $T_{C}$ value across all genes, positions, and times is 1.25±0.37 min, very close to the prediction from the single rate auto-correlation analysis (Fig. 2F).

Pooling all our data across all genes, times, locations, and embryos ($N>10^{6}$ data points) and plotting each of the computed bursting parameters ($R,\;K,\;T_{C},\;T_{OFF}$ and $T_{ON}$) against $P_{ON}$ (Fig. 5F) – including the oftenused burst frequency $F=1/\left(T_{ON}+T_{OFF}\right)$ and burst size $B=K\cdot T_{ON}$ (Fig. S8E) – reveals highly constrained relationships. Indeed, all data points occupy only a very small subset of the parameter space (see methods). Tight functions can be mapped out (black lines) that confirm the exceptional predictive power conferred by $P_{ON}$. Separating all the data into three developmental time windows (NC13 & early NC14, mid NC14, and late NC14), shows a further tightening of the relationships in these developmental stages (Fig. S9). This separation thus confirms that parts of the dispersion results from slow and moderate changes in K (and to a lesser extent $T_{C}$) over developmental time (at most 40% reduction of K and 25% for $T_{C}$).

All measured bursting dynamics seem to adhere to this simple set of rules, across genes, space, and time. The mean transcription rate R is essentially dictated by $P_{ON}$, with a largely constant K. A near-constant switching correlation time $T_{C}$ leads to a specific functional relationship for $T_{ON}$ and $T_{OFF}$ of inverse proportionality. Thus, only one of these two parameters is principally modulated and the other remains quasi-constant. While lowly transcribing alleles are tuned to higher expression levels predominantly by decreasing $T_{OFF}$, medium-to-high transcribing alleles are mostly tuned by increasing $T_{ON}$. These simple rules contain all information necessary to govern bursting and consequently transcriptional activity in the system.

### The common bursting relationships predict the effects of *cis*- and *trans*-perturbations.

Diverse regulatory mechanisms have been implicated in the control of transcriptional activity, including *cis*-regulatory elements (e.g., enhancers) and *trans*-factors (e.g., TF repressors). It is often assumed that distinct regulatory mechanisms directly control distinct bursting parameters. Will the established bursting parameter relationships based on wild-type measurements predict bursting dynamics when we perturb regulatory mechanisms?

**FIG. 6.**
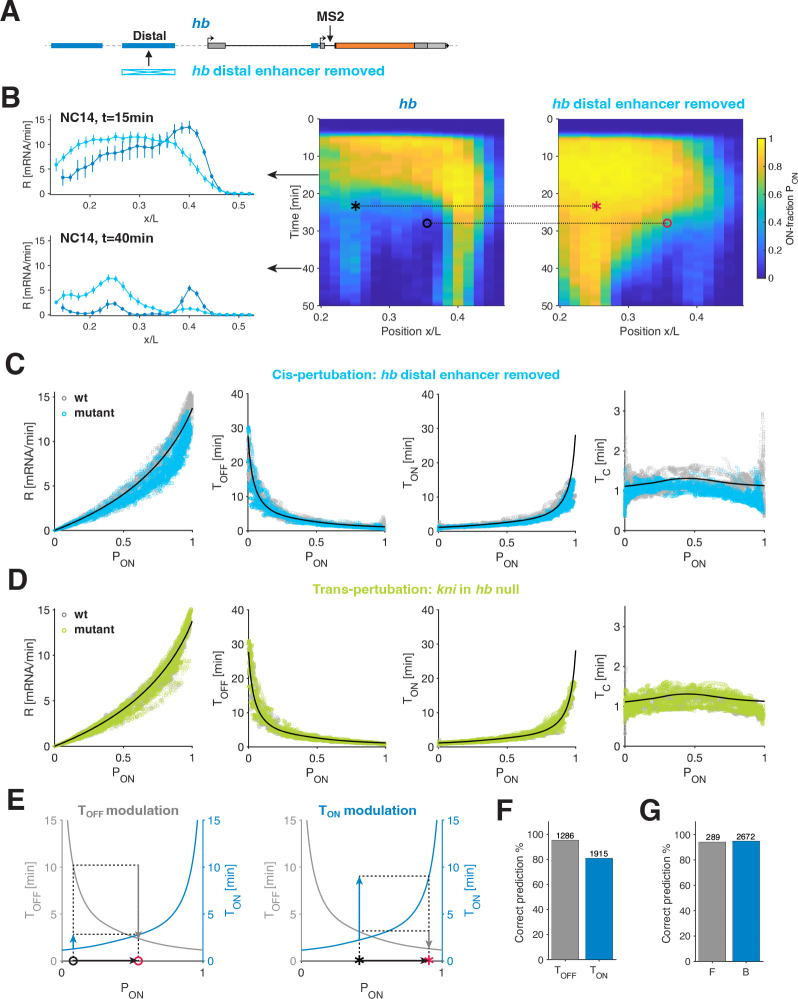
Modulation of ON and OFF times by *cis*- and *trans*-perturbations. (A) Distal hb enhancer removal. The MS2-stem loops are inserted at the same location in the mutant (enhancer deletion) and wild-type fly lines. (B) Quantification of hb wild-type and mutant (A) phenotypes. Both transcriptional rate R (left) and $P_{ON}$ level (right, kymograph) display significantly different expression patterns in the mutant. Dotted arrow indicates time point in the kymograph at which rate profiles are depicted. “o” and “⋆” mark signal two bins with predominant $T_{OFF}$ modulation and predominant $T_{ON}$ modulation, respectively. (C-D) Transcriptional parameters for hb in *cis*-mutation (C, cyan) and for kni in *trans*-mutation (D, light green) collapse on corresponding wild-type parameters (gray). *cis*-mutation is the hb distal enhancer removal from A and the *trans*-mutation is a hb null background compared to wild-type (Fig. S10D–E). Solid black lines correspond to the endogenous bursting rules from Fig. 5F. (E) Example for $T_{OFF}$ and $T_{ON}$ modulation observed upon hb enhancer removal in B (circle and star, respectively). Depending on the pair of wild-type and mutant $P_{ON}$ chosen at the same spatiotemporal location (two examples: circle and star in B), the derived endogenous rules (gray and blue solid lines) predict either larger fold-change in $T_{OFF}$ compared to $T_{ON}$ (left, $T_{OFF}$ dominated modulation) or larger fold-change in $T_{ON}$ (right, $T_{ON}$ dominated modulation). Verifying these examples using the estimated $T_{OFF}$ and $T_{ON}$ (in C) confirms that both types of modulation can be observed for the same perturbation. (F) Verification of predicted $T_{OFF}$ and $T_{ON}$ modulation for all hb wild-type and mutant $P_{ON}$ pairs (defined as in E), for most pairs (> 85%) the prediction is correct (see Fig. S11A–C). (G) Verification of predicted burst size B and frequency F modulation for all hb wt and mutant $P_{ON}$ pairs. As in E, B versus F modulation can be predicted for each $P_{ON}$ pair based on the derived endogenous rules. For most pairs (> 95%) the prediction is correct (see Fig. S11G–I).

To address this question, we devised a strategy to perturb the endogenous system in *cis* and in *trans*. For *cis*-perturbations, we delete a distal hb enhancer from the hb locus that has an MS2-stem loop cassette in the first intron (Fig. 6A). This enhancer removal has a complex effect on hb activity, including increased and decreased transcriptional activity at different times and AP locations (Fig. 6B), consistent with previous observations [42–44]. Despite the stark deviation in the mutant spatiotemporal transcriptional activity compared to the wild-type, we find that mean transcription rates across space and time are governed by $P_{ON}$. Thus, the predictive power of this parameter observed for the wild-type holds for the mutant as well (Fig. 6B). The mutant further adheres to the other bursting relationships identified in the wild-type. In particular, the restrictive $T_{ON}$ and $T_{OFF}$ to $P_{ON}$ relationships hold, as well as the largely conserved switching correlation time $T_{C}$ (Fig. 6C).

A second enhancer deletion, namely, the removal of the kni distal enhancer results in a significant reduction in kni activity. The mutant samples have a smaller dynamic range of activity, yet we find a similar data collapse within that range (Fig. S10A–C). Finally, to examine a *trans*-perturbation, we measure kni activity in embryos with a hb null background. kni activity was substantially altered, consistent with earlier studies [45] (Fig. S10D–E). Yet, again, we observe the collapse of the mutant bursting parameters onto the wild-type busting rules (Fig. 6D).

The consistency of these bursting rules suggests that the wild-type derived relationships (Fig. 5F) can predict how changes in $T_{ON}$ and $T_{OFF}$ account for the change in transcriptional activity upon the perturbation. Specifically, to examine the perturbation’s effect on transcriptional activity at a given AP bin and time in development, we can consider the pair $\left\{P_{ON}^{wt},P_{ON}^{\text{mut}\;}\right\}$ at that spatiotemporal position (two examples of such pairs are marked in the kymographs in Fig. 6B). Using the dependencies of $T_{ON}$ and $T_{OFF}$ on $P_{ON}$, we predict whether the change in transcriptional activity (i.e., the change from $P_{ON}^{wt}$ to $P_{ON}^{mut}$) stems predominantly from a change in $T_{ON}$ or in $T_{OFF}$ (Fig. 6E). For our two example pairs, one is predicted to be mostly governed by $T_{OFF}$ (“o” mark), while the other is mostly governed by TON (“⋆” mark). This exercise can be generalized to all $\left\{P_{ON}^{wt},P_{ON}^{mut}\right\}$ pairs and subsequently verified using the measured $T_{ON}$ and $T_{OFF}$ values for wild-type and the perturbations (Fig. 6C–D and Fig. S11A–C). We find that over ~ 90% of the pairs correctly verify the type of modulation predicted by our rules (Fig. 6F). We reached the same conclusions predicting changes in burst size and burst frequency (Fig. 6G and S11G–I). Importantly we find that each type of perturbation displays both predominant $T_{ON}$ or $T_{OFF}$ modulation at different times and positions.

The generalization of our bursting rules to *cis*- and *trans*-perturbations have strong consequences. From our examination, the type of perturbed regulatory mechanism can hardly be linked to changes in a specific bursting parameter and vice versa. Indeed, as is the case for wild-type, $P_{ON}$ emerges as the main governing parameter of the transcriptional activity also for the mutants, while K and $T_{C}$ remain largely unchanged. $P_{ON}$ changes upon a perturbation (i.e., wild-type to mutant) are sufficient to determine the corresponding changes in $R,\;T_{ON}$, and $T_{OFF}$. Moreover, the functional form of the relationships implies that the $P_{ON}$ regime (low versus high) is crucial in determining which parameter is predominantly affected. Different regulatory mechanisms have a different propensity for $P_{ON}$ modulation. Yet, the single-parameter regulation set in place by the identified bursting rules points towards general mechanisms conserving K and $T_{C}$, and linking $T_{ON}$ and $T_{OFF}$.

## DISCUSSION

While it is appreciated that mRNA production likely occurs in bursts across various systems, quantitatively measuring endogenous, single allele transcriptional bursting in real-time, across a wide range of gene activities, still poses a challenge. This hinders our understanding of how the kinetic parameters of bursting underlie transcriptional dynamics across genes, space, and developmental time. In this study, we devised an approach to perform such measurements in the context of the developing early *Drosophila* embryo, a system that relies on large changes in transcription rates, as a means to govern protein abundance (Fig. S4A). The spatiotemporal transcriptional activity of the examined genes is regulated by a myriad of processes (e.g. repressor and activator binding, chromatin accessibly, PIC formation and pause-release, histone modifications, etc.) [42, 46–48]. These processes are mediated by gene-specific *cis*-architectures, with distinct combinations of enhancers and promoters, further differing in their internal motif compositions. Surprisingly, despite the complexity of the regulatory processes involved and the differences between the genes, we find highly restricted, unifying properties of the underlying bursting dynamics.

We observed that the mean transcription rate is governed by a single tunable control parameter, the ON-probability ($P_{ON}$), with a near-constant Pol II initiation rate (K). We further found a conserved time scale ${(T}_{C})$ of about a minute, over which the allele states, either active (ON) or inactive (OFF), remain correlated. This time constant explicitly links the mean durations of ON and OFF periods that are thus largely determined by the control parameter ($P_{ON}).\;P_{ON}$, together with the mostly conserved K and $T_{C}$, fully parameterize the observed bursting dynamics, across genes, space, and developmental time.

While consistent with predictions from our previous measurements on fixed tissues (Fig. S12A) [25], the presented live measurements allow us to go beyond a model-based inference of kinetic parameters from a static snapshot of distributions of nascent transcripts. Our current approach measures the dynamics directly rather than inferring the kinetics indirectly. Therefore, we are relieved from the constraints imposed by a specific mechanistic model and can thus further relax the steady state assumption. Both aspects are often used in the analyses of fixed and live data [3, 6, 25, 49]. Moreover, access to the full dynamics of individual transcription time series (throughout more than 1 hour of development) allows us to estimate time-dependent transcriptional parameters from the underlying bursts, rather than obtaining only a single transcriptional parameter value per condition.

Using synthetic data, we verify the capacity of this procedure to reliably recover a wide range of bursting parameters, including the estimation of $T_{C}$ (Fig. S5C–D). The conserved nature of $T_{C}$ and its value (i.e., 1.25±0.35 min as estimated from individual bursts) quantitatively agree with the auto-correlation analysis ($\tau_{AC}=1.37\pm 0.31\;min$), an orthogonal approach not involving burst calling. It is intriguing to consider the functional consequences of a constant correlation time $T_{C}$ and of the relatively small measured value. $T_{C}$ not only sets the time scale of the bursting dynamics, linking the mean ON and OFF times, but it has further implications on transcription noise filtering: a small $T_{C}$ value minimizes noise as bursts are easily buffered by long mRNA lifetimes. A small $T_{C}$ further allows gene transcription to respond more rapidly to input TF changes, by means of facilitating a fast relaxation to a steady state (Fig. S7J–K).

**FIG. 7.**
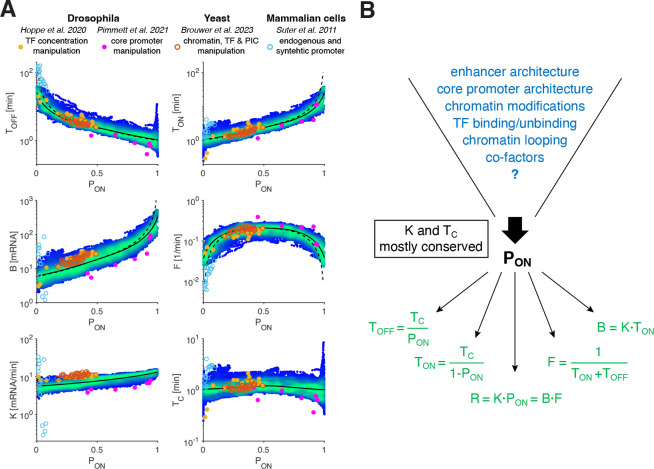
Decoupling between mechanism and response points to unifying rules. (A) Scatter plot of the transcriptional parameters as a function of $P_{ON}$ (color code same as Fig. 5F). Transcriptional parameters from two other *Drosophila* studies are largely consistent with uncovered transcription rules (black lines); one study investigates response to BMP signaling (yellow circles; Hoppe et al.), the other the effect of core promoter manipulations on a transgene (pink circles; Pimmet et al.). Transcriptional parameters resulting from multiple perturbations performed on yeast *GAL10* gene all closely follow the rules (orange circles; Brouwer et al.). Besides the initiation rate K, transcriptional parameters inferred from mammalian promoters (cyan circles; Suter et al.) appear mostly compatible with the rules, suggesting that these may apply beyond *Drosophila*. A minimal description of bursts requires three independent parameters, such as initiation rate K, switching correlation time $T_{C}$, and ON-probability $P_{ON}.\;P_{ON}$ is the main regulated parameter controlling the transcription rate R, while K only accounts for small changes (at most 2-fold), and the correlation time $T_{C}$ sets a conserved bursting time scale. Conservation of both K and $T_{C}$ implies that $T_{OFF}$ and $T_{ON}$, or, alternatively, the burst size B and burst frequency F, are fully determined by $P_{ON}$. Thus, whether changes in R are mediated by $T_{OFF}$ versus $T_{ON}$, or B versus F dominated modulation only depends on $P_{ON}$. (B) While K and $T_{C}$ appear mostly conserved, $P_{ON}$ emerges as the key regulatory parameter. It is independent of gene identity (different *cis*-architecture) or the combination of input transcription factor concentrations (that vary along the AP axis), validated by *cis*- and *trans*- perturbations and by data from other studies. Such invariance suggests a mechanistic decoupling, where regulation affects solely $P_{ON}$, which in turn determines unequivocally all of the underlying bursting dynamics.

The identified bursting rules point to a surprising predictive power of $P_{ON}$ on the mean ON and OFF times (Fig. 5F). This observation is only possible because our system allows for the quantification of bursting parameters across a large dynamic range of transcriptional activity, with $P_{ON}$ values ranging from fully off (0) to fully on (1) for most of our examined genes and conditions. This leads us to uncover a strict relationship between $T_{OFF}$ and $T_{ON}$ (Fig. 6E and Fig. 7A). It is commonly thought that distinct regulatory processes will alter transcriptional activity by predominant regulation of specific bursting parameters (e.g., the mean ON durations of a burst versus the mean OFF intervals in between bursts). Yet, when we perform a *cis*- or *trans*-perturbation, which substantially alters transcriptional activity, the predominantly modulated bursting parameters ($T_{OFF}$ versus $T_{ON}$ or burst frequency versus burst size) can be predicted by the wild-type and mutant $P_{ON}$ regimes, rather than by the type of the perturbation performed (Fig. 6E–G and S11).

To further examine the generality of these results, we investigated previously published transcription measurements in the early fly embryo. These measurements include both endogenous genes and synthetic reporters, where transcription was altered by varying BMP signal (a dorsoventral morphogen) [28] or core promoter composition [20]. Strikingly, we find that these datasets collapse on our established bursting rules (Fig. 7A). As suggested in these studies, the first dataset shows mainly effects on $T_{OFF}$, while the latter principally changes $T_{ON}$. Intriguingly, the two independent datasets cluster in disjoint halves of the full spectrum of $P_{ON}$ values captured by our measurements. Our analysis thus raises the possibility that the predominantly changed parameter ($T_{OFF}$ versus $T_{ON}$) might not be inherent to the examined regulatory manipulation (e.g., input TF concentrations or core promoter elements), but rather a consequence of the limited expression range of these genes.

The striking manifestation of general rules underlying bursting parameters in the *Drosophila* embryo, as well as the conserved nature of regulatory processes and the transcription machinery across eukaryotes, naturally leads to the question of whether our rules apply more broadly [50]. Recent measurements of an extensively perturbed yeast gene [21] provide an equivalent set of bursting parameters that faithfully adhere to our transcription rules (Fig. 7A). Although, the yeast gene initiation rate K appears mostly constant across conditions (similar to the fly genes), calibration of its value was only possible under specific hypotheses leading to a lower bound that needs to be further tested (Methods). Earlier yeast gene data have shown a significantly smaller initiation rate [5, 38]. A comprehensive study allowing multiple gene classes across multiple organisms will be necessary to verify whether our rules hold more generally in eukaryotes.

Mammalian genes are often lowly transcribed [6, 29, 51], potentially exploring a different parameter regime. Indeed, while bursting parameters inferred from measurements of a luminescent reporter protein [6] are restricted to very small $P_{ON}$ values (0 to 0.05), they appear to be consistent with our established relationships (Fig. 7A). Again, only the initiation rate K seems to deviate. Possible deviations in K across species leave open the possibility that the overall levels of K might be linked to species-specific rates, such as those linked to metabolism [52, 53]. Mammalian time-lapse data with a broader $P_{ON}$ range will be necessary to make a stronger parameter comparison possible.

Revisiting previously performed genome-wide studies in mammalian systems shows trends that are possibly compatible with our established bursting relationships. Similar to our fly genes (Fig. 7A), one study found that while burst frequency is predominantly modulated for low expressing genes, burst size is tuned for high expressing ones, independent on the reporter control sequences [13]. Another study using single-cell RNA-seq found a functional dependence of burst frequency and burst size on mean expression that seems compatible with our established rules (for $P_{ON}<0.5$) [51]. However, scRNA-seq parameters are significantly influenced by mRNA loss and long mRNA lifetimes, making the mapping of the sets of units between the vastly different approaches challenging.

The potentially wider applicability of our bursting relationships to other species calls for a new framework underlying the regulatory processes governing transcription. Instead of specific regulatory processes being inherently linked to specific bursting parameters, the tuning of transcription seems to be funneled through the sole control of $P_{ON}$, that all regulatory processes act on (Fig. 7B). Future investigations will have to determine the molecular mechanisms that can implement such a funneling at the level of $P_{ON}$ control. How do diverse processes tune $P_{ON}$? How is the constancy of the switching correlation time $T_{C}$ implemented molecularly? As for the latter, our work suggests that a highly conserved and general mechanism across eukaryotic genes should be at work. The generality of such a mechanism implies independence from the particularity of a given gene locus and thus could rather be implemented by the environment, including structural consideration of the nuclear architecture [54–56] or the molecular assembly of components of the transcription machinery [57, 58].

Despite the complexity of transcriptional dynamics across species, genes, space, developmental time, and perturbations, our quantitative real-time measurements revealed strict bursting rules, that set strong constraints on mechanistic models of transcriptional regulation. Our work also has some indicators for the generality of these rules across systems, their functional implications, and their molecular underpinning. As is the case with other areas in which organizing principles are increasingly emerging [59–63], these rules offer new ways to think about complex processes and point to conserved mechanisms at their core.

## Supplementary Material

Supplement 1

## References

[1] Lelli K. M., Slattery M., and Mann R. S., Disentangling the many layers of eukaryotic transcriptional regulation., Annual Review of Genetics 46, 43 (2012). 10.1146/annurev-genet-110711-155437 PMC4295906 22934649

[2] Cramer P., Eukaryotic transcription turns 50, Cell 179, 808 (2019). 31675494 10.1016/j.cell.2019.09.018

[3] Raj A., Peskin C. S., Tranchina D., Vargas D. Y., and Tyagi S., Stochastic mRNA synthesis in mammalian cells., PLoS biology 4, e309 (2006). 17048983 10.1371/journal.pbio.0040309 PMC1563489

[4] Chubb J. R., Trcek T., Shenoy S. M., and Singer R. H., Transcriptional Pulsing of a Developmental Gene, Current Biology 16, 1018 (2006). 16713960 10.1016/j.cub.2006.03.092 PMC4764056

[5] Zenklusen D., Larson D. R., and Singer R. H., Single-RNA counting reveals alternative modes of gene expression in yeast., Nature Structural & Molecular Biology 15, 1263 (2008). 10.1038/nsmb.1514 PMC3154325 19011635

[6] Suter D. M., Molina N., Gatfield D., Schneider K., Schibler U., and Naef F., Mammalian genes are transcribed with widely different bursting kinetics., Science 332, 472 (2011). 21415320 10.1126/science.1198817

[7] Bothma J. P., Garcia H. G., Esposito E., Schlissel G., Gregor T., and Levine M., Dynamic regulation of eve stripe 2 expression reveals transcriptional bursts in living drosophila embryos., Proc. Natl. Acad. Sci. USA 111, 10598 (2014). 24994903 10.1073/pnas.1410022111 PMC4115566

[8] Tantale K., Mueller F., Kozulic-Pirher A., Lesne A., Victor J.-M., Robert M.-C., Capozi S., Chouaib R., Bäcker V., Mateos-Langerak J., Darzacq X., Zimmer C., Basyuk E., and Bertrand E., A single-molecule view of transcription reveals convoys of RNA polymerases and multi-scale bursting, Nature Communications 7, 12248 (2016). 10.1038/ncomms12248 PMC4974459 27461529

[9] Nicolas D., Phillips N. E., and Naef F., What shapes eukaryotic transcriptional bursting?, Molecular BioSystems 13, 1280 (2017). 28573295 10.1039/c7mb00154a

[10] Tunnacliffe E. and Chubb J. R., What Is a Transcriptional Burst?, Trends in Genetics 36, 288 (2020). 32035656 10.1016/j.tig.2020.01.003

[11] Rodriguez J. and Larson D. R., Transcription in Living Cells: Molecular Mechanisms of Bursting, Annual Review of Biochemistry 89, 1 (2020). 10.1146/annurev-biochem-011520-105250 32208766

[12] Peccoud J. and Ycart B., Markovian modeling of geneproduct synthesis, Theoretical Population Biology 48, 222 (1995).

[13] Dar R. D., Razooky B. S., Singh A., Trimeloni T. V., McCollum J. M., Cox C. D., Simpson M. L., and Weinberger L. S., Transcriptional burst frequency and burst size are equally modulated across the human genome., Proc. Natl. Acad. Sci. USA 109, 17454 (2012). 23064634 10.1073/pnas.1213530109 PMC3491463

[14] Senecal A., Munsky B., Proux F., Ly N., Braye F., Zimmer C., Mueller F., and Darzacq X., Transcription Factors Modulate c-Fos Transcriptional Bursts, Cell Reports 8, 75 (2014). 24981864 10.1016/j.celrep.2014.05.053 PMC5555219

[15] Bartman C. R., Hsu S. C., Hsiung C. C.-S., Raj A., and Blobel G. A., Enhancer Regulation of Transcriptional Bursting Parameters Revealed by Forced Chromatin Looping., Molecular Cell 62, 237 (2016). 27067601 10.1016/j.molcel.2016.03.007 PMC4842148

[16] Fukaya T., Lim B., and Levine M., Enhancer control of transcriptional bursting., Cell 166, 358 (2016). 27293191 10.1016/j.cell.2016.05.025 PMC4970759

[17] Li C., Cesbron F., Oehler M., Brunner M., and Höfer T., Frequency Modulation of Transcriptional Bursting Enables Sensitive and Rapid Gene Regulation, Cell Systems 6, 409 (2018). 29454937 10.1016/j.cels.2018.01.012

[18] Donovan B. T., Huynh A., Ball D. A., Patel H. P., Poirier M. G., Larson D. R., Ferguson M. L., and Lenstra T. L., Live-cell imaging reveals the interplay between transcription factors, nucleosomes, and bursting., The EMBO Journal 38, 10.15252/embj.2018100809 (2019). PMC6576174 31101674

[19] Stavreva D. A., Garcia D. A., Fettweis G., Gudla P. R., Zaki G. F., Soni V., McGowan A., Williams G., Huynh A., Palangat M., Schiltz R. L., Johnson T. A., Presman D. M., Ferguson M. L., Pegoraro G., Upadhyaya A., and Hager G. L., Transcriptional Bursting and Co-bursting Regulation by Steroid Hormone Release Pattern and Transcription Factor Mobility, Molecular Cell 75, 1161 (2019). 31421980 10.1016/j.molcel.2019.06.042 PMC6754282

[20] Pimmett V. L., Dejean M., Fernandez C., Trullo A., Bertrand E., Radulescu O., and Lagha M., Quantitative imaging of transcription in living Drosophila embryos reveals the impact of core promoter motifs on promoter state dynamics, Nature Communications 12, 4504 (2021). 10.1038/s41467-021-24461-6 PMC8302612 34301936

[21] Brouwer I., Kerklingh E., van Leeuwen F., and Lenstra T. L., Dynamic epistasis analysis reveals how chromatin remodeling regulates transcriptional bursting, bioRxiv, 2021.12.15.472793 (2021). 10.1038/s41594-023-00981-1 PMC10191856 37127821

[22] Bass V. L., Wong V. C., Bullock M. E., Gaudet S., and Miller-Jensen K., TNF stimulation primarily modulates transcriptional burst size of NF-*κ*B-regulated genes, Molecular Systems Biology 17, e10127 (2021). 34288498 10.15252/msb.202010127 PMC8290835

[23] Neuert G., Munsky B., Tan R. Z., Teytelman L., Khammash M., and A. v. Oudenaarden, Systematic Identification of Signal-Activated Stochastic Gene Regulation, Science 339, 584 (2013). 23372015 10.1126/science.1231456 PMC3751578

[24] Xu H., Sepúlveda L. A., Figard L., Sokac A. M., and Golding I., Combining protein and mRNA quantification to decipher transcriptional regulation, Nature Methods 12, 739 (2015). 26098021 10.1038/nmeth.3446 PMC4521975

[25] Zoller B., Little S. C., and Gregor T., Diverse Spatial Expression Patterns Emerge from Unified Kinetics of Transcriptional Bursting, Cell 175, 835 (2018). 30340044 10.1016/j.cell.2018.09.056 PMC6779125

[26] Shah S., Takei Y., Zhou W., Lubeck E., Yun J., Eng C.-H. L., Koulena N., Cronin C., Karp C., Liaw E. J., Amin M., and Cai L., Dynamics and Spatial Genomics of the Nascent Transcriptome by Intron seqFISH, Cell 174, 363 (2018). 29887381 10.1016/j.cell.2018.05.035 PMC6046268

[27] Tantale K., Garcia-Oliver E., Robert M.-C., L’Hostis A., Yang Y., Tsanov N., Topno R., Gostan T., Kozulic-Pirher A., Basu-Shrivastava M., Mukherjee K., Slaninova V., Andrau J.-C., Mueller F., Basyuk E., Radulescu O., and Bertrand E., Stochastic pausing at latent HIV-1 promoters generates transcriptional bursting, Nature Communications 12, 4503 (2021). 10.1038/s41467-021-24462-5 PMC8302722 34301927

[28] Hoppe C., Bowles J. R., Minchington T. G., Sutcliffe C., Upadhyai P., Rattray M., and Ashe H. L., Modulation of the Promoter Activation Rate Dictates the Transcriptional Response to Graded BMP Signaling Levels in the Drosophila Embryo, Developmental Cell 54, 727 (2020). 32758422 10.1016/j.devcel.2020.07.007 PMC7527239

[29] Wan Y., Anastasakis D. G., Rodriguez J., Palangat M., Gudla P., Zaki G., Tandon M., Pegoraro G., Chow C. C., Hafner M., and Larson D. R., Dynamic imaging of nascent rna reveals general principles of transcription dynamics and stochastic splice site selection., Cell 184, 2878 (2021). 33979654 10.1016/j.cell.2021.04.012 PMC8183334

[30] Darzacq X., Shav-Tal Y., Turris V. d., Brody Y., Shenoy S. M., Phair R. D., and Singer R. H., In vivo dynamics of RNA polymerase II transcription, Nature Structural & Molecular Biology 14, 796 (2007–09). 10.1038/nsmb1280 PMC4942130 17676063

[31] Garcia H. G., Tikhonov M., Lin A., and Gregor T., Quantitative imaging of transcription in living Drosophila embryos links polymerase activity to patterning., Current Biology 23, 2140 (2013). 24139738 10.1016/j.cub.2013.08.054 PMC3828032

[32] Li J., Dong A., Saydaminova K., Chang H., Wang G., Ochiai H., Yamamoto T., and Pertsinidis A., Single-Molecule Nanoscopy Elucidates RNA Polymerase II Transcription at Single Genes in Live Cells, Cell 178, 491 (2019). 31155237 10.1016/j.cell.2019.05.029 PMC6675578

[33] Gregor T., Garcia H. G., and Little S. C., The embryo as a laboratory: quantifying transcription in Drosophila., Trends in Genetics 30, 364 (2014). 25005921 10.1016/j.tig.2014.06.002 PMC4129518

[34] Little S., Tikhonov M., and Gregor T., Precise Developmental Gene Expression Arises from Globally Stochastic Transcriptional Activity, Cell 154, 789 (2013). 23953111 10.1016/j.cell.2013.07.025 PMC3778922

[35] Lucas T., Ferraro T., Roelens B., Chanes J. D. L. H., Walczak A. M., Coppey M., and Dostatni N., Live imaging of bicoid-dependent transcription in drosophila embryos., Current Biology 23, 2135 (2013). 24139736 10.1016/j.cub.2013.08.053

[36] Levo M., Raimundo J., Bing X. Y., Sisco Z., Batut P. J., Ryabichko S., Gregor T., and Levine M. S., Transcriptional coupling of distant regulatory genes in living embryos., Nature 605, 754 (2022). 35508662 10.1038/s41586-022-04680-7 PMC9886134

[37] Bertrand E., Chartrand P., Schaefer M., Shenoy S. M., Singer R. H., and Long R. M., Localization of ASH1 mRNA particles in living yeast., Molecular Cell 2, 437 (1998). 9809065 10.1016/s1097-2765(00)80143-4

[38] Larson D. R., Zenklusen D., Wu B., Chao J. A., and Singer R. H., Real-time observation of transcription initiation and elongation on an endogenous yeast gene., Science 332, 475 (2011). 21512033 10.1126/science.1202142 PMC3152976

[39] Liu J., Hansen D., Eck E., Kim Y. J., Turner M., Alamos S., and Garcia H. G., Real-time single-cell characterization of the eukaryotic transcription cycle reveals correlations between RNA initiation, elongation, and cleavage, PLoS Computational Biology 17, e1008999 (2021). 34003867 10.1371/journal.pcbi.1008999 PMC8162642

[40] Dubuis J. O., Samanta R., and Gregor T., Accurate measurements of dynamics and reproducibility in small genetic networks., Molecular Systems Biology 9, 639 (2013). 23340845 10.1038/msb.2012.72 PMC3564256

[41] McKnight S. L. and Miller O. L., Post-replicative nonribosomal transcription units in D. Melanogaster embryos., Cell 17, 551 (1979). 113103 10.1016/0092-8674(79)90263-0

[42] Perry M. W., Boettiger A. N., and Levine M., Multiple enhancers ensure precision of gap gene-expression patterns in the Drosophila embryo., Proc. Natl. Acad. Sci. USA 108, 13570 (2011). 21825127 10.1073/pnas.1109873108 PMC3158186

[43] Bothma J. P., Garcia H. G., Ng S., Perry M. W., Gregor T., and Levine M., Enhancer additivity and non-additivity are determined by enhancer strength in the Drosophila embryo., eLife 4, 10.7554/eLife.07956 (2015). PMC4532966 26267217

[44] Fukaya T., Dynamic regulation of anterior-posterior patterning genes in living Drosophila embryos., Current Biology 31, 2227 (2021). 33761316 10.1016/j.cub.2021.02.050

[45] Perry M. W., Bothma J. P., Luu R. D., and Levine M., Precision of hunchback expression in the Drosophila embryo., Current Biology 22, 2247 (2012). 23122844 10.1016/j.cub.2012.09.051 PMC4257490

[46] Farkas G., Leibovitch B. A., and Elgin S. C., Chromatin organization and transcriptional control of gene expression in Drosophila, Gene 253, 117 (2000). 10940549 10.1016/s0378-1119(00)00240-7

[47] Lagha M., Bothma J. P., and Levine M., Mechanisms of transcriptional precision in animal development, Trends in Genetics 28, 409 (2012). 22513408 10.1016/j.tig.2012.03.006 PMC4257495

[48] Park J., Estrada J., Johnson G., Vincent B. J., Ricci-Tam C., Bragdon M. D., Shulgina Y., Cha A., Wunderlich Z., Gunawardena J., and DePace A. H., Dissecting the sharp response of a canonical developmental enhancer reveals multiple sources of cooperativity, eLife 8, e41266 (2019). 31223115 10.7554/eLife.41266 PMC6588347

[49] Liu J., Hansen D., Eck E., Kim Y. J., Turner M., Alamos S., and Garcia H. G., Real-time single-cell characterization of the eukaryotic transcription cycle reveals correlations between RNA initiation, elongation, and cleavage, PLoS Computational Biology 17, e1008999 (2021). 34003867 10.1371/journal.pcbi.1008999 PMC8162642

[50] Sanchez A. and Golding I., Genetic Determinants and Cellular Constraints in Noisy Gene Expression, Science 342, 1188 (2013). 24311680 10.1126/science.1242975 PMC4045091

[51] Larsson A. J. M., Johnsson P., Hagemann-Jensen M., Hartmanis L., Faridani O. R., Reinius B., Segerstolpe r., Rivera C. M., Ren B., and Sandberg R., Genomic encoding of transcriptional burst kinetics, Nature 565, 251 (2018). 10.1038/s41586-018-0836-1 PMC7610481 30602787

[52] Rayon T., Stamataki D., Perez-Carrasco R., GarciaPerez L., Barrington C., Melchionda M., Exelby K., Lazaro J., Tybulewicz V. L. J., Fisher E. M. C., and Briscoe J., Species-specific pace of development is associated with differences in protein stability, Science 369, 10.1126/science.aba7667 (2020). PMC7116327 32943498

[53] Diaz-Cuadros M., Miettinen T. P., Skinner O. S., Sheedy D., Díaz-García C. M., Gapon S., Hubaud A., Yellen G., Manalis S. R., Oldham W. M., and Pourquié O., Metabolic regulation of species-specific developmental rates, Nature 613, 550 (2023). 36599986 10.1038/s41586-022-05574-4 PMC9944513

[54] Tsai A., Muthusamy A. K., Alves M. R., Lavis L. D., Singer R. H., Stern D. L., and Crocker J., Nuclear microenvironments modulate transcription from low-affinity enhancers, eLife 6, e28975 (2017). 29095143 10.7554/eLife.28975 PMC5695909

[55] Henninger J. E., Oksuz O., Shrinivas K., Sagi I., LeRoy G., Zheng M. M., Andrews J. O., Zamudio A. V., Lazaris C., Hannett N. M., Lee T. I., Sharp P. A., Cissé I. I., Chakraborty A. K., and Young R. A., RNA-Mediated Feedback Control of Transcriptional Condensates, Cell 184, 207 (2021). 33333019 10.1016/j.cell.2020.11.030 PMC8128340

[56] Brückner D. B., Chen H., Barinov L., Zoller B., and Gregor T., Stochastic motion and transcriptional dynamics of pairs of distal dna loci on a compacted chromosome, bioRxiv, 2023.01.18.524527 (2023). 10.1126/science.adf5568 PMC10439308 37384691

[57] Cho W.-K., Spille J.-H., Hecht M., Lee C., Li C., Grube V., and Cisse I. I., Mediator and RNA polymerase II clusters associate in transcription-dependent condensates., Science 361, 412 (2018). 29930094 10.1126/science.aar4199 PMC6543815

[58] Nguyen V. Q., Ranjan A., Liu S., Tang X., Ling Y. H., Wisniewski J., Mizuguchi G., Li K. Y., Jou V., Zheng Q., Lavis L. D., Lionnet T., and Wu C., Spatiotemporal coordination of transcription preinitiation complex assembly in live cells, Molecular Cell 81, 3560 (2021). 34375585 10.1016/j.molcel.2021.07.022 PMC8420877

[59] Tkačik G., Callan C. G., and Bialek W., Information flow and optimization in transcriptional regulation, Proc. Natl. Acad. Sci. USA 105, 12265 (2008), 0705.0313. 10.1073/pnas.0806077105 PMC2527900 18719112

[60] Jones D. L., Brewster R. C., and Phillips R., Promoter architecture dictates cell-to-cell variability in gene expression, Science 346, 1533 (2014). 25525251 10.1126/science.1255301 PMC4388425

[61] Hausser J., Mayo A., Keren L., and Alon U., Central dogma rates and the trade-off between precision and economy in gene expression, Nature Communications 10, 68 (2018). 10.1038/s41467-018-07391-8 PMC6325141 30622246

[62] Petkova M. D., Tkačik G., Bialek W., Wieschaus E. F., and Gregor T., Optimal Decoding of Cellular Identities in a Genetic Network, Cell 176, 844 (2019). 30712870 10.1016/j.cell.2019.01.007 PMC6526179

[63] Balakrishnan R., Mori M., Segota I., Zhang Z., Aebersold R., Ludwig C., and Hwa T., Principles of gene regulation quantitatively connect DNA to RNA and proteins in bacteria, Science 378, eabk2066 (2022). 36480614 10.1126/science.abk2066 PMC9804519

[64] Becker K., Balsa-Canto E., Cicin-Sain D., Hoermann A., Janssens H., Banga J. R., and Jaeger J., Reverse-Engineering Post-Transcriptional Regulation of Gap Genes in Drosophila melanogaster, PLoS Computational Biology 9, e1003281 (2013). 24204230 10.1371/journal.pcbi.1003281 PMC3814631

