# Gene Activity as the Predictive Indicator for Transcriptional Bursting Dynamics

**Published:** 2023-10-02

**Authors:** Po-Ta Chen, Benjamin Zoller, Michal Levo, Thomas Gregor

**Affiliations:** 1 Joseph Henry Laboratories of Physics & Lewis-Sigler Institute for Integrative Genomics, Princeton University, Princeton, NJ 08544, USA; 2 Department of Stem Cell and Developmental Biology, CNRS UMR3738 Paris Cité, Institut Pasteur, 25 rue du Docteur Roux, 75015 Paris, France

## Abstract

Transcription commonly occurs in bursts, with alternating productive (ON) and quiescent (OFF) periods, governing mRNA production rates. Yet, how transcription is regulated through bursting dynamics remains unresolved. In this study, we conduct real-time measurements of endogenous transcriptional bursting with single-mRNA sensitivity. Leveraging the diverse transcriptional activities in early fly embryos, we uncover stringent relationships between bursting parameters. Specifically, we find that the durations of ON and OFF periods are linked. Regardless of the developmental stage or body-axis position, gene activity levels predict the average ON and OFF periods of individual alleles. Lowly transcribing alleles predominantly modulate OFF durations (burst frequency), while highly transcribing alleles primarily tune ON durations (burst size). Importantly, these relationships persist even under perturbation of cis-regulatory elements or trans-factors. This suggests a novel mechanistic constraint governing bursting dynamics rather than a modular control of distinct parameters by distinct regulatory processes. Our study provides a foundation for future investigations into the molecular mechanisms underpinning spatiotemporal transcriptional control.

Eukaryotic transcriptional regulation is an inherently dynamic and stochastic process, orchestrated by a series of molecular events governing productive transcription initiation by individual RNA polymerases (Pol II complexes) [1, 2]. This process culminates in nascent RNA synthesis, which in turn shapes protein production, and thus dictates cellular identity and behavior in both space and time. Consequently, revealing the fundamental principles underpinning transcriptional dynamics is paramount for understanding and predicting cellular phenotypes.

Research across diverse biological systems, from yeast to mammalian cells, has revealed that transcription occurs in bursts. These bursts entail the release of multiple Pol II complexes during an active phase, often referred to as the “ON” period, followed by a quiescent “OFF” period [3–9]. However, critical questions remain unanswered: how does the regulation of bursting kinetics shape mRNA production and transcriptional dynamics across developmental time and cell types? Is the transcription rate primarily regulated by adjusting the durations of the ON or OFF periods, the initiation rate (i.e., the rate of Pol II release during active phases), or a combination of these parameters? Furthermore, do different genes employ distinct bursting strategies? Do these strategies vary in temporal and spatial (tissue-specific) transcriptional control, and how do they depend on the regulatory factors at play?

One hypothesis that has emerged from previous work suggests that different regulatory factors, including transcription factor (TF) binding, *cis*-regulatory elements, nucleosome occupancy, histone modification, Pol II pausing, and enhancer–promoter interactions, may influence distinct aspects of bursting dynamics [10–19]. For instance, it has been proposed that enhancers primarily impact burst frequency, while promoters primarily affect burst size [20–22]. However, integrating diverse observations into a unified and quantitatively predictive understanding of transcriptional control through bursting dynamics has proven to be challenging.

In our previous study [23], relying on inference from a static snapshot of mRNA abundance, we explored the intriguing possibility that a simple and unified control of bursting kinetics might be at play, even in complex developmental systems. Specifically, we demonstrated that a straightforward two-state kinetic model of transcription, with a single free parameter, could account for transcript abundance of key developmental genes in two-hour-old *Drosophila* embryos. This finding motivated our efforts to perform highly-sensitive live transcription measurements, enabling us to directly measure bursting dynamics without relying on model-specific kinetic assumptions.

Here, we present real-time endogenous transcription measurements with single mRNA sensitivity in developing fly embryos, where tightly regulated spatiotemporal transcriptional dynamics play a pivotal role in determining cell fate. Transcription of the examined genes is monitored across developmental time in addition to body-axis positions, as well as and under perturbation to *cis*-and *trans*-regulatory factors. Surprisingly, all data collapse onto strict relationships between bursting parameters. We find that transcriptional activity is primarily determined by the probability of an allele being in the ON period, while the initiation rate remains largely constant. Moreover, we identify a tight relationship between the durations of ON and OFF periods, implying that these periods are not tuned independently. This discovery hints at the existence of a novel mechanistic constraint on the transcription initiation process.

A corollary of the observed relationship is that lowly transcribing alleles increase activity by predominantly shortening OFF periods, increasing burst frequency, while medium-to-high transcribing alleles predominantly extend ON periods, thereby increasing burst size. Importantly, perturbations to transcription prompt a re-examination of our mechanistic understanding, as these highlight that activity levels, rather than specific regulatory determinants (such as enhancer sequences or transcription factor concentrations), can predict bursting parameters. Overall, our study reveals constraints on the molecular implementation of transcriptional dynamics, thereby guiding future mechanistic investigations.

## RESULTS

### Instantaneous single allele transcription rate measurements.

**FIG. 1.**
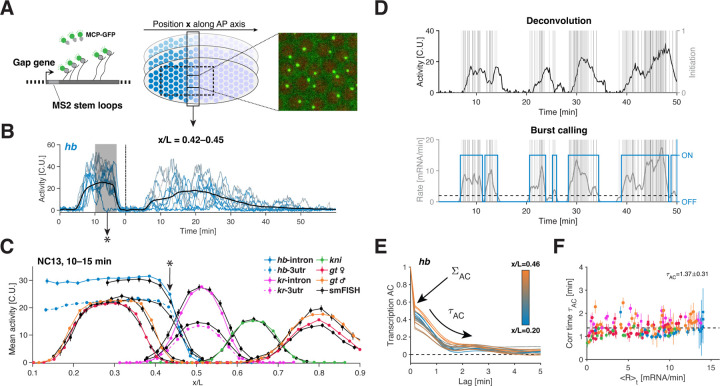
Live single-cell transcription rate measurements of endogenous gap genes. (A) Live fluorescence imaging of nascent transcripts using MS2 stem-loops measures single allele transcriptional activity (green hotspots) along the anterior-posterior (AP) axis of the fly embryo (also see Fig. S1A and methods). (B) Transcription time series for 10 alleles (blue) of the gap gene *hunchback* (*hb*) at position *x*/*L* = 0.435 ± 0.010 sampled every 10 s. Low embryo-to-embryo variability (Fig. S1F) enables pooling alleles from multiple spatially and temporally aligned embryos (*n* =10–20) to average over 200–350 alleles at a given position (black). (C) Calibration of transcriptional activity in absolute units performed by matching mean spatial activity profiles from previously calibrated fixed smFISH measurements [23] (black) with 5-min-interval averages (gray shade in B) of live time series at each AP position (color) for all examined gap genes. A single global conversion factor matches live and fixed profiles to within 5% error (Fig. S1B), defining a unit for transcriptional activity (i.e., the cytoplasmic unit, C.U. [24]) equivalent to a fully tagged transcript. (D) Reconstruction of transcription initiation events (gray bars) and underlying bursts from single allele transcription time series (black). (Top) Bayesian deconvolution enables sampling possible configuration of initiation events (see Methods and Fig. S2A–B). (Bottom) Clustering of sampled initiation events (using a moving average of width ~ 1 min (gray curve) and a threshold at two mRNA/min (dashed line)) identifies individual bursts (blue). (E) Auto-correlation (AC) functions of single allele *hb* transcription rates averaged over time for different positions along the AP axis (color). AC functions are normalized by the variance; uncorrelated (Σ_AC_) and time-correlated (*τ*_AC_) components of the rate fluctuations are highlighted (see Fig. S5). (F) Correlation time *τ*_AC_ (from fitted exponentials, see Methods) of the single allele transcription rate as a function of mean transcription rate *R* (color code as in C). Dashed line corresponds to overall mean correlation time ${\bar{\tau }}_{\text{AC}}=1.37\pm 0.31\;\min.$ Error bars are 68% confidence intervals.

We developed a quantitative approach to measure endogenous bursting dynamics at a single allele level in living *Drosophila* embryos. To achieve this we utilized a versatile CRISPR-based scheme [25] to incorporate MS2 cassettes into intronic or 3’ untranslated regions (3’UTRs) of the gap genes. These cassettes form stem-loops in the transcribed nascent RNA, which are subsequently bound by fluorescent coat-proteins (Fig. 1A, S1A, and Methods) [26–29]. We employed a custom-built two-photon microscope to generate fluorescence images, allowing us to capture RNA synthesis from one tagged allele per nucleus with nearly single-mRNA sensitivity (Fig. S1D–E). Our optimized field-of-view provided 10-second interval time-lapses (Fig. 1B) for hundreds of nuclei per embryo during a critical 1.5-hour period of embryonic development, specifically nuclear cycles 13 (NC13) and 14 (NC14), essential for robust statistical analysis (Fig. 1A–B; Videos V1–V4).

We calibrated our fluorescence signal using smFISH data to express our dynamic transcription measurements in terms of absolute mRNA counts (Fig. 1C and S1B–C, and details in Methods). This calibration, combined with the nearly single-transcript sensitivity of our measurements enabled us to reconstruct the underlying Pol II transcription initiation events for each allele using Bayesian deconvolution (see Methods). The convolution kernel we employed describes the fluorescent signal resulting from the release of Pol II complexes onto the gene, which subsequently engage in the elongation process [17, 21] (assuming constant and deterministic elongation, Fig. S2A). For each time trace, our Bayesian approach generates multiple configurations of transcription initiation events (Fig. 1D). By averaging these configurations, we obtained a time-dependent instantaneous single allele transcription rate, denoted as *r*(*t*) (Fig. S2B). Importantly this approach also provides corresponding error estimates, which we propagated in all subsequent analyses.

Our kernel-based deconvolution approach was validated by control measurements involving dual-color tagging of the gene body, both at 5’ and 3’ regions (Fig. S2C). These measurements support our key assumptions regarding the elongation process and the absence of co-transcriptional splicing (see Methods). Furthermore, they allow us to extract a Pol II elongation rate, denoted as *K*_elo_, which we determined to be 1.8±0.1 kb/min. This value aligns with previous measurements reported in the literature [28, 30] (Fig. S2D–G).

With our approach, the extracted single allele transcription rates are no longer masked by the Pol II elongation dwell time, unlike the directly measured intensities. Instead, they capture initiation events (i.e., Pol II release for productive elongation). Consequently, these rates are independent of gene length, allowing for direct comparisons across different genes. This facilitated the intriguing observation that the genes reach a similar maximum average transcription rate, denoted as *R* = 〈*r*〉 (Fig. S3A, Video V5). Moreover, these average transcription rates closely mirror the well-documented average protein dynamics [31]. Simple assumptions related to diffusion and lifetime, without the need for explicit post-transcriptional regulation, are sufficient to quantitatively predict protein patterns from the mean transcription rates *R* (Fig. S4 and Video V6). Thus, in this system, the functional output, namely protein synthesis, predominantly relies on transcription regulation. Our quantitative imaging and deconvolution approach pave the way for uncovering how this regulation emerges from the single-allele transcription dynamics.

### Single allele transcription rates hint at a universal bursting regime.

The gap genes differ in their transcriptional activities both spatially and temporally. However, when we examine the distributions *P*(*r*|*R*) of single-allele transcription rates *r* that yield a similar mean transcription rate *R*, an intriguing pattern emerges. These distributions converge consistently across different genes (Fig. S3C, see Methods), suggesting the existence of a common transcriptional regime. For transcription rates in the low- to mid range of *R*, we observe an abundance of non-transcribing or barely transcribing alleles. These distributions starkly contrast with a regime characterized by constitutive transcription. Conversely, for transcription rates in the high range of *R*, the distributions converge towards the constitutive, or Poissonian, regime (Fig. S3D), indicating a higher proportion of active ON alleles. These observations are consistent with the concept of transcriptional bursting, where an allele dynamically transitions between productive ON and quiescent OFF periods [3, 32].

We obtain additional support for a common bursting regime when we analyze the temporal dynamics of single-allele time traces. Bursting is expected to introduce temporal correlations in transcriptional activity, reflecting the persistence of the ON and OFF periods (Fig. S5A–B). To characterize such correlations, we compute auto-correlation functions for the deconvolved single-allele transcription rates. By using the deconvolved rates we effectively remove the correlated component arising from Pol II elongation along the gene, and isolates only the correlations stemming from the initiation and the ON–OFF switching process. When we calculate these auto-correlation functions for different anterior-posterior (AP) bins, nuclear cycles, and various genes (Fig. 1E), we find striking similarities. An initial sharp drop at our sampling time scale (~ 10 s) indicates the presence of uncorrelated noise, consistent with independent Pol II initiation events (Fig. S5C). This drop is followed by a longer decay of correlated noise at a time scale denoted as *τ*_AC_, which we find to be confined within 1- to 2-minute range (Fig. 1F).

The remarkable consistency of *τ*_AC_ across different spatial locations, genes, and transcriptional activity levels (spanning *R*, Fig. 1F) implies the preservation of this fundamental time scale of transcription dynamics. To delve deeper into potential regularities in bursting dynamics, our next step involves directly extracting individual bursts from single-allele time traces.

### Allele ON-probability is the primary transcription control parameter.

From the deconvolved initiation events along individual time traces (Fig. 1D top) we identify distinct ON and OFF periods of active and inactive transcription, respectively. The ON periods are characterized by consecutive initiation events (i.e., multiple Pol IIs released for productive elongation), while the OFF periods are transcriptionally inactive (Fig. 1D bottom). To delineate the transition of an allele from an OFF to an ON state, we employ a a simple threshold on the moving average of the single allele transcription rate, set to 2 mRNA/min over a 1-min-window. This criterion is selected based on our detection sensitivity, allowing us to reliably detect of 1 – 2 mRNA molecules, and the window is consistent with the time scale derived from the auto-correlation analysis (see Methods).

We conducted extensive testing to evaluate the impact of these detection parameters on our analysis, and our results confirm that our intuitive choice minimizes errors in burst characterization (see below, Fig. S9E). Importantly, the primary strength of this burst-calling routine lies in its exclusive reliance on a minimal clustering model. Consequently it is inherently devoid of assumptions about the distributions of ON and OFF periods. As a result, our burst detection process remains agnostic to the underlying transcription models, as long as transcription can be described by at least one ON and OFF state (which is the case for common N-sate models [9, 12, 17, 21, 33–35].

The next goal of our analysis is to elucidate how the consecutive switches between ON and OFF periods quantitatively govern the transcription rate *R*. Specifically, the mean transcription rate at time *t*, denoted as *R*(*t*), can be decomposed into two distinct parameters: the instantaneous probability of an allele being in the ON state (*P*_ON_(*t*)), representing the fraction of ON alleles at time *t*, and the mean initiation rate (*K*(*t*)) for ON alleles. Given the above decomposition, most of the variation in *R* could arise from changes in either *K*, *P*_ON_, or both.

**FIG. 2.**
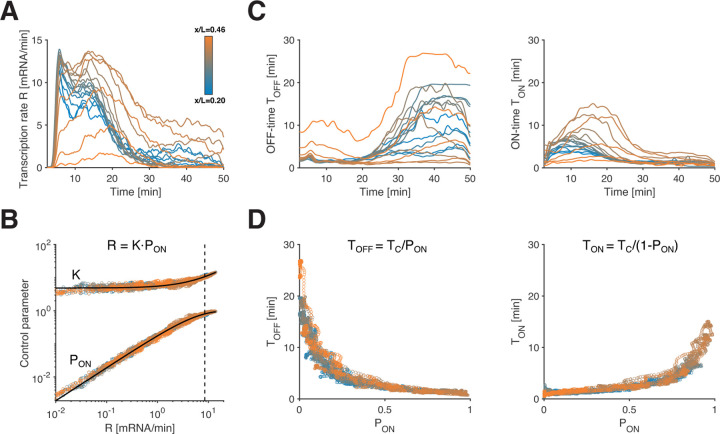
Direct estimation of instantaneous mean transcription parameters. (A) *Hb* mean transcription rate *R* as a function of time in NC14 (color encodes AP position). (B) Control parameters *P*_ON_ and *K* as a function of mean transcription rate *R* in log-space. Since log (*R*) = log (*K*) + log (*P*_ON_) by construction, changes in *P*_ON_ determine changes in *R* below the dashed line (*R* ~ 8.5 mRNA/min, corresponding to *P*_ON_ = 0.75). (C) *hb* mean OFF-time *T*_OFF_ and mean ON-time *T*_ON_ as a function of time in NC14 for all AP positions (color code as in (A)). (D) Mean OFF-time *T*_OFF_ and mean ON-time *T*_ON_ as a function of *P*_ON_, for all positions and time points beyond the 7.5 min mark in (C).

Starting with the gene *hb*, we estimate the time-dependent parameters *R*(*t*) and *P*_ON_(*t*) for each AP bin. To obtain *R*(*t*), we calculate the average of ~250 single-allele instantaneous transcription rates (Fig. S6A). Concurrently, we determine *P*_ON_(*t*) by quantifying the fraction of alleles in the ON state at each time point (Fig. S6B). To compute *K*(*t*), we average initiation events restricted to the ON state. By repeating this procedure for all AP positions, we reveal the spatiotemporal variations in *P*_ON_ and *K* (Fig. 2A, Fig. S6C–D). We validate our approach for burst calling and the recovery of bursting parameters from transcription time traces across a wide range of simulated data. We achieve an overall median error of 10%, gaining insights into the robustness of our analysis across the potential parameter space (see Methods, Fig. S9 and Fig. S10).

We find that all three parameters vary significantly across space and time (Fig. 2A and S6C–D). While we observe the expected interdependence *R* = *K* · *P*_ON_ (Fig. S6F), our analysis indicates that changes in *R* are primarily governed by changes in *P*_ON_, while the influence of *K* is more moderate and less predictive of *R* (Fig. 2B, Fig. S7). These results for *hb* suggest that transcriptional activity is predominantly controlled by the probability of an allele being in the ON state, and once in the ON state, transcription initiates at a quasi-constant rate.

Any given ON-probability can result from various combinations of ON and OFF periods. This raises the possibility that alleles at different spatial positions and times employ distinct combinations, or alternatively, there could be underlying regularities governing these periods. We computed the mean ON and OFF times denoted as *T*_ON_ and *T*_OFF_ (Fig. S8A–C; see Methods), and found that these also vary substantially across space and time (Fig. 2C). However, when we plot *T*_ON_ and *T*_OFF_ against *P*_ON_, we find that all data points collapse onto two tight anti-symmetric relationships (Fig. 2D). Despite the potential for multiple combinations of *T*_ON_ and *T*_OFF_ for any given *P*_ON_, these relationships consistently associate a given *P*_ON_ value with a unique pair of *T*_ON_ and *T*_OFF_ values, irrespective of spatial position or time.

The dynamic switching between ON and OFF states is associated with a correlation time *T*_C_, which determines the time separation required for the transcription rate of a single allele to become independent. *T*_C_ can be computed directly from the mean ON and OFF times using the equation 1/*T*_C_ = 1/*T*_ON_ + 1/*T*_OFF_ (Fig. S8E). For the *hb* gene, *T*_C_ is confined between 1 – 1.5 min across all positions and time points, and seems independent of *P*_ON_. The constancy of *T*_C_ effectively links the mean ON and OFF times. Additionally, since *T*_ON_ and *T*_OFF_ can be expressed as functions of *P*_ON_ and *T*_C_ (Fig. 2D, Fig. S8B), the constancy of *T*_C_ provides a mathematical explanation for the tight anti-symmetric relationships between *T*_ON_, *T*_OFF_, and *P*_ON_ (Fig. 2D). Thus, not only does *P*_ON_ govern the mean transcription rate *R*, but also the entire transcriptional bursting dynamics.

### Common bursting relationships underlie the regulation of all gap genes.

The gap genes differ in the composition, number, and arrangements of their *cis*-regulatory elements (Fig. S1A), resulting in distinct regulatory binding events (e.g., by transcription factors, transcription machinery components, and chromatin modifiers) [36]. Consequently, each gene displays unique spatiotemporal transcriptional activities (Fig. 1C, Video V5). Despite these differences, we find that the relationships governing bursting parameters for *hb* appear to generalize to other gap genes.

**FIG. 3.**
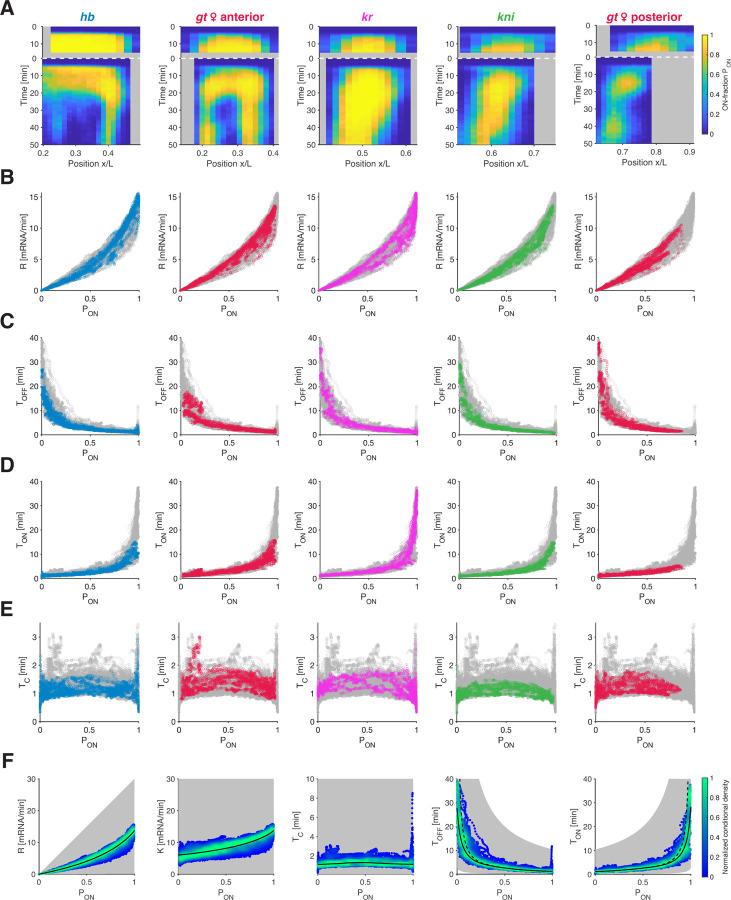
Transcription parameters collapse for all gap genes. (A) Kymographs of ON-probability *P*_ON_ for all gap genes as a function of position and time for NC13 and NC14. The spatiotemporal transcriptional pattern of the gap genes arises from a complex regulation of *P*_ON_ (color map). (B-E) Transcription parameters collapse for all gap genes across time and position. Transcription rate *R* (B), Mean OFF-time *T*_OFF_ (C), ON-time *T*_ON_ (D), and switching correlation time *T*_C_ (E) as a function of *P*_ON_. Colored data points represent individual gap genes (same color code as in (A), see Fig. S11A–B for *gt* male data). Each panel shows all the remaining genes in gray. (F) Density (color) of all data points across space and time of the transcription parameters for all gap genes, normalized by the maximum density. Potentially accessible space (gray shade) for plausible ranges of *K* (0.1–30 mRNA/min) and *T*_C_ (0.1–10 min). *P*_ON_ almost fully determines *R* and sets the combinations of *T*_OFF_ and *T*_ON_. For *T*_OFF_ and *T*_ON_, the dashed lines are the 2-state model predictions based on *T*_C_, and the solid lines take the finite recording length into account (see Fig. S11D).

When we applied our burst calling procedure (Fig. 1D) to the transcription time traces of other gap genes (*gt*, *Kr*, and *kni*), we obtain distinct spatiotemporal *P*_ON_ profiles (Fig. 3A) that closely mirrored the gene-specific transcription rates *R*. Indeed, all genes exhibit nearly identical relationships between *R* and *P*_ON_ (Fig. 3B) and between *K* and *P*_ON_ (Fig. S11C), affirming that *P*_ON_ is the predominant factor governing transcriptional activity across time, space, and genes.

Furthermore, the genes display similar relationships between *T*_OFF_ and *P*_ON_ (Fig. 3C) and between *T*_ON_ and *P*_ON_ (Fig. 3D). Thus, when different genes exhibit a specific *P*_ON_ value, potentially at different spatiotemporal coordinates, the underlying *T*_ON_ and *T*_OFF_ periods are nonetheless largely identical. This finding can be related to the conservation of the switching correlation time *T*_C_ across all positions, times, and genes (Fig. 3E), with the average *T*_C_ value (1.25±0.37 min) aligning quantitatively with the time scale predicted by the auto-correlation analysis above (Fig. 1F). Notably, the common bursting relationships apply not only across genes but also across distinct spatiotemporal domains of activity of a single gene, known to be driven by distinct enhancers [37, 38]. This is particularly evident in the large and yet distinct anterior and posterior domain of the gene *gt* (Fig 3A and Fig. S11). Use of similar bursting parameters in distinct spatial patterns of a single gene was indeed proposed in a study of a reporter construct of the gene *eve* [39, 40].

Pooling the parameters derived from all genes, times, locations, and embryos (comprising over 10^6^ data points) accentuates the limited subset of the parameter space utilized and underscores the stringent quantitative relationships emerging from our dataset (Fig. 3F, Fig. S13). When we segregate the data into three developmental time windows, these relationships tighten even further, hinting at a modest developmental time dependence (Fig. S12). Overall, our analysis shows that, across all data points, the mean transcription rate *R* is primarily governed by *P*_ON_, While *K* remains largely constant. The near-constant switching correlation time *T*_C_ results in an apparent inverse proportionality between *T*_ON_ and *T*_OFF_, with only one of these two parameters primarily modulated when *P*_ON_ changes.

While lowly-transcribing alleles (as characterized by *P*_ON_) tend to achieve higher expression levels mainly by reducing *T*_OFF_, medium-to-high-transcribing alleles are predominantly tuned by extending *T*_ON_ (Fig. 3F). This observation means that changes in burst frequency (*F* = 1/(*T*_ON_ + *T*_OFF_)) govern tuning of the transcriptional activity of low-transcribing alleles, while changes in burst size (*B* = *K* · *T*_ON_) exert greater influence on the tuning of medium-to-high-transcribing alleles (Fig. S11E). Consequently, despite the inherent differences among these genes, shared functional relationships associate any given ON-probability with specific underlying bursting characteristics.

### Common bursting relationships predict effects of *cis*- and *trans*-perturbations.

Diverse regulatory mechanisms have been implicated in the control of transcriptional activity, including the contributions of *cis*-regulatory elements such as enhancers and *trans*-factors like TF repressors. It is often assumed that distinct regulatory mechanisms exert direct control over specific bursting parameters. Thus, we sought to perturb distinct regulatory mechanisms and to assess whether they yield distinct effects on bursting dynamics, or whether the relationships elucidated from wild-type measurements, could account for the modified transcriptional activity in these perturbed scenarios.

**FIG. 4.**
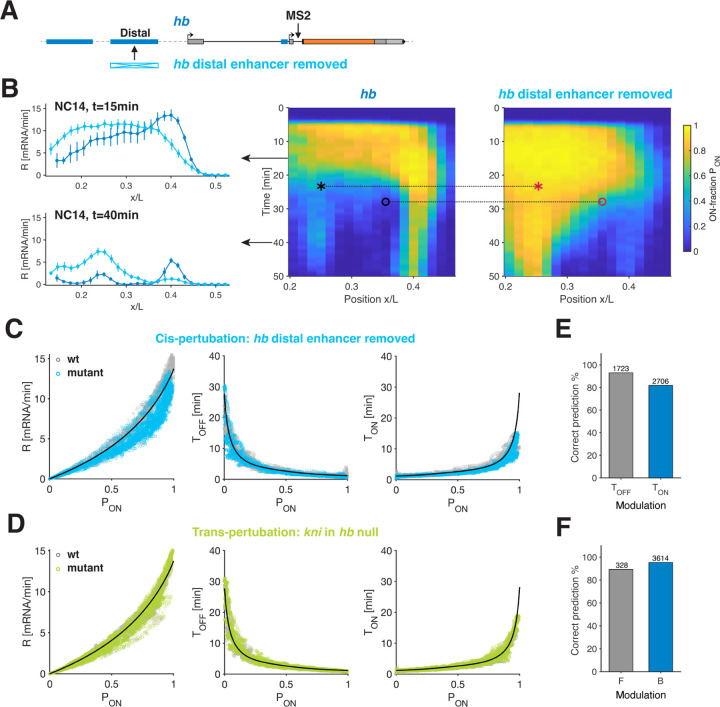
Effect of *cis*- and *trans*-perturbations on ON and OFF times. (A) Distal *hb* enhancer removal from the fly line carrying MS2 stem-loops in the endogenous *hb* locus. (B) Quantification of *hb* wild-type and mutant phenotypes. Both transcription rate *R* (left) and *P*_ON_ (right) levels display significantly different expression patterns for the enhancer deletion mutant. Black arrows indicate the time points (15 and 40 min into NC14) in the kymograph at which rate profiles are depicted. “o” and “∗” mark two bins with predominant *T*_OFF_ modulation and predominant *T*_ON_ modulation, respectively.(C) Transcription parameters for *hb* enhancer deletion (cyan) collapse on corresponding wild-type parameters (gray). (D) Transcription parameters for *kni* (green) in a *hb* null background (i.e. a *trans*-mutation, Fig. S14D–E) collapse on corresponding wild-type parameters (gray). Solid black lines in (C) and (D) correspond to the endogenous bursting relationships from Fig. 3F. (E-F) Verification of predicted changes in *T*_OFF_ and *T*_ON_ (E) (or changes in burst size *B* and frequency *F* (F)) for all wild-type and mutant *P*_ON_ pairs (two example pairs shown in (A) for *hb*). For most pairs (> 85%) the prediction is correct (see also Fig. S15A–C.

Upon endogenous deletion of the distal enhancer of *hb*, we observe significant alterations in transcriptional activity (Fig. 4A–B, Video V7). The perturbation leads to both increased and decreased activity at different times and locations along the AP axis, consistent with previous findings [41]. However, we find that bursting dynamics in this mutant still adhere to the relationships identified in wild-type context. Specifically, transcription rates across different spatial and temporal coordinates are predominantly governed by *P*_ON_, the stringent relationships between *T*_ON_/*T*_OFF_ and *P*_ON_ hold, and the switching correlation time *T*_C_ remains broadly conserved (Fig. 4C and S15A–D).

Two additional perturbations further confirmed these findings. A second enhancer deletion, removing the distal enhancer of *kni* results in a significant reduction in *kni* activity (Fig. S14A–B, Video V8). Although the mutant exhibits a narrower dynamic range of activity, we still observe a similar data collapse within this reduced range (Fig. S14C and S15A–D). Next, we explore the effect of a *trans*-perturbation by measuring *kni* activity in embryos with a *hb* null background (Fig. S14D–E, Video V9). This *trans*-perturbation significantly alters *kni* activity, consistent with earlier studies [42]). However, the underlying bursting dynamics again collapse onto the same busting relationships (Fig. 4D and S15A–D).

The consistency of these relationships across all examined conditions suggests that they can predict how changes in *T*_ON_ and *T*_OFF_ account for the change in transcriptional activity following a perturbation. In essence, since these relationships relate any given *P*_ON_ to specific *T*_ON_ and *T*_OFF_ pairs, a measured change in *P*_ON_ upon a perturbation provides a prediction as to which of the two parameters was primarily altered. Remarkably, for each type of the examined perturbations, we find both predominant *T*_ON_ and *T*_OFF_ modulation at different spatiotemporal coordinates (Fig. 4E). When comparing the wild-type-derived predictions with the directly measured *T*_ON_ and *T*_OFF_ from the mutant time traces, we find an agreement of better than ~86% for all spatiotemporal coordinates (Fig. 4E and S15E,G and I). Similar successful predictions are achieved when assessing the change in transcriptional activity in terms of altered burst size versus burst frequency (Fig. 4F and S15F,H and J). These findings challenges previous intuitions linking perturbations of specific regulatory elements or mechanisms to changes in a particular bursting parameter and instead suggest the predictive power of *P*_ON_ across different perturbations.

**FIG. 5.**
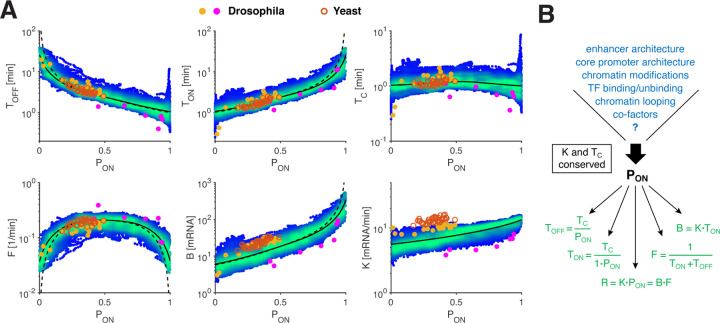
Generality of relationships and decoupling of regulatory mechanisms and transcriptional response. (A) Scatter plots of the transcription parameters versus *P*_ON_ (color code as in Fig. 3F). Transcription parameters computed from two other *Drosophila* studies (yellow circles [16] and pink circles [21], respectively, see text) are largely consistent with transcription relationships (black lines). Transcription parameters resulting from multiple perturbations performed on the yeast *GAL10* gene (orange circles [19]) also closely follow our relationships, suggesting that these may apply beyond *Drosophila*. (B) While *K* and *T*_C_ appear mostly conserved, *P*_ON_ emerges as the key regulatory parameter. It is independent of gene identity (different *cis*-architecture) or the combination of input transcription factor concentrations (that vary along the AP axis), validated by *cis*- and *trans*- perturbations and by data from other studies. Such invariance suggests a mechanistic decoupling, where regulation affects solely *P*_ON_, which in turn determines unequivocally all of the underlying bursting dynamics.

To further explore the generality of these observations, we examined data from two previous studies in the early fly embryo. In one study, transcription was modulated by varying BMP signal, a dorsoventral morphogen [16], while in another, transcription was altered by the use of different core promoter sequences in synthetic reporters [21]. Remarkably, we found that these datasets also collapsed onto our established bursting relationships (Fig. 5A). As suggested in these studies, the first dataset shows predominantly *T*_OFF_ modulation, while the latter has primarily *T*_ON_ changes. Intriguingly, the two independent datasets cluster in disjoint halves of the full spectrum of *P*_ON_ values captured by our measurements. Our analysis raises the possibility that the predominantly changed parameter (*T*_OFF_ versus *T*_ON_) might not be inherent to the examined regulatory manipulation (e.g., input TF concentrations or core promoter elements), but rather a consequence of the expression range (the *P*_ON_ regime) of these genes.

## DISCUSSION

In this study, we developed an approach to quantify real-time single allele transcriptional bursting in the context of the developing early *Drosophila* embryo. The wide range of transcriptional activities that shape protein abundance in this system played a crucial role in our ability to uncover the fundamental relationships governing bursting dynamics. Rather than specific regulatory processes being inherently tied to distinct bursting parameters, our data suggests that most transcriptional tuning is channeled through a single control parameter: the ON-probability *P*_ON_ (Fig. 5B). Moreover, we discovered that active (ON) and inactive (OFF) states are correlated over a conserved time scale (*T*_C_) of approximately a minute. This time constant explicitly links the mean durations of ON and OFF periods, which are thus predominantly determined by the control parameter, *P*_ON_. Therefore, gene activity, as characterized by *P*_ON_ (given a relatively constant initiation rate), conveys extensive information about the underlying bursting parameters.

As our parameter estimation does not rely on fitting a specific mechanistic model of transcription to the data but, instead, directly identifies ON and OFF periods from single-allele initiation events, the uncovered bursting relationships are both effective and broadly applicable across various models. Any detailed N-state model [9, 12, 21, 33] must adhere to these relationships once coarsely adapted to produce ON and OFF periods akin to those observed in the data. Although the identified relationships do not originate from model-specific kinetic assumptions, they impose constraints on the underlying kinetic parameters of any transcription model (Fig. S16). This, in turn, encourages the development of models that inherently yield the observed dependencies.

Notably, the linkage between *T*_ON_ and *T*_OFF_ — or, equivalently, the constancy of *T*_C_ — is not inherent to prevailing models of transcription and isn’t readily mapped to commonly invoked molecular processes. This strongly suggests an as-yet-unappreciated mechanistic constraint. Given the conserved nature of the transcription machinery and regulatory mechanisms across eukaryotes, it is highly probable that such a fundamental constraint, and the identified relationships more broadly, will apply to a wider range of systems [43].

Comparing quantitative parameters between systems is challenging due to measurement and analysis differences across distinct experimental setups. However, we managed to examine bursting parameters derived from extensive perturbations of a yeast gene [19], and we observe strong agreement with our findings from the early fly embryo (Fig. 5A and methods). Specific rates, like the initiation rate *K*, may differ [5, 27] (see Methods), possibly due to species-specific metabolic variations [44, 45]. Nonetheless, it is the extracted relationships, likely reflecting underlying mechanisms, that we anticipate to generalize. Two additional studies in mammalian systems, despite employing vastly different approaches, point to trends consistent with our established bursting relationships [22, 46]. In one study, testing a library of reporter genes derived from human loci, the authors found that while burst frequency is predominantly modulated to increase activity for low expressing reporters, burst size tunes high-expressing ones, independent of control sequences [46]. Another study, which examined endogenous mouse and human genes using single-cell RNA-seq, identified a functional dependence of burst frequency and burst size on mean expression, which seems compatible with our established relationships (for *P*_ON_ < 0.5) [22].

The discovered bursting relationships, and their potential applicability to other species, warrant the development of a novel mechanistic framework for transcriptional regulation. In future studies, it will be imperative to unravel the molecular mechanisms responsible for integrating diverse regulatory processes (e.g., repressor and activator binding, chromatin accessibly, PIC formation and pause-release, histone modifications, etc.) into the control parameter *P*_ON_. Of even greater intrigue is deciphering how the constancy of *T*_C_ is molecularly implemented. Our work suggests a mechanism that operates independently of the specific gene locus and might be conserved across eukaryotic systems. Intriguing possibilities involve the transcriptional environment, including structural aspects of nuclear architecture or the molecular assembly and disassembly of transcription machinery components and their localized accumulation [47–52].

In addition to opening new research directions into the molecular underpinning of the observed relationships and conserved parameters, our study raises questions about the functional consequences and potential benefits of this identified regime. For instance, the central time scale of the bursting dynamics, *T*_C_, could play a key role in transcription noise filtering and efficient regulation. Its measured small value minimizes noise, as bursts are easily buffered by longer mRNA lifetimes. Moreover, the small *T*_C_ allows gene transcription to respond rapidly to input TF changes (Fig. S8C–D). This swift responsiveness can have significant implications for the dynamic regulation of gene expression. Thus, similar to other fields where organizing principles are emerging, such as, e.g., optimizing information flow [53], and many others[54–57], our elucidated relationships offer valuable insights into the functionality encoded by complex processes and guide future investigations into the conserved mechanisms at their core.

## Supplementary Material

1

## References

[1] Lelli K. M., Slattery M., and Mann R. S., Disentangling the many layers of eukaryotic transcriptional regulation., Annual Review of Genetics 46, 43 (2012). 10.1146/annurev-genet-110711-155437 PMC4295906 22934649

[2] Cramer P., Eukaryotic transcription turns 50, Cell 179, 808 (2019). 31675494 10.1016/j.cell.2019.09.018

[3] Raj A., Peskin C. S., Tranchina D., Vargas D. Y., and Tyagi S., Stochastic mRNA synthesis in mammalian cells., PLoS biology 4, e309 (2006). 17048983 10.1371/journal.pbio.0040309 PMC1563489

[4] Chubb J. R., Trcek T., Shenoy S. M., and Singer R. H., Transcriptional Pulsing of a Developmental Gene, Current Biology 16, 1018 (2006). 16713960 10.1016/j.cub.2006.03.092 PMC4764056

[5] Zenklusen D., Larson D. R., and Singer R. H., Single-RNA counting reveals alternative modes of gene expression in yeast., Nature Structural & Molecular Biology 15, 1263 (2008). 10.1038/nsmb.1514 PMC3154325 19011635

[6] Suter D. M., Molina N., Gatfield D., Schneider K., Schibler U., and Naef F., Mammalian genes are transcribed with widely different bursting kinetics., Science 332, 472 (2011). 21415320 10.1126/science.1198817

[7] Bothma J. P., Garcia H. G., Esposito E., Schlissel G., Gregor T., and Levine M., Dynamic regulation of eve stripe 2 expression reveals transcriptional bursts in living drosophila embryos., Proc. Natl. Acad. Sci. USA 111, 10598 (2014). 24994903 10.1073/pnas.1410022111 PMC4115566

[8] Tantale K., Mueller F., Kozulic-Pirher A., Lesne A., Victor J.-M., Robert M.-C., Capozi S., Chouaib R., Bäcker V., Mateos-Langerak J., Darzacq X., Zimmer C., Basyuk E., and Bertrand E., A single-molecule view of transcription reveals convoys of RNA polymerases and multi-scale bursting, Nature Communications 7, 12248 (2016). 10.1038/ncomms12248 PMC4974459 27461529

[9] Wan Y., Anastasakis D. G., Rodriguez J., Palangat M., Gudla P., Zaki G., Tandon M., Pegoraro G., Chow C. C., Hafner M., and Larson D. R., Dynamic imaging of nascent rna reveals general principles of transcription dynamics and stochastic splice site selection., Cell 184, 2878 (2021). 33979654 10.1016/j.cell.2021.04.012 PMC8183334

[10] Senecal A., Munsky B., Proux F., Ly N., Braye F., Zimmer C., Mueller F., and Darzacq X., Transcription Factors Modulate c-Fos Transcriptional Bursts, Cell Reports 8, 75 (2014). 24981864 10.1016/j.celrep.2014.05.053 PMC5555219

[11] Bartman C. R., Hsu S. C., Hsiung C. C.-S., Raj A., and Blobel G. A., Enhancer Regulation of Transcriptional Bursting Parameters Revealed by Forced Chromatin Looping., Molecular Cell 62, 237 (2016). 27067601 10.1016/j.molcel.2016.03.007 PMC4842148

[12] Li C., Cesbron F., Oehler M., Brunner M., and Höfer T., Frequency Modulation of Transcriptional Bursting Enables Sensitive and Rapid Gene Regulation, Cell Systems 6, 409 (2018). 29454937 10.1016/j.cels.2018.01.012

[13] Nicolas D., Zoller B., Suter D. M., and Naef F., Modulation of transcriptional burst frequency by histone acetylation, Proc. Natl. Acad. Sci. USA 115, 201722330 (2018). 10.1073/pnas.1722330115 PMC6142243 29915087

[14] Donovan B. T., Huynh A., Ball D. A., Patel H. P., Poirier M. G., Larson D. R., Ferguson M. L., and Lenstra T. L., Live-cell imaging reveals the interplay between transcription factors, nucleosomes, and bursting., The EMBO Journal 38, 10.15252/embj.2018100809 (2019). PMC6576174 31101674

[15] Falo-Sanjuan J., Lammers N. C., Garcia H. G., and Bray S. J., Enhancer Priming Enables Fast and Sustained Transcriptional Responses to Notch Signaling, Developmental Cell 50, 411 (2019). 31378591 10.1016/j.devcel.2019.07.002 PMC6706658

[16] Hoppe C., Bowles J. R., Minchington T. G., Sutcliffe C., Upadhyai P., Rattray M., and Ashe H. L., Modulation of the Promoter Activation Rate Dictates the Transcriptional Response to Graded BMP Signaling Levels in the Drosophila Embryo, Developmental Cell 54, 727 (2020). 32758422 10.1016/j.devcel.2020.07.007 PMC7527239

[17] Tantale K., Garcia-Oliver E., Robert M.-C., L’Hostis A., Yang Y., Tsanov N., Topno R., Gostan T., Kozulic-Pirher A., Basu-Shrivastava M., Mukherjee K., Slaninova V., Andrau J.-C., Mueller F., Basyuk E., Radulescu O., and Bertrand E., Stochastic pausing at latent HIV-1 promoters generates transcriptional bursting, Nature Communications 12, 4503 (2021). 10.1038/s41467-021-24462-5 PMC8302722 34301927

[18] Bass V. L., Wong V. C., Bullock M. E., Gaudet S., and Miller-Jensen K., TNF stimulation primarily modulates transcriptional burst size of NF-*κ*B-regulated genes, Molecular Systems Biology 17, e10127 (2021). 34288498 10.15252/msb.202010127 PMC8290835

[19] Brouwer I., Kerklingh E., Leeuwen F. v., and Lenstra T. L., Dynamic epistasis analysis reveals how chromatin remodeling regulates transcriptional bursting, Nature Structural & Molecular Biology 30, 692 (2023). 10.1038/s41594-023-00981-1 PMC10191856 37127821

[20] Fukaya T., Lim B., and Levine M., Enhancer control of transcriptional bursting., Cell 166, 358 (2016). 27293191 10.1016/j.cell.2016.05.025 PMC4970759

[21] Pimmett V. L., Dejean M., Fernandez C., Trullo A., Bertrand E., Radulescu O., and Lagha M., Quantitative imaging of transcription in living Drosophila embryos reveals the impact of core promoter motifs on promoter state dynamics, Nature Communications 12, 4504 (2021). 10.1038/s41467-021-24461-6 PMC8302612 34301936

[22] Larsson A. J. M., Johnsson P., Hagemann-Jensen M., Hartmanis L., Faridani O. R., Reinius B., Segerstolpe r., Rivera C. M., Ren B., and Sandberg R., Genomic encoding of transcriptional burst kinetics, Nature 565, 251 (2018). 10.1038/s41586-018-0836-1 PMC7610481 30602787

[23] Zoller B., Little S. C., and Gregor T., Diverse Spatial Expression Patterns Emerge from Unified Kinetics of Transcriptional Bursting, Cell 175, 835 (2018). 30340044 10.1016/j.cell.2018.09.056 PMC6779125

[24] Little S., Tikhonov M., and Gregor T., Precise Developmental Gene Expression Arises from Globally Stochastic Transcriptional Activity, Cell 154, 789 (2013). 23953111 10.1016/j.cell.2013.07.025 PMC3778922

[25] Levo M., Raimundo J., Bing X. Y., Sisco Z., Batut P. J., Ryabichko S., Gregor T., and Levine M. S., Transcriptional coupling of distant regulatory genes in living embryos., Nature 605, 754 (2022). 35508662 10.1038/s41586-022-04680-7 PMC9886134

[26] Bertrand E., Chartrand P., Schaefer M., Shenoy S. M., Singer R. H., and Long R. M., Localization of ASH1 mRNA particles in living yeast., Molecular Cell 2, 437 (1998). 9809065 10.1016/s1097-2765(00)80143-4

[27] Larson D. R., Zenklusen D., Wu B., Chao J. A., and Singer R. H., Real-time observation of transcription initiation and elongation on an endogenous yeast gene., Science 332, 475 (2011). 21512033 10.1126/science.1202142 PMC3152976

[28] Garcia H. G., Tikhonov M., Lin A., and Gregor T., Quantitative imaging of transcription in living Drosophila embryos links polymerase activity to patterning., Current Biology 23, 2140 (2013). 24139738 10.1016/j.cub.2013.08.054 PMC3828032

[29] Lucas T., Ferraro T., Roelens B., Chanes J. D. L. H., Walczak A. M., Coppey M., and Dostatni N., Live imaging of bicoid-dependent transcription in drosophila embryos., Current Biology 23, 2135 (2013). 24139736 10.1016/j.cub.2013.08.053

[30] Liu J., Hansen D., Eck E., Kim Y. J., Turner M., Alamos S., and Garcia H. G., Real-time single-cell characterization of the eukaryotic transcription cycle reveals correlations between RNA initiation, elongation, and cleavage, PLoS Computational Biology 17, e1008999 (2021). 34003867 10.1371/journal.pcbi.1008999 PMC8162642

[31] Dubuis J. O., Samanta R., and Gregor T., Accurate measurements of dynamics and reproducibility in small genetic networks., Molecular Systems Biology 9, 639 (2013). 23340845 10.1038/msb.2012.72 PMC3564256

[32] Peccoud J. and Ycart B., Markovian modeling of gene-product synthesis, Theoretical Population Biology 48, 222 (1995).

[33] Zoller B., Nicolas D., Molina N., and Naef F., Structure of silent transcription intervals and noise characteristics of mammalian genes, Molecular Systems Biology 11, 823 (2015). 26215071 10.15252/msb.20156257 PMC4547851

[34] Corrigan A. M., Tunnacliffe E., Cannon D., and Chubb J. R., A continuum model of transcriptional bursting, eLife 5, e13051 (2016). 26896676 10.7554/eLife.13051 PMC4850746

[35] Lammers N. C., Galstyan V., Reimer A., Medin S. A., Wiggins C. H., and Garcia H. G., Multimodal transcriptional control of pattern formation in embryonic development, Proc. Natl. Acad. Sci. USA 117, 836 (2020). 31882445 10.1073/pnas.1912500117 PMC6969519

[36] Lagha M., Bothma J. P., and Levine M., Mechanisms of transcriptional precision in animal development, Trends in Genetics 28, 409 (2012). 22513408 10.1016/j.tig.2012.03.006 PMC4257495

[37] Schroeder M. D., Pearce M., Fak J., Fan H., Unnerstall U., Emberly E., Rajewsky N., Siggia E. D., and Gaul U., Transcriptional Control in the Segmentation Gene Network of Drosophila, PLoS Biology 2, e271 (2004). 15340490 10.1371/journal.pbio.0020271 PMC514885

[38] Perry M. W., Bothma J. P., Luu R. D., and Levine M., Precision of Hunchback Expression in the Drosophila Embryo, Current Biology 22, 2247 (2012). 23122844 10.1016/j.cub.2012.09.051 PMC4257490

[39] Berrocal A., Lammers N. C., Garcia H. G., and Eisen M. B., Kinetic sculpting of the seven stripes of the Drosophila even-skipped gene, eLife 9, e61635 (2020). 33300492 10.7554/eLife.61635 PMC7864633

[40] Berrocal A., Lammers N. C., Garcia H. G., and Eisen M. B., Unified bursting strategies in ectopic and endogenous even-skipped expression patterns, eLife 10.7554/elife.88671.1 (2023). PMC11627552 39651963

[41] Fukaya T., Dynamic regulation of anterior-posterior patterning genes in living Drosophila embryos., Current Biology 31, 2227 (2021). 33761316 10.1016/j.cub.2021.02.050

[42] Hülskamp M., Lukowitz W., Beermann A., Glaser G., and Tautz D., Differential regulation of target genes by different alleles of the segmentation gene hunchback in Drosophila., Genetics 138, 125 (1994). 8001780 10.1093/genetics/138.1.125 PMC1206124

[43] Sanchez A. and Golding I., Genetic Determinants and Cellular Constraints in Noisy Gene Expression, Science 342, 1188 (2013). 24311680 10.1126/science.1242975 PMC4045091

[44] Rayon T., Stamataki D., Perez-Carrasco R., Garcia-Perez L., Barrington C., Melchionda M., Exelby K., Lazaro J., Tybulewicz V. L. J., Fisher E. M. C., and Briscoe J., Species-specific pace of development is associated with differences in protein stability, Science 369, 10.1126/science.aba7667 (2020). PMC7116327 32943498

[45] Diaz-Cuadros M., Miettinen T. P., Skinner O. S., Sheedy D., Díaz-García C. M., Gapon S., Hubaud A., Yellen G., Manalis S. R., Oldham W. M., and Pourquié O., Metabolic regulation of species-specific developmental rates, Nature 613, 550 (2023). 36599986 10.1038/s41586-022-05574-4 PMC9944513

[46] Dar R. D., Razooky B. S., Singh A., Trimeloni T. V., McCollum J. M., Cox C. D., Simpson M. L., and Weinberger L. S., Transcriptional burst frequency and burst size are equally modulated across the human genome., Proc. Natl. Acad. Sci. USA 109, 17454 (2012). 23064634 10.1073/pnas.1213530109 PMC3491463

[47] Tsai A., Muthusamy A. K., Alves M. R., Lavis L. D., Singer R. H., Stern D. L., and Crocker J., Nuclear microenvironments modulate transcription from low-affinity enhancers, eLife 6, e28975 (2017). 29095143 10.7554/eLife.28975 PMC5695909

[48] Cho W.-K., Spille J.-H., Hecht M., Lee C., Li C., Grube V., and Cisse I. I., Mediator and RNA polymerase II clusters associate in transcription-dependent condensates., Science 361, 412 (2018). 29930094 10.1126/science.aar4199 PMC6543815

[49] Li J., Hsu A., Hua Y., Wang G., Cheng L., Ochiai H., Yamamoto T., and Pertsinidis A., Single-gene imaging links genome topology, promoter–enhancer communication and transcription control, Nature Structural & Molecular Biology 27, 1032 (2020). 10.1038/s41594-020-0493-6 PMC7644657 32958948

[50] Henninger J. E., Oksuz O., Shrinivas K., Sagi I., LeRoy G., Zheng M. M., Andrews J. O., Zamudio A. V., Lazaris C., Hannett N. M., Lee T. I., Sharp P. A., Cissé I. I., Chakraborty A. K., and Young R. A., RNA-Mediated Feedback Control of Transcriptional Condensates, Cell 184, 207 (2021). 33333019 10.1016/j.cell.2020.11.030 PMC8128340

[51] Nguyen V. Q., Ranjan A., Liu S., Tang X., Ling Y. H., Wisniewski J., Mizuguchi G., Li K. Y., Jou V., Zheng Q., Lavis L. D., Lionnet T., and Wu C., Spatiotemporal coordination of transcription preinitiation complex assembly in live cells, Molecular Cell 81, 3560 (2021). 34375585 10.1016/j.molcel.2021.07.022 PMC8420877

[52] Brückner D. B., Chen H., Barinov L., Zoller B., and Gregor T., Stochastic motion and transcriptional dynamics of pairs of distal dna loci on a compacted chromosome, bioRxiv , 2023.01.18.524527 (2023). 10.1126/science.adf5568 PMC10439308 37384691

[53] Tkačik G., Callan C. G., and Bialek W., Information flow and optimization in transcriptional regulation, Proc. Natl. Acad. Sci. USA 105, 12265 (2008), 0705.0313. 18719112 10.1073/pnas.0806077105 PMC2527900

[54] Jones D. L., Brewster R. C., and Phillips R., Promoter architecture dictates cell-to-cell variability in gene expression, Science 346, 1533 (2014). 25525251 10.1126/science.1255301 PMC4388425

[55] Hausser J., Mayo A., Keren L., and Alon U., Central dogma rates and the trade-off between precision and economy in gene expression, Nature Communications 10, 68 (2018). 10.1038/s41467-018-07391-8 PMC6325141 30622246

[56] Petkova M. D., Tkačik G., Bialek W., Wieschaus E. F., and Gregor T., Optimal Decoding of Cellular Identities in a Genetic Network, Cell 176, 844 (2019). 30712870 10.1016/j.cell.2019.01.007 PMC6526179

[57] Balakrishnan R., Mori M., Segota I., Zhang Z., Aebersold R., Ludwig C., and Hwa T., Principles of gene regulation quantitatively connect DNA to RNA and proteins in bacteria, Science 378, eabk2066 (2022). 36480614 10.1126/science.abk2066 PMC9804519

[58] Becker K., Balsa-Canto E., Cicin-Sain D., Hoermann A., Janssens H., Banga J. R., and Jaeger J., Reverse-Engineering Post-Transcriptional Regulation of Gap Genes in Drosophila melanogaster, PLoS Computational Biology 9, e1003281 (2013). 24204230 10.1371/journal.pcbi.1003281 PMC3814631

[59] McKnight S. L. and Miller O. L., Post-replicative nonribosomal transcription units in D. Melanogaster embryos., Cell 17, 551 (1979). 113103 10.1016/0092-8674(79)90263-0

[60] Rogers W. A., Goyal Y., Yamaya K., Shvartsman S. Y., and Levine M. S., Uncoupling neurogenic gene networks in the Drosophila embryo, Genes & Development 31, 634 (2017). 28428262 10.1101/gad.297150.117 PMC5411704

[61] Chen H., Levo M., Barinov L., Fujioka M., Jaynes J. B., and Gregor T., Dynamic interplay between enhancer–promoter topology and gene activity, Nature Genetics 50, 1296 (2018). 30038397 10.1038/s41588-018-0175-z PMC6119122

[62] Bothma J. P., Norstad M. R., Alamos S., and Garcia H. G., LlamaTags: A Versatile Tool to Image Transcription Factor Dynamics in Live Embryos, Cell 173, 1810 (2018). 29754814 10.1016/j.cell.2018.03.069 PMC6003873

[63] Gregor T., Wieschaus E. F., McGregor A. P., Bialek W., and Tank D. W., Stability and Nuclear Dynamics of the Bicoid Morphogen Gradient, Cell 130, 141 (2007). 17632061 10.1016/j.cell.2007.05.026 PMC2253672

[64] Liu F., Morrison A. H., and Gregor T., Dynamic interpretation of maternal inputs by the Drosophila segmentation gene network, Proceedings of the National Academy of Sciences 110, 6724 (2013). 10.1073/pnas.1220912110 PMC3637740 23580621

[65] Hayden L., Hur W., Vergassola M., and Talia S. D., Manipulating the nature of embryonic mitotic waves, Current Biology 32, 4989 (2022). 36332617 10.1016/j.cub.2022.10.014 PMC9691596

[66] deLeeuw J., Introduction to Akaike (1973) Information Theory and an Extension of the Maximum Likelihood Principle, in Breakthroughs in Statistics, Foundations and Basic Theory, Springer Series in Statistics (Springer, 1992) pp. 599–609.

[67] Andrieu C., Freitas N. D., Doucet A., and Jordan M. I., An introduction to MCMC for machine learning, Machine learning 50, 5 (2003).

[68] Andrieu C. and Thoms J., A tutorial on adaptive MCMC, Statistics and Computing 18, 343 (2008).

[69] Rosenthal J. S., Optimal Proposal Distributions and Adaptive MCMC, Handbook of Markov Chain Monte Carlo 4 (2010).

[70] Lestas I., Paulsson J., Ross N. E., and Vinnicombe G., Noise in Gene Regulatory Networks, IEEE Transactions on Automatic Control 53, 189 (2008).

[71] Walczak A. M., Mugler A., and Wiggins C. H., Computational Modeling of Signaling Networks, Methods in Molecular Biology 880, 273 (2012), 1005.2648. 23361990 10.1007/978-1-61779-833-7_13

[72] Gillespie D. T., Stochastic Simulation of Chemical Kinetics, Annual Review of Physical Chemistry 58, 35 (2007). 10.1146/annurev.physchem.58.032806.104637 17037977

[73] Sidje R. B., Expokit: a software package for computing matrix exponentials, ACM Transactions on Mathematical Software (TOMS) 24, 130 (1998).

