# Gene activity fully predicts transcriptional bursting dynamics

**Published:** 2024-06-28

**Authors:** Po-Ta Chen, Michal Levo, Benjamin Zoller, Thomas Gregor

**Affiliations:** 1 Joseph Henry Laboratories of Physics & Lewis-Sigler Institute for Integrative Genomics, Princeton University, Princeton, NJ 08544, USA; 2 Department of Biochemistry and Molecular Biophysics, Columbia University, New York, NY, USA; 3 Department of Stem Cell and Developmental Biology, CNRS UMR3738 Paris Cité, Institut Pasteur, 25 rue du Docteur Roux, 75015 Paris, France

## Abstract

Transcription commonly occurs in bursts, with alternating productive (ON) and quiescent (OFF) periods, governing mRNA production rates. Yet, how transcription is regulated through bursting dynamics remains unresolved. Here, we conduct real-time measurements of endogenous transcriptional bursting with single-mRNA sensitivity. Leveraging the diverse transcriptional activities in early fly embryos, we uncover stringent relationships between bursting parameters. Specifically, we find that the durations of ON and OFF periods are linked. Regardless of the developmental stage or body-axis position, gene activity levels predict individual alleles’ average ON and OFF periods. Lowly transcribing alleles predominantly modulate OFF periods (burst frequency), while highly transcribing alleles primarily tune ON periods (burst size). These relationships persist even under perturbations of cis-regulatory elements or trans-factors and account for bursting dynamics measured in other species. Our results suggest a novel mechanistic constraint governing bursting dynamics rather than a modular control of distinct parameters by distinct regulatory processes.

Eukaryotic transcriptional regulation is an inherently dynamic and stochastic process, orchestrated by a series of molecular events governing productive transcription initiation by individual RNA polymerases (Pol II complexes) [1, 2]. This process culminates in nascent RNA synthesis, which in turn shapes protein production, and thus dictates cellular identity and behavior in both space and time. Consequently, revealing the fundamental principles underpinning transcriptional dynamics is paramount for understanding and predicting cellular phenotypes.

Research on various biological systems, from yeast to mammalian cells, revealed that transcription occurs in bursts. These bursts involve the release of multiple Pol II complexes during an active phase, known as the “ON” period, followed by a quiescent “OFF” period [3–9]. However, several critical questions remain unanswered: How does the regulation of bursting kinetics determine mRNA production and transcriptional dynamics across developmental time and cell types? Is the transcription rate primarily regulated by adjusting the durations of the ON or OFF periods, the initiation rate (i.e., the rate of Pol II release during active phases), or a combination of these parameters? What is the spectrum of parameters utilized by tightly regulated genes, such as developmental genes? Do different genes employ distinct bursting strategies? Do these strategies vary in temporal and spatial (tissue-specific) transcriptional control, and how do they depend on the regulatory factors at play?

One hypothesis that has emerged from previous work suggests that different regulatory factors, including transcription factor (TF) binding, *cis*-regulatory elements, nucleosome occupancy, histone modification, Pol II pausing, and enhancer–promoter interactions, may influence distinct aspects of bursting dynamics [10–19]. For instance, it has been proposed that enhancers primarily impact burst frequency, while promoters primarily affect burst size [20–22]. However, integrating diverse observations into a unified and quantitatively predictive understanding of transcriptional control through bursting dynamics has proven to be challenging.

Our previous study [23], which relied on inference from a static snapshot of mRNA abundance, raised the possibility of a simple and unified control of bursting kinetics, even in complex developmental systems. Specifically, mRNA abundance of key developmental genes in fixed *Drosophila* embryos within a narrow developmental window (~ 10 min) could be accounted for by a straightforward two-state kinetic model of transcription, with a single free parameter. This motivated our current efforts to directly measure bursting dynamics rather than rely on specific kinetic assumptions to infer dynamics from a fixed sample. Moreover, by conducting measurements throughout cell cycles and under conditions where key regulatory determinants are perturbed, we aim to assess the impact of developmental time and regulation on bursting behavior.

Here we present real-time transcription measurements with single-mRNA sensitivity, characterizing the bursting parameters of cell fate-determining genes in early *Drosophila* embryos. Our findings unveil unexpected relationships between bursting parameters, revealing tight coupling between ON and OFF periods (or, alternatively, burst size and frequency). This coupling suggests that these distinct parameters are not solely governed by independent regulatory processes. Instead, any gene activity level is achieved through a specific combination of ON and OFF periods, irrespective of gene identity or spatiotemporal coordinates within the developing embryo. This stringent coupling thus holds across diverse regulatory landscapes. Indeed, we demonstrate through cis- and trans-perturbations that predominant modulation of burst size or frequency correlates with activity level rather than specific regulatory determinants. Low-activity alleles primarily modulate OFF periods, altering burst frequency, while mid-to-high-activity alleles primarily modulate ON periods, altering burst size. While previous measurements in various organisms, ranging from yeast to mammalian cells, sample a narrow range of activities, we find that they largely adhere to the elucidated dependencies. However, as these dependencies do not trivially arise from commonly implicated molecular events in transcription initiation, our work prompts a re-examination, guiding future investigations into the mechanisms of mRNA production.

## RESULTS

### Instantaneous single allele transcription rate measurements.

**FIG. 1.**
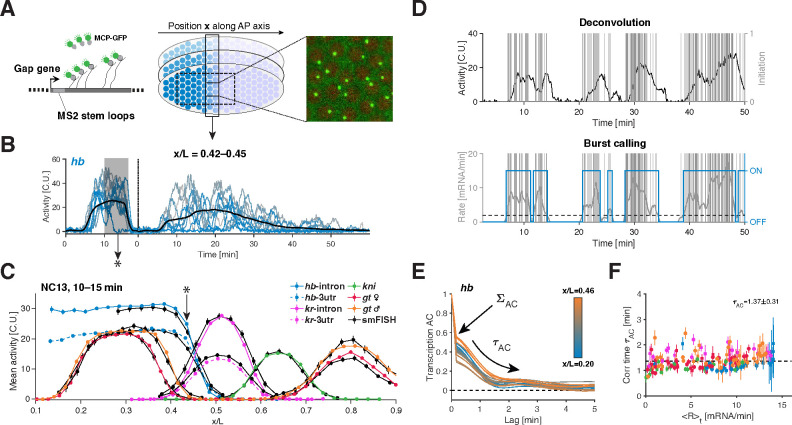
Live single-cell transcription rate measurements of endogenous gap genes. (A) Live fluorescence imaging of nascent transcripts using MS2 stem-loops measures single allele transcriptional activity (green hotspots) along the anterior-posterior (AP) axis of the fly embryo (also see Fig. S1A and Methods). (B) Transcription time series for 10 alleles (blue) of the gap gene *hunchback* (*hb*) at position x/L=0.435±0.010 sampled every 10 s. Low embryo-to-embryo variability (Fig. S1F) enables pooling alleles from multiple spatially and temporally aligned embryos (n=10-20) to average over 200–350 alleles at a given position (black). (C) Calibration of transcriptional activity in absolute units performed by matching mean spatial activity profiles from previously calibrated fixed smFISH measurements [23] (black) with 5-min-interval averages (gray shade in B) of live time series at each AP position (color) for all examined gap genes. A single global conversion factor matches live and fixed profiles to within 5% error (Fig. S1B), defining a unit for transcriptional activity (i.e., the cytoplasmic unit, C.U. [24]) equivalent to a fully tagged transcript. (D) Reconstruction of transcription initiation events (gray bars) and underlying bursts from single allele transcription time series (black). (Top) Bayesian deconvolution enables sampling possible configuration of initiation events (see Methods and Fig. S2A–B). (Bottom) Clustering of sampled initiation events (using a moving average of width ~1 min (gray curve) and a threshold at two mRNA/min (dashed line)) identifies individual bursts (blue). (E) Autocorrelation (AC) functions of single allele *hb* transcription rates averaged over time for different positions along the AP axis (color). AC functions are normalized by the variance; uncorrelated ($\Sigma_{AC}$) and time-correlated $\left(\tau_{AC}\right)$ components of the rate fluctuations are highlighted (see Fig. S5). (F) Correlation time $\tau_{AC}$ (from fitted exponentials, see Methods) of the single allele transcription rate as a function of mean transcription rate R (color code as in C). Dashed line corresponds to overall mean correlation time ${\overset{‾}{\tau }}_{AC}=1.37\pm 0.31\;min$. Error bars are 68% confidence intervals.

We developed a quantitative approach to measure endogenous bursting dynamics at a single allele level in living *Drosophila* embryos. To achieve this we utilized a versatile CRISPR-based scheme [25] to incorporate MS2 cassettes into intronic or 3’ untranslated regions (3’UTRs) of the gap genes. These cassettes form stem-loops in the transcribed nascent RNA, which are subsequently bound by fluorescent coat-proteins (Fig. 1A, S1A, and Methods) [26–29]. We employed a custombuilt two-photon microscope to generate fluorescence images, allowing us to capture RNA synthesis from one tagged allele per nucleus with nearly single-mRNA sensitivity (Fig. S1D–E). Our optimized field-of-view provided 10-second interval time-lapses (Fig. 1B) for hundreds of nuclei per embryo during a critical 1.5-hour period of embryonic development, specifically nuclear cycles 13 (NC13) and 14 (NC14), essential for robust statistical analysis (Fig. 1A–B; Videos V1–V4).

We calibrated our fluorescence signal using smFISH data to express our dynamic transcription measurements in terms of absolute mRNA counts (Fig. 1C and S1B–C, and details in Methods). This calibration, combined with the nearly single-transcript sensitivity of our measurements enabled us to reconstruct the underlying Pol II transcription initiation events for each allele using Bayesian deconvolution (see Methods). The convolution kernel we employed describes the fluorescent signal resulting from the release of Pol II complexes onto the gene, which subsequently engages in the elongation process [17, 21] (assuming constant and deterministic elongation, Fig. S2A). For each time trace, our Bayesian approach generates multiple configurations of transcription initiation events (Fig. 1D). By averaging these configurations, we obtained a time-dependent instantaneous single allele transcription rate, denoted as r(t) (Fig. S2B). Importantly this approach also provides corresponding error estimates, which we propagated in all subsequent analyses.

Our kernel-based deconvolution approach was validated by control measurements involving dual-color tagging of the gene body, both at 5’ and 3’ regions (Fig. S2C). These measurements support our key assumptions regarding the elongation process and the absence of co-transcriptional splicing (see Methods). Furthermore, they allow us to extract a Pol II elongation rate, denoted as $K_{elo}$, which we determined to be 1*.*8 ± 0*.*1kb/min. This value aligns with previous measurements reported in the literature [28, 30] (Fig. S2D–G).

With our approach, the extracted single allele transcription rates are no longer masked by the Pol II elongation dwell time, unlike the directly measured intensities. Instead, they capture initiation events (i.e., Pol II release for productive elongation). Consequently, these rates are independent of gene length, allowing for direct comparisons across different genes. This facilitated the intriguing observation that the gap genes average transcription rate, computed over nuclei per spatial position and time, denoted as R=⟨r⟩, reach a similar maximum $R_{max}=14.8\pm 0.9\;mRNA/min$ (Fig. S3A, Video V5). Moreover, these average transcription rates closely mirror the well-documented average protein dynamics [31]. Simple assumptions related to diffusion and lifetime, without the need for explicit post-transcriptional regulation, are sufficient to quantitatively predict protein patterns from the mean transcription rates R (Fig. S4 and Video V6). Thus, in this system, the functional output, namely protein synthesis, predominantly relies on transcription regulation. Our quantitative imaging and deconvolution approach paves the way for uncovering how this regulation emerges from the single-allele transcription dynamics.

### Single allele transcription rates hint at a universal bursting regime.

The gap genes differ in their transcriptional activities both spatially and temporally. However, when we examine the distributions P(r∣R) of single-allele transcription rates r that yield a similar mean transcription rate R, an intriguing pattern emerges. These distributions converge consistently across different genes (Fig. S3C, see Methods), suggesting the existence of a common transcriptional regime. For transcription rates in the low- to mid-range of R, we observe an abundance of non-transcribing or barely transcribing alleles. These distributions starkly contrast with a regime characterized by constitutive transcription. Conversely, for transcription rates in the high range of R, the distributions converge towards the constitutive, or Poissonian, regime (Fig. S3D), indicating a higher proportion of active ON alleles. These observations are consistent with the concept of transcriptional bursting, where an allele dynamically transitions between productive ON and quiescent OFF periods [3, 32].

We obtain additional support for a common bursting regime when we analyze the temporal dynamics of single-allele time traces. Bursting is expected to introduce temporal correlations in transcriptional activity, reflecting the persistence of the ON and OFF periods (Fig. S5A–B). To characterize such correlations, we compute auto-correlation functions for the deconvolved single-allele transcription rates. By using the deconvolved rates we effectively remove the correlated component arising from Pol II elongation along the gene and isolate only the correlations stemming from the initiation and the ON–OFF switching process. When we calculate these auto-correlation functions for different anterior-posterior (AP) bins, nuclear cycles, and various genes (Fig. 1E), we find striking similarities. An initial sharp drop at our sampling time scale (~10 s) indicates the presence of uncorrelated noise, consistent with independent Pol II initiation events (Fig. S5C). This drop is followed by a longer decay of correlated noise at a time scale denoted as $\tau_{AC}$, which we find to be confined within 1- to 2-minute range (Fig. 1F). The remarkable consistency of $\tau_{AC}$ across different spatial locations, genes, and transcriptional activity levels (spanning R, Fig. 1F) implies the preservation of this fundamental time scale of transcription dynamics. To delve deeper into potential regularities in bursting dynamics, our next step involves directly extracting individual bursts from single-allele time traces.

### Allele ON-probability is the primary transcription control parameter.

From the deconvolved initiation events along individual time traces (Fig. 1D top) we identify distinct ON and OFF periods of active and inactive transcription, respectively. The ON periods are characterized by consecutive initiation events (i.e., multiple Pol IIs released for productive elongation), while the OFF periods are transcriptionally inactive (Fig. 1D bottom). To delineate the transition of an allele from an OFF to an ON state, we employ a simple threshold on the moving average of the single allele transcription rate, set to 2 mRNA/min over a one-minute window. This criterion is selected based on our detection sensitivity, allowing us to reliably detect 1–2mRNA molecules, and the window is consistent with the time scale derived from the auto-correlation analysis (see Methods).

We conducted extensive testing to evaluate the impact of these detection parameters on our analysis, and our results confirm that our intuitive choice minimizes errors in burst characterization (see below, Fig. S9E). Importantly, the primary strength of this burst-calling routine lies in its exclusive reliance on a minimal clustering model. Consequently, it is inherently devoid of assumptions about the distributions of ON and OFF periods. As a result, our burst detection process remains agnostic to the underlying transcription models, as long as transcription can be described by at least one ON and OFF state (which is the case for common N-state models [9, 12, 17, 21, 33–35].

The next goal of our analysis is to elucidate how the consecutive switches between ON and OFF periods quantitatively govern the transcription rate R. Specifically, the mean transcription rate at time t, denoted as R(t), can be decomposed into two distinct parameters: the instantaneous probability of an allele being in the ON state $\left(P_{ON}(t)\right)$, representing the fraction of ON alleles at time t, and the mean initiation rate (K(t)) for ON alleles. Given the above decomposition, most of the variation in R could arise from changes in either K, $P_{ON}$, or both.

**FIG. 2.**
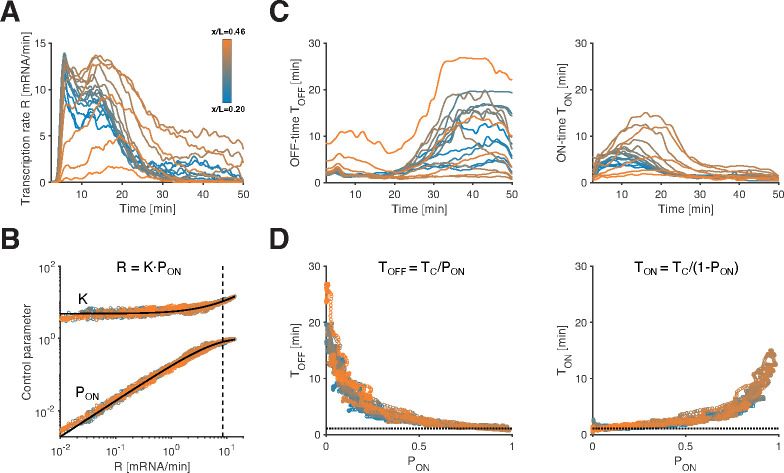
Direct estimation of instantaneous mean transcription parameters. (A) *Hb* mean transcription rate R as a function of time in NC14 (color encodes AP position). (B) Control parameters $P_{ON}$ and K as a function of mean transcription rate R in log space. Since $log(R)=log(K)+log\left(P_{ON}\right)$ by construction, changes in $P_{ON}$ determine changes in R below the dashed line $\left(R\sim 8.5\;mRNA/min\right.$, corresponding to $\left.P_{ON}=0.75\right)$. (C) *hb* mean OFF-time $T_{OFF}$ and mean ON-time $T_{ON}$ as a function of time in NC14 for all AP positions (color code as in (A)). (D) Mean OFF-time $T_{OFF}$ and mean ON-time $T_{ON}$ as a function of $P_{ON}$, for all positions and time points beyond the 7.5 min mark in (C). Dotted line corresponds to mean $T_{C}=1.1\pm 0.2$ (see Fig. S8E), which sets a lower bound on possible $T_{OFF}$ and $T_{ON}$ values.

Starting with the gene *hb*, we estimate the time-dependent parameters R(t) and $P_{ON}(t)$ for each AP bin. To obtain R(t), we calculate the average of ~250 single-allele instantaneous transcription rates (Fig. S6A). Concurrently, we determine $P_{ON}(t)$ by quantifying the fraction of alleles in the ON state at each time point (Fig. S6B). To compute K(t), we average initiation events restricted to the ON state. By repeating this procedure for all AP positions, we reveal the spatiotemporal variations in $P_{ON}$ and K (Fig. 2A, Fig. S6C–D). We validate our approach for burst calling and the recovery of bursting parameters from transcription time traces across a wide range of simulated data. We achieve an overall median error of 10%, gaining insights into the robustness of our analysis across the potential parameter space (see Methods, Fig. S9 and Fig. S10).

We find that all three parameters vary significantly across space and time (Fig. 2A and S6C–D). While we observe the expected interdependence $R=K\cdot P_{ON}$ (Fig. S6F), our analysis indicates that changes in R are primarily governed by changes in $P_{\text{ON}}$, while the influence of K is more moderate and less predictive of R (Fig. 2B and Fig. S7A). K only covers a two-fold change in dynamic range, which is marginal compared to R spanning from 0 to 15 (mRNA/min) (Fig. S6H–I). This two-fold change is largely due to the existence of two optically unresolved sister chromatids and a modest time dependence of K throughout the nuclear cycle (Fig. S7B–G, see Methods). With these considerations, we estimate for a single active chromatid the mean initiation rate at 6.3 ± 1.4mRNA/min and the mean Pol II spacing at 287±68 bp, consistent with the classic Miller spreads with average Pol II spacing of 330±180bp [36]. These results for *hb* suggest that transcriptional activity is predominantly controlled by the probability of an allele being in the ON state, and once in the ON state, transcription initiates at a quasi-constant rate.

Any given ON-probability can result from various combinations of mean ON and OFF periods, denoted as $T_{ON}$ and $T_{OFF}$. Indeed, since $P_{ON}=T_{ON}/\left(T_{ON}+T_{OFF}\right)$ near steady state, re-scaling both $T_{ON}$ and $T_{OFF}$ would achieve the same $P_{\text{ON}}$. This raises the possibility that alleles at different spatial positions and times employ distinct combinations, or there could be underlying regularities governing these periods. We computed the mean ON and OFF times $T_{ON}$ and $T_{OFF}$ (Fig. S8A–C; S8F for full distributions; see Methods), and found that these also vary substantially across space and time (Fig. 2C). However, when we plot $T_{ON}$ and $T_{OFF}$ against $P_{ON}$, we find that all data points collapse onto two tight anti-symmetric relationships (Fig. 2D). Despite the potential for multiple combinations of $T_{ON}$ and $T_{OFF}$ for any given $P_{ON}$, these relationships consistently associate a given $P_{ON}$ value with a unique pair of $T_{ON}$ and $T_{OFF}$ values, irrespective of spatial position or time.

The dynamic switching between ON and OFF states is associated with a correlation time $T_{C}$, which determines the time separation required for the transcription rate of a single allele to become uncorrelated. $T_{C}$ can be computed directly from the mean ON and OFF times using the equation $1/T_{C}=1/T_{ON}+1/T_{OFF}$ (Fig. S8E). For the *hb* gene, we find that $T_{C}$ is confined around 1.1 ± 0.2 min across all positions and time points, and seems independent of $P_{ON}$. Since $T_{ON}$ and $T_{OFF}$ can be expressed as functions of $P_{ON}$ and $T_{C}$ (via $T_{ON}=T_{C}/\left(1-P_{ON}\right)$ and $T_{OFF}=T_{C}/P_{ON}$, Fig. 2D and Fig. S8B), the value of $T_{C}$ sets the lower limit of $T_{ON}$ and $T_{OFF}$ (when $P_{ON}$ approaches one, or zero, accordingly). We thus find that mean ON and OFF periods share a similar minimal value on the order of 1–1.5min, which is larger than the minimal ON period we can reliably detect (~30 s, see Methods). Furthermore, the constancy of $T_{C}$ effectively links the mean ON and OFF times, and provides a mathematical explanation for the tight anti-symmetric relationships between $T_{ON},$ $T_{OFF}$, and $P_{ON}$ (Fig. 2D). Thus, not only does $P_{ON}$ govern the mean transcription rate R, but also the entire transcriptional bursting dynamics, with a characteristic combination of ON and OFF periods associated with any $P_{ON}$ value.

### Common bursting relationships underlie the regulation of all gap genes.

The gap genes differ in the composition, number, and arrangements of their *cis*-regulatory elements (Fig. S1A), resulting in distinct regulatory binding events (e.g., by transcription factors, transcription machinery components, and chromatin modifiers) [37]. Consequently, each gene displays unique spatiotemporal transcriptional activities (Fig. 1C, Video V5). Despite these differences, we find that the relationships governing bursting parameters for *hb* appear to generalize to other gap genes.

**FIG. 3.**
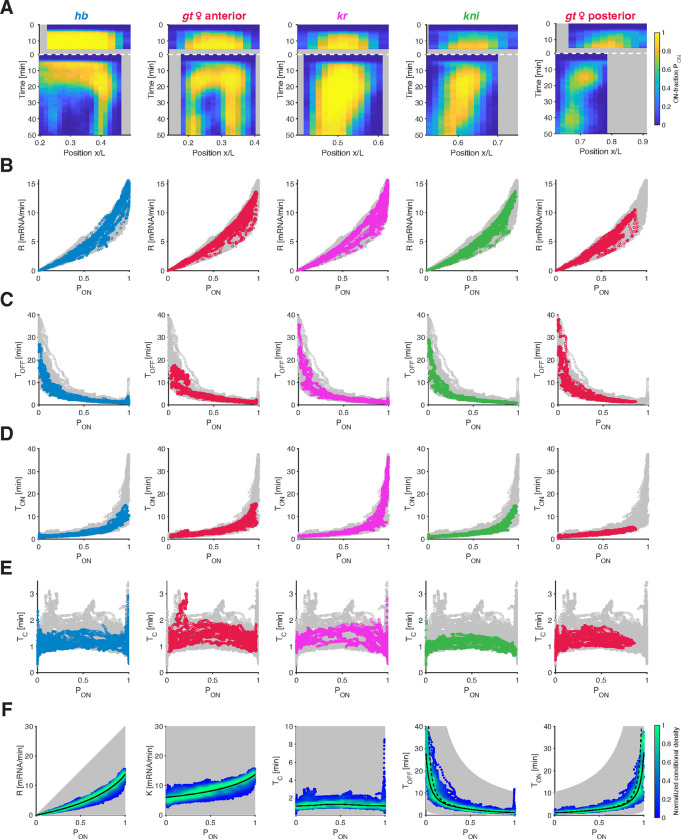
Transcription parameters collapse for all gap genes. (A) Kymographs of ON-probability $P_{ON}$ for all gap genes as a function of position and time for NC13 and NC14. The spatiotemporal transcriptional pattern of the gap genes arises from a complex regulation of $P_{ON}$ (color map). (B-E) Transcription parameters collapse for all gap genes across time and position. Transcription rate R (B), Mean OFF-time $T_{OFF}$ (C), ON-time $T_{ON}$ (D), and switching correlation time TC (E) as a function of $P_{ON}$. Colored data points represent individual gap genes (same color code as in (A), see Fig. S11A–B for gt male data). Each panel shows all the remaining genes in gray. (F) Density (color) of all data points across space and time of the transcription parameters for all gap genes, normalized by the maximum density. Potentially accessible space (gray shade) for plausible ranges of K (0.1–30 mRNA/min) and TC (0.1–10 min). $P_{ON}$ almost fully determines R and sets the combinations of $T_{OFF}$ and $T_{ON}$. For $T_{OFF}$ and $T_{ON}$, the dashed lines are the 2-state model predictions based on TC, and the solid lines take the finite recording length into account (see Fig. S11D).

When we applied our burst calling procedure (Fig. 1D) to the transcription time traces of other gap genes (*gt*, *Kr*, and *kni*), we obtained distinct spatiotemporal $P_{ON}$ profiles (Fig. 3A) that closely mirrored the gene-specific transcription rates R. Indeed, all genes exhibit nearly identical relationships between R and $P_{ON}$ (Fig. 3B) and between K and $P_{ON}$ (Fig. S11C), affirming that $P_{ON}$ is the predominant factor governing transcriptional activity across time, space, and genes.

Furthermore, the genes display similar relationships between $T_{OFF}$ and $P_{ON}$ (Fig. 3C) and between $T_{ON}$ and $P_{ON}$ (Fig. 3D). Thus, when different genes exhibit a specific $P_{ON}$ value, potentially at different spatiotemporal coordinates, the underlying $T_{ON}$ and $T_{OFF}$ periods are nonetheless largely identical. This finding can be related to the conservation of the switching correlation time $T_{C}$ across all positions, times, and genes (Fig. 3E), with the average $T_{C}$ value (1.25 ± 0.37 min) aligning quantitatively with the time scale predicted by the autocorrelation analysis above (Fig. 1F). Notably, the common bursting relationships apply not only across genes but also across distinct spatiotemporal domains of activity of a single gene, known to be driven by distinct enhancers [38, 39]. This is particularly evident in the large and yet distinct anterior and posterior domain of the gene *gt* (Fig 3A and Fig. S11). The use of similar bursting parameters in distinct spatial patterns of a single gene was indeed proposed in a study of a reporter construct of the gene *eve* [40, 41].

Pooling the parameters derived from all genes, times, locations, and embryos (comprising over 10^6^ data points) accentuates the limited subset of the parameter space utilized and underscores the stringent quantitative relationships emerging from our dataset (Fig. 3F). Overall, our analysis shows that, across all data points, the mean transcription rate R is primarily governed by $P_{ON}$, with only limited modulation of K. When we segregate the data into three developmental time windows, these relationships tighten even further, hinting at a modest developmental time dependence (Fig. S12A). Notably, the relationships obtained within the first developmental window generalize with greater precision (2-fold improvement) our sparse estimates using fixed measurements in a previous study, restricted to this window [23] (Fig. S13). Furthermore, once we convert our single allele parameters into their single gene copy equivalent, accounting for the combined contribution of two sister chromatids, the resulting relationships display even greater simplicity (Fig. S12B), with a near constant K and an almost linear R to $P_{ON}$ relationship.

The near-constant switching correlation time $T_{C}$ observed across the pooled data set, is associated with an apparent inverse proportionality between $T_{ON}$ and $T_{OFF}$, with a predominant modulation of one of these two parameters when $P_{ON}$ changes. While lowly-transcribing alleles (as characterized by $P_{ON}$) tend to achieve higher expression levels mainly by reducing $T_{OFF}$, medium-to-high-transcribing alleles are predominantly tuned by extending $T_{ON}$ (Fig. 3F). This observation means that changes in burst frequency $\left(F=1/\left(T_{ON}+T_{OFF}\right)\right)$ govern tuning of the transcriptional activity of low-transcribing alleles, while changes in burst size ($B=K\cdot T_{ON}$) exert greater influence on the tuning of medium-to-high-transcribing alleles (Fig. S11E,F).

The association between gene activity level and bursting parameters is discernible due to the wide range of transcriptional activities exhibited by the measured genes. This association holds consistently across genes, spatial locations, and time points (see Fig. 3F, Fig. S11, Fig. S12), indicating its relevance across diverse regulatory landscapes, which may involve varied TF concentrations, chromatin accessibility, and distinct activating enhancers. However, predicting the direct impact of individual regulatory determinants on bursting kinetics is challenging, given the multitude of factors collectively shaping transcriptional outcomes. Thus, targeted perturbation experiments are necessary to elucidate the specific effects of each determinant on bursting behavior.

### Common bursting relationships predict effects of *cis*- and *trans*-perturbations.

Diverse regulatory determinants, including *cis*-regulatory elements like enhancers and *trans*-factors such as TF repressors, contribute to controlling transcriptional activity. It is commonly assumed that distinct regulatory mechanisms directly influence specific bursting parameters. Thus, we sought to perturb various regulatory determinants to assess whether they produce distinct effects on bursting dynamics or if the relationships identified from wild-type measurements can account for the modified transcriptional activity upon perturbations.

**FIG. 4.**
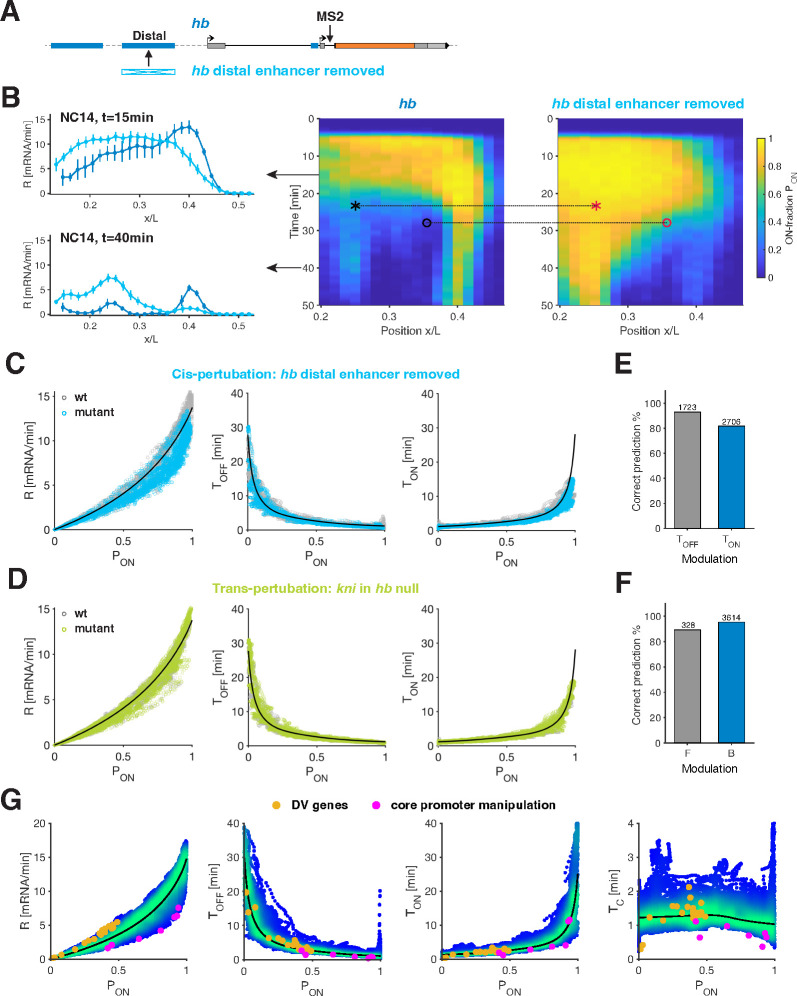
Effect of *cis*- and *trans*-perturbations on ON and OFF times. (A) Distal hb enhancer removal from the fly line carrying MS2 stem-loops in the endogenous hb locus. (B) Quantification of hb wild-type and mutant phenotypes. Both transcription rate R (left) and $P_{ON}$ (right) levels display significantly different expression patterns for the enhancer deletion mutant. Black arrows indicate the time points (15 and 40 min into NC14) in the kymograph at which rate profiles are depicted. “o” and “⋆” mark two bins with predominant $T_{OFF}$ modulation and predominant $T_{ON}$ modulation, respectively. (C) Transcription parameters for hb enhancer deletion (cyan) collapse on corresponding wild-type parameters (gray). (D) Transcription parameters for kni (green) in a hb null background (i.e. a trans-mutation, Fig. S14D–E) collapse on corresponding wild-type parameters (gray). Solid black lines in (C) and (D) correspond to the endogenous bursting relationships from Fig. 3F. (E-F) Verification of predicted changes in $T_{OFF}$ and $T_{ON}$ (E) (or changes in burst size B and frequency F (F)) for all wild-type and mutant $P_{ON}$ pairs (two example pairs shown in (A) for hb). For most pairs (> 85%) the prediction is correct (see also Fig. S15A–C. (G) Transcription parameters computed from two other Drosophila studies (yellow circles [16] and pink circles [21], respectively) are consistent with the gap genes relationships (black lines, data density color coded as in Fig. 3F).

Upon endogenous deletion of the distal enhancer of *hb*, we observe significant alterations in transcriptional activity (Fig. 4A–B, Video V7), including increased or decreased activity, at different times and locations along the AP axis, consistent with previous findings [42]. However, we find that bursting dynamics in this mutant still adhere to the relationships identified in the wild-type context. Specifically, transcription rates across different spatial and temporal coordinates are again governed by $P_{ON}$, the stringent relationships between $T_{ON}/T_{OFF}$ and $P_{ON}$ hold, and the switching correlation time $T_{C}$ remains broadly conserved around 0.9 ± 0.2 min (Fig. 4C and S15A–D).

Two additional perturbations further confirmed these findings. Deleting the distal enhancer of *kni* results in a significant reduction in *kni* activity (Fig. S14A–B, Video V8). Although the mutant exhibits a narrower dynamic range of activity, we observe a similar data collapse within this reduced range (Fig. S14C and S15A–D). Next, we explored the effect of a *trans*-perturbation by measuring *kni* activity in embryos with a *hb* null background (Fig. S14D–E, Video V9). This *trans*-perturbation significantly alters *kni* activity, consistent with earlier studies [43]). However, the underlying bursting dynamics again collapse onto the same busting relationships (Fig. 4D and S15A–D).

The consistency of $P_{ON}$ to $T_{ON}$ and $T_{OFF}$ relationships in wild-type and mutants suggests that we can predict how ON and OFF periods change upon a perturbation. Such a prediction relies solely on how the activity level, captured by $P_{ON}$, changed, and thus should remain valid at any given spatiotemporal coordinate. Remarkably, for each type of perturbation, we observe both predominant $T_{ON}$ and predominant $T_{OFF}$ modulation, at different spatiotemporal coordinates (Fig. 4E). Comparing predictions based only on the wild-type-derived relationships with the directly measured $T_{ON}$ and $T_{OFF}$ from the mutant, we find agreement as to which parameter was primarily altered in more than ~86% of cases for all spatiotemporal coordinates (Fig. 4E and S15E,G and I). Additionally, similar successful predictions are achieved when assessing the change in transcriptional activity in terms of altered burst size versus burst frequency (Fig. 4F and S15F,H and J). These findings challenge previous intuitions linking perturbations of specific regulatory elements or mechanisms to changes in a particular bursting parameter. Instead, these findings suggest the predictive power of $P_{ON}$, a proxy of the transcription activity, across different perturbations.

To further explore the generality of these observations, we examined data from two previous studies in the early fly embryo. One focused on the transcriptional effect of BMP signaling, a dorsoventral (DV) morphogen [16]. Transcription of a BMP target gene, u-shaped (ush), was measured across different DV positions and under ectopic signaling. A second study employed synthetic reporter constructs to examine the transcriptional effect of two core promoter motifs (TATA box and Initiator) [21]. These studies pointed to the modulation of distinct bursting parameters, and while the analyzed genes and perturbed regulatory determinants differ from those we measured, we found the datasets collapse onto our identified bursting relationships (Fig. 4G).

As suggested in these studies, the first dataset shows predominantly $T_{OFF}$ modulation, while the latter study has primarily $T_{ON}$ changes. Intriguingly, when plotted in the context of the full spectrum of $P_{ON}$ values captured by our measurements, the two independent datasets cluster in disjoint halves (Fig. 4G). Our analysis raises the possibility that the predominantly changed parameter ($T_{OFF}$ versus $T_{ON}$) might not be inherent to the examined regulatory manipulation (e.g., input TF concentrations or core promoter elements), but rather a consequence of the expression range (the $P_{ON}$ regime) of these genes.

### Data across different organisms is consistent with the identified bursting relationships

The conserved nature of the transcription machinery and regulatory mechanisms across eukaryotes, suggests that fundamental properties, likely reflecting molecular constraints, apply to numerous systems [44]. However, technical and biological factors currently hinder the ability to perform direct measurements of bursting dynamics and quantitatively compare parameter values or dependencies across diverse settings. For example, our analysis above highlights the necessity of a large dynamic range of gene activity to reveal underlying relationships. Yet, a large dynamic range is not readily observed in many setups. Using absolute units (e.g., calibrating arbitrary fluorescent units to mRNA counts) is crucial for estimating measurement sensitivity and facilitating comparisons across genes, perturbations, and systems. Additionally, an analysis that decouples the contribution of different biological steps to the measured signal (e.g., transcription initiation, elongation and mRNA half lives) is essential. These considerations guided our reexamination of data in yeast and mammalian cells.

**FIG. 5.**
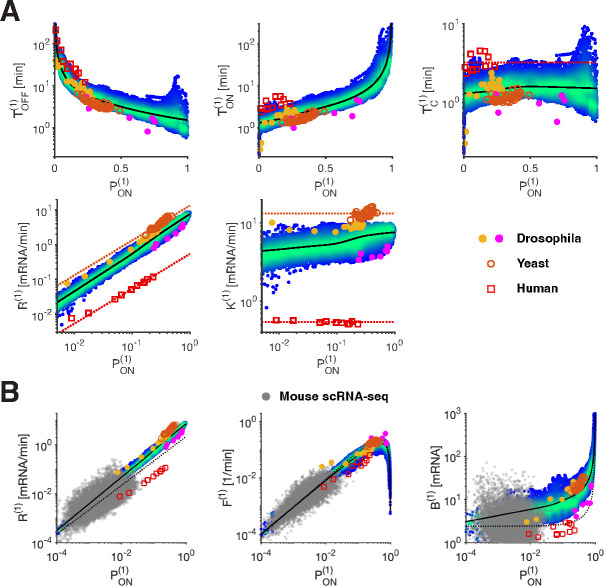
Generality of bursting relationships. (A) Scatter plots of the transcription parameters for a single gene copy (see Method) versus $P_{ON}$ (color code as in Fig. 3F). As in Fig. 4G, transcription parameters computed from two other *Drosophila* studies (yellow circles [16] and pink circles [21]. Transcription parameters resulting from multiple perturbations performed on the yeast *GAL10* gene (orange circles [19]) also closely follow our relationships (black lines), suggesting that these may apply beyond *Drosophila*. Transcription parameters estimated using our deconvolution approach on 11 human genes (red squares [9, 45]) further highlight the constant nature of $T_{C}$ and K (see Methods). (B) Transcription rate R, burst frequency F and burst size B for a single gene copy versus $P_{\text{ON}}$. Transcription parameters estimated from single-cell RNA-seq in mouse cells (gray circles [22]) are mostly consistent with our relationships (black lines), though parameters are likely underestimated due to low mRNA recovery rate (~10–30%) as demonstrated in original study. Black dotted lines correspond to expected relationships in scRNA-seq data assuming constant K and $T_{C}$.

To facilitate comparison, we computed the equivalent single gene copy parameters for all the fly data (Fig. S12B and Fig. S16, see Methods), assuming the independence of the two unresolved sister chromatids [24]. We examined bursting parameters derived from extensive perturbations of a yeast gene [19], and found strong agreement with our observations from the early fly embryo (Fig. 5A and Methods). While the yeast data points span a relatively small range of bursting parameters, compared to the developmentally regulated *Drosophila* genes, they are consistent with the $T_{ON}$-to-$P_{ON}$ and $T_{OFF}$-to-$P_{ON}$ relationships and show a highly constrained $T_{C}$ value of 1.1 ± 0.1 min, within our observed range.

Additionally, we re-analyzed transcription time traces from 11 endogenous human genes imaged with the MS2 system in cell lines [9, 45]. We aimed to extract promoter ON-time, capturing initiation, rather than previously reported ON-times encompassing initiation and mRNA dwell-time. We calibrated the signal in absolute units using smFISH measurements of nascent mRNA from one of these genes (*TFF1*) [45], and we performed a fluctuation analysis to assess measurement noise and mean mRNA dwell time (see Methods). We deconvolved initiation events and estimated bursting parameters for these genes, acknowledging longer mRNA dwell-time (6–16 min vs 1–2 min in flies), lower temporal resolution (100 s), and measurement noise as contributors to further uncertainties in these estimates (see Methods). The resulting parameters closely match those obtained from the fluctuation analysis performed in the original study on the gene *TFF1* [45].

Across genes, we find that both the initiation rate K and the switching correlation time $T_{C}$ are mainly constant, around K=0.54±0.02mRNA/min and 3.2 ± 0.8 min, respectively (Fig. 5A). While absolute values for K, and to a lesser extent $T_{C}$, appear to differ from fly genes (12- and 2-fold difference, respectively), the predictive power of $P_{ON}$ is preserved, and the human genes follow very similar relationships.

Thus, while the initiation rate K appears mostly conserved across genes and conditions in a given organism, we observe a clear difference between the three species that we probed (yeast K=13.2±1.3, fly K=6.2±1.8 and human K=0.54±0.02; Fig. 5A). Such differences may highlight species-specific metabolic variations [46, 47]. However, the $T_{C}$ values seem very close across species. Importantly, we find that the extracted relationships between bursting parameters hold across these datasets. They are therefore general, likely reflecting conserved underlying mechanisms.

Two high-throughput studies conducted in mammalian systems utilized vastly different approaches (library of reporters and single-cell RNAseq), but also indicate trends that align with our established bursting relationships [22, 48]. Yet, long mRNA dwell times (on the order of hours) complicate the extraction of initiation dynamics in these studies, and the quantitative comparison of bursting parameters. Nevertheless, we were able to reanalyze the scRNA-seq data in mouse cells [22], by fitting the steady-state mRNA distributions (as in the original study), with an additional weak prior on $T_{C}$ (see Methods). The resulting fits are as good as the original ones (marginal loss in likelihood), and provide parameter estimates with plausible physical scales. Overall, scRNA-seq parameters are consistent with our bursting relationships (Fig. 5B), highlighting the predictive power of $P_{ON}$. Furthermore, our analysis highlights the potential of using parameters derived from live imaging to interpret scRNA-seq data in terms of physical kinetic rates, which can be linked to the underlying molecular events.

## DISCUSSION

In this study, we developed a method to quantify real-time single allele transcriptional bursting in the developing early *Drosophila* embryo. The system’s wide range of transcriptional activities allowed us to uncover fundamental relationships governing bursting dynamics. We found a highly restrictive regime with strong dependencies between bursting parameters, characterized by tight asymmetric relationships between $T_{OFF},$ $T_{ON}$, and $P_{ON}$, underpinned by a relatively low and largely constant time scale $\left(T_{C}\right)$. Importantly, these relationships are consistent with data from yeast and mammalian cells[19, 22, 45], indicating that gene activity, predominantly governed by $P_{ON}$, predicts the characteristic ON and OFF times utilized across systems.

**FIG. 6.**
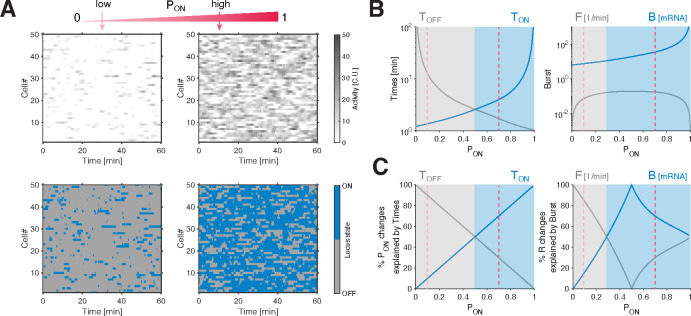
Gene activity as a predictor of bursting dynamics (A) Heatmaps of simulated single allele activity (top) and of corresponding ON-OFF gene state (bottom) for a low and high $P_{ON}$ regime, as predicted from our bursting relationships (see B). (B) The bursting relationships extracted from our data characterize single-allele bursting dynamics, as shown in A. (C) Fractional changes in activity explained by $T_{OFF}$ and $T_{ON}$ (left), and explained by burst frequency F and size B (right). For low $P_{ON}<0.5$, changes in activity are predominantly dictated by changes in $T_{OFF}$, while for high $P_{ON}>0.5$ by changes in $T_{ON}$. Similarly, for low $P_{ON}<1/3$, changes in activity are mainly dictated by changes in F, while for high $P_{ON}>1/3$ by changes in B.

Low activity alleles exhibit longer OFF times and shorter ON times, while high activity alleles $\left(P_{ON}>0.5\right)$ display longer ON times and shorter OFF times (Fig. 6A–B). Changes in activity level are associated predominantly with shortening OFF periods at low activity and primarily with prolonging ON periods at mid-to-high activity (Fig. 6C, left). Correspondingly, transcriptional tuning of either very low or very high activity alleles is associated with larger changes in burst frequency $\left(F=1/\left(T_{ON}+T_{OFF}\right)\right)$. However, while changes in burst frequency predominate over changes in burst size at low activity $\left(B=K\cdot T_{ON}\right)$, at mid-to-high activity, changes in burst size take over (Fig. 6C, right).

Our findings do not rely on parameters inferred by fitting specific mechanistic models of transcription to the data. Instead, we directly identify ON and OFF periods from single-allele initiation events. The elucidated bursting dynamics further align with single-allele timetrace auto-correlation analysis, which refrains from any ON-OFF calling. Thus, our bursting relationships are effective and broadly applicable across various models. However, while not originating from model-specific kinetic assumptions, these relationships impose constraints on the kinetic parameters of any underlying transcription model. Any detailed N-state model must adhere to these relationships once coarsely adapted to produce ON and OFF periods akin to those observed in the data [9, 12, 21, 33].

Since, to the best of our knowledge, the observed relationships do not trivially map to commonly invoked molecular processes shaping transcriptional dynamics, our findings suggest a novel mechanistic constraint. Our work raises the need to elucidate molecular mechanisms that integrate various regulatory processes into the control parameter $P_{ON}$. Furthermore, the largely constant and relatively small value of $T_{C}$ raises intriguing questions about its molecular implementation. The measured value of 1 min is reliably above our detection capability (see Methods), yet small given the theoretically accessible parameter space (Fig. 4E). This value further corresponds to the shortest ON and OFF durations that we observe (at $P_{ON}$ close to 0 or 1, correspondingly, and given $T_{ON}=T_{C}/\left(1-P_{ON}\right)$ and $\left.T_{OFF}=T_{C}/P_{ON}\right)$. It is non-trivial that these periods display a similar minimal duration, as potentially distinct molecular events might govern the ON and OFF states. Our analysis across genes, perturbations, and organisms suggests a mechanism that operates independently of the specific gene locus and might be conserved across eukaryotic systems. It is intriguing to consider that the tight linkage between ON and OFF periods could stem from more recently appreciated aspects of the transcriptional environment such as nuclear architecture or the assembly and disassembly of transcription machinery components [49–54].

Intriguingly, the identified bursting relationships align with a naive ON-OFF bursting regime where periods have a lower limit, and any $P_{ON}$ is encoded with the shortest ON-OFF combination, i.e., minimizing $T_{ON}+T_{OFF}$ (or maximizing burst frequency, Fig S17). These two simple assumptions give rise to a narrow parameter space utilized, asymmetric relationships for $P_{ON}$ versus $T_{OFF}$ and $T_{ON}$, and a consistently low $T_{C}$ throughout the full $P_{ON}$ range. Such a regime could benefit developmental regulation by permitting fine-tuning gene expression with minimal resource expenditure.

More generally, any bursting regime characterized by a preserved low value of $T_{C}$ offers noise minimization as bursts are easily buffered by longer mRNA lifetimes. It also offers swift responsiveness as transcription outcomes rapidly adjust to input TF changes (Fig. S8C–D). Thus, similar to other fields where organizing principles are emerging, such as optimized information flow and others [55–59], the elucidated relationships offer insights into the functionality encoded by complex processes and guide future investigations into conserved mechanisms at their core.

## METHODS

### I. FLY STRAINS AND GENETICS

#### A. Plasmid construction

MS2 and PP7 stem-loops cassettes were produced by a series of cloning steps, duplicating the annealed oligos below. The final cassette consists of 24 stem-loops (12 repetitions of the initial annealed oligos)

- MS1 oligo 1: CTAGTTACGGTACTTATTGCCAAGAAAGCACGAGCATCAGCCGTGCCTCCAGGTCGAATCTTCAAACGACGACGATCACGCGTCGCTCCAGTATTCCAGGGTTCATCC
- MS2 oligo 2: CTAGGGATGAACCCTGGAATACTGGAGCGACGCGTGATCGTCGTCGTTTGAAGATTCGACCTGGAGGCACGGCTGATGCTCGTGCTTTCTTGGCAATAAGTACCGTAA
- PP7 oligo 1: CTAGTTACGGTACTTATTGCCAAGAAAGCACGAGACGATATGGCGTCCGTGCCTCCAGGTCGAATCTTCAAACGACGAGAGGATATGGCCTCCGTCGCTCCAGTATTCCAGGGTTCATCC
- PP7 oligo 2: CTAGGGATGAACCCTGGAATACTGGAGCGACGGAGGCCATATCCTCTCGTCGTTTGAAGATTCGACCTGGAGGCACGGACGCCATATCGTCTCGTGCTTTCTTGGCAATAAGTACCGTAA

All 2attP-dsRed plasmids were made by cloning homology arms into a previously used 2attp-dsRed plasmid [61]. All 2attB-insert plasmid were made by cloning the inserts into a previously used 2attB-insert plasmid [62]. Plasmid maps and cloning details are available upon request.

#### B. Transgenic fly generation

For the endogenous tagging of the gap genes (*hb*, *kni*, *Kr*, and *gt*) a two-step transgenic strategy was used. First, a CRISPR-mediated replacement of each locus was performed. Specifically, the upstream regulatory regions (including annotated enhancers) and coding regions were replaced by a 2attp-dsRed cassette. This CRISPR step was performed with the following guides:

**Table:** 

Gene	Guide1	Guide2	Stem loops insertion coordinates (in dm6)
* kni*	CTTGAAGCTCAT	GGGAGGGCTTGA	Intronic (3L:20694142)
	TAATTCCACGG	TTCGGGAAAGG	
* hb*	ATGAACACTCAT	GTCACGGCTAAG	Intronic (3R:8694188), 3utr (3R:8691669*)
	ACATATCCTGG	ACGCCTTAAGG	
*gt*	TCTTACGTGTAA	CGGCCGGCGAGG	Intronic (X:2428789)
	GAATTCATGGG	AAGTGAACGGG	
* Kr*	GTAAATCCCAGA	AAGACTTGAACC	Intronic (2R:25227036), 3utr (2R:25228873)
	TGTATAATTGG	AAATACACAGG	

* An additional ~ 1*.*5kb fragment of the gene yellow was placed downstream of the stem-loop cassette to insure sufficient length for signal detection.

Homology arms were amplified from genomic DNA of the *nos-Cas9/CyO* injection line (BDSC #78781). For *kni* and *gt*, loss of gap gene proteins was verified by antibody staining as previously described [31]. For all four genes segmentation defects previously ascribed to the loss of protein were observed. PCR verification was performed (i.e., from the dsRed to the flanking genomic regions). These lines are referred to as a gap gene null line (e.g., *hb*-null).

In a second step, the deleted region for each gap gene was PCR amplified from the *nos-Cas9/CyO* line and cloned into a 2attB plasmid. MS2 stem-loops (see description above) were cloned into the gene (see insertion position in the above table and fig. S1A). This 2attB-insert was subsequently delivered into the 2attp site of the corresponding gap gene line from step one by co-injection with phiC31 integrase (RMCE injection with ~ 0.25*μg/μl* [DNA] and hsp-PhiC31 DNA ~0.1*μg/μl*). Flies were screened for loss of dsRed and PCR verified for the presence of the insert in the correct orientation, with primers from inside the insert to the flanking genomic regions.

In addition to MS2 lines for each gap gene, two lines with dual (orthogonal) stem-loop systems (MS2 and PP7) on the same gene were produced. They were primarily used for elongation measurements (see Section VA0e and fig. S2C–G). We generated 1) a *hb* line with intronic insertion of MS2 and a 3’utr insertion of PP7, 2) a *Kr* line with intronic insertion of PP7 and 3’utr insertion of MS2 (insertion positions are as in the single stem-loop lines, see table above). An additional control line with a cassette of alternating MS2 and PP7 stem-loops [62] in the intronic *Kr* gene was used to assess imaging error (fig. S1D–E).

A *hb*-MS2 fly line was generated without the distal *hb* enhancer. To this end, the 2attB-insert included the deleted region from the *hb* locus, the MS2 (intronic) insertion, and a replacement of the distal enhancer (3R:8698553–8700369, dm6) by a fragment of the same length with the *lacZ* gene. The modified 2attB-insert was delivered into the *hb*-2attp site as described above. Plasmid maps and cloning details are available upon request.

#### C. Genetic crosses for live imaging

A fly line with the fluorophore (*yw; His2Av-mRFP; nanos>MCP-eGFP*) [28] was crossed with wild-type (Ore-R) flies to reduce the background fluorescence. Female offspring (*yw/+; His2Av-mRFP/+; nanos*>*MCP-eGFP/+*) was subsequently crossed with our CRISPR-MS2 transgenic fly lines.

##### a. Male gt-MS2 crossing scheme:

Since *gt* is on the X-chromosome, the above crossing scheme only works for female *gt-MS2* embryos. For male embryos, we followed a different crossing strategy, where Female *gt-MS2* flies are crossed with a 3-color fluorophore line (*yw; His2Av-mRFP; nanos>MCP-eGFP,nanos>PCP-mCherry*). The female offspring of that cross (*gt-MS2/+; His2Av-mRFP/+; nanos*>*MCP-eGFP,nanos>PCP-mCherry/+*) was then crossed with (*gt-MS2PP7-interlaced*) males. To ensure we measure male embryos, imaging was performed only with embryos expressing *gt-MS2* signal but not PP7-PCP-mCherry signal.

##### b. kni-MS2 in hb-null background crossing scheme.

Since *kni* and *hb* are both on Chromosome-III, we adopted a two-generation crossing scheme for this line. The first generation had two sets of crosses. A first fly line (*hb-null/Tm3sb*) was crossed with *hb-3’UTR-MS2*. A second fly line (*hb-null,kni-MS2/Tm3sb*) was crossed with a dual fluorophore line (*yw; His2Av-mRFP; nanos>MCP-eGFP*). Male offspring from the first cross (*hb-null*/*hb-3’UTR-MS2*, selected against Tm3sb) was then crossed with female offspring from the second (*His2Av-mRFP/+; nanos*>*MCP-eGFP/hb-null,kni-MS2*, selected against Tm3sb). Imaging was performed with embryos that had *kni-MS2* expression but without the *hb-3UTR-MS2* signal (spatially distinguishable within embryos). This ensured the measured embryos had a genotype of *hb-null,kni-MS2/hb-null* on Chromosome-III.

### II. LIVE IMAGING

#### A. Sample preparation

Sample preparation for live imaging is adapted from previous work [28, 63]. We prepare an air-permeable membrane (roughly 2 cm by 2 cm) on a sample mounting slide. Heptane glue is evenly distributed on the membrane. Since we record embryos starting from late nuclear cycle 12, the flies are caged on agar plates for 2 to 2.5 hours. Embryos laid on these plates in that time window are transferred to a piece of double-sided tape by a dissection needle with which the embryos are also hand-dechorionated and placed on the glued membrane. After mounting, we immerse embryos in Halocarbon 27 oil (Sigma) and compress the embryos with a cover glass (Corning #1 1/2, 18x18 mm). Excess oil is removed by a tissue if needed. All embryos are mounted dorsal up (dorsal side facing objective lens).

#### B. Imaging settings

Live embryo imaging is performed from late NC12 to the end of NC14 (onset of gastrulation) using a custom-built inverted two-photon laser scanning microscope (similar in design to previous studies [64, 65]). The laser sources for two-photon excitation are a Chameleon Ultra (wavelength at 920 nm, for channel 1) and a HighQ-2 from Spectra Physics (wavelength at 1045 nm, for channel 2). Excitation and emission photons are focused and collected by a 40x oil-immersion objective lens (1.3NA, Nikon Plan Fluor). The average laser power measured at the objective back aperture are 20 mW and 4 mW, for Ultra and HighQ-2 lasers, respectively. In dual color Imaging Error Experiments (Section 4.4) 20 mW was used for both channels. Laser scanning and image acquisition are controlled by ScanImage 5.6–1 (Vidrio Technologies, LLC). Fluorescence signal from both channels is simultaneously detected by separated GaAsP-PMTs (both Hamamatsu H10770P-40). The pixel size is 220 nm, with an image size of 960×540 pixels. Each image stack contains 12 frames, separated by 1 *μ*m in the axial z-direction. Pixel dwell time is 1.4 *μ*s. The overall temporal resolution for one image stack is around 10 s.

### III. IMAGE PROCESSING

#### A. Nuclei tracking

Nuclei tracking was performed on a specifically dedicated image acquisition channel with red fluorophores, i.e. redfluorescently labeled histone proteins (His2Av-mRFP) that are provided maternally in all imaged embryos. Typical imaging windows span from late NC12 to late NC14 during embryonic development, when the embryos undergo two rounds of nuclear division. As during this period, the size of the nuclei changes continuously (from ~12*μ*m to ~7*μ*m in diameter), we use adaptive parameters for nuclei filtering and identification. First, as a pre-filter, a simple 2D Gaussian filter was applied to the Z-projections of the image stack of the red nuclei channel. This procedure roughly captures the average nuclear size of the population at each time point. Second, this time-dependent mean nuclear size is used to construct dynamic parameters for a 3D difference of Gaussian (DOG) filter to smooth the nuclei images, whose spherical shape is enhanced with a disk filter. For each 3D embryo image stack (per time point), filtered images are normalized to the maximum pixel intensity and subsequently binarized and segmented using a watershed algorithm. From each of these 3D segmented image stacks we constructed a 2D Voronoi diagram. For consecutive time points, the identity for all nuclei is propagated based on the shortest pair-wise distance of centroids and the largest overlapping fraction of Voronoi surfaces.

#### B. Transcription spot tracking and signal integration

To detect low-intensity signal-to-noise ratio (SNR) spots, our detection algorithm is tuned to high sensitivity at the cost of increasing the false positive rate when no obvious bright object throughout the detection region is present. Thus, we could find multiple spot candidates per nucleus at a given time point. However, in all single-allele-labeling experiments, we expect at most one spot per nucleus at all times. With this knowledge, a dedicated custom detection and tracking (modified Viterbi) algorithm identifies and tracks the real low SNR transcription spots based on the spot candidates.

##### a. Detecting spot candidates and transcription trajectories.

Identification of transcription spots is simplified by dividing the original images into 3D image stacks containing individual nuclei. The transcription channel for each such stack, tracked across time, is filtered for salt-and-pepper noise with a 3D median filter and subsequently, a 3D difference of Gaussian (DOG) filter is applied to detect round, concave objects. These objects are initial candidates for identifying transcription active sites. If consecutive time points present multiple spot candidates, we determined the most likely association using the pair-wise distance between potential pairs and a diffusion-based potential. The latter is constructed iteratively from the random walk of all transcription spots across all time points. This potential is penalizing for less likely displacements. This procedure identifies the most likely trajectory for each nucleus.

##### b. Signal integration and transcription time-series.

We calculated the mean pixel intensity of a 3D ellipsoid volume centered at the centroid coordinates of each transcription spot as identified by the spot-tracking procedure. The ellipsoid volume has diameters of (X, Y, Z)=(7, 7, 5) pixels, determined empirically based on the objective point spread function and the image pixel size (see image settings). Similarly, the mean local fluorescence background was estimated by averaging the intensities of all pixels in a larger 3D ellipsoid (i.e. (X, Y, Z)=(19, 19, 7) pixels, also centered at the spot centroid), except for the pixels of the inner spot ellipsoid. The fluorescence intensity of transcription spots is then quantified by subtracting this mean local background value from the mean spot intensity. This procedure is performed to avoid intensity contributions of freely floating MCP-GFP molecules within the nuclei from the transcription spot intensities. Our detection and tracking pipeline results in background-subtracted intensity time series, each corresponding to the real-time transcriptional activity of a single labeled allele within a nucleus.

##### c. AP position assignment.

The anterior-posterior axis (AP) was determined based on a mid-sagittal image of the full embryo in the histone-RFP channel. The tips of the long axis of the full embryo set the positions of both the anterior and posterior poles. Both poles are routinely checked manually. The XY coordinates of the nuclei identified in the surface images are registered onto the mid-sagittal image to determine their AP coordinates in that reference frame, relative to the poles.

### IV. DATA PROCESSING, CALIBRATION, AND MEASUREMENT ERRORS

#### A. Spatiotemporal alignment of embryos

All data sets (consisting of multiple embryos) are realigned in time (with respect to mitotic events) and space to minimize inter-embryo variabilities. These are stemming from differences in the speed of developmental progression (mainly due to temperature fluctuations at the sample) and from misalignment in space resulting from the AP-axis determination.

##### a. Developmental time normalization and temporal alignment.

To determine the speed of developmental progression, we compute for each nucleus in each embryo the time between mitosis 12 and 13, i.e., the duration of nuclear cycle 13 (NC13) $t_{13}$. $t_{13}$ is thus the time for individual nuclei between two mitotic events (that way accounting for the well-documented mitotic waves that propagate along the AP-axis [66]. It is identified when our nucleus-segmentation algorithm detects two separate entities (calling error ~10s). The embryo-intrinsic developmental time is determined by estimating the average duration of NC13 over all nuclei of a given embryo, $\langle t_{13}\rangle \;=\;T_{13}$. The global mean measured over *n* = 123 embryos is $\left\langle T_{13}\right\rangle =\;18.4\;\pm \;1.2min$, corresponding to 6*.*5% variability at 24 ± 1°C.

While most of our transcription measurements are performed during NC14, the mean duration of NC14 $T_{14}$ is difficult to measure as the end of the cycle occurs beyond gastrulation and the completion of the cycle is asynchronous among cells. Even the onset of gastrulation (cells no longer form a monolayer) is difficult to time precisely due to the way our imaging is performed: specimen labeling is optimized to measure transcription, which does not allow for optimal determination of the onset of gastrulation. However strong correlations of the durations of earlier cycles ($T_{11}$, $T_{12}$ and $T_{13}$, Pearson correlation $\rho_{11–12}=0.75$ and $\rho_{12–13}=0.81$) suggest that $T_{13}$ serves as a good proxy for the speed of developmental progression. Thus each embryo is aligned in time to match this unified speed. Temporal alignment is achieved in each embryo by normalizing the sampling time Δt for both NC13 and NC14 by $T_{13}/\langle T_{13}\rangle$, leading to $\Delta t\prime \;=\;\Delta t\langle T_{13}\rangle /T_{13}$. Consequently, all embryos are aligned with respect to the onset and the end of NC13 (and thus the onset of NC14), making further translational temporal shifts between embryos unnecessary. The resulting temporal alignment typically provides a reduction in embryo-to-embryo variability (defined as the variance across mean embryo transcriptional activity) by a factor of two at 15 min into NC13 (near the end of NC13).

##### b. Spatial alignment.

All nuclei are projected on each embryo’s internally determined AP-axis. However, axis determination is error-prone due to image analysis constraints, small changes in the azimuthal angle of the embryo with respect to the optical axis, and small tissue deformation due to the compression from the coverslip. We thus adjusted the spatial alignment for each embryo by correcting for possible errors stemming from these technical constraints. Indeed, the embryo elliptic mask fit (see Section IIIB0c; AP position assignment) together with small deformation of the embryo surface and natural variability typically leads to a positional error of ~1% egg length (corresponding to approximately one cell diameter, which is statistically significant to our subsequent analyses, see also [23]). Spatial alignment is achieved directly on the image stack that measures gene activity. The mean and variance of gene activity are measured across nuclei for each embryo as a function of time and space within AP bins (bin sizes of 2.5% and 1.5% egg length in NC13 and NC14, respectively). For each embryo i and each nuclear cycle, we thus obtain an activity surface (kymograph) for the mean $\mu_{i}(x,t)$ and variance $\sigma_{i}^{2}(x,t)$ as a function of AP position x and time t with respect to mitosis. We then introduce new coordinates for each embryo that account for spatial shift Δx, spatial dilatation α, and temporal dilatation β, i.e., $x^{\prime }=\Delta x+\alpha (x-\langle x\rangle )+\langle x\rangle$ and $t^{\prime }=\beta t$, such that ${\tilde{\mu }}_{i}\left(x^{\prime },t^{\prime }\right)=\mu_{i}(x,t)$ and ${\tilde{\sigma }}_{i}^{2}\left(x^{\prime },t^{\prime }\right)=\sigma_{i}^{2}(x,t)$. Next, we define least squares sums based on the shifted and dilated surfaces ${\tilde{\mu }}_{i}(x,t)$ and ${\tilde{\sigma }}_{i}^{2}(x,t)$:

$$\chi_{1}\left(x,t\right)=\sum_{i=1}^{N_{e}}{\left({\tilde{\mu }}_{i}\left(x,t\right)-\langle {\tilde{\mu }}_{i}\left(x,t\right)\rangle \right)}^{2},$$

$$\chi_{2}\left(x,t\right)=\sum_{i=1}^{N_{e}}\;{\left({\tilde{\sigma }}_{i}^{2}\left(x,t\right)-\left\langle {\tilde{\sigma }}_{i}^{2}\left(x,t\right)\right\rangle \right)}^{2},$$

$$\chi \left(\left\{\Delta x_{i},\alpha_{i},\beta_{i}\right\}\right)=\iint \left(\chi_{1}(x,t)+\sqrt{\chi_{2}(x,t)}\right)dxdt,$$

where $N_{e}$ is the total number of embryos for a given gene.

We then minimized $\chi \left(\left\{\Delta x_{i},\alpha_{i},\beta_{i}\right\}\right)$ to learn $\Delta x_{i},\alpha_{i}$ and $\beta_{i}$ for each embryo, under the following constraints $\sum_{i}\;\Delta x_{i}=0$, $\sum_{i}\;log\;\alpha_{i}=0$ and $\sum_{i}\;log\;\beta_{i}=0$. These constraints ensure that on average (over embryos), position and time remain the same, i.e., $\left\langle x_{i}\right\rangle =0$, $\left\langle \alpha_{i}\right\rangle =1$ and $\left\langle \beta_{i}\right\rangle =1$. When learning the new alignment in NC13, no time dilation was needed, and $\beta_{i}=0$, ∀i. The $\beta_{i}$ were only learned for NC14 since the absence of well-defined events for the exact onset of gastrulation made the temporal alignment harder in that case. In the end, the standard deviation (std) for $\left\{\Delta x_{i}\right\}$ is 1.2% (NC13) and 0.8% (NC14) egg length, the std for $\alpha_{i}$ is 8% (NC13) and 6% (NC14), and the std for $\beta_{i}$ is 5% (NC14).

#### B. Embryo-to-embryo variability and pooling

To increase our allele sample size in each condition, we use a pooling strategy, effectively pooling alleles from multiple spatially and temporally aligned embryos (see Section IV A). This is facilitated by the high reproducibility of the *Drosophila* embryos, which intrinsically exhibit low embryo-to-embryo variability.

##### a. Embryo-to-embryo variability.

We characterize embryo-to-embryo variability on the measured transcriptional activity across embryos. As we have done for aligning embryos, we compute the mean and variance of gene activity across nuclei for each embryo as a function of time and space within AP bins (bin sizes of 2.5% and 1.5% egg length in NC13 and NC14, respectively). We thus obtain for each embryo i and each nuclear cycle the mean $\mu_{i}(x,t)$ and variance $\sigma_{i}^{2}(x,t)$ as a function of AP position x and time t with respect to mitosis. We define embryo-to-embryo variability as the variance of the mean $\sigma_{emb}^{2}$ across $N_{e}$ embryos, i.e.,

(1) $$\sigma_{\text{emb}\;}^{2}\left(x,t\right)=\sum_{i=1}^{N_{e}}w_{i}\left(x,t\right){\left(\mu_{i}\left(x,t\right)-\mu \left(x,t\right)\right)}^{2},$$

where the weight are given by $w_{i}(x,\;t)=1/N_{i}(x,\;t)$ with $N_{i}(x,\;t)$ the number of alleles at time t in the AP-bin centered at position x for embryo i, and the global mean $\mu (x,t)=\sum_{i=1}^{N_{e}}\;w_{i}(x,\;t)\mu_{i}(x,\;t)$. Applying the law of total variance, the total variance in the measured transcriptional activity $\sigma^{2}$ is given by

(2) $$\sigma^{2}\left(x,t\right)=\sigma_{\text{emb}}^{2}\left(x,t\right)+\sum_{i=1}^{N_{e}}w_{i}\left(x,t\right)\sigma_{i}^{2}\left(x,t\right),$$

where the last term corresponds to intra-embryo variability, i.e. variability across nuclei (plus a small contribution coming from measurement error, see Section IV D). To determine the contribution of the embryo-to-embryo variability on the measured total variance, we compute the ratio $\sigma_{emb}^{2}(x,\;t)/\sigma^{2}(x,\;t)\leq 1$ for all gap genes in NC13 and NC14. As it has been previously measured in fixed embryos [23], the embryo-to-embryo variability remains small compared to the total variance throughout developmental time, overall averaging to less than 10% of the total variance (see fig. S1F for specific time points).

##### b. Pooling nuclei across embryos.

Having shown that embryo-to-embryo variability represents only a small fraction (~10%) of the total variability, we can treat (to a good approximation) individual embryos as independent samples drawn from the same underlying distribution. Thus, to characterize the fluctuations in transcriptional activity for a given gene, we pool together all the nuclei (at the same time and position along the AP axis) from all measured embryos (aligned in space and time beforehand, see Section IV A) generating a large sample of nuclei (∼200 nuclei). Specifically, once the embryos are spatiotemporally aligned, we define common spatial bins along the AP axis (bin sizes of 2.5% and 1.5% egg length in NC13 and NC14, respectively) allowing us to pool nuclei at same position and at each time point. Pooling provides a large gain in sample size (by a factor $N_{e}=10-20$ embryos), facilitating the estimation of the mean profiles and higher moments of the alleles’ transcriptional activity (Section IV C), as well as the estimation of the bursting parameters (Section V). Moreover, embryo pooling is justified as 1) embryo-to-embryo variability is almost negligible, and 2) we focus explicitly on variability across nuclei, which mostly reflects transcription dynamics and the underlying bursts (provided the measurement noise is small). Hence we assume that for a given AP bin nuclei sample the same distribution function in each embryo.

#### C. Calibration of absolute units in live imaging data

We calibrate our live measurements to absolute units, which are defined as cytoplasmic units (C.U.) and correspond to equivalent single mRNA molecule counts at the site of transcription. The calibration is achieved by matching mean activity profiles in NC13 from live and previously calibrated fixed smFISH measurements [23]. Here we explain this procedure in detail.

##### a. Expected live activity.

First, we use calibrated smFISH measurements of nascent transcriptional activity (in C.U.) for all trunk gap genes in late NC13 (10 – 15 min into NC13) to compute the expected live activity. From these calibrated fixed measurements, we compute mean activity profiles for each gene by pooling the nuclei from multiple embryos (n=10-20) within AP bins of 2.5% egg length (∼2 nuclei wide region along the AP axis) (see Zoller et al. [23], Fig. 1C). The resulting smFISH mean activity profiles ${\overset{‾}{A}}_{F}(x)$ predict the expected levels of live mean activity ${\overset{‾}{A}}_{L}(x)$ (in C.U.) as a function of position x along the AP axis. We compute the expected ${\overset{‾}{A}}_{L}$ by adjusting ${\overset{‾}{A}}_{F}$ for differences in gene copy number ($n_{F}=2$ alleles imaged in fixed vs $n_{L}=1$ allele in live), gene length (endogenous length in fixed versus an additional ∼1.5 kb length due to the insertion of the MS2 cassette in live measurements), and the specificity of targeted locations by FISH probes and stem-loops. We thus get

$${\overset{‾}{A}}_{L}=\frac{n_{L}{\tilde{L}}_{L}}{n_{F}{\tilde{L}}_{F}}{\overset{‾}{A}}_{F},$$

where ${\tilde{L}}_{F}$ and ${\tilde{L}}_{L}$ are effective gene lengths that account for the effect of probe locations on the signal.

##### b. Computing effective lengths.

Second, we compute the effective gene length $\tilde{L}$ for all constructs. Knowing the exact location of the binding sequences for the fluorescent smFISH probes (fixed) and of the MS2 sequences (live) along the gene, we calculate the relative contribution of a single nascent transcript (compared to a fully tagged transcript corresponding to one C.U.) to the signal s(l) as a function of its length l:

(3) $$s\left(l\right)=\frac{1}{m}\sum_{i=1}^{m}H\left(l-l_{i}\right),$$

where H is the (Heaviside) unit step function, $l_{i}$ the end position of the $i^{\text{th}\;}$ probe binding sequences and m the total number of probes. Integrating s(l) over the gene length $L_{g}$ (from TSS to polyA site) leads to the effective length:

(4) $$\tilde{L}=\int_{0}^{L_{g}}s\left(l\right)dl=\frac{1}{m}\sum_{i=1}^{m}\left(L_{g}-l_{i}\right)=L_{g}-\langle l_{i}\rangle .$$

The resulting physical gene length $L_{g}$ and the effective gene length $\tilde{L}$ are given in Table S1. The physical length $L_{g}$ is slightly longer than the annotated gene length, as it accounts for a small retention time (∼30 s) of nascent transcripts at the transcription site, due to termination and possibly Pol II running further than the annotated 3’UTR. We have previously estimated the equivalent quantity of this retention time as an extra transcribed length in dual color smFISH measurements [23].

##### c. Estimating conversion factor for profile matching.

Third, we compute the non-calibrated live mean activity profiles $\overset{‾}{S}(x)$ for all constructs by averaging the single-allele activity time series over all nuclei in a given 2.5% AP bin over a 10–15 min time window in NC13, effectively pooling together nuclei from n=10-20 embryos. The conversion factor ν (allowing us to express S in absolute units) is estimated by defining a pseudo-likelihood function for a given gene g:

$${ℒ}_{g}\left(\overset{‾}{S}|\nu ,\Delta x\right)=\prod_{i=1}^{N_{x}}𝒩\left(\nu \overset{‾}{S}\left(x_{i}\right)|\mu ={\overset{‾}{A}}_{L}\left(x_{i}-\Delta x\right),\sigma^{2}=\sigma_{A}^{2}\left(x_{i}-\Delta x\right)+\nu^{2}\sigma_{S}^{2}\left(x_{i}\right)\right),$$

where 𝒩 is the normal probability density function (i.e. a Gaussian), $N_{x}$ the number of positions along the profiles, and Δx a possible small shift in space registration (typically <1% egg length) between fixed and live experiments. $\sigma_{A}$ and $\sigma_{S}$ are standard errors of the mean based on the smFISH prediction and live averaging, respectively. Typically, ν accounts for the different imaging parameters (such as quantum yield and PMT amplification, etc.) between the fixed (confocal microscopy) and live (two-photon microscopy) experiments. The inference of the factor ν was performed globally over all genes at once, i.e., by maximizing

$$ℒ\left(\left\{{\overset{‾}{S}}_{g}\right\}|\nu ,\left\{\Delta x_{g}\right\}\right)=\prod_{g}{ℒ}_{g}\left({\overset{‾}{S}}_{g}|\nu ,\Delta x_{g}\right),$$

leading to a single ν that was used to define our global calibration unit 1/ν. We also inferred ν per individual gene to assess our calibration error (fig. S1B).

##### d. Comparison of higher cumulants.

To further validate the quantitative nature and the proper calibration of our measurements, we compute the mean-to-higher cumulant relationships and compare them to previously extracted relationships where we used smFISH [23]. The first four cumulants of the measured activity A are defined as

(5) $$\begin{array}{ll} & \kappa_{1}\equiv \mu =\langle A\rangle \\ & \kappa_{2}\equiv \sigma^{2}=\left\langle A^{2}\right\rangle -\langle A\rangle^{2}\\ & \kappa_{3}=\left\langle A^{3}\right\rangle -3\left\langle A^{2}\right\rangle \langle A\rangle +2\langle A\rangle^{3}\\ & \kappa_{4}=\left\langle A^{4}\right\rangle -4\left\langle A^{3}\right\rangle \langle A\rangle -3{\left\langle A^{2}\right\rangle }^{2}+12\left\langle A^{2}\right\rangle \langle A\rangle^{2}-6\langle A\rangle^{4}. \end{array}$$

The cumulants are extensive, meaning that the contributions from independent random variables are additive, which is convenient as the measured transcriptional activities result from the sum of $n_{g}$ independent gene copies. Assuming replicated sister chromatids, $n_{g}=2$ for our live data (only one allele per nucleus has MS2 stem-loops) and $n_{g}=4$ for smFISH data (both alleles in each nucleus are tagged with probes). Using the definition above, we compute the higher cumulants of the calibrated activity from both, smFISH and live measurements. The same pooling strategy as for the means is applied (see previous paragraphs), using 2.5% AP bins over a 10–15 min time window in NC13.

We have previously demonstrated that the higher cumulants can be normalized, assuming Poisson background, such that we account for differences in gene length, probe locations, and gene copy number [23]. We compute the normalized cumulants $\kappa_{k}^{\prime }$ for a single gene copy as follows:

(6) $$\kappa_{k}^{\prime }=\frac{1}{n_{g}C_{k}}{\left(\frac{L^{\prime }}{L_{g}}\right)}^{k}\kappa_{k},$$

where $n_{g}$ is the gene copy number, $L_{g}$ the original gene length, $L^{\prime }$ the normalized gene length. The k-th order coefficients $C_{k}$ are defined as

$$C_{k}=\frac{1}{L_{g}}\int_{0}^{L_{g}}s(l{)}^{k}dl=\sum_{i=1}^{m}{\left(\frac{i}{m}\right)}^{k}\frac{l_{i+1}-l_{i}}{L_{g}},$$

where s(l) is the relative contribution of a single nascent transcript (Eq. 3). Note that for k=1, we have $C_{1}L_{g}=\tilde{L}$, which is the effective gene length.

We compare the normalized cumulants computed from live and smFISH data using a normalized gene length of $L^{\prime }=3.3\;kb$ (which corresponds to the average gap gene length in the absence of stem-loops, also used in Zoller et al. [23]). Once normalized, the first cumulant $\kappa_{1}^{\prime }$ corresponds to the mean number of nascent transcripts ⟨g⟩ on a single gene copy of length $L^{\prime }$. We plot the normalized higher cumulants $\kappa_{2}^{\prime }$, $\kappa_{3}^{\prime }$, and $\kappa_{4}^{\prime }$ as a function of that ⟨g⟩ (fig. S1C). We further normalized all the cumulants by $g_{0}^{k}$. The value $g_{0}$ corresponds to the largest intercept between a k-th order polynomial fit to mean-cumulant relationships (continuous line for live and dotted line for smFISH data) and the straight line of the Poisson background (dashed line) set by $\kappa_{k}^{\prime }=\langle g\rangle$. The number $g_{0}$ provides the maximal possible value of ⟨g⟩ based on the assumption that transcriptional variability $\kappa_{2}^{\prime }$ cannot be lower than Poisson. Thus, $g_{0}$ can be interpreted as the mean number of nascent transcripts (on a 3.3 kb long gene) at maximal activity. We get $g_{0}=13.6$ from live and $g_{0}=15.2$ with smFISH measurements, a difference of 12% based on higher cumulants, not too far from the 5% error obtained from calibrating the means (fig. S1B). Overall, the higher cumulants versus mean relationships obtained from live (fig. S1C left column) and from smFISH (fig. S1C right column) measurements match almost perfectly (black solid versus dotted line), strongly confirming the quantitative nature and the proper calibration of both our live and fixed assay.

#### D. Measurement error

We evaluate imaging error through two distinct approaches: a direct measurement conducted through a specially designed experiment, and an auto-correlation analysis of the recorded transcription time series. The utilization of these two independent methods not only provides a robust internal control but also validates the reliability and effectiveness of our chosen approach. The convergence of results obtained from both approaches strengthens the credibility of our findings.

##### a. Imaging error via direct dual color measurement.

In order to experimentally assess imaging or measurement error, we performed the same experiment twice and simultaneously with two different colors. A discrepancy in both experiments from a perfect match can be assigned to measurement error. We inserted a cassette of interlaced (alternating) MS2 and PP7 stem-loops in the first intron of *Kr*. The MS2 stem-loops were tagged with MCP-GFP (green) and the PP7 stem-loops with PCP-mCherry (red). By plotting the green channel against the red channel (fig. S1D, left), we characterize the spread of the data along the expected line of slope one (when correctly calibrated and without noise both channels are expected to perfectly correlate). We build a simple effective model to describe measurement noise in each channel:

$$P\left(A|G\right)=𝒩\left(A|G,\sigma^{2}\left(G\right)\right),$$

where, for a given channel, the measured transcriptional activity A (in C.U.) is normally distributed with mean G and variance $\sigma^{2}(G)$. G is the corresponding activity (in C.U.) in the absence of noise, and the variance $\sigma^{2}$ explicitly depends on G to account for the heteroscedasticity (i.e., heterogeneity of variance) in the data as the observed spread in the data increases with transcriptional activity.

To estimate the variance $\sigma^{2}$, we fit a straight line y=αx+γ assuming error on both $x\equiv A_{\text{green}}$ and $y\equiv A_{\text{red}}$. We expand the variance as a function of the scalar projection along the line v:

$$\sigma^{2}\left(v\right)=\sigma_{b}^{2}+\beta_{1}v+\beta_{2}v^{2},$$

$$v\;=\;\frac{x\;+\;\alpha (y\;-\;\gamma )}{\sqrt{1\;+\;\alpha^{2}}}.$$

Assuming the same error for green (x) and red (y), we maximize the following likelihood to estimate the set of parameters $\theta =\left\{\alpha ,\gamma ,\sigma_{b},\beta_{1},\beta_{2}\right\}$:

$$ℒ\left(\left\{x_{i},y_{i}\right\}|\theta \right)=\prod_{i=1}^{N_{d}}\frac{1}{\sqrt{2\pi \sigma^{2}\left(v_{i}\right)}}\text{exp}\left(-\frac{{\left(y_{i}\;-\;\alpha x_{i}\;-\;\gamma \right)}^{2}}{2\left(1\;+\;\alpha^{2}\right)\sigma^{2}\left(v_{i}\right)}\right),$$

where $N_{d}$ is the total number of pairs of red-green data points.

Since only the green channel is calibrated in absolute units, we normalize the red channel such that the slope α=1. Using the Akaike information criterion [67], we find that the best model was parameterized by only $\left(\sigma_{b},\beta_{1}\right)$ with α=1, γ=0 and $\beta_{2}=0$. The best fitting parameters are $\sigma_{b}=1.22$ and $\beta_{1}=0.11$. The imaging error derived from the noise measurement model follows:

(7) $$\sigma \left(G\right)=\sqrt{\sigma_{b}^{2}+{\tilde{\beta }}_{1}G},$$

with ${\tilde{\beta }}_{1}=\sqrt{2}\beta_{1}=0.16$. The resulting imaging noise is shown in fig. S1D and S1E. The functional form of the imaging error is exactly what one would expect from laser scanning microscopy, that is a background term $\sigma_{b}^{2}$ and a Poisson shot noise term ${\tilde{\beta }}_{1}G$, the latter being proportional to the activity G.

##### b. Imaging error from correlation-based approach.

Due to the elongation of individual nascent transcripts adding a persistent signal to the measured activity, we expect a strong temporal correlation in the measured single allele activity a(t). Indeed, based on gene length, i.e. Table S1), and elongation rate, i.e. $K_{\text{elo}}=1.8kb/min$, (see Section VA f for validation of deconvolution and estimation of elongation rate), imaged transcripts should persist at the transcription site for at least τ=2min on average. Thus, at a short timescale near our sampling time Δt=10s(Δt≪τ), most biological variability should be correlated in time. Consequently, the remaining uncorrelated variability $\sigma_{u}^{2}$ is likely related to the imaging error as the latter is also expected to be temporally uncorrelated.

We estimate the uncorrelated variability $\sigma_{u}^{2}$ from the transcriptional activity time series A(t) within each AP bin for each gene individually. To this end, we compute the time-dependent mean activity μ(t)=⟨A(t)⟩, variance $\sigma^{2}(t)=\left\langle (A(t)-\mu (t){)}^{2}\right\rangle$ and covariance Cov(t,t+Δt)=⟨(A(t)-μ(t))(A(t+Δt)-μ(t+Δt))⟩, where Δt is the sampling time (and the averaging is performed over all alleles in each AP bin pooled from multiple embryos). For a Δt sufficiently small compared to the correlation time τ imposed by elongation, $\sigma_{u}^{2}(t)=\sigma^{2}(t)-Cov(t,t+\Delta t)$ should be a good estimate of the uncorrelated variability in the data.

When we plot $\sigma_{u}^{2}(t)$ as a function μ(t) for all genes and AP bins across NC13 and NC14 (fig. S1D left), we observe a strong linear relationship between μ(t) and $\sigma_{u}^{2}(t)$. That is, $\sigma_{u}^{2}(t)=\sigma_{b}^{2}+\beta \mu (t)$ with $\sigma_{b}=0.75$ and β=0.19, which is reminiscent of the functional form that we find using the interlaced approach above (Eq. 7). Indeed, we find again that the variance is given by a background $\sigma_{b}^{2}$ and a Poisson shot noise term βμ that is proportional to the mean activity. The interlaced- and the correlation-based quantifications of the imaging error are numerically very close over the entire range of measured activities with a sensitivity close to a single mRNA (fig. S1E).

### V. DATA ANALYSIS AND MODELING

#### A. Deconvolution of the single-allele transcription rate

##### a. Modeling initiation events.

We designed a simple model to reconstruct the individual initiation events of productive transcription by Pol II from the calibrated single allele transcriptional activity A(t) (in C.U.). Our model for measurement noise σ(G) (Eq. 7) links the activity A(t) to the signal in the absence of measurement noise G(t) by:

(8) $$A\left(t\right)=G\left(t\right)+\sigma \left(G\left(t\right)\right)\eta \left(t\right),$$

where η(t) is an uncorrelated Gaussian white noise with zero mean and unit standard deviation. The signal G(t) is expressed as a convolution between a kernel κ(t) (modeling the fluorescent signal resulting from the Pol II elongation process through the stem-loop cassette) and a function I(t) that models Pol II initiation events:

(9) $$G\left(t\right)=\kappa \left(t\right)*I\left(t\right).$$

The initiation function I(t) can be expressed as

(10) $$I\left(t\right)=\sum_{i=1}\delta \left(t-t_{i}\right),$$

where δ are unit pulses (δ-Dirac) representing to individual initiation events at time $t_{i}$. The kernel function κ(t) is built according to the signal contribution of individual transcripts (see Eq. 3):

(11) $$\kappa \left(t\right)=\frac{1\;-\;H\left(t\;-\;\frac{L_{g}}{K_{\text{elo}}}\right)}{m}\sum_{i=1}^{m}H\left(t-\frac{l_{i}}{K_{\text{elo}}}\right),$$

where H is the unit (Heaviside) step function, $L_{g}$ the physical length of the gene, $l_{i}$ the end position of the *i^th^* MS2 stem-loop sequence and m the total number of loops (in our construct m=24). The key assumptions behind this kernel are i) constant elongation rate $K_{\text{elo}}$, ii) deterministic elongation (limited fluctuations, no pausing in the gene body), iii) fast termination, and iv) no co-transcriptional splicing (with short gene lengths, facilitating fast transcription). Importantly, all the parameters in Eq. 11 are determined by the DNA sequence, except the elongation rate $K_{\text{elo}}$ that needs to be inferred from measurements.

##### b. Single allele Bayesian deconvolution of transcription initiation events.

We performed Bayesian deconvolution to reconstruct the possible configuration of initiation events I from individual single allele activity time series $A=\left(A_{1},A_{2},\ldots ,A_{N_{t}}\right)$, where $A_{i}\equiv A\left(t_{i}\right)$, $t_{i+1}-t_{i}=\Delta t\approx 10\text{s}$ ∀i and $N_{t}$ the number of time points. We first wrote the likelihood of the measured activity time series A given the true signal time series G (discretely sampled at the same time points as A):

(12) $$P\left(A|G\right)=\prod_{i=1}^{N_{t}}𝒩\left(A_{i}|G_{i},\sigma^{2}\left(G_{i}\right)\right).$$

Individual time points, $A_{i}$, are assumed to be normally distributed with mean $G_{i}$ and variance $\sigma^{2}\left(G_{i}\right)$ given by our measurement noise model (see Eq. 7).

According to our model above (Eq. 9), the signal G results from the convolution of κ*I. To compute this convolution, we first discretize the time variable t in the kernel κ(t) and the initiation function I(t) (see Eq. 10 and 11) using a smaller sampling time $\Delta t^{\prime }=f/\left(2K_{\text{elo}}\right)=1\;s$, where f=60bp is a conservative estimate on the Pol II footprint on the DNA, $K_{\text{elo}}=1.8\;kb/min$ is the measured elongation rate (see Section V A 0 f; Validation of deconvolution and estimation of elongation rate) and the factor 2 accounts for having two unresolved active sister chromatids. $\Delta t^{\prime }=1\;s$ is the minimal physically possible time interval between two consecutive Pol II loading events (oversampling beyond $\Delta t^{\prime }$ is superfluous). When discretized, the initiation configuration I becomes a binary vector $I=\left(I_{1},I_{2},\ldots ,I_{N_{I}}\right)$ with $I_{i}\equiv I\left(t_{i}^{\prime }\right)\in \{0,1\},t_{i+1}^{\prime }-t_{i}^{\prime }=\Delta t^{\prime }\forall i$ and $N_{I}=\left\lceil N_{t}\Delta t/\Delta t^{\prime }\right\rceil -1$. Thus G can be computed as a discrete convolution:

$$G_{i}=\sum_{j=1}^{min(k,i)}I_{i-j+1}\kappa_{j},$$

where $k=\left\lceil L_{g}/\left(K_{\text{elo}}\Delta t^{\prime }\right)\right\rceil +1$ is the number of time points needed to discretize the kernel κ and $L_{g}$ the physical length of the gene (see Table S1). The resulting time series G is then downsampled to evaluate P(A∣G) (Eq. 12) at the same time points as the measured activity time series A. We thus have effectively designed a simple procedure to evaluate P(A∣I), i.e., the likelihood of A given an initiation configuration I:

(13) $$P\left(A|I\right)=\sum_{G}P\left(A|G\right)P\left(G|I\right),$$

where P(G∣I)=1 if G=κ*I and P(G∣I)=0 otherwise.

Applying Bayes’ theorem gives us the posterior distribution P(I∣A), i.e., the distribution of possible configurations of initiation events I given a measured single allele activity time series A:

(14) $$P\left(I|A\right)=\frac{P\left(A|I\right)P\left(I\right)}{\sum_{I}\;P\left(A|I\right)P\left(I\right)},$$

where P(I) is the prior distribution on the configuration of initiation events (see Section V A 0 c; Setting the prior). The posterior P(I∣A) is our Bayesian representation of the deconvolved initiation configurations I and naturally provides the uncertainty on these configurations as a probability distribution. Importantly, P(I∣A) accounts for measurement noise and incorporates our assumptions about the elongation process underlying the fluorescent signal. Overall, the Bayesian approach to our deconvolution problem has a few advantages over maximum likelihood estimation (MLE). First, in the presence of measurement noise, there could be a risk with MLE that the unique resulting configuration I (the one that maximizes P(I∣A)) overfits the noise. In contrast, Bayesian estimation readily provides all the possible configurations compatible with measurement noise. Second, when the transcriptional activity is high and many Pol II/ transcripts contribute to the measured activity A, the underlying initiation configurations I may be degenerate (meaning that multiple I once convolved with the kernel κ would lead to the same or very close A. In that context, choosing a single configuration seems arbitrary, as MLE would do, whereas Bayesian estimation naturally captures the symmetries underlying the initiation configurations.

##### c. Setting the prior.

A prior P(I) over the initiation configurations needs to be defined in order to calculate the posterior distribution P(I∣A) in Eq. 14. As $I_{i}\in \{0,\;1\}$, expressing the prior P(I) as a product of multiple independent Bernoulli distributions represents a simple and natural solution:

(15) $$P\left(I\right)=\prod_{i=1}^{N_{I}}p_{i}^{I_{i}}{\left(1-p_{i}\right)}^{1-I_{i}},$$

with the parameters $p_{i}\in [0,\;1]$ for each time point. For a given gene and AP bin, we set these parameters to $p_{i}=R\left(t_{i}\right)\Delta t^{\prime }$, where R(t) is the time-dependent mean transcription rate in the AP bin. A good proxy for R(t) is given by $R(t)\approx \mu \left(t+\tau_{\text{elo}}/2\right)/\tau_{\text{elo}}$, where μ(t) is the mean activity in the AP bin and $\tau_{\text{elo}}$ the effective elongation time of the gene given by $\tilde{L}/K_{\text{elo}}$ (see Eq. 23 and Table S1). Thus, our prior mainly encapsulates our knowledge about the mean transcription rate and not much about the underlying single allele initiation configurations. Interestingly, a constant prior P(I)=cst. implies that $p_{i}=0.5\;\forall i$, which is equivalent to a constant mean transcription rate set at $R_{max}/2$ with $R_{max}=1/\Delta t^{\prime }=60\;mRNA/min$ the maximal physical rate given the Pol II footprint and the measured elongation rate. However, in practice, the prior only has a small impact on inferred initiation configurations as the data is strong, i.e., given our imaging sensitivity and a rather short elongation time the likelihood P(A∣I) strongly constrains the possible configurations I allowed by the prior.

##### d. MCMC sampling of initiation configurations.

We designed a Monte-Carlo Markov Chain (MCMC) algorithm [68], to directly sample from the posterior distribution P(I∣A) in Eq. 14. This method relies on building a Markov Chain $P\left(I^{\prime }|I\right)$, whose stationary distribution π(I) is the target distribution, i.e., π(I)≡P(I∣A). Such a Markov chain is obtained by imposing detailed balance $P\left(I^{\prime }|I\right)\pi (I)=P\left(I|I^{\prime }\right)\pi \left(I^{\prime }\right)$, which ensures that the chain has the correct stationary distribution. Once the chain has asymptotically reached after a few iterations the stationary distribution, samples drawn from $P\left(I^{\prime }|I\right)$ at each subsequent iteration are equivalent to samples directly drawn from $P\left(I^{\prime }|A\right)$ (where $I^{\prime }$ is the new sample given the previous one I).

In practice, the sampling procedure at each iteration is performed in two steps. First, a new sample $I^{\prime }$ is drawn from a proposal distribution $Q\left(I^{\prime }|I\right)$. Second, the proposed sample $I^{\prime }$ is accepted $\left(I^{\prime }\to I\right)$ or rejected (I→I), with acceptance probability $\alpha \left(I^{\prime },I\right)$:

(16) $$\alpha \left(I,I^{\prime }\right)=min\left(1,\frac{P\left(A|I^{\prime }\right)P\left(I^{\prime }\right)Q\left(I,I^{\prime }\right)}{P(A|I)P(I)Q\left(I^{\prime },I\right)}\right),$$

where P(A∣I) is the likelihood (Eq. 13) and P(I) is the prior (Eq. 15). The proposal distribution $Q\left(I^{\prime }|I\right)$ must be carefully chosen to ensure a good acceptance probability (a good acceptance is typically between 10–70%) [69, 70]. Indeed, if the acceptance is too high ($I^{\prime }$ is too close to I) or too low ($I^{\prime }$ is too far from I), the chain will generate highly correlated samples (poor mixing), and the sampling will be inefficient (slow convergence). In principle, a good proposal is a distribution that closely mimics the target distribution. Since the target distribution is a priori unknown, we use an adaptative method to learn $Q\left(I^{\prime }|I\right)$ on the fly such that it approaches the target distribution [69, 70]. In that case, the proposal distribution can typically be chosen as independent from the previous sample $Q\left(I^{\prime }|I\right)=Q\left(I^{\prime }\right)$, and our prior (Eq. 15) represents a decent candidate proposal distribution:

(17) $$Q\left(I\right)=\prod_{i=1}^{N_{I}}p_{i}^{I_{i}}{\left(1-p_{i}\right)}^{1-I_{i}}$$

Here, the goal is to learn the parameters $q_{i}\in [0,\;1]$ while sampling the configurations I. As initial values for the $q_{i}$, we chose $q_{i}=\tilde{A}\left(t_{i}^{\prime }\right)\Delta t^{\prime }$, where $\tilde{A}(t)=A\left(t+\tau_{\text{elo}}/2\right)/\tau_{\text{elo}}$ with A(t) the activity time series and $\tau_{\text{elo}}$ the elongation time. After each iteration of the MCMC sampler (after the acceptance or rejection of the proposed $I^{\prime }$), we then update the $q_{i}$ according to the following adaptative scheme:

$$q_{i}^{\prime }=q_{i}\left(1-l\right)+lI_{i},$$

where $q_{i}^{\prime }$ is the new $q_{i}$, I the current configuration, and the learning rate $l=1/\sqrt{3j}$ with j the iteration number. The function l, which vanishes when j→∞, is chosen such that the Markov chain converges despite adaptation. Importantly, we enforce $q_{i}>0.05\;\forall i$ at each iteration to ensure that all time points of configuration I are sampled.

In practice, even with adaptation, attempting to update the whole configuration I at once according to the proposal (Eq. 17) can lead to sub-optimal acceptance. Indeed, the $I_{i}$ ‘s are often quite correlated, whereas our proposal assumes independent $I_{i}$. A simple workaround is to update the $I_{i}$ ‘s by block and increase the number of iterations: only the $I_{i}$ from a subset $i\in \{b,\ldots ,b+w-1\}\subset \left\{1,\ldots ,N_{I}\right\}$ are drawn from the proposal (Eq. 17) at each iteration (while the remaining $I_{i}$ are left unchanged), with b a random integer and w the size of the block. The size w is then tuned to achieve the desired acceptance ratio. We found that block size w covering approximately 6 min led to a good compromise between a decent acceptance ratio and sampling speed. To sample I, we performed $N_{s}N_{I}/w$ iterations with $N_{s}=3500$ and we discarded the first $N_{b}N_{I}/w$ iteration, where $N_{b}=500$.

##### e. Estimating single allele transcription rate.

Once the posterior distribution P(I∣A) of possible initiation configuration has been sampled by MCMC, we compute the single-cell transcription rate r. For each configuration I (Eq. 10 and 13), we can compute a single r(t) as follows:

(18) $$r\left(t\right)=\frac{1}{v\Delta t}\int_{t-v\Delta t}^{t}I\left(t^{\prime }\right)dt^{\prime }=\frac{1}{v\Delta t}g\left(t\right),$$

where g(t) is the number of initiation events during the time interval t∈[t-vΔt,t] given by

$$g\left(t\right)=\sum_{i\in \left\{i:t-v\Delta t\leq t_{i}^{\prime }\leq t\right\}}I_{i}.$$

In the equations above, Δt≈10s is our sampling time, and v is a positive integer defining the time window over which r is estimated. Except when explicitly specified otherwise, we estimated r using v=1 which we defined as the instantaneous single allele transcription rate.

Using our MCMC samples, P(r∣A) is easily estimated by

(19) $$P\left(r|A\right)=\sum_{I}P\left(r|I\right)P\left(I|A\right),$$

where P(r∣I)=1 if Eq. 18 is satisfied, and P(r∣I)=0 otherwise. The distribution P(r∣A) enables the computation of the instantaneous mean and standard deviation of r(t) for individual single-allele activity time series A (fig. S2B). Of note, P(r∣A) is actually a discrete probability distribution since we have a finite amount of MCMC samples and r is discrete (as is g).

##### f. Validation of deconvolution and estimation of elongation rate.

To validate the hypothesis behind our kernel-based deconvolution (see Section VA 0 a; Modeling initiation events), we tested our approach on dual color data, where the MS2 and PP7 stem-loop cassettes were inserted in the first intron and 3’UTR of *hb*, respectively. Similarly, our approach was tested on a data set for an additional gene, *Kr*, where the stem-loop insertions were permuted (PP7 in intron and MS2 in 3’UTR). In both cases MS2 stem-loops were tagged with MCP-GFP (green) and PP7 stem-loop via PCP-mCherry (red).

After calibrating the green and red channels in absolute units (see Section IV C), we expressed the likelihood of obtaining the green $A_{g}$ and the red activity $A_{r}$ together, which is simply $P\left(A_{g}|G_{g}\right)P\left(A_{r}|G_{r}\right)$, where $G_{g}$ and $G_{r}$ are the true signals in each channel. Both $P\left(A_{g}|G_{g}\right)$ and $P\left(A_{r}|G_{r}\right)$ are given by Eq. 12, with $\sigma^{2}(G)$ that is specific to each channel (see Section IV D). In principle, the true signals $G_{g}$ and $G_{r}$ should be highly correlated since they should result from the same initiation configuration I. $G_{g}=\kappa_{g}\;*\;I$ and $G_{r}=\kappa_{r}\;*\;I$ contain two kernels $\kappa_{g}$ and $\kappa_{r}$ that are determined from Eq. 11. Whereas these kernels mainly differ in the location of the stem-loop insertions, they share one free parameter, i.e., the elongation rate $K_{\text{elo}}$ that is a priori unknown. Importantly, the overall delay between $G_{g}$ and $G_{r}$ should be given by the relative position $\left|l_{g}-l_{r}\right|$ of the MS2 and PP7 stem-loop cassettes divided by $K_{\text{elo}}$. It implies that $K_{\text{elo}}$ can be extracted from these dual-color data. With these ingredients, the likelihood function for the pair $A_{g}$ and $A_{r}$ is as follows:

(20) $$P\left(A_{\text{g}},A_{\text{r}}|I,K_{\text{elo}\;}\right)=\sum_{G_{\text{g}},G_{\text{r}}}P\left(A_{\text{g}}|G_{\text{g}}\right)P\left(A_{\text{r}}|G_{\text{r}}\right)P\left(G_{\text{g}},G_{\text{r}}|I,K_{\text{elo}\;}\right),$$

where $P\left(G_{g},G_{r}|I,K_{\text{elo}}\right)$ is trivially determined from $G_{g}=\kappa_{g}\left(K_{elo}\right)\;*\;I$ and $G_{r}=\kappa_{r}\left(K_{\text{elo}\;}\right)\;*\;I$ (see Eq. 13).

Next, from our dual-color data, we inferred I and $K_{\text{elo}}$ for each nucleus using the likelihood in Eq. 20. Specifically, we sampled from the posterior distribution $P\left(I,K_{\text{elo}}|A_{g},A_{r}\right)$ according to our MCMC sampler (see Section V A 0 d; MCMC sampling of initiation configurations). We set the prior of $K_{\text{elo}}$ to log-uniform $P\left(K_{\text{elo}}\right)∼1/K_{\text{elo}}$. For both *hb* and *Kr* data, we assessed the quality of the deconvolution by computing the normalized residuals for each channel c∈{g,r} and each nucleus:

$$z_{c}=\frac{A_{c}\;-\;{\overset{‾}{G}}_{c}}{\sigma_{c}\left({\overset{‾}{G}}_{c}\right)},$$

where $\sigma_{c}(G)$ is the measurement error of channel c, and ${\overset{‾}{G}}_{c}$ the mean reconstructed signal computed as the marginal mean of

$$P\left(G_{g},G_{r}|A_{g},A_{r}\right)=\sum_{I}\int P\left(G_{g},G_{r}|I,K_{elo}\right)P\left(I,K_{elo}|A_{g},A_{r}\right)dK_{elo}.$$

Since the residuals $z_{c}$ are given by a time series, we thus reported the mean $\mu \left(z_{c}\right)$ and the standard deviation of $\sigma \left(z_{c}\right)$ in the two channels for each nucleus (fig. S2E). We then compared the $\mu \left(z_{c}\right)$ and $\sigma \left(z_{c}\right)$ to a perfect model for which the $z_{c}$ have the same length than our data and each $z_{c}(t)$ is independent and normally distributed with μ=0 and σ=1. We computed the 95% confidence ellipse of $\mu \left(z_{c}\right)$ and $\sigma \left(z_{c}\right)$ for both our data (solid black line in fig. S2E) and the perfect model (dotted black line in fig. S2E). Our reconstructions are close to the perfect model, meaning that the two channels can be reconstructed from the same initiation configurations within confidence intervals, overall justifying our kernel assumptions. Indeed, the good agreement between the predicted and the measured dual color signal implies that the elongation process must be close to deterministic (with approximately constant elongation rate) and co-transcriptional splicing must be limited for these short genes. Otherwise, we would have observed a striking mismatch between the model prediction and the measurements in the 5’-to-3’ amplitude ratio and the 5’-to-3’ delay.

Lastly, we estimate the elongation rate $K_{\text{elo}}$ for each individual nucleus numerically as the mean of the marginal posterior distribution $P\left(K_{\text{elo}}|A_{g},A_{r}\right)$. Within each embryo and for both *hb* and *Kr*, the average $K_{\text{elo}}$ across nuclei at different AP bins remains stable along the AP axis (fig. S2F). We estimated the global mean $K_{\text{elo}}$ by averaging all nuclei for each embryo, the resulting averages are very similar across genes and embryos (fig. S2G). The overall mean elongation rate is ${\overset{‾}{K}}_{\text{elo}}=1.8\pm 0.1\;kb/min$.

#### B. Gap gene mean expression pattern

##### a. Transcription pattern.

From our deconvolution-based estimates of the single-allele transcription rate r(t), we reconstruct the mean expression pattern as a further test for the validity of our approach. For each gene, we align the imaged embryos in space and time, allowing us to pool nuclei from different embryos (see Section IV B; Embryo-to-embryo variability and pooling). Thus, for a given gene and nuclear cycle, we obtain a set 𝒜(x,t) of activity time series A∈𝒜(x,t) coming from all the nuclei in the same AP bin at time t (of width 2.5% and 1.5% egg length in NC13 and NC14, respectively) centered at position x. The pooling procedure results in the distribution of single-allele transcription rate P(r∣x,t) defined as the following mixture:

(21) $$P\left(r|x,t\right)=\sum_{A\in 𝒜\left(x,t\right)}P\left(r\left(t\right)|A\right)P\left(A\right),$$

where P(r∣A) is the distribution of deconvolved r given a single-allele time series A (defined in Eq. 19) and $P(A)=1/N_{A}(x,t)$ with $N_{A}(x,t)=\#𝒜(x,t)∼200$ the total number of nuclei in the AP bin at position x and time t. From P(r∣x,t), we compute the time-dependent mean transcription rate R(x,t) at position x:

(22) $$R\left(x,t\right)=\langle r\left(x,t\right)\rangle =\sum_{r}rP\left(r|x,t\right).$$

Using R(x,t), we reconstruct the mean profiles for all genes as a function of time t, which are displayed for three different time points in fig. S3A (color dots).

To validate our estimation of the mean transcription rate R(x,t), we compare it to an alternative approximation that is independent of the deconvolution procedure:

(23) $$R(x,t)\approx \frac{\mu \left(x,t\;+\;\tau_{elo}/2\right)}{\tau_{\text{elo}\;}},$$

where μ(x,t) is the time-dependent mean activity in the AP bin centered at position x and $\tau_{\text{elo}}$ the effective elongation time of the gene given by $\tilde{L}/K_{\text{elo}}$ (see Table S1). We see in fig. S3A that both approaches (color and black dashed line for deconvolution approach and approximation via Eq. 23, respectively) strongly agree and can thus be used interchangeably to estimate the mean transcription rate R(x,t).

##### b. Transcription and protein pattern comparison.

We further tested whether our mean gap gene patterns measured directly from the transcriptional output, i.e., MS2-signal (fig. S4A, left column) are consistent with previously measured protein patterns (fig. S4A, right column) [31]. To this end, we extrapolate protein accumulation from the characterized transcriptional mean output using a minimal model (fig. S4A, middle column).

We model the accumulation of protein as a convolution between the mean transcription rate R(x,t) and a kernel κ(x,t) that accounts for diffusion, degradation, and a delay in protein production:

(24) $$\kappa (x,t)=\left\{\begin{array}{cc} exp\left(-\frac{t\;-\;\delta }{\tau_{m}}\right)exp\left(-\frac{x^{2}}{4D(t\;-\;\delta )}\right) & \text{if}\;t-\delta \geq 0\\ 0 & \text{otherwise} \end{array}\right.$$

Thus κ(x,t) above is parameterized by three free parameters: a diffusion constant D, a mean lifetime $\tau_{m}$, and a delay δ. We compute the predicted protein pattern $\tilde{P}(x,t)$ as bidimensional convolution (along x and t, hence ** symbol) $\tilde{P}(x,t)=R(x,t)**\kappa (x,t)$, where R(x,t) is estimated using Eq. 23. During NC14, we multiply R(x,t) by a factor of two to account for the doubling of nuclei during mitosis 13. In addition, the resulting $\tilde{P}(x,t)$ is normalized such that its maximum value is one to facilitate direct comparison with previously similarly normalized protein data [31].

To estimate the three kernel parameters $\left\{D,\tau_{m},\delta \right\}$ (Eq. 24), we minimize the squared difference between the predicted protein pattern $\tilde{P}(x,t)$ and the measured protein pattern P(x,t). We performed the optimization for all gap genes individually and together (fig. S4B). The resulting parameters are in line with previous estimates and the modeled accumulation is consistent with previously measured protein patterns (fig. S4A). Given our minimal model, the agreement between the modeled and measured protein patterns suggests that protein levels are mainly determined by transcription.

#### C. Transcription fluctuation analysis

##### a. Distributions of single-allele transcription rates.

To characterize the transcriptional fluctuations around the mean transcription rate R(x,t), we aimed to reconstruct the conditional distribution P(r∣R) of single-allele transcription rate r for each gene. In principle, P(r∣R) should exhibit key signatures of the transcriptional regime at play. Importantly, to magnify these signatures, here we specifically estimate the single-allele transcription rate r over a τ=1-min time window (v=6, in Eq. 18). Indeed, with limited time-averaging, i.e., r estimated over Δt=10s, the distribution P(r∣R) should approach the Poisson distribution at all R.

For each gene and nuclear cycle, our deconvolution method enables estimating the distribution of single-allele transcription rate P(r∣x,t) by pooling nuclei at position x and time point t (see Eq. 21). The mean transcription rate is then given by $R(x,t)=\langle r(x,t)\rangle =\sum_{r}\;rP(r|x,t)$ (Eq. 22).

We reconstruct the conditional distribution P(r∣R) by pooling together all nuclei from the distributions P(r∣x,t) at all position x and time t that satisfy a specific range of means $\left|R(x,t)-R_{0}\right|\leq \epsilon$ (where ϵ is a positive parameter). By defining the set $𝒱\left(R_{0}\right)=\left\{\left(x,t\right)\;:\left|R(x,t)-R_{0}\right|\leq \epsilon \right\}$, we express the conditional as a mixture of the single-allele transcription rate distributions P(r∣x,t):

(25) $$P\left(r|R_{0}\right)\;=\sum_{\left(x,t\right)\in 𝒱\left(R_{0}\right)}P\left(r|x,t\right)P\left(x,t\right)$$

$$P\left(x,t\right)=\frac{N_{A}\left(x,t\right)}{\sum_{\left(x,t\right)\in 𝒱\left(R_{0}\right)}\;N_{A}\left(x,t\right)},$$

where $N_{A}(x,\;t)$ is the number of nuclei in the bin at position x and time t. Importantly, a reconstruction of $P\left(r|R_{0}\right)$ through pooling is only valid under the condition that the distributions P(r∣x,t) for which $(x,\;t)\in 𝒱\left(R_{0}\right)$ are mostly similar.

To verify the validity of our reconstruction of P(r∣R) for each gene, we binned the range of observed R values in 15 equal bins from 0 to 16 mRNA/min. Each respective $R_{0}$ is thus defined as the centers of these R-bins, which sets ϵ=0.53 (the half width of the R bins). We then computed the overall P(r∣R) by applying Eq. 25 for each $R_{0}$. Given $P\left(r|R_{0}\right)$, we determined the 95% confidence envelope on its cumulative distribution. We assessed what fraction of the individual P(r∣x,t) (with $\left.(x,t)\in 𝒱\left(R_{0}\right)\right)$ are within the global 95% envelope. It turns out that when pooling alleles irrespective of developmental time, we observe a significant fraction of (x,t)-bins (position and time point) in NC14 for which P(r∣x,t) significantly deviates from the global distribution (fig. S3B, bottom right). When performing the same operation on three separate periods - NC13 (t≥6.5min after mitosis) plus early NC14 (7.5≤t<20.5min), mid-NC14 (20.5≤t<34.5min) and late NC14 (34.5≤t<48min) – the agreement between the individual P(r∣x,t) and the overall distribution P(r∣R) improves significantly (fig. S3B). This suggests that transcription might be undergoing slow kinetic changes over developmental time, warranting a partitioning of NC14 into broad periods that are roughly stationary.

Having checked the pooling validity underlying the reconstruction of P(r∣R), we then analyze the single-allele transcription rates underlying the period encompassing NC13 and early NC14 (7.5≤t<20.5min). Within this period and for each of the $15{\;R}_{0}$ values defined as the center of the R-bin, we reconstruct the conditional P(r∣R) distribution according to Eq. 25. We show the resulting distributions for each gene in fig. S3C. Strikingly, the conditional P(r∣R) at a given $R=R_{0}$ value looks very similar across genes and periods (NC13 vs early NC14). Moreover, these distributions differ from the typical distribution one would expect in a constitutive (i.e., non-bursting) regime.

##### b. Constitutive regime.

In a constitutive regime, initiation events are assumed to happen as a simple Poisson process at a constant rate of R. Thus over some time τ, we would expect to observe g initiation events with probability given by the Poisson distribution:

$$P_{P}\left(g|\lambda =R\tau \right)=\frac{\lambda^{g}\exp\left(-\lambda \right)}{g!}.$$

Given our definition of the single-allele transcription rate (see Eq. 18), we have r=g/τ implying that in a constitutive regime $P(r|R)=P_{P}(g=r\tau |\lambda =R\tau )$. Such a description of the constitutive regime implicitly assumes that Pol II has no footprint, meaning that initiation could happen in very quick succession. However, this is unrealistic as the Pol II footprint f=60bp (conservative estimate) should set a refractory period $\Delta t^{\prime }=f/\left(2K_{\text{elo}}\right)=1s$ below which successive initiations cannot occur (see Section V A 0 b; Single allele Bayesian deconvolution of transcription initiation). As a simple correction to account for such a refractory period, we discretize the period $\tau >\Delta t^{\prime }$ using the interval $\Delta t^{\prime }$ as we did for our deconvolution procedure. In that case, the probability to observe g initiation events over some time τ would be given by the Binomial distribution:

(26) PBg∣nt=τ/Δt′,p=RΔt′=ntgpg(1-p)nt-g.

Thus, considering the Pol II footprint, the distribution of single-allele transcription rate in a constitutive regime should be well approximated by $P(r|R)=P_{B}\left(g=r\tau |n_{t}=\left\lceil \tau /\Delta t^{\prime }\right\rceil ,p=R\Delta t^{\prime }\right)$. Of note, when $\tau \gg \Delta t^{\prime }\left(\Delta t^{\prime }\to 0\right)$, we recover the Poisson distribution and $P(r|R)=P_{P}(g=r\tau |\lambda =R\tau )$. This means that the binomial correction to the Poisson regime only matters when $\Delta t^{\prime }≲\tau$ and it becomes negligible when $\Delta t^{\prime }\ll \tau$.

To highlight the difference between the empirically determined conditional distributions P(r∣R) and the constitutive “Poisson” regime, we plotted $P_{B}\left(g=r\tau |n_{t}=\left\lceil \tau /\Delta t^{\prime }\right\rceil ,p=R\Delta t^{\prime }\right)$ in fig. S3C (dashed line), where we used =1min. We also computed the 2^nd^ to 4^th^ cumulants from the empirically determined conditional distributions P(r∣R) and the constitutive “Poisson” regime as a function of R. The cumulants are defined as follows:

$$\sigma^{2}(R)\;=\left\langle r^{2}\right\rangle -\langle r\rangle^{2}\;\kappa_{3}(R)\;=\left\langle r^{3}\right\rangle -3\left\langle r^{2}\right\rangle \langle r\rangle +2\langle r\rangle^{3}\;\kappa_{4}(R)\;=\left\langle r^{4}\right\rangle -4\left\langle r^{3}\right\rangle \langle r\rangle -3{\left\langle r^{2}\right\rangle }^{2}+12\left\langle r^{2}\right\rangle \langle r\rangle^{2}-6\langle r\rangle^{4}$$

where $\left\langle r^{k}\right\rangle =\sum_{r}^{k}\;P(r|R)$. Assuming a constitutive regime (Eq. 26), we get the following cumulants:

$$\sigma^{2}(R)\;=n_{t}p(1-p)/\tau^{2}=R\left(1-R\Delta t^{\prime }\right)/\tau \;\kappa_{3}(R)\;=n_{t}p(1-p)(1-2p)/\tau^{3}=R\left(1-R\Delta t^{\prime }\right)\left(1-2R\Delta t^{\prime }\right)/\tau^{2}\;\kappa_{4}(R)\;=n_{t}p(1-p)(1-6p(1-p))/\tau^{4}=R\left(1-R\Delta t^{\prime }\right)\left(1-6R\Delta t^{\prime }\left(1-R\Delta t^{\prime }\right)\right)/\tau^{3}$$

We plot the cumulants from the empirically determined conditional distributions P(r∣R) (color) and the constitutive “Poisson” regime (black dashed line) as a function of R in fig. S3D. Overall, the empirical cumulants deviate significantly from the “Poisson” regime, except at the extreme ends of the R spectrum where we observe a convergence towards the constitutive regime. This can be interpreted as evidence for a bursting regime spanning all the way from $P_{ON}=0$ to $P_{ON}=1$ [23]. Errors on R and the cumulants were computed from bootstrapping.

##### c. Transcription rate auto-correlation.

To characterize the transcriptional fluctuations over time, we compute the auto-correlation of the instantaneous single-allele transcription rate r(t)(v=1, in Eq. 18). For a given gene, nuclear cycle, and for each AP-bin (centered at position x), pooling gives a set 𝒜(x) of activity time series A∈𝒜(x) for all nuclei in that particular AP bin. Our Bayesian deconvolution approach enables sampling from the posterior distribution P(r∣A). It generates MCMC samples of the instantaneous single-allele transcription rate given an activity time series A (see Eq. 19). We denote such a sample $r_{s}(t)|A$, where s labels a specific MCMC sample drawn from P(r∣A). Thus, for a given AP-bin (centered at position x), we compute the normalized auto-correlation function $\rho_{s}(\tau )$ as follows:

(27) $$\rho_{s}\left(\tau \right)=\frac{\left\langle \left(r_{s}\left(t\right)|A\;-\;\mu_{s}\left(t\right)\right)\left(r_{s}\left(t\;+\;\tau \right)\left|A\;-\;\mu_{s}\left(t+\tau \right)\right\rangle \right.\right.}{\sigma_{s}\left(t\right)\sigma_{s}\left(t\;+\;\tau \right)},$$

where τ is the lag (which is a multiple of Δt) and the averaging is performed over all the nuclei in the AP-bin, i.e., $\left\langle F\left(r_{s}(t)|A\right)\right\rangle =\frac{1}{N_{A}(x)}\sum_{A\in 𝒜(x)}\;F\left(r_{s}(t)|A\right)$ with $N_{A}(x)=\#𝒜(x,t)∼200$ nuclei. Hence, $\mu_{s}(t)=\left\langle r_{s}(t)|A\right\rangle$ is the time-dependent mean and $\sigma_{s}(t)=\sqrt{\left\langle {\left(r_{s}(t)|A-\mu (t)\right)}^{2}\right\rangle }$ the time-dependent standard deviation in the AP-bin. The final estimate of the normalized auto-correlation ρ(τ) and the corresponding error $\sigma_{\rho }(\tau )$ are obtained by averaging over MCMC samples:

(28) $$\rho \left(\tau \right)\;=\frac{1}{N_{s}}\sum_{s=1}^{N_{s}}\rho_{s}\left(\tau \right)\;\sigma_{\rho }^{2}\left(\tau \right)\;=\frac{1}{N_{s}}\sum_{s=1}^{N_{s}}{\left(\rho_{s}\left(\tau \right)-\rho \left(\tau \right)\right)}^{2},$$

where $N_{s}=10^{3}$ is the total number of samples used per allele.

We show auto-correlation functions ρ(τ) for *hb* in early NC14 (7.5≤t<20.5 min after mitosis) in Fig. 1E (color code stands for the AP-bin). An auto-correlation is characterized by two key quantities: the magnitude of correlated fluctuations $\Sigma_{AC}\;:=\rho (\Delta t)$ (where Δt=10s is the sampling time) and the correlation time $\tau_{AC}$ that captures the temporal scale of the fluctuations. We estimated $\Sigma_{AC}$ and $\tau_{AC}$ for all gap genes at all AP positions in NC13 and early NC14 (fig. S5C and Fig. 1F). The correlation time $\tau_{\text{AC}}$ was estimated by fitting ρ(τ) with the following exponential function for Δt≤τ<10min:

(29) $$\tilde{\rho }\left(\tau \right)=\Sigma_{AC}\left(1-\beta \right)\exp\left(-\frac{\tau \;-\;\Delta t}{\tau_{AC}}\right)+\beta ,$$

where $\Sigma_{AC}$ is estimated from ρ(Δt) and $\left\{\tau_{AC},\beta \right\}$ are obtained by maxmimum likelihood. The parameter β accommodates for a possible noise floor in ρ(τ), which typically results from residual embryo-to-embryo variability; on average β=0.04. Errors on $\Sigma_{AC}$ and $\tau_{AC}$ are computed from bootstrapping the fit to $\rho_{s}(\tau )$ (Eq. 27) over MCMC samples.

##### d. Two-state model auto-correlation.

To help us interpret the auto-correlation function, we investigated the prediction of the 2-state model of transcriptional bursting [23, 32]. In the 2-state model, the gene toggles stochastically between an ON (n=1) and OFF(n=0) states with rates $k_{ON}$ and $k_{OFF}$, respectively. In the ON state, the gene promoter can initiate transcription with initiation rate k leading to the synthesis of g transcripts. Within that model, we further define the switching correlation time $T=1/\left(k_{ON}+k_{OFF}\right)$ and steady-state mean occupancy $\eta =k_{ON}/\left(k_{ON}+k_{OFF}\right.$). In what follows, we focus on the stationary case ($k_{ON}$, $k_{OFF}$, and k don’t change over time), which is easier to interpret. From the master equation [71, 72], we derive the equations that describe the time evolution of the moments:

(30) $$\left\{\begin{array}{l} \frac{d}{dt}\langle n\left(t\right)\rangle \;=\frac{1}{T}\left(\eta -\langle n\left(t\right)\rangle \right)\\ \frac{d}{dt}\langle g\left(t\right)\rangle \;=k\langle n\left(t\right)\rangle \\ \frac{d}{dt}\langle g\left(1,t\right)\rangle \;=k\langle n\left(t\right)\rangle +\frac{1}{T}\left(\eta \langle g\left(t\right)\rangle -\langle g\left(1,t\right)\rangle \right)\\ \frac{d}{dt}g^{2}\left(t\right)\;=k\left(\langle n\left(t\right)\rangle +2\langle g\left(1,t\right)\rangle \right) \end{array}\right.$$

⟨n(t)⟩ is the mean occupancy of the ON-state (or equivalently the ON-probability $P_{ON}$), ⟨g(t)⟩ is the mean number of initiation events (or transcripts), ⟨g(1,t)⟩=⟨g(t)⟩-⟨g(0,t)⟩ is the mean number of initiation events conditioned on the gene being ON, and $\left\langle g^{2}(t)\right\rangle$ is the second moment of g. Solving the equations above (Eq. 30) with initial conditions $\langle n(t=0)\rangle =n_{0},\langle g(t=0)\rangle =\langle g(1,t=0)\rangle =\left\langle g^{2}(t=0)\right\rangle =0$, we determine the mean μ(t)≡⟨g(t)⟩ and the variance $\sigma^{2}(t)=\left\langle g^{2}(t)\right\rangle -\langle g(t)\rangle$ of the number of transcription initiation events g over the period [0,t]. In the special case $t=\tau_{\text{elo}}$ (the elongation time), $\mu \left(\tau_{\text{elo}}\right)$ and $\sigma^{2}\left(\tau_{\text{elo}}\right)$ correspond to the mean and the variance of the number of nascent transcripts on the gene (i.e., the transcriptional activity), under the assumption that elongation is a deterministic process at constant rate $K_{\text{elo}}$.

Assuming the gene is initially at steady-state $\left(n_{0}=\eta \right)$, we solve Eq. 30 and get:

(31) $$\begin{array}{l} \langle g(t)\rangle \;=k\eta t\\ \left\langle g(1,t)\rangle \;=k\eta^{2}t+k\eta (1-\eta )T(1-exp(-t/T)\right)\\ \left\langle g(0,t)\rangle \;=k\eta (1-\eta )t-k\eta (1-\eta )T(1-exp(-t/T)\right)\\ \left\langle g^{2}(t)\right\rangle \;=k\eta t+k^{2}\eta^{2}t^{2}+2k^{2}\eta (1-\eta )\left(T^{2}exp(-t/T)+tT-T^{2}\right). \end{array}$$

To compute the auto-correlation, we further need the transient of the first moments obtained by solving Eq. 30 with generic initial condition $\left\langle n(t=0)\rangle =n_{0}\in \{0,\;1\right\}$:

(32) $$\begin{array}{l} \left\langle n\left(t|n_{0}\right)\right\rangle \;=\eta (1-exp(-t/T))+n_{0}exp(-t/T)\\ \left\langle g\left(t|n_{0}\right)\right\rangle \;=k\eta t+k\left(n_{0}-\eta \right)T(1-exp(-t/T)). \end{array}$$

The transcriptional activity at time t is given by $g\left(t;t-\tau_{\text{elo}}\right.$), which is the total number of nascent transcripts (on the gene) initiated over the period $\left[t,t-\tau_{\text{elo}}\right]$. In the stationary case, the transcriptional activity is simply given by $\left\langle g\left(t;t-\tau_{\text{elo}}\right)\right\rangle =\left\langle g\left(\tau_{\text{elo}}\right)\right\rangle$. Using this definition, we then define the normalized auto-correlation of the transcriptional activity as

(33) $$\begin{array}{l} \rho_{A}(\tau )\;=\frac{\left\langle \left(g\left(t;\;t\;-\;\tau_{\text{elo}}\right)\;-\;\mu \left(\tau_{\text{elo}}\right)\right)\left(g\left(t\;+\;\tau ;\;t\;+\;\tau \;-\;\tau_{\text{elo}}\right)\;-\;\mu \left(\tau_{\text{elo}}\right)\right)\right\rangle }{\sigma^{2}\left(\tau_{\text{elo}}\right)}\\ \;=\frac{\left\langle g\left(t\;+\;\tau ;\;t\;+\;\tau \;-\;\tau_{\text{elo}}\right)g\left(t;\;t\;-\;\tau_{\text{elo}\;}\right)\right\rangle \;-\;\mu^{2}\left(\tau_{\text{elo}}\right)}{\sigma^{2}\left(\tau_{\text{elo}}\right)}. \end{array}$$

And similarly for the normalized auto-correlation of the transcription rate r(t)=g(t;t-Δt)/Δt (where Δt=10s is the sampling time), we have:

(34) $$\begin{array}{l} \rho_{R}(\tau )\;=\frac{\langle (g\left(t;\;t\;-\;\Delta t\right)-\;\mu (\Delta t))(g\left(t\;+\;\tau ;\;t\;+\;\tau \;-\;\Delta t\right)-\;\mu (\Delta t))\rangle /(\Delta t{)}^{2}}{\sigma^{2}(\Delta t)/(\Delta t{)}^{2}}\\ \;=\frac{\left\langle g\left(t\;+\;\tau ;\;t\;+\;\tau \;-\;\Delta t\right)g\left(t;\;t\;-\;\Delta t\right)\right\rangle -\;\mu^{2}(\Delta t)}{\sigma^{2}(\Delta t)}. \end{array}$$

As it turns out, these auto-correlation functions are almost identical and only differ on the integration period $\tilde{\tau }$, which is set to $\tilde{\tau }=\tau_{\text{elo}}$ to compute $\rho_{A}(\tau )$ and to $\tilde{\tau }=\Delta t$ to compute $\rho_{R}(\tau )$. In addition, the mean μ(t)≡⟨g(t)⟩ and the variance $\sigma^{2}(t)=\left\langle g^{2}(t)\right\rangle -\langle g(t)\rangle$ are fully determined by the solutions in Eq. 31. However, we still need to calculate the term $\left\langle g(t+\tau ;t+\tau -\tilde{\tau })g(t;t-\tilde{\tau })\right\rangle$. This term is easily calculated by splitting the calculation into two separate cases:

1. When $g(t+\tau ;t+\tau -\tilde{\tau })$ and $g(t;t-\tilde{\tau })$ share the same underlying trajectory, i.e., when $0\leq \tau <\tilde{\tau }$.
2. When $g(t+\tau ;t+\tau -\tilde{\tau })$ and $g(t;t-\tilde{\tau })$ are disjoint, i.e., when $\tau \geq \tilde{\tau }$.

We can then take advantage of the fact that $g\left(t_{1};t_{3}\right)=g\left(t_{1};t_{2}\right)+g\left(t_{2};t_{3}\right)$, provided we preserve the underlying connection of the gene state n at $t_{2}$.

###### Case 1: $0\leq \tau <\tilde{\tau }$.

Introducing $t_{1}\;:=t+\tau$, $t_{2}\;:=t$, $t_{3}\;:=t-\tilde{\tau }+\tau$ and $t_{4}\;:=t-\tilde{\tau }$ such that $t_{1}\geq t_{2}\geq t_{3}\geq t_{4}$, we get:

(35) $$\begin{array}{l} \langle g(t+\tau ;t-\tilde{\tau }+\tau )g(t;t-\tilde{\tau })\rangle \;=\left\langle \left(g\left(t_{1};t_{2}\right)+g\left(t_{2};t_{3}\right)\right)\left(g\left(t_{2};t_{3}\right)+g\left(t_{3};t_{4}\right)\right)\right\rangle \\ \;={\left\langle g\left(t_{1}-t_{2}\right)\right\rangle }_{n|n}\left\langle g\left(t_{2}-t_{3}\right)\right\rangle +{\left\langle g\left(t_{1}-t_{2}\right)\right\rangle }_{n|n^{\prime }}\left\langle g\left(t_{3}-t_{4}\right)\right\rangle \\ \;+{\left\langle g\left(t_{2}-t_{3}\right)\right\rangle }_{n|n}\left\langle g\left(t_{3}-t_{4}\right)\right\rangle +\left\langle g^{2}\left(t_{2}-t_{3}\right)\right\rangle , \end{array}$$

where the symbol n∣n means that the products are “connected” through the temporal correlation in n. These products are computed as follows:

$${\left\langle g\left(t_{1}-t_{2}\right)\right\rangle }_{n|n}\left\langle g\left(t_{2}-t_{3}\right)\right\rangle =\sum_{n=0}^{1}\;\langle g(\tau |n)\rangle \langle g(n,\tilde{\tau }-\tau )\rangle$$

$${\left\langle g\left(t_{1}-t_{2}\right)\right\rangle }_{n|n^{\prime }}\left\langle g\left(t_{3}-t_{4}\right)\right\rangle =\sum_{n,n^{\prime }=0}^{1}\;\langle g(\tau |n)\rangle P\left(n|n^{\prime };\tilde{\tau }-\tau \right)\left\langle g\left(n^{\prime },\tau \right)\right\rangle$$

$${\left\langle g\left(t_{2}-t_{3}\right)\right\rangle }_{n|n}\left\langle g\left(t_{3}-t_{4}\right)\right\rangle =\sum_{n=0}^{1}\;\left\langle g\left(\tilde{\tau }-\tau |n\right)\right\rangle \left\langle g\left(n,\tau \right)\right\rangle ,$$

where both ⟨g(t∣n)⟩ and ⟨g(n,t)⟩ have been calculated above (Eq. 31 and 32), and $P\left(n|n^{\prime };t\right)$ is the propagator (i.e., the transition probabilities) of the telegraph process:

(36) $$P\left(n|n^{\prime };t\right)=(n\eta +(1-n)(1-\eta ))(1-exp(-t/T))+\delta_{nn^{\prime }}exp(-t/T),$$

where $\delta_{nn^{\prime }}$ is the Kronecker delta. We thus have determined all the terms in Eq. 35 and we can finally obtain:

(37) $$\begin{array}{l} \left\langle g(t+\tau ;t-\tilde{\tau }+\tau )g(t;t-\tilde{\tau })\rangle \;=k\eta (\tilde{\tau }-\tau )+k^{2}\eta^{2}{\tilde{\tau }}^{2}+2k^{2}\eta (1-\eta )T(\tilde{\tau }-\tau \right)\\ \;+k^{2}\eta (1-\eta )T^{2}(exp(-(\tilde{\tau }+\tau )/T)+exp(-(\tilde{\tau }-\tau )/T)-2exp(-\tau /T)). \end{array}$$

When τ=0, the expression above reduces to $\left\langle g^{2}(\tilde{\tau })\right\rangle$, consistent with the fact that all the terms in Eq. 35 vanish except $\left\langle g^{2}\left(t_{2}-t_{3}\right)\right\rangle =\left\langle g^{2}(\tilde{\tau })\right\rangle$. Since, $\left\langle g^{2}(\tilde{\tau })\right\rangle -\langle g(\tilde{\tau })\rangle^{2}=\sigma^{2}(\tilde{\tau })$, we thus properly recover ρ(0)=1 as expected.

###### Case 2: $\tau \geq \tilde{\tau }$.

Introducing $t_{1}\;:=t+\tau$, $t_{2}\;:=t+\tau -\tilde{\tau }$, $t_{3}\;:=t$ and $t_{4}\;:=t-\tilde{\tau }$ such that $t_{1}\geq t_{2}\geq t_{3}\geq t_{4}$, we get:

$$\begin{array}{l} \langle g(t+\tau ;t-\tilde{\tau }+\tau )g(t;t-\tilde{\tau })\rangle \;=\left\langle \left(g\left(t_{1};t_{2}\right)g\left(t_{3};t_{4}\right)\right\rangle \right.\\ \;={\left\langle g\left(t_{1}-t_{2}\right)\right\rangle }_{n|n^{\prime }}\left\langle g\left(t_{3}-t_{4}\right)\right\rangle , \end{array}$$

where the connected product is given by

$${\left\langle g\left(t_{1}-t_{2}\right)\right\rangle }_{n|n^{\prime }}\left\langle g\left(t_{3}-t_{4}\right)\right\rangle =\sum_{n,n^{\prime }=0}^{1}\left\langle g\left(\tilde{\tau }|n\right)\right\rangle P\left(n|n^{\prime };\tau -\tilde{\tau }\right)\left\langle g\left(n^{\prime },\tilde{\tau }\right)\right\rangle .$$

Again, using the expression for ⟨g(t∣n)⟩ and ⟨g(n,t)⟩ (Eq. 31 and 32), and for $P\left(n|n^{\prime };t\right)$ (Eq. 36), we finally get:

(38) $$\left\langle g(t+\tau ;t-\tilde{\tau }+\tau )g(t;t-\tilde{\tau })\rangle =k^{2}\eta^{2}{\tilde{\tau }}^{2}+k^{2}\eta (1-\eta )T^{2}(1-exp(-\tilde{\tau }/T){)}^{2}exp(-\tau /T\right)$$

###### Conclusion.

We have calculated the normalized auto-correlation functions $\rho_{A}(\tau )$ (Eq. 33, $\tilde{\tau }=\tau_{\text{elo}}$) and $\rho_{R}(\tau )$ (Eq. 34, $\tilde{\tau }=\Delta t$) using the two-state model. Indeed, using Eq. 31, 37 and 38, we found

(39) $$\rho (\tau )=\left\{\begin{array}{cc} \frac{\tilde{\tau }\;-\;\tau \;+\;k(1\;-\;\eta )\left(2\left(\tilde{\tau }\;-\;\tau \right)T\;+\;T^{2}\left(exp\left(-\frac{\tilde{\tau }+\tau }{T}\right)+exp\left(-\frac{\tilde{\tau }-\tau }{T}\right)-2exp\left(-\frac{\tau }{T}\right)\right)\right)}{\tilde{\tau }\;+\;2k(1\;-\;\eta )\left(T^{2}\exp\left(-\frac{\tilde{\tau }}{T}\right)\;+\;\tilde{\tau }T\;-\;T^{2}\right)} & \text{if}\;\tau <\tilde{\tau }\\ \frac{k(1\;-\;\eta )T^{2}{\left(1\;-\;exp\left(-\frac{\tilde{\tau }}{T}\right)\right)}^{2}exp\left(-\frac{\tau }{T}\right)}{\tilde{\tau }\;+\;2k(1\;-\;\eta )\left(T^{2}\exp\left(-\frac{\tilde{\tau }}{T}\right)+\;\tilde{\tau }T\;-\;T^{2}\right)} & \text{if}\;\tau \geq \tilde{\tau } \end{array}\right.$$

Using the expression above, we plot $\rho_{A}(\tau )$ and $\rho_{R}(\tau )$ for different values of η in fig. S5A (left and right respectively). For $\rho_{A}(\tau )$, we clearly see two regimes: one that is dominated by the elongation process $\tau <\tau_{\text{elo}}$ and one that is only dictated by the switching process $\tau \geq \tau_{\text{elo}}$. As $\rho_{R}(\tau )$ is unencumbered by the elongation process, only the exponential decay due to promoter switching remains. Importantly, these auto-correlation functions are identical for two independent gene copies (2 sister chromatids), since the correlated contributions and the variances add (in the independent case).

From Eq. 39, the auto-correlation of the single-allele transcription rate $\rho_{R}(\tau )$ (with $\tilde{\tau }=\Delta t$) can be further simplified. Indeed, since for real data τ becomes a multiple of the sampling time Δt, we simply get

(40) $$\rho_{R}(\tau )=\left\{\begin{array}{cc} 1 & \text{if}\;\tau =0\\ \Sigma_{AC}exp\left(-\frac{\tau -\Delta t}{T}\right) & \text{if}\;\tau \geq \Delta t \end{array}\right.$$

Here the amplitude of the correlated variability $\Sigma_{AC}$ is given by

(41) $$\Sigma_{AC}=\frac{k\Delta t(1\;-\;\eta )\phi_{2}(\Delta t/T)}{1\;+\;k\Delta t(1\;-\;\eta )\phi_{1}(\Delta t/T)},$$

with filtering functions $\phi_{1}(x)\in [0,\;1]$ and $\phi_{2}(x)\in [0,\;1]$ given by

$$\phi_{1}(x)=2\frac{exp(-x)\;+\;x\;-\;1}{x^{2}}$$

$$\phi_{2}\left(x\right)=\frac{(1\;-\;exp(-x){)}^{2}}{x^{2}}.$$

From Eq. 40, we clearly see that in the 2-state model, the time scale of the exponential decay is solely dictated by the switching correlation time T. This suggests that the measured correlation time $\tau_{AC}$ results from bursts and is in fact related to T (Fig. 1E and 1F). Importantly, $\Sigma_{AC}$ displays two opposite behaviors depending on whether the mean transcription rate R=kη is varied through k or η (Eq. 41 and fig. S5B). Indeed, an increase in k leads to an increase in $\Sigma_{AC}$, while an increase in η leads to a decrease $\Sigma_{AC}$. Interestingly, the scaling of $\Sigma_{AC}$ with R observed in data is very much in line with the latter scenario (fig. S5C), i.e. changes in R driven by the ON-probability η. Lastly, we also see that $\Sigma_{AC}$ vanishes when Δt→0 whereas the exponential decay is lost when Δt≳T. This implies there is a range of suitable Δt that provide sufficient integration Δt≫0 but still remain sufficiently small Δt<T to observe the correlation in the signal. Given, the range of measured $\Sigma_{AC}∼0.2-0.6$ and $\tau_{AC}∼1-2min$ (see Fig. 1F and fig. S5C), it appears that our sampling time Δt≈10s is adequate.

##### e. Validating auto-correlation on simulated data.

To assess our ability to estimate the auto-correlation (AC) function properly, we performed single allele deconvolution on simulated data mimicking experimental conditions. Specifically, we compared the estimated AC parameters ($\Sigma_{AC}$ and $\tau_{AC}$) obtained through deconvolution to the known input parameters used to generate the simulated data. For each combination of input parameters, we generated 200 synthetic alleles and 50 min long activity time series using the Gillespie algorithm [73], including measurement noise consistent with real data and assuming two independent gene copies (i.e., sister chromatids) described by the 2-state model (See Section V D0e “Validation using synthetic data” for further details). Overall, we probed 56 combinations of (single gene copy) input parameters with $k\equiv K^{\left(1\right)}=8\;mRNA/min$ (consistent with real data), $\eta \equiv P_{ON}^{\left(1\right)}\in \{0.03,\;0.12,\;0.23,\;0.36,\;0.52,\;0.78,\;0.90\}$ and $T\equiv T_{C}^{\left(1\right)}\in \left\{0.5,\;1,\;2,\;3,\;4,\;5,\;7,\;10\right\}\;min$.

For each combination of input parameters, we calculated the input $\Sigma_{AC}$ according to Eq. 41, while the input $\tau_{AC}$ is simply given by the input T. Using the simulated data, we estimated $\Sigma_{AC}$ and $\tau_{AC}$ from the instantaneous single-allele transcription rate, as described in Section V C 0 c “Transcription rate auto-correlation”. In fig. S5D, we show that for both $\Sigma_{AC}$ and $\tau_{AC}$, the input and the estimated AC parameters strongly correlate over a large range of $P_{ON}^{\left(1\right)}$ and $T_{C}^{\left(1\right)}$ values, demonstrating our ability to faithfully determine $\Sigma_{AC}$ and $\tau_{AC}$ from deconvolved instantaneous single-allele transcription rates. Thus, the striking collapse of $\Sigma_{AC}$ and $\tau_{AC}$ observed in real data (Fig. 1F and fig. S5C), across genes and activity, should not be markedly affected by our approach and appears to be a solid result.

Lastly, to assess the potential impact of errors in the elongation rate on the deconvolution process and the autocorrelation estimation, we compared the estimated AC parameters using the correct deconvolution kernel (used to generate the simulated data and computed here with $K_{\text{elo}}=2\;mRNA/min$) and incorrect kernels (computed with either with $K_{\text{elo}}=1.5$ or 2.5 mRNA/min, i.e., a ±25% error). We focused on the analysis of the $T_{C}^{\left(1\right)}=2\;min$ simulated data set (that is closest to real data) and show the estimated $\tau_{AC}$ and $\Sigma_{AC}$ using the three kernels for all values of input $P_{ON}^{\left(1\right)}$ (in fig. S5E). The analysis determines that while the effect of a 25% deviation on $K_{\text{elo}}$ has only a very limited impact on $\Sigma_{AC}$ (resulting error on $\Sigma_{AC}∼4\%$), the estimation of $\tau_{AC}$ is almost entirely unaffected by a 25% overestimation of $K_{\text{elo}}$ (error on $\tau_{AC}∼7\%$). However, an underestimation of $K_{\text{elo}}$ leads to a significant underestimation of $\tau_{AC}$, albeit with errors on $\tau_{AC}$ not exceeding 25% on average. Overall, this demonstrates the robustness of our approach to changes in the elongation rate.

#### D. Burst calling and transcriptional parameter estimation

##### a. Calling bursts from deconvolved time series.

To call bursts from individual single-allele transcriptional activity time series A, we opt for a simple approach with minimal mechanistic assumptions. We simply aim to cluster the deconvolved configurations of initiation events I underlying A over time (see Eq. 14 and Fig. 1D). We first compute the single-allele transcription rate r(t) from I (see Eq. 18). We then perform a (centered) temporal moving average of r(t) using the following kernel:

(42) $$\kappa \left(t\right)=\frac{1}{Z\left(w\right)}\exp\left(-{\left(\frac{2t}{w}\right)}^{4}\right),$$

where w is the width of the moving window and $Z(w)=\sum_{i=1}^{N_{t}}\;\kappa \left(t_{i}\right)$ is the normalization. We compute the moving average through discrete convolution resulting in the following rate $r_{w}(t)=r(t)\;*\;\kappa (t)$. Subsequently, a threshold $r_{b}=g_{b}/w$ is applied to the resulting rate estimate $r_{w}$, where $g_{b}$ corresponds to the desired minimum number of initiations events for a burst. This threshold allows us to determine the cluster of initiation events (bursts) defining the allele’s ON-state n(t)∈{0,1}:

(43) $$n(t)=\left\{\begin{array}{lll} 1 & \text{if} & r_{w}(t)\geq r_{b}\\ 0 & \text{if} & r_{w}(t)<r_{b} \end{array}\right.$$

It follows that the clustering approach described above and the ensuing definition of the ON state n(t) depend on two free parameters: the width of the averaging window w and the minimal number of initiation events $g_{b}$. As a rule of thumb, w needs to be large enough to average the Poisson initiation fluctuations and prevent the calling of spurious bursts, but small enough to preserve the temporal correlations attributed to ON-OFF switching in the data. In practice, we set w=5Δt≈50s, which is slightly below the measured correlation time $\tau_{AC}∼1-2\;min$ (Fig. 2F). Regarding the threshold, we set $g_{b}=2$ corresponding to a detection of at least 2 initiation events within the moving window, which is motivated by our detection sensitivity of around 1–2 mRNAs (see fig. S1E. and Section IVD). Notably, the shape of the kernel (Eq. 42) and the chosen parameter values (w=5Δt and $g_{b}=2$) set a lower bound on the minimal ON interval duration that can be reliably detected, which is 3Δt≈30s. This value corresponds to the duration of the ON interval calculated from n(t) with input $I(t)=g_{b}\delta (t)$. To verify the impact of our choice of clustering parameters on the estimation of bursting parameters go to Section V D 0 e Validation using synthetic data.

Importantly, our estimation of the ON state n through clustering can be applied to each individual initiation configuration I sampled from the posterior distribution P(I∣A) (Eq. 14). It implies that we can easily reconstruct P(n∣A) for each allele, as we did for the instantaneous distribution of the single-allele transcription rate P(r∣A) (see Section V A 0 e; Estimating single allele transcription rate). Using our MCMC samples we get

(44) $$P\left(n|A\right)=\sum_{I}P\left(n|I\right)P\left(I|A\right),$$

where P(n∣I)=1 when Eq. 43 is satisfied and P(n∣I)=0 otherwise. The distribution P(n∣A) allows us to assess the uncertainty on burst calling for each time series of transcriptional activity A.

##### b. Estimating transcriptional parameters.

To characterize the bursting dynamics of each gap gene in space and time, we define a set of bursting parameters and estimate them empirically from the single-allele transcription rates r(t) and the ON-state n(t) (see fig. S6A–B). For a given single-allele transcriptional activity time series A, we sampled individual initiation configurations $I_{s}$ from the posterior distribution P(I∣A) using MCMC (see Section V A). The index $s\in \left\{1,\;2,\ldots ,N_{s}\right\}$ denotes one specific MCMC sample drawn from the posterior, where $N_{s}=10^{3}$ is the total number of samples used per allele. For each $I_{s}$, we further computed $r_{s}(t)|A$ (Eq. 18) and $n_{s}(t)|A$ (Eq. 43), where the index s and the notation ∣A specify that these come from the sample $I_{s}$ given the time series A. We then defined the mean transcription rate R and the ON-probability $P_{ON}$ over all nuclei in a specific spatiotemporal (x,t)-bin, that is over all the A∈𝒜(x,t). For a single MCMC sample s per A we get

(45) $$\left.R_{s}\left(x,t\right)=\frac{1}{N_{A}\left(x,t\right)}\sum_{A\in 𝒜\left(x,t\right)}r_{s}\left(t\right)\right|\;A$$

(46) $$\left.P_{\text{ON},s}\left(x,t\right)=\frac{1}{N_{A}\left(x,t\right)}\sum_{A\in 𝒜\left(x,t\right)}n_{s}\left(t\right)\right|\;A,$$

where $N_{A}(x,t)=\#𝒜(x,t)$. We then compute a single point-estimate per bursting parameter Θ for each position x and time t by averaging over our MCMC samples as follows

(47) $$\text{Θ}\left(x,t\right):=\langle {\text{Θ}}_{s}\left(x,t\right)\rangle =\frac{1}{N_{s}}\sum_{s=1}^{N_{s}}{\text{Θ}}_{s}\left(x,t\right)$$

(48) $$\sigma_{\Theta }(x,t)=\sqrt{\left\langle {\left(\Theta_{s}(x,t)-\Theta (x,t)\right)}^{2}\right\rangle },$$

where $\sigma_{\Theta }$ is our estimation of the error on Θ. Thus, the mean transcription rate and the ON-probability are given by $R(x,t)=\left\langle R_{s}(x,t)\right\rangle$ and $P_{ON}(x,t)=\left\langle P_{ON,s}(x,t)\right\rangle$ (see Fig. 2A and fig. S6C). Importantly, this construction of R(x,t) is equivalent to our previous calculation using the mixture of posterior distributions (see Eq. 22).

The mean initiation rate K is defined over all nuclei in a spatiotemporal (x,t)-bin by averaging the single-allele transcription rate r(t) conditioned on the allele being ON(n(t)=1). It is expressed as

(49) $$K_{s}\left(x,t\right)\;=\frac{1}{Z_{K}}\sum_{A\in 𝒜\left(x,t\right)}r_{s}\left(t\right)\left|A\cdot n_{s}\left(t\right)\right|A\;\;Z_{K}\;=\sum_{A\in 𝒜\left(x,t\right)}n_{s}\left(t\right)|A,$$

where $Z_{K}$ counts the number of ON alleles at time t. Averaging over MCMC samples (Eq. 47) leads to the the mean initiation rate $K(x,t)=\left\langle K_{s}(x,t)\right\rangle$ (see fig. S6D). By construction $R=K\cdot P_{ON}$, which we verify empirically (see fig. S6F). As additional control, we define a “leaking” rate $K_{L}$ by averaging the single-allele transcription rate r(t) conditioned on the allele being OFF(n(t)=0):

(50) $$K_{L,s}\left(x,t\right)\left.=\frac{1}{Z_{L}}\sum_{A\in 𝒜\left(x,t\right)}r_{s}\left(t\right)\right|\;A\cdot \left(1-n_{s}\left(t\right)|A\right)\;Z_{L}\;=\sum_{A\in 𝒜\left(x,t\right)}1-n_{s}\left(t\right)|A.$$

We verified that $K_{L}(x,t)=\left\langle K_{L,s}(x,t)\right\rangle$ is indeed negligible compared to K(x,t), supporting our ability to identify well-demarcated bursts (fig. S6G).

To calculate the mean durations of the ON and OFF periods ($T_{ON}$ and $T_{OFF}$) for each spatiotemporal (x,t)-bin, we first determine the boundaries of the ON(n(t)=1) and OFF(n(t)=0) periods for individual alleles. Using n(t), we built a function b(t) such that b(t)=1 whenever time point t corresponds to an ON-OFF boundary:

(51) $$b(t)=\left\{\begin{array}{lll} 1 & \text{if} & |n(t+\Delta t)-n(t)|>0\\ 0 & \text{if} & |n(t+\Delta t)-n(t)|=0 \end{array}\right.$$

where Δt is the sampling time in our measurements. b(t) defines ON-OFF boundaries that are consistent with our definition of the instantaneous single-allele transcription rate r(t) (see Eq. 18), i.e., we associate the state n(t) and the rate r(t) to the same time interval (t-Δt,t]. For convenience, we also enforce $b\left(t_{1}\right)=1$ and $b\left(t_{N_{t}}\right)=1$ for the first and last time point $t_{1}$ and $t_{N_{t}}$ of the time series.

From b(t) we construct the set of boundary time points, i.e, $𝒯=\{t\;:b(t)=1\}=\left\{t_{b_{1}},\ldots ,t_{b_{k}}\right\}$ such that $b_{j}<b_{j+1}\;\forall j\in \{1,\;2,\ldots ,k-1\}$ where k≥2 (in each case $t_{b_{1}}=t_{1}$ and $t_{b_{k}}=t_{N_{t}}$). To measure the durations of individual ON and OFF periods, we introduce a function τ(t) such that

(52) $$\tau (t)=\left\{\begin{array}{ll} t_{b_{2}}-t_{b_{1}} & \;\text{if}\;t=t_{1}\\ t_{b_{j+1}}-t_{b_{j}} & \;\text{else}\;\text{if}\;\;t\in \left(t_{b_{j}},t_{b_{j+1}}\right] \end{array}\right.$$

We further introduce a function π(t)=τ(t)/Δt that counts the number of Δt intervals underlying τ(t). We calculate $T_{ON}$ from the activity time series A as

(53) $$T_{\text{ON},s}\left(x,t\right)\left.=\frac{1}{Z_{\text{ON}}}\sum_{A\in 𝒜\left(x,t\right)}\tau_{s}\left(t\right)\right|\;A\cdot \frac{n_{s}\left(t\right)|A}{\pi_{s}\left(t\right)|A}\;\;Z_{\text{ON}}\;=\sum_{A\in 𝒜\left(x,t\right)}\frac{n_{s}\left(t\right)|A}{\pi_{s}\left(t\right)|A},$$

where $Z_{ON}$ counts the number of ON alleles at time t weighted by the inverse of the length of the underlying ON periods (s denotes an individual MCMC sample as before). The rationale behind the $\frac{n_{s}(t)|A}{\pi_{s}(t)|A}/Z_{ON}$ weights is as follows: as we aim to estimate mean durations across nuclei at time t (fig. S8A), we need to account for the length of each period τ(t) and correct for their overall contribution to π(t) time points (e.g. longer periods contribute to more time points, thus we need weights that balance over-counting).

Finally, by averaging over MCMC samples (Eq. 47), we obtain the mean ON duration $T_{ON}(x,t)=\left\langle T_{ON,s}(x,t)\right\rangle$ (see Fig. 2C). Likewise, for $T_{OFF}$, we have

(54) $$T_{\text{OFF},s}\left(x,t\right)\left.=\frac{1}{Z_{\text{OFF}}}\sum_{A\in 𝒜\left(x,t\right)}\tau_{s}\left(t\right)\right|\;A\cdot \frac{1-n_{s}\left(t\right)|A}{\pi_{s}\left(t\right)|A}\;\;Z_{\text{OFF}}\;=\sum_{A\in 𝒜\left(x,t\right)}\frac{1-n_{s}\left(t\right)|A}{\pi_{s}\left(t\right)|A}.$$

Again, averaging over MCMC samples (Eq. 47) leads to the mean OFF duration $T_{OFF}(x,t)=\left\langle T_{OFF,s}(x,t)\right\rangle$ (see Fig. 2C).

When estimating the initiation rate K (Eq. 49), and the mean durations of the ON and OFF periods $T_{ON}$ (Eq. 53) and $T_{OFF}$ (Eq. 54), we sometimes encounter missing values, e.g. absence of ON or OFF periods over all nuclei at a given time point t. This typically occurs when $P_{ON}(t)\to 0$ or $P_{ON}(t)\to 1$. Therefore, we filter each bursting parameter estimate $\Theta_{s}(x,t)$ using a Gaussian kernel over a short timescale of 1 min. This “smoothing” interpolates the eventual missing values and prevents gaps in our time-dependent parameter estimation.

Using K, $T_{ON}$ and $T_{OFF}$, we can further define the following bursting parameters:

(55) $$T_{C}\;:=\frac{T_{ON}T_{OFF}}{T_{ON}+T_{OFF}}$$

(56) $$B\;:=K\cdot T_{ON}$$

(57) $$F\;:=\frac{1}{T_{ON}+T_{OFF}},$$

where $T_{C}$ is the switching correlation time, B the burst size and F the burst frequency. These parameters were directly computed from our estimates of K, $T_{ON}$, and $T_{OFF}$ as defined above. Importantly, we tested extensively our ability to estimate static and time-dependent bursting parameters correctly using simulated data (see Section V D 0 e; Validation using synthetic data).

##### c. Sister chromatids and single copy parameters

In the blastoderm phase of the Drosophila embryo, DNA replication occurs during the first few minutes of each nuclear cycle (2–3 min after mitosis). Therefore, as our microscopy does not allow for separating individual sister chromatids, imaged spots decorated with fluorescently labeled nascent transcripts typically correspond to two optically unresolved sites of transcription. Assuming the measured activity results from two *identical* and *independent* alleles, we transform our effective bursting parameters into single-gene copy (SGC) parameters. In that case, the mean SGC transcription rate $R^{\left(1\right)}$ is simply given by $R^{\left(1\right)}=R/2$, which is half the effective one. In addition, the SGC ON-probability $P_{ON}^{\left(1\right)}$ must satisfy

$$\underset{P(0\;\text{copy}\;\text{active)}}{\underbrace{{\left(1-P_{ON}^{\left(1\right)}\right)}^{2}}}+\underset{P(1\geq \;\text{copy}\;\text{active)}}{\underbrace{2P_{ON}^{\left(1\right)}\left(1-P_{ON}^{\left(1\right)}\right)+P_{ON}^{(1{)}^{2}}}}=1,$$

where the probability to observe at least one active gene copy corresponds to the effective $P_{ON}$. Thus, $1-{\left(1-P_{ON}^{\left(1\right)}\right)}^{2}=P_{\text{ON}}$ and we obtain the SGC ON-probability given by

(58) $$P_{ON}^{\left(1\right)}=1-{\left(1-P_{ON}\right)}^{1/2}.$$

Regarding the SGC mean initiation rate $K^{\left(1\right)}$, it must satisfy $K^{\left(1\right)}=R^{\left(1\right)}/P_{ON}^{\left(1\right)}=KP_{ON}/\left(2P_{ON}^{\left(1\right)}\right)$. Thus, using Eq. 58 we get

(59) $$K^{\left(1\right)}=\frac{K}{2}\left(1+{\left(1-P_{ON}\right)}^{1/2}\right).$$

For a single gene copy, the switching correlation time $T_{C}^{\left(1\right)}$ is exactly given by $T_{C}^{\left(1\right)}=1/\left(k_{ON}+k_{OFF}\right)$, where $k_{ON}$ and $k_{OFF}$ are the ON and OFF rates of the 2-state model (see Section VC 0 d). For a system made of two identicaland independent gene copies we can write the following kinetic equation:

(60) $$\lambda_{0}\underset{k_{OFF}}{\overset{2k_{ON}}{⇌}}\lambda_{1}\underset{2k_{OFF}}{\overset{k_{ON}}{⇌}}\lambda_{2},$$

where $\lambda_{0}$, $\lambda_{1}$, and $\lambda_{2}$ are states corresponding to zero, one, and two active gene copies, respectively. Again, the effective OFF-state is equivalent to $\lambda_{0}$, while the effective ON-state is either $\lambda_{1}$ or $\lambda_{2}$. Further assuming steady-state, from Eq. 60 we calculate the mean residence time in the OFF and ON states (using phase-type distributions), i.e., $T_{OFF}$ and $T_{ON}$ as a function of $k_{ON}$ and $k_{OFF}$:

(61) $$T_{OFF}\;=\frac{1}{2k_{ON}}\;T_{ON}\;=\frac{k_{ON}\;+\;2k_{OFF}}{2k_{OFF}^{2}}.$$

From our definition of the effective switching correlation time $T_{C}$ (Eq. 55), and the steady state relationships $k_{ON}=P_{ON}^{\left(1\right)}/T_{C}^{\left(1\right)}$ and $k_{OFF}=\left(1-P_{ON}^{\left(1\right)}\right)/T_{C}^{\left(1\right)}$, we get

$$T_{C}\;:=\frac{T_{ON}T_{OFF}}{T_{ON}\;+\;T_{OFF}}=\frac{k_{ON}\;+\;2k_{OFF}}{2{\left(k_{ON}\;+\;k_{OFF}\right)}^{2}}=\frac{1}{2}T_{C}^{\left(1\right)}\left(2-P_{ON}^{\left(1\right)}\right).$$

Using Eq. 58, we express the SGC switching correlation time as a function of $T_{C}$ and $P_{ON}$:

(62) $$T_{C}^{\left(1\right)}=\frac{2T_{C}}{1\;+\;{\left(1\;-\;P_{ON}\right)}^{1/2}}$$

Together Eq. 58, 59 and 62 provide the key relationships to transform our effective bursting parameters to SGC parameters (see fig. S10E–H, S7B–G and S12B). Note that the relationships between $T_{OFF}$, $T_{ON}$ and $T_{C}^{\left(1\right)}$ (Eq. 61 and 62) are in principle valid only near steady state. Nevertheless, these relationships provide good approximations in the non-stationary case given that the temporal changes of the underlying kinetics over developmental time are slow (see Section V D 0 e; Validation using synthetic data).

##### d. Predicting time-dependent bursting parameters.

To test our deconvolution and burst calling approaches on simulated data (modeled via the master equation), we need to predict the time dependence of the effective bursting parameters (ground truth) given the underlying input kinetic parameters. Indeed, most bursting parameters (i.e., $P_{ON}(t)$, $T_{ON}(t)$, $T_{OFF}(t)$ and $T_{C}(t)$; but not $\left.K(t)\right)$ result from non-trivial time integration of the underlying gene switching rates $k_{OFF}(t)$ and $k_{ON}(t)$. In addition, the time scales $T_{ON}(t)$, $T_{OFF}(t)$ and $T_{C}(t)$ when estimated from data are typically subjected to biases due to discreteness (e.g., missing events) and the finite nature (e.g., censoring) of the measured time intervals. To account for these effects, we compute the expected $P_{ON}(t)$, $T_{ON}(t)$, $T_{OFF}(t)$ and $T_{C}(t)$ given a 2-state model of transcription (for either a single allele or for each of the two independent alleles of the replicated sister chromatids) and the kinetic rates $k_{OFF}(t)$ and $k_{ON}(t)$ [23, 32].

Starting with the time evolution of the gene state n (with n∈{0,1} for a single gene copy and n∈{0,1,2} in the two gene copies system), the non-stationary master equation of the system [71, 72] is given by

(63) $$\frac{d}{dt}P\left(t\right)=\overset{ˆ}{M}\left(t\right)P\left(t\right),$$

where P(t) is the time-dependent vector whose components are the probabilities to find the system in any of the states n at time t. Thus, $\sum_{n}\;P_{n}(t)=1$ with the sum taken over all components of P(t). $\overset{ˆ}{M}(t)$ is the time-dependent state transition matrix containing the propensity functions of the different reactions. For a single gene copy, $\overset{ˆ}{M}(t)$ is given by

(64) $${\overset{ˆ}{M}}_{1}\left(t\right)=\left(\begin{array}{cc} -k_{ON}\left(t\right) & k_{OFF}\left(t\right)\\ k_{ON}\left(t\right) & -k_{OFF}\left(t\right) \end{array}\right).$$

For two independent and identical gene copies, $\overset{ˆ}{M}(t)$ is given by

(65) $${\overset{ˆ}{M}}_{2}\left(t\right)=\left(\begin{array}{ccc} -2k_{ON}\left(t\right) & k_{OFF}\left(t\right) & 0\\ 2k_{ON}\left(t\right) & -\left(k_{OFF}\left(t\right)+k_{ON}\left(t\right)\right) & 2k_{OFF}\left(t\right)\\ 0 & k_{ON}\left(t\right) & -2k_{OFF}\left(t\right) \end{array}\right).$$

The solution of the non-stationary master equation for the system (Eq. 63) is given by:

(66) $$P\left(t\right)=\overset{ˆ}{U}\left(t,t_{1}\right)P\left(t_{1}\right),$$

where $P\left(t_{1}\right)$ is the initial condition vector, and the propagator $\overset{ˆ}{U}\left(t,t_{1}\right)\equiv OE[\overset{ˆ}{M}]\left(t,t_{1}\right)$ is the time-ordered exponential of $\overset{ˆ}{M}(t)$. Intuitively, $\overset{ˆ}{U}\left(t,t_{1}\right)$ is a matrix describing the time evolution of the system whose entries correspond to the transition probabilities, i.e., ${\overset{ˆ}{U}}_{nn^{\prime }}\left(t,t_{1}\right)=P\left(n;t|n^{\prime };t_{1}\right)$, such that $P_{n}(t)=\sum_{n^{\prime }}\;{\overset{ˆ}{U}}_{nn^{\prime }}\left(t,t_{1}\right)P_{n^{\prime }}\left(t_{1}\right)$. There are multiple ways to define the time-ordered exponential $OE[\overset{ˆ}{M}]\left(t,t_{1}\right)$; here we define it such that our computation becomes an infinite product of matrix exponential:

(67) $$OE\left[\overset{ˆ}{M}\right]\left(t,t_{1}\right)=\underset{N\to \infty }{lim}\;\left(\exp\left(\int_{t_{N-1}}^{t_{N}}\;\overset{ˆ}{M}\left(t^{\prime }\right)dt^{\prime }\right)\ldots \exp\left(\int_{t_{2}}^{t_{3}}\;\overset{ˆ}{M}\left(t^{\prime }\right)dt^{\prime }\right)\exp\left(\int_{t_{1}}^{t_{2}}\;\overset{ˆ}{M}\left(t^{\prime }\right)dt^{\prime }\right)\right),$$

where $t_{i}=t_{1}+(i-1)\Delta t$ for i∈{1,…,N} and $\Delta t=\left(t-t_{1}\right)/(N-1)$. Provided the temporal changes in $k_{OFF}(t)$ and $k_{ON}(t)$ are small over individual Δt, the product above provides a good approximation to the time-ordered exponential when N is finite. In practice, we set Δt to our sampling time (∼10 s) and we approximated the integral using the trapezoidal rule such that $\int_{t_{i-1}}^{t_{i}}\;\overset{ˆ}{M}(t)dt\approx \frac{1}{2}\left(\overset{ˆ}{M}\left(t_{i}\right)+\overset{ˆ}{M}\left(t_{i-1}\right)\right)\Delta t$ leading to

(68) $$OE\left[\overset{ˆ}{M}\right]\left(t_{i},t_{1}\right)\approx \exp\left(\frac{1}{2}\left(\overset{ˆ}{M}\left(t_{i}\right)+\overset{ˆ}{M}\left(t_{i-1}\right)\right)\Delta t\right)\ldots \exp\left(\frac{1}{2}\left(\overset{ˆ}{M}\left(t_{2}\right)+\overset{ˆ}{M}\left(t_{1}\right)\right)\Delta t\right).$$

This approximation is quite convenient for discretized time intervals as is often the case for real data. Moreover, the matrix exponential is straightforward to compute and rather well-behaved (i.e., it preserves the normalization of the columns to one) [74].

We compute $P_{ON}(t)$ using the transition probabilities determined by the propagator (Eq. 66 and 68), namely $P_{ON}(t)=\sum_{n>0}\;P_{n}(t)$ with $P_{n}(t)=\sum_{n^{\prime }}\;{\overset{ˆ}{U}}_{nn^{\prime }}\left(t,t_{1}\right)P_{n^{\prime }}\left(t_{1}\right)$. Incidentally, for a single gene copy in the stationary case ($\overset{ˆ}{M}$ given by Eq. 64 with $k_{ON}$ and $k_{OFF}$ constant in time), the computation of $P_{ON}(t)$ above becomes equivalent to solving the moment equation for ⟨n(t)⟩, introduced previously (see Eq. 30).

To determine the expected $T_{ON}(t)$ and $T_{OFF}(t)$, we compute the discrete residence time distribution of the ON and OFF states in the non-stationary case. We define the sets of residence states ${𝒱}_{r}$ and absorbing states ${𝒱}_{a}$ such that $n\in {𝒱}_{r}\cup {𝒱}_{a}$ with ${𝒱}_{r}\cap {𝒱}_{a}=\emptyset$. For the ON-time we have ${𝒱}_{r}=\{1\}$ and ${𝒱}_{a}=\{0\}$ (1 gene copy) or ${𝒱}_{r}=\{1,\;2\}$ and ${𝒱}_{a}=\{0\}$ (2 gene copies). For the OFF-time we have ${𝒱}_{r}=\{0\}$ and ${𝒱}_{a}=\{1\}$ (1 gene copy) or ${𝒱}_{r}=\{0\}$ and ${𝒱}_{a}=\{1,\;2\}$ (2 gene copies). We define the probability $P_{r}\left(\tau =t_{j}-t_{i}|t_{i}\right)$ that the system has just settled at time $t_{i}$ in any of the residence states $\left(n\left(t_{i}\right)\in {𝒱}_{r}\right)$ and is found for the first time after a duration τ in any absorbing state $\left(n\left(t_{j}\right)\in {𝒱}_{a}\right)$. Given that time is discrete and finite, with $t_{i+1}-t_{i}=\Delta t\;\forall i\in \left\{1,\ldots ,N_{t}-1\right\}$ and $N_{t}$ the total number of time points, the residence time (or first-passage time) distribution $P_{r}\left(\tau =t_{j}-t_{i}|t_{i}\right)$ can be written as

(69) $$P_{r}\left(\tau =t_{j}-t_{i}|t_{i}\right)\equiv W_{ji},$$

where W is a triangular matrix (with $W_{ji}=0$ if j<i) whose columns correspond to truncated discrete phase-type distributions (truncated due to a finite number of time points $N_{t}$). The coefficients $W_{ji}$ are defined as follows

(70) $$W_{ji}=\left\{\begin{array}{ll} \sum_{n\in {𝒱}_{a}}Q_{n}\left(t_{j},t_{i}\right) & \;\text{if}\;i\leq j<N_{t}\\ 1-\sum_{k=i}^{N_{t}-1}W_{ki} & \;\text{if}\;j=N_{t} \end{array}\right.$$

where the value for $j=N_{t}$ ensures that the $W_{ji}$ form normalized distributions along j, i.e. $\sum_{j=i}^{N_{t}}\;W_{ji}=1$. In practice, these distributions were computed by defining $Q_{n}\left(t_{j},t_{i}\right)$ recursively for $1\leq i<j\leq N_{t}$ using the propagator (Eq. 66 and 68):

(71) $$Q_{n}\left(t_{j},t_{i}\right)=\sum_{n^{\prime }\in {𝒱}_{r}}{\hat{U}}_{nn^{\prime }}\left(t_{j},t_{j-1}\right)Q_{n^{\prime }}\left(t_{j-1},t_{i}\right)$$

The initial condition of our recursion $Q_{n}\left(t_{i},t_{i}\right)$ (when i=j) corresponds to the probability that the system has just settled in any of the residence states at time $t_{i}$. For $1<i\leq N_{t}$, this probability is given by

(72) $$Q_{n}\left(t_{i},t_{i}\right)=\left\{\begin{array}{cc} \frac{1}{Z_{i}}\sum_{n^{\prime }\in {𝒱}_{a}}{\hat{U}}_{nn^{\prime }}\left(t_{i},t_{i-1}\right)P_{n^{\prime }}\left(t_{i-1}\right) & \;\text{if}\;n\in {𝒱}_{r}\\ 0 & \;\text{otherwise}\; \end{array}\right.$$

where $Z_{i}=\sum_{n\in {𝒱}_{r}}\;\sum_{n^{\prime }\in {𝒱}_{a}}\;{\overset{ˆ}{U}}_{nn^{\prime }}\left(t_{i},t_{i-1}\right)P_{n^{\prime }}\left(t_{i-1}\right)$ with $P_{n}(t)$ the occupancy of the states given by Eq. 66. For i=1, $Q_{n}\left(t_{1},t_{1}\right)$ is actually ill-defined and we chose

(73) $$Q_{n}\left(t_{1},t_{1}\right)=\left\{\begin{array}{cc} \frac{1}{Z_{1}}P_{n}\left(t_{1}\right) & \text{if}\;\;n\in {𝒱}_{r}\\ 0 & \text{otherwise} \end{array}\right.$$

where $Z_{1}=\sum_{n\in {𝒱}_{r}}\;P_{n}\left(t_{1}\right)$. Consistent with our definition of $P_{r}\left(\tau |t_{i}\right)$, the probability that the system remains in a given state for a null duration is zero. Indeed, by construction $P_{r}\left(\tau =0|t_{i}\right)\equiv W_{ii}=0$ since $W_{ii}=\sum_{n\in {𝒱}_{a}}\;Q_{n}\left(t_{t},t_{i}\right)$ where $Q_{n}\left(t_{t},t_{i}\right)=0\;\forall n\in {𝒱}_{a}$.

To compute the expected $T_{ON}(t)$ and $T_{OFF}(t)$ according to our definition (see Eq. 53 and 54), we renormalize the computed residence time distributions. Specifically, we want to compute the average of $\tau =t_{j}-t_{i}$ for all possible $t_{i}$ given $t_{j}$, meaning we must properly normalize the $W_{ij}$ along i (they only form distributions along j). To do so, we first need to weight each $t_{i}$ according to the probability that the system settled in any residence state at that particular time, which is given by the previously computed $Z_{i}$ (see Eq. 72 and 73). In addition, we need to take into account that any τ contributes to j-i time points along j. Thus, with that in mind, we defined the following renormalized coefficient ${\tilde{W}}_{ji}$ for $1\leq i<j\leq N_{t}$:

(74) $${\tilde{W}}_{ji}=\frac{W_{ji}}{j-i}\frac{Z_{i}}{{\tilde{Z}}_{j}},$$

with ${\tilde{Z}}_{j}=\sum_{i<j}\;\frac{W_{ji}}{j-i}\frac{Z_{i}}{{\tilde{Z}}_{j}}$, such that the ${\tilde{W}}_{ji}$ properly form distributions along i. Note that in practice, the $Z_{i}$ ‘s in the equation above cancel out with those in Eq. 72 and 73. The expected $T_{ON}(t)$ and $T_{OFF}(t)$ are then computed using the ${\tilde{W}}_{ji}$ as

(75) $$T\left(t_{j}\right)=\sum_{i<j}{\tilde{W}}_{ji}\left(t_{j}-t_{i}\right),$$

where the appropriate residence state $\left(n\in {𝒱}_{r}\right)$ and absorbing state $\left(n\in {𝒱}_{a}\right)$ have to be used to determine the ${\tilde{W}}_{ji}$. Thus, Eq. 75 allows us to computationally predict the expected $T_{ON}(t)$ and $T_{OFF}(t)$, while accounting for discrete and finite number of time points time.

##### e. Validation using synthetic data.

To validate our Bayesian deconvolution and burst calling approaches, we tested our method on synthetic data mimicking experimental conditions. In essence, we used a 2-state model to generate single-gene copy initiation events I given a set of input parameters $\Theta =\left\{k,k_{ON},k_{OFF}\right\}$, where k is the initiation rate and $k_{ON}$ and $k_{OFF}$ are the gene switching rates. The resulting initiation events are convolved with the elongation kernel κ of the gene *hb* (see Eq. 11) to generate transcriptional activity time series G, i.e., G=κ*I. Assuming two independent sister chromatids as in real data, we sum over two independently generated transcriptional activity time series G per nucleus. Finally, we add the characterized measurement noise on the resulting activity according to Eq. 7 and 8, thus generating realistic single allele activity time series A per simulated nucleus.

###### Stationary bursting parameters.

We first investigate the performance of our method in recovering stationary bursting parameters (that do not vary in time). We generate an extensive simulated data set using the following input parameter ranges: $k\equiv K^{\left(1\right)}=8\;mRNA/min$ (consistent with real data), $k_{ON}=\eta /T$ and $k_{OFF}=(1-\eta )/T$ with $\eta \equiv P_{ON}^{\left(1\right)}\in \{0.03,\;0.12,\;0.23,\;0.36,\;0.52,\;0.78,\;0.90\}$ and $T\equiv T_{C}^{\left(1\right)}\in \left\{0.5,\;1,\;2,\;3,\;4,\;5,\;7,\;10\right\}\;min$. For each of the 56 combinations of input parameters $\left\{K^{\left(1\right)},T_{C}^{\left(1\right)},P_{ON}^{\left(1\right)}\right\}$, we generate 200 alleles and 50 min long activity time series A at 10 s intervals (as in real data). For each allele, the underlying initiation events of the 2-state model are sampled using the Gillespie algorithm [73].

We next perform single-allele deconvolution and burst calling (see Sections V A and V D) to estimate the effective mean transcription parameters (R, K, $P_{ON}$, $T_{ON}$, $T_{OFF}$ and $\left.T_{C}\right)$ for each simulated set of alleles. As an example, we show both the predicted parameters based on input (fig. S9A–B top, also see VD0d) and the resulting estimated parameters (fig. S9A–B bottom, also see V 0 b) for T=2min as a function of time and $P_{ON}$ (color coded). Overall, the estimated parameters (bottom) mimic the predicted ones (top) well, albeit with fluctuations around the mean, which is expected from a finite-sized sample (200 alleles).

We observe that the parameters $T_{ON}$ and $T_{OFF}$ are affected by different biases (fig. S9B): 1) bias due to censoring that lead to underestimation and the bending observed near the beginning and the end of the time interval, 2) bias due to missing short ON/OFF periods compared to the sampling time leading to overestimation for small values of $T_{ON}$ and $T_{OFF}$, 3) bias due to the finite length of the recording leading to underestimation for values of $T_{ON}$ and $T_{OFF}$ that are close or larger than the recording duration. Nevertheless, the estimates for $T_{ON}$ and $T_{OFF}$ remain good for the range of probed values. Interestingly, biases on $T_{C}$ are rather limited, whereas fluctuations seem larger than observed for $T_{ON}$ and $T_{OFF}$.

To fully characterize the errors on our estimates, we compare the estimated effective bursting parameters to the predicted ones for all simulated data sets as a function of the burst calling parameters w and $r_{b}$ (see Eqn. 43). Starting with our default burst calling parameters, i.e. w=1u and $r_{b}=2/u$, where u=5Δt=5/6min, we compute the median (over time) and 68% confidence intervals of the estimated bursting parameters for each data set (i.e., a combination of input parameters). Comparing the median bursting parameters with the predicted ones for all data sets (see fig. S9C) demonstrates excellent agreement between the two quantities for a large range of input $P_{ON}^{\left(1\right)}\in [0.02,\;0.90]$ and input $T_{C}^{\left(1\right)}\in \left[0.5,\;10\right]\;min$. We quantified the median relative error between the estimated and predicted parameters, both for each individual parameter and globally, as a function of the input $T_{C}^{\left(1\right)}$ (see fig. S9D). Although a fixed moving window of size w=1u was used to call bursts, our approach still recovers bursting parameters properly (global median relative error < 31%) over a rather large range of correlation times $\left(T_{C}∼0.5-8\;min\right.$) that encompasses the values estimated from real data ($T_{C}∼1-2\;min$). Thus, our choice of burst calling parameters did not prevent us from detecting possibly smaller or larger values of switching correlation times than observed in real data (see Fig. 3). Lastly, we investigate the impact of different values of the burst calling parameters w and $r_{b}$ on the global median relative error (see fig. S9D). This analysis shows that our default choice of burst calling parameters leads to the lowest global relative error over the probed burst calling parameters.

###### Time-dependent bursting parameters.

We assess the performance of our method for estimating time-dependent bursting parameters in a realistic setting by generating a data set that mimics the spatiotemporal transcriptional output of *hb* in NC14 within x/L∼0.20-0.46. The corresponding time-dependent input bursting parameters for 18 virtual AP bins (color coded) are displayed in fig. S10E–F top. For each virtual position, we generate 200 alleles and 50 min long activity time series A at 10 s intervals (as in real data). Instead of sampling the initiation times series I given the 2-state model using a time-dependent Gillespie algorithm, we use the propagator (Eq. 66) to sample both the allele state n and the resulting initiation events g at fixed and short time interval such that $I_{j}=g\left(t_{j}\right)$.

To compute the full propagator of the system, we expand the previously defined state transition matrix (Eq. 65) to also include the initiation process. The new state transition matrix ${\overset{ˆ}{M}}^{\prime }(t)$ can be written using tensor products:

(76) $${\overset{ˆ}{M}}^{\prime }\left(t\right)={\overset{ˆ}{I}}_{g}⊗{\overset{ˆ}{M}}_{2}\left(t\right)+{\overset{ˆ}{K}}_{g}\left(t\right)⊗{\overset{ˆ}{R}}_{2},$$

where ${\overset{ˆ}{I}}_{g}$ is the identity matrix of size $N_{g}$, ${\overset{ˆ}{M}}_{2}(t)$ is the transition matrix of the allele state for two independent sister chromatids (see Eq. 65), ${\overset{ˆ}{K}}_{g}(t)$ is a square matrix of size $N_{g}$ that describes the initiation of transcripts and is given by

$${\overset{ˆ}{K}}_{g}\left(t\right)=\left(\begin{array}{ccccc} -k\left(t\right) & 0 & \cdots & \cdots & 0\\ k\left(t\right) & -k\left(t\right) & \ddots & \ddots & \vdots \\ 0 & k\left(t\right) & \ddots & \ddots & \vdots \\ \vdots & \ddots & \ddots & -k\left(t\right) & 0\\ 0 & \ldots & 0 & k\left(t\right) & -k\left(t\right) \end{array}\right),$$

where $k\equiv K^{\left(1\right)}$ corresponds to the single gene copy (SGC) initiation rate, and lastly the matrix ${\overset{ˆ}{R}}_{2}$ indicates (based on the allele state) how many gene copies participate in the initiation process:

$${\overset{ˆ}{R}}_{2}=\left(\begin{array}{lll} 0 & 0 & 0\\ 0 & 1 & 0\\ 0 & 0 & 2 \end{array}\right).$$

Given the expanded state transition matrix (Eq. 76), we compute the complete time-dependent propagator of the system using the trapezoidal rule:

(77) $$\overset{ˆ}{U}\left(t_{j+1},t_{j}\right)\approx \exp\left(\frac{1}{2}\left({\overset{ˆ}{M}}^{\prime }\left(t_{j+1}\right)+{\overset{ˆ}{M}}^{\prime }\left(t_{j}\right)\right)\Delta t^{\prime }\right).$$

For accurate sampling, we set $t_{j+1}-t_{j}=\Delta t^{\prime }$ to the minimal discretized time interval $\Delta t^{\prime }=f/\left(2K_{\text{elo}}\right)=1$ s given by the Pol II footprint of f=60bp and the measured elongation rate $K_{\text{elo}}=1.8\;kb/min$. In addition, we set $N_{g}=6$, which is a safe cutoff given the probed input parameters and the small $\Delta t^{\prime }$.

Once the propagator has been computed (Eq. 77), sampling the allele state and the initiation events becomes straightforward. Given a specific allele state $n\left(t_{j}\right)\in \{0,\;1,\;2\}$ at time $t_{j}$, we define the expanded state $\lambda =n\left(t_{j}\right)N_{g}+1$ and the corresponding zero vector $\Lambda \left(t_{j}\right)$ of size $3N_{g}$, whose λ-th entry has value 1. The vector $\Lambda \left(t_{j}\right)$ and the propagator enables computing the probability $P_{\lambda^{\prime }}\left(t_{j+1}\right)$ to find the system in any state $\lambda^{\prime }=n\left(t_{j+1}\right)N_{g}+g\left(t_{j+1}\right)+1\in \left\{1,2,\ldots ,3N_{g}\right\}$ at time $t_{j+1}=t_{i}+\Delta t^{\prime }$, where $g\left(t_{j+1}\right)\in \left\{0,\;1,\ldots ,N_{g}-1\right\}$ is the number of initiation events during the time interval $\Delta t^{\prime }$. We compute the vector $P\left(t_{j+1}\right)$ whose entries are the probability $P_{\lambda^{\prime }}\left(t_{j+1}\right)$ as

(78) $$P\left(t_{j+1}\right)=\overset{ˆ}{U}\left(t_{j+1},t_{j}\right)\Lambda \left(t_{j}\right).$$

Using $P\left(t_{j+1}\right)$, we sample the state $\lambda^{\prime }$ at time $t_{j+1}$ by drawing a single random number $\lambda^{\prime }\in \left\{1,\;2,\ldots ,3N_{g}\right\}$ according to $P_{\lambda^{\prime }}\left(t_{j+1}\right)$. The corresponding new allele state is then given by $n\left(t_{j+1}\right)=\left\lfloor \left(\lambda^{\prime }-1\right)/N_{g}\right\rfloor$ and the number of initiation events during $\Delta t^{\prime }$ by $g\left(t_{j+1}\right)=\left(\lambda^{\prime }-1\right)\;mod{\;N}_{g}$. Setting the new state vector $\Lambda \left(t_{j+1}\right)$ as the zero vector whose $\lambda^{\prime \prime }$-th entry is set to 1, with $\lambda^{\prime \prime }=n\left(t_{j+1}\right)N_{g}+1$, defines an iterative procedure to generate times series according to the 2-state model with time-dependent input parameters. Specifically, this approach enables sampling single initiation time series I such that $I_{j}=g\left(t_{j}\right)$, i.e., the number of initiation events during the time interval $\left(t_{j_{1}},t_{j}\right]$. Given the short interval $\Delta t^{\prime }=1\;s$ and the probed value of the initiation rate k≤8mRNA/min, the sampled number of initiation events $g\left(t_{j}\right)$ is usually comprised between 0 and 1, and almost never exceeds 2.

Capitalizing on the sampling procedure described above, we generate 200 initiation time series I and the corresponding activity time series A at 10 s intervals for each virtual position. We first compared the estimated time-dependent bursting parameters with the predicted ones (fig. S10A–D). The temporal changes in effective bursting parameters are recovered well (fig. S10A–B). Indeed, both the predicted and estimated parameters are highly correlated (fig. S10C–D). We further estimate the single gene copy (SGC) input parameters from the estimated effective ones using the relationships derived in Section V D 0 c “Sister chromatids and single copy parameters”. Even though the SGC input parameters are deeply buried within the data, our deconvolution and burst calling approaches lead to a well-behaved time-dependent estimation of the latter (fig. S10E–H). Albeit with some distortions, we recover the input parameters adequately, confirming our ability to estimate bursting parameters in a real-case scenario.

#### E. Exploring the generality of bursting rules.

We test the validity of our derived bursting relationships beyond the *Drosophila* gap genes. To this end, we extract bursting parameters from four other studies performed in different laboratories on different genes and systems.

##### a. Drosophila *data*.

While our study mainly focusses on the *Drosophila* gap genes, two other studies performed in early *Drosophila* embryos (i.e., one on dorsoventral genes [16] and another one on synthetic core promoter modifications [21]) generated a similar set of bursting parameters that allow for a test of our established rules. For both studies, we assume that the estimated parameters are effective ones (for two indistinguishable sister chromatids) and not single gene copy parameters.

From the first study (Figure 5D in [16]), we recover the ON-probability (occupancy) $P_{ON}$, the initiation rate (loading rate) K, and the burst frequency F for *ush*-wt and st2-*dpp* for the ten positions along the dorsoventral axis considered in that study. We calculate the mean ON-time $T_{ON}=P_{ON}/F$, the mean OFF-time $T_{OFF}=\left(1-P_{ON}\right)/F$, and the switching correlation time and plot the resulting parameters in yellow in Fig. 5A.

From the second study [21], we directly use the estimated parameters ($T_{OFF}$, $T_{ON}$, K and $\left.P_{ON}\right)$ for the 2 -state model provided in Supplementary Table 2. These correspond to the following seven constructs: snaE > *sna* >24xMS2-y, snaE > *snaTATAlight* >24xMS-y, snaE >*snaTATAmut* >24xMS2-y, snaE > *snaTATAlight+INR* >24xMS2-y, snaE>*kr*-INR1 >24xMS2-y, snaE >*kr*-INR2 >24xMS2-y and snaE > *Ilp4*-*INR* >24xMS2-y. The resulting seven data points are plotted in pink in Fig. 5A. We did not use the estimated parameters for the 3 -state model due to some arbitrariness in the potential definitions of an equivalent 2-state $T_{OFF}$ in that particular model.

##### b. Yeast data.

We test estimated parameters in a recent study using yeast genes [19]. We recover the mean ON-time (burst duration) $T_{ON}$ and the mean OFF-time (time between bursts) $T_{OFF}$ for *GAL10* under multiple conditions from their Extended Data Fig. 6. We calculated the ON-probability assuming steady state as $P_{ON}=T_{ON}/\left(T_{ON}+T_{OFF}\right)$. We determine the initiation rate K from the uncalibrated Burst intensity $\tilde{K}$. To this end, we estimate the residence time of nascent mRNA on *GAL10* from the estimated genomic gene length (2200bp+862bp for the PP7 cassette) and the estimated elongation rate of 3.9 kb/min[14]. As there might be some retention of nascent transcripts at the transcription site, we round up the gene length to 3.9 kb, leading to a residence time for nascent mRNAs of approximately 1 min. We use smFISH analysis results (in triplicate) of the -RSC (DMSO) experiment [19] to calibrate the burst intensity by calculating the mean number of nascent transcripts on the gene. With a conservative definition of transcription sites having at least 5 nascent transcripts, we obtain on average 13.2 ± 0.2 nascent transcripts. Since the residence time of nascent mRNA on this yeast gene is short 1 min compared to $T_{ON}∼1.7\;min$, we assume as a first approximation that the mean number of nascent transcripts corresponds only to the ON period. We thus estimate the initiation rate as $K=13.2\tilde{K}/\langle \tilde{K}\rangle$, which is a lower bound on the true initiation rate. We plot the resulting parameters for the 42 different conditions as orange circles in Fig. 5A. While these yeast parameters are extracted from a single gene copy system, the difference between effective and single gene copy is not very pronounced below $P_{ON}<5$ (see Section V D 0 c).

##### c. Human data.

We re-analyzed single-cell transcriptional time traces of eleven endogenous human genes obtained through live imaging of a MS2 reporter [9, 45]. Two key reasons motivated such an analysis: 1) the definition of the ON period in the original studies is different from ours, conflating both the mRNA dwell-time (resulting from elongation, cleavage and termination) with the promoter ON-time; 2) the fitted generalized transcription model relies on different assumptions (3 promoter states, maximum number of nascent mRNA between 2 and 5, non-trivial dwell-time distribution due to early intron ejection and initiation prevented by early PolII engagement), which make the mapping of their parameters onto ours difficult. Thus, we chose to apply our deconvolution method on the original data and reconstruct the initiation events, allowing us to estimate bursting parameters whose definition are identical to ours.

The single-cell time traces from the original studies were obtained using a 24×MS2 stem-loops cassette inserted either in the 3’UTR (*TFF1*, [45]) or within introns (10 other genes, [9]). The fluorescent signal resulted from MCP-GFP imaged using a confocal microscope (Zeiss LSM780), with a temporal resolution of Δt=100s. The original dataset comprised 35 to 123 cells per gene, whose time traces last on average 11.5 hours. As these human genes are lowly expressed, estimating the fluorescent background in the data was straightforward. Indeed, only a few bursts are noticeable in each single-cell time trace and the majority of the time points correspond to background fluctuations. We thus computed the histogram of intensities for each cell, resulting in a mixture of a Gaussian (background fluctuation) and a heavy-tailed distribution (nascent mRNA counts plus noise). We kernel smoothed the resulting distributions to robustly estimate the mode and the 2.5^th^ percentile. For each cell, we defined the mean of the background $\mu_{b}$ as the mode of the mixture and the standard deviation of the background $\sigma_{b}$ as the half difference between the mode and 2.5^th^ percentile. We checked the consistency of our assessment of the background distribution across all cells for a given gene, noticing only a very limited number of outlier time traces corresponding to highly active allele. For these traces, we replaced $\mu_{b}$ and $\sigma_{b}$ by $med\left(\mu_{b}\right)$ and $med\left(\sigma_{b}\right)$, the median over all cells of the corresponding gene. Lastly, we subtracted the cell specific background $\mu_{b}$ to the intensities of all traces, such that in absence of transcription the signal is centered around the true zero.

Next, we calibrated the fluorescent signal in absolute units, i.e., nascent mRNA counts. We used the gene *TFF1* as nascent mRNA counts were characterized by smFISH (see Fig. S2C–D in [45]). Importantly, these smFISH distributions do not include non transcribing alleles (as with dual color smFISH, the detection of transcription sites necessitates intron signal). Thus, to match the histogram of live fluorescent intensities with the smFISH counts for calibration, one needs to account for the unknown fraction of non-transcribing alleles. We fitted the live histogram of intensities (maximum likelihood) with a mixture distribution composed of Gaussian background noise $𝒩\left(s|\mu ,\sigma_{b}\right)$ and smFISH mRNA count distribution P(g), which is given by

$$P\left(s|\rho ,\alpha \right)=\frac{1}{Z}\left(\rho 𝒩\left(s|0,\sigma_{b}\right)+\left(1-\rho \right)\sum_{g=1}^{\infty }\;𝒩\left(s|\alpha g,\sigma_{b}\right)P\left(g\right)\right),$$

where s is the live intensity, ρ∈[0,1] the fraction of time nascent mRNA are present at the locus, and α∈[0,+∞) the calibration factor (i.e., the live intensity of a single nascent mRNA). Z is the normalization constant such that ∫P(s)ds=1 and P(g) is normalized such that $\sum_{g=1}^{\infty }\;P(g)=1$ (thus excluding P(g=0) corresponding to the unknown fraction of non-transcribing alleles). By maximizing the likelihood above for the TFF1 data, we obtained ρ=0.81 and α=38. We managed to calibrate the other genes in absence of available smFISH data by extrapolation. We first noticed that $med\left(\sigma_{b}\right)$ (median over all cells for a given gene) scales linearly with med $\left(\mu_{b}\right)$ across genes and over a 2-fold range $\left(R^{2}=0.96\right)$, which points mainly to a gain difference between datasets. As the different genes were imaged using the same MCP-GFP in the same condition, the background fluctuations should in principle be the same. We thus rescaled the background subtracted signal of the other 10 genes using med $\left(\sigma_{b}\right)/\left(\alpha med\left(\sigma_{b}^{TFF1}\right)\right)$, giving us absolute units for all the dataset.

In order to deconvolve the single-cell time traces, we first need to characterize the gene-specific mRNA dwell-time and assess measurements noise. For each gene, we computed the auto-correlation (AC) function using all the time traces. From the AC (at lag τ=0), we estimated the fraction of uncorrelated variability $\sigma_{\text{img}}^{2}/\sigma^{2}$, where $\sigma^{2}$ is the total variance (Table S2, see Fig. S1D for comparison with our data). Measurements noise represents 34% of total variability on average (5% in ours). Given these numbers, we extrapolated a simple measurement noise model in absolute units, i.e., $\sigma (g)=\sqrt{\sigma_{b}^{2}+\beta g}$, with $\sigma_{b}=0.5$ and β=0.38. This noise model includes Poisson shot noise as in ours (see Equ. 7) and complies with the single mRNA sensitivity claimed in the original studies (though no dual color control were available to us to verify it).

Regarding the correlated component of the AC (for lag τ>0), it is well approximated by a single exponential that decays with the lag τ over a timescale $T_{d}$. We thus fitted these AC functions with an exponential estimating $T_{d}$ for each gene (Table S2). Notably, these time scales are much larger than what we observed in flies (6-fold larger on average). For the gap genes, we know that the mRNA dwell-time is rather small (∼2 min), due to the rapid elongation of short mRNAs (see Section V A 0 f and Table S1) and their small retention time at the locus [23]. On the other hand, it has been demonstrated in the original studies [9, 45] that the mRNA-dwell time of these human genes is rather long (typically around 10 min, even for short mRNAs), due to a combination of transcription elongation, splicing and termination. In accordance with this observation, we assumed that the measured $T_{d}$ for each gene mainly correspond to mRNA dwell-times, thus setting an upper bound on possible switching correlation time (see Sec. V C 0 c and Equ. 39).

Having calibrated the data and characterized both the measurement noise and the mRNA dwell-time, we proceeded to re-analyzed all the transcriptional time traces according to our deconvolution approach (see Section V A). We assumed a squared elongation kernel whose duration was set by $T_{d}$ (Table S2), thus neglecting the discrete effect of the individual MS2 loops. Indeed, the whole MS2 sequence length is ∼1.4 kb long, and thus transcribed in 0.7 min $\left(K_{\text{elo}}=2\;kb/min\right.$ in humans [9], which is negligible compared to the total dwell-time. To sample the initiation events, we used $\Delta t^{\prime }=10\;s$ (instead of $\Delta t^{\prime }=1$ for our data), which was small enough given the temporal resolution (Δt=100s) and the maximal amount of nascent mRNAs (∼10). Once the initiation events have been reconstructed, we performed our burst calling routine (see Section VD). For the burst calling, we set the averaging window to w=3Δt=300s and $g_{b}=1$. The window w is roughly half the average dwell-time (∼10 min), which is the limit of what we can reliably detect based on simulated data (Fig. S9). The threshold $g_{b}$ corresponds to one mRNA, consistent with their claimed single mRNA sensitivity [9, 45]. We then estimated the bursting parameters as described in Section VD0b. For individual time points, the bursting parameter relative errors is on average 6 -fold larger than for our data (27% vs 4% relative error on average). However, since these human genes are at steady state (their bursting parameters are stable in time), we only report the time averaged parameters. These parameters are given in Table S3 and S4, and shown in Fig. 5.

##### d. Mouse scRNA-seq data

We reanalyzed single-cell RNA-seq data from mouse fibroblasts and embryonic stem cells [22]. We followed a very similar approach as in the original study to estimate bursting parameters from UMI (unique molecular identifier) counts. Namely, we fitted the steady state distribution of UMI counts for each gene through maximum likelihood, using a Beta-Poisson distribution (the steady state distribution of the 2-state model of transcription). We also used mRNA lifetime estimates [75] to get physical time units. Unlike the original study, we explicitly imposed a prior on the switching correlation time $T_{C}$, effectively regularizing the parameter inference. Indeed, for the vast majority of genes, the empirical steady state distribution is not sufficient to determine all the three independent parameter of the 2 -state model $\left(K,P_{ON},T_{C}\right)$. Typically, using mature mRNA counts, one cannot resolve a $T_{C}$ value that is smaller than the mRNA lifetime $T_{M}$, which is of the order of a few hours $\left(\mathrm{med}\left(T_{M}\right)=5.3\;h)\right.$. To alleviate this identifiability issue, we imposed a prior on $T_{C}$ such that the effective log-likelihood $log\;\tilde{ℒ}$ is given by

(79) $$\log\tilde{ℒ}\left(D|K,P_{ON},T_{C}\right)=\log ℒ\left(D|K,P_{ON},T_{C}\right)-\frac{1}{2}{\left(\frac{\log_{10}T_{C}-\mu }{\sigma }\right)}^{2},$$

where logℒ(D∣Θ) is the Beta-Poisson distribution (see [22]), and the prior parameters are set to μ=0 and σ=0.5. The prior corresponds to a Gaussian in log-space centered around $T_{C}=1\;min$, whose 95% coverage spans two order of magnitude $\left(T_{C}\in [0.1,\;10]\;min\right)$. Given the range of observed $T_{C}$ values (Fig. 5A), this prior is not strongly informative and simply enforces a plausible range compatible with observations.

For each gene, we estimated the bursting parameters K, $P_{ON}$ and $T_{C}$ by maximizing the effective likelihood (Equ. 79) on UMI counts. We used the following range for the optimization: $P_{ON}\in \left[10^{-5},1\right]$, $T_{C}\in \left[10^{-2},10^{3}\right]\;min$ and $K\in \left[10^{-2.5},10^{2.5}\right]{\;min}^{-1}$. To assess inference errors $\sigma_{\Theta }$, we bootstrapped the estimated parameters by resampling the UMI distributions. We only reported genes whose parameters satisfy $2\sigma_{\Theta }<0.5$ in log-space and $B=KT_{C}/\left(1-P_{ON}\right)>1$. This effectively removes 32% of the genes, which is comparable to the effect of the thresholding applied in the original study. We show the mean transcription rate $R=KP_{ON}$, the burst frequency $F=P_{ON}\left(1-P_{ON}\right)/T_{C}$ and the burst size B in Fig. 5B. Importantly, as in the orginal study [22], we did not attempt to correct the UMI counts for low mRNA recovery rate (typically ∼10–30% recovery rate in recent scRNA-seq). Thus, parameters such as R, $P_{ON}$ and F are very likely underestimated by a factor 3 to 10. Lastly, we assessed the impact of our prior on the maximum log-likelihood values. The median penalty imposed by our prior on the log-likelihood is 0.06, and for 80% of the genes this penalty is less than one. It turns out that the resulting best-fit distributions are barely different from the ones obtained without priors, suggesting that indeed the mRNA distributions contain very little information about $T_{C}$ when $T_{C}\ll T_{M}$ (the mRNA lifetime).

## Supplementary Material

Supplement 1

## References

[1] Lelli K. M., Slattery M., and Mann R. S., Disentangling the many layers of eukaryotic transcriptional regulation., Annual Review of Genetics 46, 43 (2012). 10.1146/annurev-genet-110711-155437 PMC4295906 22934649

[2] Cramer P., Eukaryotic transcription turns 50, Cell 179, 808 (2019). 31675494 10.1016/j.cell.2019.09.018

[3] Raj A., Peskin C. S., Tranchina D., Vargas D. Y., and Tyagi S., Stochastic mRNA synthesis in mammalian cells., PLoS biology 4, e309 (2006). 17048983 10.1371/journal.pbio.0040309 PMC1563489

[4] Chubb J. R., Trcek T., Shenoy S. M., and Singer R. H., Transcriptional Pulsing of a Developmental Gene, Current Biology 16, 1018 (2006). 16713960 10.1016/j.cub.2006.03.092 PMC4764056

[5] Zenklusen D., Larson D. R., and Singer R. H., Single-RNA counting reveals alternative modes of gene expression in yeast., Nature Structural & Molecular Biology 15, 1263 (2008). 10.1038/nsmb.1514 PMC3154325 19011635

[6] Suter D. M., Molina N., Gatfield D., Schneider K., Schibler U., and Naef F., Mammalian genes are transcribed with widely different bursting kinetics., Science 332, 472 (2011). 21415320 10.1126/science.1198817

[7] Bothma J. P., Garcia H. G., Esposito E., Schlissel G., Gregor T., and Levine M., Dynamic regulation of eve stripe 2 expression reveals transcriptional bursts in living drosophila embryos., Proc. Natl. Acad. Sci. USA 111, 10598 (2014). 24994903 10.1073/pnas.1410022111 PMC4115566

[8] Tantale K., Mueller F., Kozulic-Pirher A., Lesne A., Victor J.-M., Robert M.-C., Capozi S., Chouaib R., Bäcker V., Mateos-Langerak J., Darzacq X., Zimmer C., Basyuk E., and Bertrand E., A single-molecule view of transcription reveals convoys of RNA polymerases and multi-scale bursting, Nature Communications 7, 12248 (2016). 10.1038/ncomms12248 PMC4974459 27461529

[9] Wan Y., Anastasakis D. G., Rodriguez J., Palangat M., Gudla P., Zaki G., Tandon M., Pegoraro G., Chow C. C., Hafner M., and Larson D. R., Dynamic imaging of nascent rna reveals general principles of transcription dynamics and stochastic splice site selection., Cell 184, 2878 (2021). 33979654 10.1016/j.cell.2021.04.012 PMC8183334

[10] Senecal A., Munsky B., Proux F., Ly N., Braye F., Zimmer C., Mueller F., and Darzacq X., Transcription Factors Modulate c-Fos Transcriptional Bursts, Cell Reports 8, 75 (2014). 24981864 10.1016/j.celrep.2014.05.053 PMC5555219

[11] Bartman C. R., Hsu S. C., Hsiung C. C.-S., Raj A., and Blobel G. A., Enhancer Regulation of Transcriptional Bursting Parameters Revealed by Forced Chromatin Looping., Molecular Cell 62, 237 (2016). 27067601 10.1016/j.molcel.2016.03.007 PMC4842148

[12] Li C., Cesbron F., Oehler M., Brunner M., and Höfer T., Frequency Modulation of Transcriptional Bursting Enables Sensitive and Rapid Gene Regulation, Cell Systems 6, 409 (2018). 29454937 10.1016/j.cels.2018.01.012

[13] Nicolas D., Zoller B., Suter D. M., and Naef F., Modulation of transcriptional burst frequency by histone acetylation, Proc. Natl. Acad. Sci. USA 115, 201722330 (2018). 10.1073/pnas.1722330115 PMC6142243 29915087

[14] Donovan B. T., Huynh A., Ball D. A., Patel H. P., Poirier M. G., Larson D. R., Ferguson M. L., and Lenstra T. L., Live-cell imaging reveals the interplay between transcription factors, nucleosomes, and bursting., The EMBO Journal 38, 10.15252/embj.2018100809 (2019). PMC6576174 31101674

[15] Falo-Sanjuan J., Lammers N. C., Garcia H. G., and Bray S. J., Enhancer Priming Enables Fast and Sustained Transcriptional Responses to Notch Signaling, Developmental Cell 50, 411 (2019). 31378591 10.1016/j.devcel.2019.07.002 PMC6706658

[16] Hoppe C., Bowles J. R., Minchington T. G., Sutcliffe C., Upadhyai P., Rattray M., and Ashe H. L., Modulation of the Promoter Activation Rate Dictates the Transcriptional Response to Graded BMP Signaling Levels in the Drosophila Embryo, Developmental Cell 54, 727 (2020). 32758422 10.1016/j.devcel.2020.07.007 PMC7527239

[17] Tantale K., Garcia-Oliver E., Robert M.-C., L’Hostis A., Yang Y., Tsanov N., Topno R., Gostan T., KozulicPirher A., Basu-Shrivastava M., Mukherjee K., Slaninova V., Andrau J.-C., Mueller F., Basyuk E., Radulescu O., and Bertrand E., Stochastic pausing at latent HIV-1 promoters generates transcriptional bursting, Nature Communications 12, 4503 (2021). 10.1038/s41467-021-24462-5 PMC8302722 34301927

[18] Bass V. L., Wong V. C., Bullock M. E., Gaudet S., and Miller-Jensen K., TNF stimulation primarily modulates transcriptional burst size of NF-κ B-regulated genes, Molecular Systems Biology 17, e10127 (2021). 34288498 10.15252/msb.202010127 PMC8290835

[19] Brouwer I., Kerklingh E., Leeuwen F. v, and Lenstra T. L, Dynamic epistasis analysis reveals how chromatin remodeling regulates transcriptional bursting, Nature Structural & Molecular Biology 30, 692 (2023). 10.1038/s41594-023-00981-1 PMC10191856 37127821

[20] Fukaya T., Lim B., and Levine M., Enhancer control of transcriptional bursting., Cell 166, 358 (2016). 27293191 10.1016/j.cell.2016.05.025 PMC4970759

[21] Pimmett V. L., Dejean M., Fernandez C., Trullo A., Bertrand E., Radulescu O., and Lagha M., Quantitative imaging of transcription in living Drosophila embryos reveals the impact of core promoter motifs on promoter state dynamics, Nature Communications 12, 4504 (2021). 10.1038/s41467-021-24461-6 PMC8302612 34301936

[22] Larsson A. J. M., Johnsson P., Hagemann-Jensen M., Hartmanis L., Faridani O. R., Reinius B., r. Segerstolpe, C. M. Rivera, B. Ren, and R. Sandberg, Genomic encoding of transcriptional burst kinetics, Nature 565, 251 (2018). 10.1038/s41586-018-0836-1 PMC7610481 30602787

[23] Zoller B., Little S. C., and Gregor T., Diverse Spatial Expression Patterns Emerge from Unified Kinetics of Transcriptional Bursting, Cell 175, 835 (2018). 30340044 10.1016/j.cell.2018.09.056 PMC6779125

[24] Little S., Tikhonov M., and Gregor T., Precise Developmental Gene Expression Arises from Globally Stochastic Transcriptional Activity, Cell 154, 789 (2013). 23953111 10.1016/j.cell.2013.07.025 PMC3778922

[25] Levo M., Raimundo J., Bing X. Y., Sisco Z., Batut P. J., Ryabichko S., Gregor T., and Levine M. S., Transcriptional coupling of distant regulatory genes in living embryos., Nature 605, 754 (2022). 35508662 10.1038/s41586-022-04680-7 PMC9886134

[26] Bertrand E., Chartrand P., Schaefer M., Shenoy S. M., Singer R. H., and Long R. M., Localization of ASH1 mRNA particles in living yeast., Molecular Cell 2, 437 (1998). 9809065 10.1016/s1097-2765(00)80143-4

[27] Larson D. R., Zenklusen D., Wu B., Chao J. A., and Singer R. H., Real-time observation of transcription initiation and elongation on an endogenous yeast gene., Science 332, 475 (2011). 21512033 10.1126/science.1202142 PMC3152976

[28] Garcia H. G., Tikhonov M., Lin A., and Gregor T., Quantitative imaging of transcription in living Drosophila embryos links polymerase activity to patterning., Current Biology 23, 2140 (2013). 24139738 10.1016/j.cub.2013.08.054 PMC3828032

[29] Lucas T., Ferraro T., Roelens B., Chanes J. D. L. H., Walczak A. M., Coppey M., and Dostatni N., Live imaging of bicoid-dependent transcription in drosophila embryos., Current Biology 23, 2135 (2013). 24139736 10.1016/j.cub.2013.08.053

[30] Liu J., Hansen D., Eck E., Kim Y. J., Turner M., Alamos S., and Garcia H. G., Real-time single-cell characterization of the eukaryotic transcription cycle reveals correlations between RNA initiation, elongation, and cleavage, PLoS Computational Biology 17, e1008999 (2021). 34003867 10.1371/journal.pcbi.1008999 PMC8162642

[31] Dubuis J. O., Samanta R., and Gregor T., Accurate measurements of dynamics and reproducibility in small genetic networks., Molecular Systems Biology 9, 639 (2013). 23340845 10.1038/msb.2012.72 PMC3564256

[32] Peccoud J. and Ycart B., Markovian modeling of geneproduct synthesis, Theoretical Population Biology 48, 222 (1995).

[33] Zoller B., Nicolas D., Molina N., and Naef F., Structure of silent transcription intervals and noise characteristics of mammalian genes, Molecular Systems Biology 11, 823 (2015). 26215071 10.15252/msb.20156257 PMC4547851

[34] Corrigan A. M., Tunnacliffe E., Cannon D., and Chubb J. R., A continuum model of transcriptional bursting, eLife 5, e13051 (2016). 26896676 10.7554/eLife.13051 PMC4850746

[35] Lammers N. C., Galstyan V., Reimer A., Medin S. A., Wiggins C. H., and Garcia H. G., Multimodal transcriptional control of pattern formation in embryonic development, Proc. Natl. Acad. Sci. USA 117, 836 (2020). 31882445 10.1073/pnas.1912500117 PMC6969519

[36] McKnight S. L. and Miller O. L., Post-replicative nonribosomal transcription units in D. Melanogaster embryos., Cell 17, 551 (1979) 113103 10.1016/0092-8674(79)90263-0

[37] Lagha M., Bothma J. P., and Levine M., Mechanisms of transcriptional precision in animal development, Trends in Genetics 28, 409 (2012). 22513408 10.1016/j.tig.2012.03.006 PMC4257495

[38] Schroeder M. D., Pearce M., Fak J., Fan H., Unnerstall U., Emberly E., Rajewsky N., Siggia E. D., and Gaul U., Transcriptional Control in the Segmentation Gene Network of Drosophila, PLoS Biology 2, e271 (2004). 15340490 10.1371/journal.pbio.0020271 PMC514885

[39] Perry M. W., Bothma J. P., Luu R. D., and Levine M., Precision of Hunchback Expression in the Drosophila Embryo, Current Biology 22, 2247 (2012). 23122844 10.1016/j.cub.2012.09.051 PMC4257490

[40] Berrocal A., Lammers N. C., Garcia H. G., and Eisen M. B., Kinetic sculpting of the seven stripes of the Drosophila even-skipped gene, eLife 9, e61635 (2020). 33300492 10.7554/eLife.61635 PMC7864633

[41] Berrocal A., Lammers N. C., Garcia H. G., and Eisen M. B., Unified bursting strategies in ectopic and endogenous even-skipped expression patterns, eLife 10.7554/elife.88671.1 (2023). PMC11627552 39651963

[42] Fukaya T., Dynamic regulation of anterior-posterior patterning genes in living Drosophila embryos., Current Biology 31, 2227 (2021). 33761316 10.1016/j.cub.2021.02.050

[43] Hülskamp M., Lukowitz W., Beermann A., Glaser G., and Tautz D., Differential regulation of target genes by different alleles of the segmentation gene hunchback in Drosophila., Genetics 138, 125 (1994). 8001780 10.1093/genetics/138.1.125 PMC1206124

[44] Sanchez A. and Golding I., Genetic Determinants and Cellular Constraints in Noisy Gene Expression, Science 342, 1188 (2013). 24311680 10.1126/science.1242975 PMC4045091

[45] Rodriguez J., Ren G., Day C. R., Zhao K., Chow C. C., and Larson D. R., Intrinsic dynamics of a human gene reveal the basis of expression heterogeneity., Cell 176, 213 (2019). 30554876 10.1016/j.cell.2018.11.026 PMC6331006

[46] Rayon T., Stamataki D., Perez-Carrasco R., GarciaPerez L., Barrington C., Melchionda M., Exelby K., Lazaro J., Tybulewicz V. L. J., Fisher E. M. C., and Briscoe J., Species-specific pace of development is associated with differences in protein stability, Science 369, 10.1126/science.aba7667 (2020). PMC7116327 32943498

[47] Diaz-Cuadros M., Miettinen T. P., Skinner O. S., Sheedy D., Díaz-García C. M., Gapon S., Hubaud A., Yellen G., Manalis S. R., Oldham W. M., and Pourquié O., Metabolic regulation of species-specific developmental rates, Nature 613, 550 (2023). 36599986 10.1038/s41586-022-05574-4 PMC9944513

[48] Dar R. D., Razooky B. S., Singh A., Trimeloni T. V., McCollum J. M., Cox C. D., Simpson M. L., and Weinberger L. S., Transcriptional burst frequency and burst size are equally modulated across the human genome., Proc. Natl. Acad. Sci. USA 109, 17454 (2012). 23064634 10.1073/pnas.1213530109 PMC3491463

[49] Tsai A., Muthusamy A. K., Alves M. R., Lavis L. D., Singer R. H., Stern D. L., and Crocker J., Nuclear microenvironments modulate transcription from low-affinity enhancers, eLife 6, e28975 (2017). 29095143 10.7554/eLife.28975 PMC5695909

[50] Cho W.-K., Spille J.-H., Hecht M., Lee C., Li C., Grube V., and Cisse I. I., Mediator and RNA polymerase II clusters associate in transcription-dependent condensates., Science 361, 412 (2018). 29930094 10.1126/science.aar4199 PMC6543815

[51] Li J., Hsu A., Hua Y., Wang G., Cheng L., Ochiai H., Yamamoto T., and Pertsinidis A., Single-gene imaging links genome topology, promoter–enhancer communication and transcription control, Nature Structural & Molecular Biology 27, 1032 (2020). 10.1038/s41594-020-0493-6 PMC7644657 32958948

[52] Henninger J. E., Oksuz O., Shrinivas K., Sagi I., LeRoy G., Zheng M. M., Andrews J. O., Zamudio A. V., Lazaris C., Hannett N. M., Lee T. I., Sharp P. A., Cissé I. I., Chakraborty A. K., and Young R. A., RNA-Mediated Feedback Control of Transcriptional Condensates, Cell 184, 207 (2021) 33333019 10.1016/j.cell.2020.11.030 PMC8128340

[53] Nguyen V. Q., Ranjan A., Liu S., Tang X., Ling Y. H., Wisniewski J., Mizuguchi G., Li K. Y., Jou V., Zheng Q., Lavis L. D., Lionnet T., and Wu C., Spatiotemporal coordination of transcription preinitiation complex assembly in live cells, Molecular Cell 81, 3560 (2021). 34375585 10.1016/j.molcel.2021.07.022 PMC8420877

[54] Brückner D. B., Chen H., Barinov L., Zoller B., and Gregor T., Stochastic motion and transcriptional dynamics of pairs of distal dna loci on a compacted chromosome, bioRxiv, 2023.01.18.524527 (2023). 10.1126/science.adf5568 PMC10439308 37384691

[55] Tkačik G., Callan C. G., and Bialek W., Information flow and optimization in transcriptional regulation, Proc. Natl. Acad. Sci. USA 105, 12265 (2008), 0705.0313. 10.1073/pnas.0806077105 PMC2527900 18719112

[56] Jones D. L., Brewster R. C., and Phillips R., Promoter architecture dictates cell-to-cell variability in gene expression, Science 346, 1533 (2014). 25525251 10.1126/science.1255301 PMC4388425

[57] Hausser J., Mayo A., Keren L., and Alon U., Central dogma rates and the trade-off between precision and economy in gene expression, Nature Communications 10, 68 (2018). 10.1038/s41467-018-07391-8 PMC6325141 30622246

[58] Petkova M. D., Tkačik G., Bialek W., Wieschaus E. F., and Gregor T., Optimal Decoding of Cellular Identities in a Genetic Network, Cell 176, 844 (2019). 30712870 10.1016/j.cell.2019.01.007 PMC6526179

[59] Balakrishnan R., Mori M., Segota I., Zhang Z., Aebersold R., Ludwig C., and Hwa T., Principles of gene regulation quantitatively connect DNA to RNA and proteins in bacteria, Science 378, eabk2066 (2022). 36480614 10.1126/science.abk2066 PMC9804519

[60] Becker K., Balsa-Canto E., Cicin-Sain D., Hoermann A., Janssens H., Banga J. R., and Jaeger J., Reverse-Engineering Post-Transcriptional Regulation of Gap Genes in Drosophila melanogaster, PLoS Computational Biology 9, e1003281 (2013). 24204230 10.1371/journal.pcbi.1003281 PMC3814631

[61] Rogers W. A., Goyal Y., Yamaya K., Shvartsman S. Y., and Levine M. S., Uncoupling neurogenic gene networks in the Drosophila embryo, Genes & Development 31, 634 (2017). 28428262 10.1101/gad.297150.117 PMC5411704

[62] Chen H., Levo M., Barinov L., Fujioka M., Jaynes J. B., and Gregor T., Dynamic interplay between enhancer–promoter topology and gene activity, Nature Genetics 50, 1296 (2018). 30038397 10.1038/s41588-018-0175-z PMC6119122

[63] Bothma J. P., Norstad M. R., Alamos S., and Garcia H. G., LlamaTags: A Versatile Tool to Image Transcription Factor Dynamics in Live Embryos, Cell 173, 1810 (2018). 29754814 10.1016/j.cell.2018.03.069 PMC6003873

[64] Gregor T., Wieschaus E. F., McGregor A. P., Bialek W., and Tank D. W., Stability and Nuclear Dynamics of the Bicoid Morphogen Gradient, Cell 130, 141 (2007). 17632061 10.1016/j.cell.2007.05.026 PMC2253672

[65] Liu F., Morrison A. H., and Gregor T., Dynamic interpretation of maternal inputs by the Drosophila segmentation gene network, Proceedings of the National Academy of Sciences 110, 6724 (2013). 10.1073/pnas.1220912110 PMC3637740 23580621

[66] Hayden L., Hur W., Vergassola M., and Talia S. D., Manipulating the nature of embryonic mitotic waves, Current Biology 32, 4989 (2022). 36332617 10.1016/j.cub.2022.10.014 PMC9691596

[67] deLeeuw J., Introduction to Akaike (1973) Information Theory and an Extension of the Maximum Likelihood Principle, in Breakthroughs in Statistics, Foundations and Basic Theory, Springer Series in Statistics (Springer, 1992) pp. 599–609.

[68] Andrieu C., Freitas N. D., Doucet A., and Jordan M. I., An introduction to MCMC for machine learning, Machine learning 50, 5 (2003).

[69] Andrieu C. and Thoms J., A tutorial on adaptive MCMC, Statistics and Computing 18, 343 (2008).

[70] Rosenthal J. S., Optimal Proposal Distributions and Adaptive MCMC, Handbook of Markov Chain Monte Carlo 4 (2010).

[71] Lestas I., Paulsson J., Ross N. E., and Vinnicombe G., Noise in Gene Regulatory Networks, IEEE Transactions on Automatic Control 53, 189 (2008).

[72] Walczak A. M., Mugler A., and Wiggins C. H., Computational Modeling of Signaling Networks, Methods in Molecular Biology 880, 273 (2012), 1005.2648. 10.1007/978-1-61779-833-7_13 23361990

[73] Gillespie D. T., Stochastic Simulation of Chemical Kinetics, Annual Review of Physical Chemistry 58, 35 (2007). 10.1146/annurev.physchem.58.032806.104637 17037977

[74] Sidje R. B., Expokit: a software package for computing matrix exponentials, ACM Transactions on Mathematical Software (TOMS) 24, 130 (1998).

[75] Herzog V. A., Reichholf B., Neumann T., Rescheneder P., Bhat P., Burkard T. R., Wlotzka W., Haeseler A. v, Zuber J., and Ameres S. L., Thiol-linked alkylation of RNA to assess expression dynamics, Nature Methods 14, 1198 (2017). 28945705 10.1038/nmeth.4435 PMC5712218

