# Supplementary material for: Gene activity fully predicts transcriptional bursting dynamics
Source: ArXiv. 2024 Jun 28:arXiv:2304.08770v3. Originally published 2023 Apr 18. Preprint. [Version 3] (PMC10153294)
Supplement: Supplement 1 [file NIHPP2304.08770v3-supplement-1.pdf]

SUPPLEMENTAL FIGURES

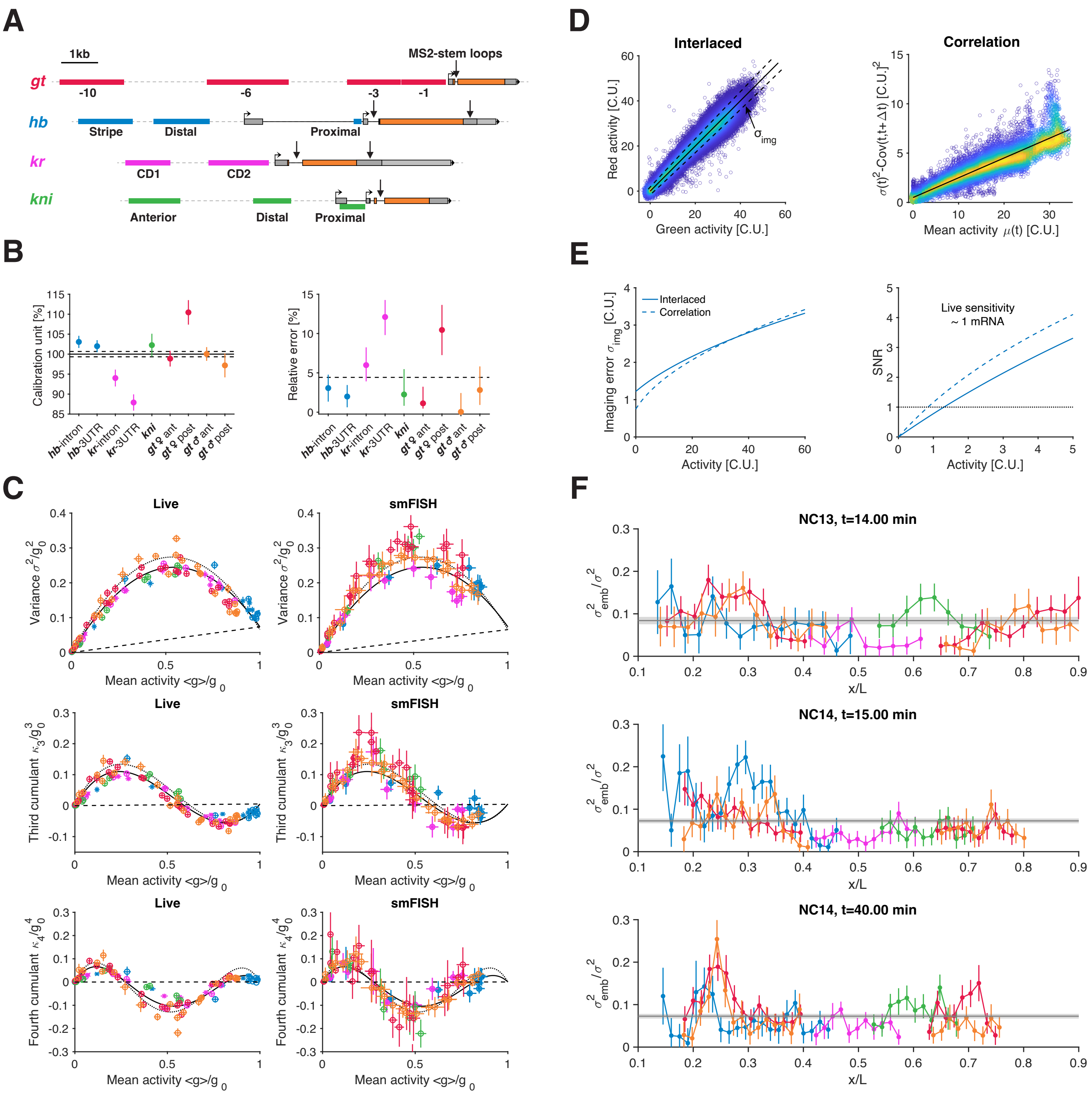

FIG. S1. **Signal calibration, measurement error and embryo-embryo variability.** [Caption see next page.]

FIG. S1. **Signal calibration, measurement error and embryo-to-embryo variability.** (A) The four trunk gap genes, *giant* (*gt*), *hunchback* (*hb*), *Kruppel* (*Kr*) and *knirps* (*kni*) were imaged using the MS2/PP7 stem-loop labeling systems. Stem-loop cassettes (vertical black arrow) were inserted either in the first/second intron or in the 3'UTR of each gene. Gap genes harbor different *cis*-architectures as characterized by the number of promoters, number of enhancers (color boxes), and composition of these *cis*-regulatory elements (TF binding motifs, core promoter elements, etc.). (B) Relative calibration unit (left) and relative error (right) for each gap gene construct related to Fig. 1C. The conversion of the live signal to absolute units is performed by comparison to a smFISH-based measurement. Calibration was performed by matching the mean full embryo length transcriptional activity profiles (reconstructed by averaging over all nuclei in 2.5% AP bins, within a 5 min time window in NC13) measured by live imaging to those previously measured with smFISH (Zoller et al., 2018). (Left) The procedure was performed using all measured gap profiles at once, leading to a final calibration unit (horizontal back line, dashed lines are plus/minus one standard error). We then repeated the procedure for each individual construct separately (color circles), and the derived units are expressed in percent of the global fit. (Right) Relative error for the calibration unit of each individual gene construct with respect to the global unit and mean relative error (dashed line), which is below 5%. Error bars are 68% confidence intervals. (C) Comparison of higher cumulants versus mean activity relationships obtained by live imaging and smFISH measurements; right column panels are reproduced from Figure 3B-D in Zoller et al. (2018), left column panels are from live data analyzed equivalently. Live cumulants of transcriptional activity (mean, variance, 3rd and 4th cumulant) are estimated over all nuclei in 2.5% AP bins, within a 5 min time window in NC13. Cumulants are converted from equivalent cytoplasmic mRNA units (C.U.) to Pol II counts for a single gene copy of average length (3.3 kb). The cumulants are normalized with respect to g_0 defined as the intercept of the Poisson background (dashed line) and the polynomial fit to the data (black solid line for live and doted for smFISH). The number g_0 can be interpreted as the mean number of Pol II on a 3.3 kb long gap gene at maximal activity. We get $g_0 = 13.6$ for live and $g_0 = 15.2$ with smFISH measurements, a difference of 12%. Overall, the higher cumulants versus mean relationships obtained from live (left column) and from smFISH (right column) are extremely close (black solid versus dotted line), confirming the quantitative nature and the proper calibration of our live assay. Two independent methods (one being non-invasive genetically but involving fixation, while the other involves gene editing and stem-loop cassette insertions) leading to the same quantitative conclusions validate each other reciprocally. It strongly suggests that our synthetic modifications of the endogenous gap gene loci have no currently measurable effect on the transcriptional output of the system. (D) Two independent methods to assess the imaging error. (Left) An interlaced cassette of alternating MS2 and PP7 stem-loops, labeled with two differently colored coat proteins (MCP-GFP and PCP-mCherry), is inserted in the first intron of *Kr*. In absence of imaging error, the transcriptional activity in the green and red channels when calibrated to C.U. should perfectly correlate (on the diagonal). We fitted the spread σ_{img} orthogonal to the diagonal (black line, slope one) to characterize the imaging error; assuming σ^2_{img} scales as $\sigma^2_b + \alpha I$ with mean intensity I, where σ^2_b is the background noise and αI a Poisson shot noise term. The resulting fit for σ_{img} is highlighted by the dashed lines (plus minus one std around the diagonal). (Right) Imaging error estimation from the single allele transcriptional time series (with the assumption that the measured transcriptional fluctuations result from the sum of uncorrelated imaging noise and correlated noise due to the elongation of tagged nascent transcripts). We computed the time-dependent mean activity $\mu(t)$, variance $\sigma^2(t)$ and covariance between consecutive time points $\text{Cov}(t, t + \Delta t)$ (where Δt is 10 s), over all nuclei within 1.5–2.5% AP bins for all measured genes. The uncorrelated imaging variability σ^2_{img} is then approximated by $\sigma(t)^2 - \text{Cov}(t, t + \Delta t)$, which is plotted as a function of $\mu(t)$ for all time points. We characterized σ^2_{img} by fitting the data with a line $\sigma^2_b + \alpha\mu$. Fitting results are shown in E. Overall, the fractional imaging variability $\sigma^2_{\text{img}}/\sigma^2$ is $\sim 5\%$. (E) Our two estimates for the imaging error (interlaced dual-color construct (solid line) and correlation-based approach (dashed line)) are consistent. The signal-to-noise-ratio (SNR), defined as μ/σ_{img}, is close to one (dotted line) when $\mu \approx 1$, indicating that the sensitivity of our live measurements is close to one mRNA molecule. (F) Fractional embryo variability profiles as a function of AP position and developmental time, for all gap genes. We define embryo variability σ^2_{emb} as the variance of the mean activity across embryos, and report the fractional embryo variability as the ratio $\sigma^2_{\text{emb}}/\sigma^2$, where $\sigma^2 = \sigma^2_{\text{emb}} + \sigma^2_{\text{img}} + \sigma^2_{\text{nuc}}$ is the total variance, and σ^2_{nuc} corresponds to the transcriptional allele-to-allele noise across nuclei. Overall, the fractional embryo variability $\sigma^2_{\text{emb}}/\sigma^2$ is $\sim 10\%$, meaning that most of the variability arises from σ^2_{nuc}. Thus, together D, E, and F show that $\sigma^2 = +\sigma^2_{\text{emb}} + \sigma^2_{\text{img}} + \sigma^2_{\text{nuc}}$ is a good proxy for σ^2_{nuc}, which is the relevant noise contribution that contains all the bursting phenomenology.

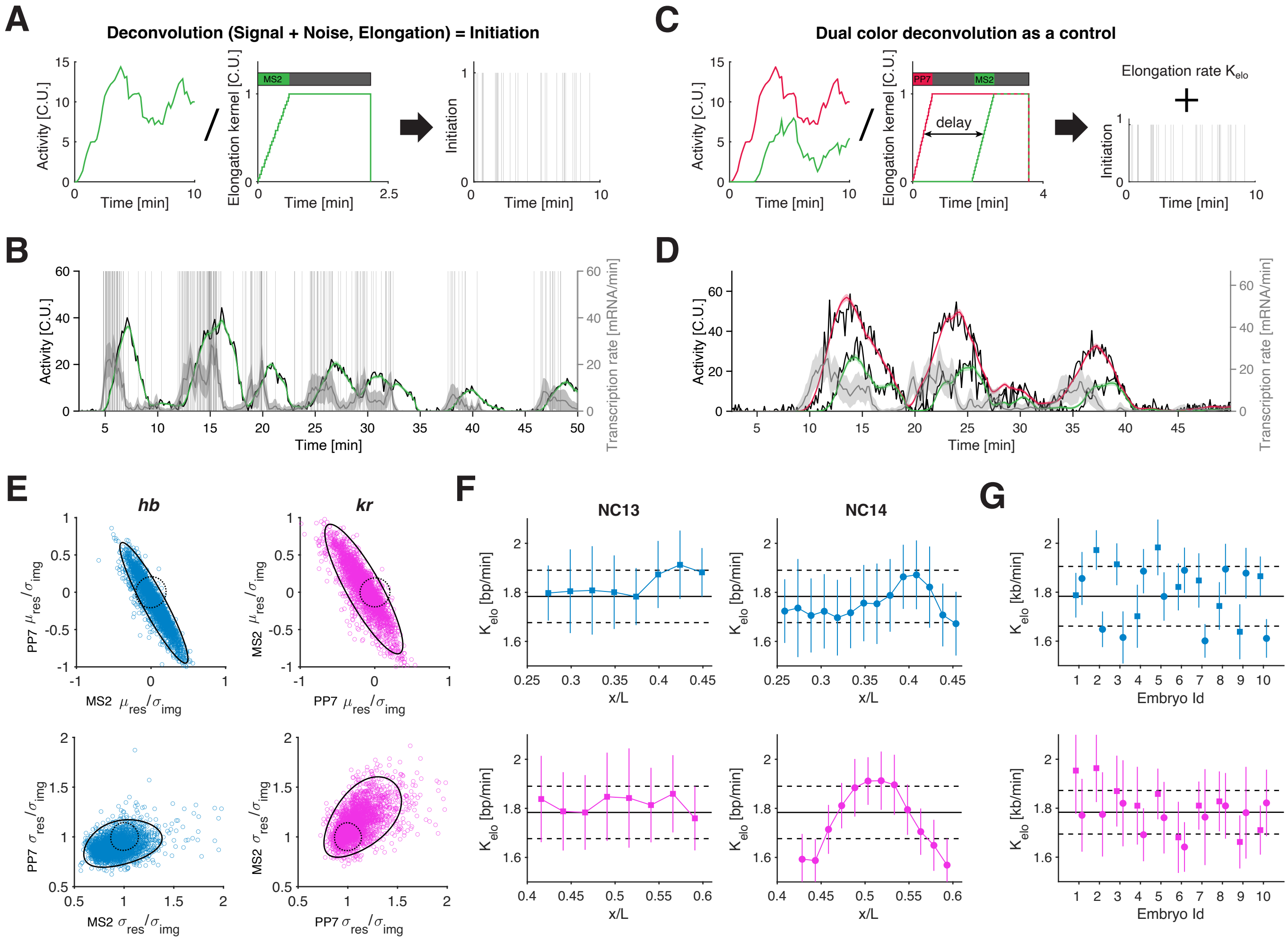

FIG. S2. **Dual color measurements to validate single-cell deconvolution and measure elongation rate.** [Caption see next page.]

FIG. S2. **Dual color measurements to validate single-cell deconvolution and measure elongation rate.** (A) Reconstruction of transcription initiation events from deconvolution of single allele transcription time series. The signal is modeled as a convolution between transcription initiation events and a kernel accounting for the elongation of a single Pol II through the MS2 cassette and the gene body (using an elongation rate $K_{\mathrm{elo}} = 1.8\,\mathrm{kb/min}$, see D-E). Bayesian deconvolution is performed by sampling from the posterior distribution of possible configuration of initiation events given the measured activity and measurement noise (Fig. S1F-G). (B) Example deconvolved initiation configuration (vertical gray bars) and corresponding reconstructed signal (green) from a single allele transcription time series (black). Single allele transcription rate (gray line) is estimated by counting the number of initiation events within 10 s intervals for a given sampled configuration and averaged over 1'000 of such configurations. The displayed solid line and envelope for transcription rate (gray) and reconstructed signal (green) correspond to the mean and one standard deviation of the posterior distribution. (C) Validation of the kernel assumption for the deconvolution of initiation events from single allele transcription time series using a dual-color (confocal) imaging approach for *hb* and *Kr*. For *hb* (*Kr*), we generated fly lines with dual insertions of an MS2 (PP7) stem-loop cassette in the respective first intron and a PP7 (MS2) stem-loop cassette in the 3'UTR. In both cases, the two cassettes were labeled using two different colors (MCP-GFP green and PCP-mCherry red). Since the two signals are correlated through the elongation process, the simultaneously measured pair of time series has a further constrained set of underlying initiation configurations and represents thus a good test for the approach. To deconvolve single allele dual color time series together (i.e., a single train of polymerases needs to match two signals), using two kernels modeling each loop-cassette location and satisfying our key assumptions (i. constant and deterministic elongation rate; ii. no Pol II pausing/dropping in gene body; iii absence of co-transcriptional splicing; iv. fast termination). In addition, the dual-color strategy allows estimation of the average elongation rate from the overall delay between the two signals (using the known genomic distance between the MS2 and PP7 insertion sites). (D) Dual-color signal reconstructed from deconvolved single allele transcription time series (black lines for raw measured data). single allele transcription rate (gray line with one std envelope) is deconvolved from the single depicted pair of measured time series (black lines). The signal (red and green lines with one std envelope) is devoid of imaging noise (as it was modeled from Fig. S1D during the deconvolution process) and is reconstructed by convolving back the resulting transcription rate with the kernel of each channel. Qualitatively, the signal (color) matches well (see E) the measured time series (black) in strong support of our kernel assumptions. (E) Distribution of residuals from the dual-color reconstruction. We quantified the mean and standard deviation of the normalized residuals, i.e., of the difference between the measured signal (black in D) and the reconstructed signal (color in D) divided by the standard deviation of the imaging noise, for each recorded individual allele (for *hb* $N = 2666$ (blue) and for *Kr* $N = 2594$ (pink)). Overall, the dispersion of the means and standard deviations of normalized residuals (black line, 95% confidence ellipse) is close to the expected dispersion of a perfect model (dotted line, 95% confidence ellipse). (F-G) Estimated elongation rate K_{elo} from dual-color measurements. (F) Average elongation rate computed over nuclei across 10 embryos as a function AP position (both *hb* (blue) and *Kr* (pink)) in NC13 (square) and NC14 (circle), with error bars representing one standard deviation across the embryo means. (G) Average elongation rate was computed for individual embryos (color code and symbols as F), with error bars representing the standard deviation across the means over positions. The elongation rate is globally conserved across genes and nuclear cycles, with $K_{\mathrm{elo}} = 1.8 \pm 0.1$ kb/min (corresponding to the mean across embryos (black line) plus/minus one standard deviation (dashed line).

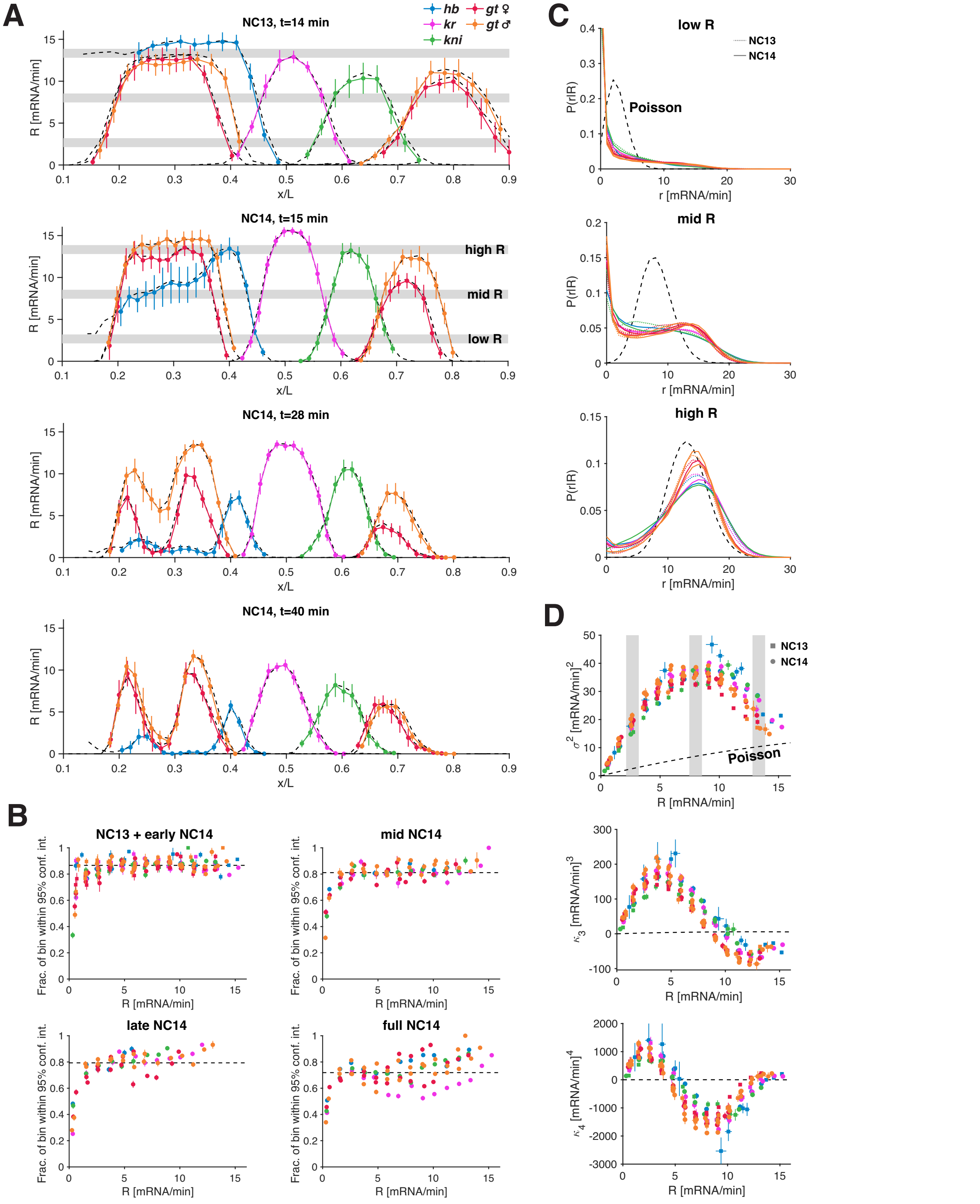

FIG. S3. **Single-allele transcription rate distributions reveal common bursting characteristics.** [Caption see next page.]

FIG. S3. **Single-allele transcription rate distributions reveal common bursting characteristics.** (A) Snapshots of the gap gene mean transcription rate R as a function of AP position in late NC13, as well as early, mid and late NC14 (time t min after mitosis). Gap gene profiles (color) are obtained by averaging the deconvolved single allele transcription rate over all nuclei within each AP bin (width of 2.5% and 1.5% embryo egg length in NC13 and NC14 respectively) and at each time point (10 s temporal resolution). The black dashed lines correspond to the mean activity (Fig. 1C) of each gap gene at the same position and time normalized by the effective elongation time (see Methods, Fig. S4A). Both the colored and dashed profiles agree, justifying our deconvolution approach. Error bars are one standard deviation across embryo means. Overall, we have effectively deconvolved $N_g = 7$ "genes" ($4{+}gt$ male and female and anterior and posterior regions), over $N_t = 362$ time points (NC13+NC14), across N_x =9–18 positions, leading to a total of $33'214$ bins, each averaging ~ 200 nuclei (with a single allele per nucleus). Strikingly, the gap genes reach a similar maximum average transcription rate $R_{\max} = 14.8 \pm 0.9$ mRNA/min. (B) Fraction of spatial and temporal bins whose single allele transcription rate distribution $P(r)$ is consistent with the conditional transcription rate distribution $P(r|R)$ determined by pooling nuclei over multiple bins at a given mean transcription rate R (see C). We computed the 95% confidence interval on the cumulative distribution of $P(r|R)$ and checked for all the underlying bins at a given R whether their individual cumulative distribution was within the overall confidence interval. We repeated this process for four distinct developmental time windows: NC13 ($6.5 \leq t$ min after mitosis) plus early NC14 ($7.5 \leq t < 20.5$ min), mid NC14 ($20.5 \leq t < 34.5$ min), late NC14 ($34.5 \leq t < 48$ min), and a wider NC14 window ($7.5 \leq t < 48$ min). Overall, bins that share similar R within the same time window have very similar $P(r)$ distribution (median given by dashed line over 80%), which justifies the pooling of these bins. On the other hand, when pooling bins over the whole NC14 we observe further dissimilarities between bins, suggesting that $P(r|R)$ might moderately change over time. (C) Distribution $P(r|R)$ of single allele transcription rates estimated within 1-min-intervals in both NC13 (color dotted lines) and early NC14 (color solid lines). These distributions are computed over all the nuclei from time points and AP bins whose mean transcription rate R corresponds either to a low $[2.1, 3.2]$, mid $[7.5, 8.5]$ or high transcription level $[12.8, 13.9]$ (as gray shade in A and D). The various gap gene distributions collapse at all transcription levels indicating an underlying common mode of transcription. Furthermore, these distributions differ from the Poisson distribution (black dashed line), which is the expected distribution for a constitutive regime (in which the gene is continuously active and always ON). The difference is most-pronounced for low- to mid-levels of R, where the gap distributions highlight two modes (instead of one for Poisson): a large probability mass around zero suggesting an abundance of non-transcribing or barely transcribing alleles and an enrichment of highly transcribing alleles beyond the Poisson expectation. Such features are highly suggestive of a universal bursting regime. (D) Variance (2nd cumulant), 3rd cumulant and 4th cumulant of single allele transcription rate as a function of mean transcription rate R in NC13 (square) and early NC14 ($7.5 \leq t < 20.5$ min; circle). The single allele transcription rates are estimated within 1-min-intervals, highlighting a strong departure of the cumulants from a constitutive Poisson regime (dashed line, σ^2, κ_3 and $\kappa_4 = R$). Our data approaches the Poissonian regime only on the extreme ends of the R spectrum. Moreover, the mean-variance relationship has a marked concave parabolic shape. Such a mean-variance relationship is consistent with the prediction of a 2-state model of bursting, where changes in R results from modulation of $P_{\rm ON}$ [23]. Together, these results suggest that the gap genes transition all the way from fully OFF ($P_{\rm ON} = 0$) to fully ON ($P_{\rm ON} = 1$), following a common bursting regime. Vertical gray bars correspond to low, mid, and high R, as in A.

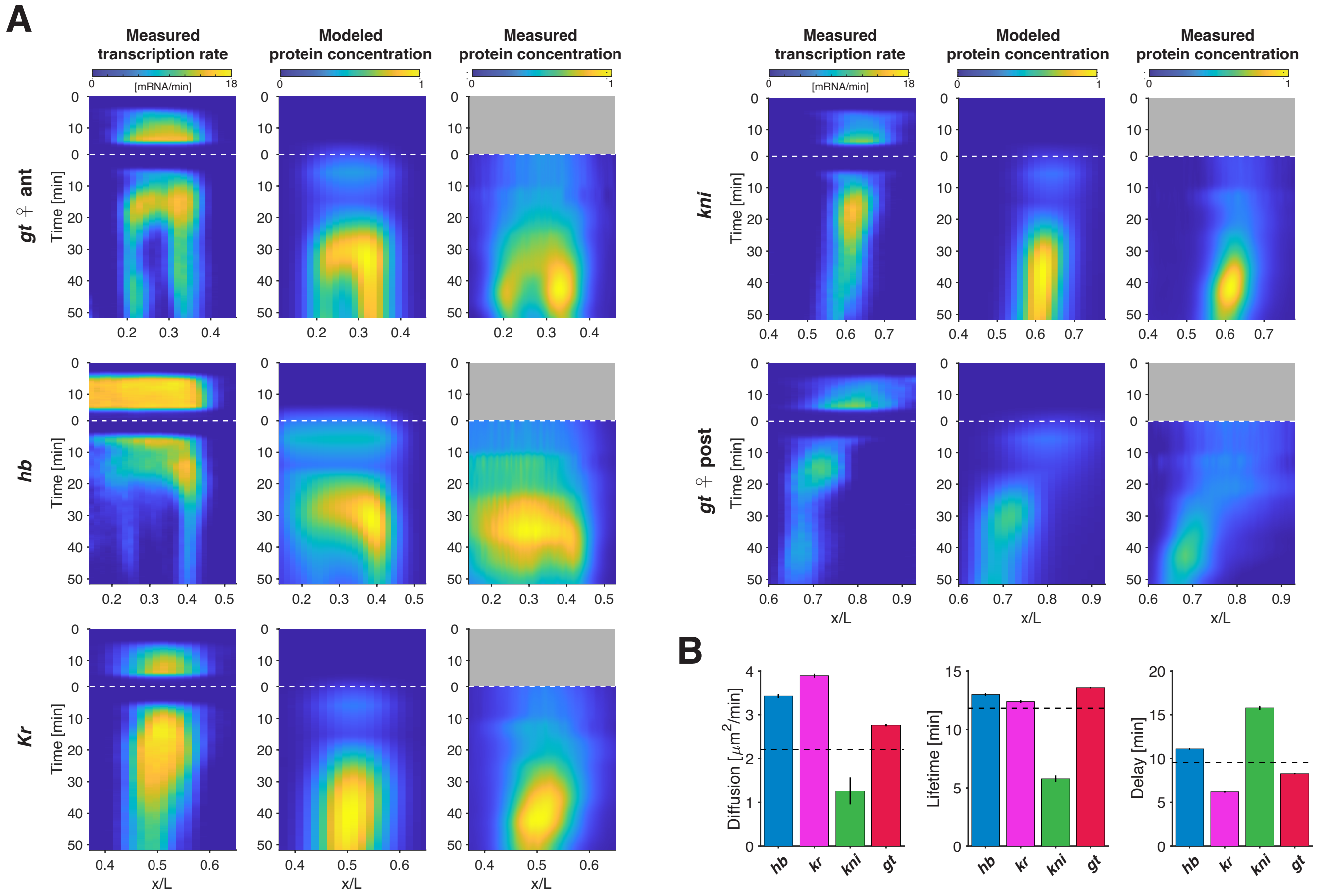

FIG. S4. **Mean transcription rate explains dynamic pattern establishment.** (A) A simple modeling attempt for protein accumulation from mean transcription rate measurements. The mean transcription rate (left column) across space and time is estimated by normalizing the measured mean activity by the elongation time and applying a minor correction for the delay (< 1 min) resulting from the loop insertion location. Horizontal white dashed lines correspond to the transition (mitosis) from NC13 to NC14. Protein accumulation (middle column) is computed from the mean transcription rate as the convolution of the latter with a kernel modeling protein decay, diffusion, and delay due to mRNA export, translation, and nuclear import. This simple model introduces three free parameters, a protein lifetime, a diffusion constant, and a time delay (see B). These three parameters were set by minimizing the mean squared error with previously measured protein patterns from carefully staged gap gene antibody staining (right column; Dubuis et al., 2013). Small residual deviations between the middle and right columns might be due to post-transcriptional regulatory processes that our simple model does not account for. (B) Parameters estimated for the modeled accumulation of effective proteins as described in A. The three parameters were either estimated for each gene separately (color bars) or all genes together (dashed lines, used for middle column in A). Overall, the effective parameters are mostly in line with previous estimates [60].

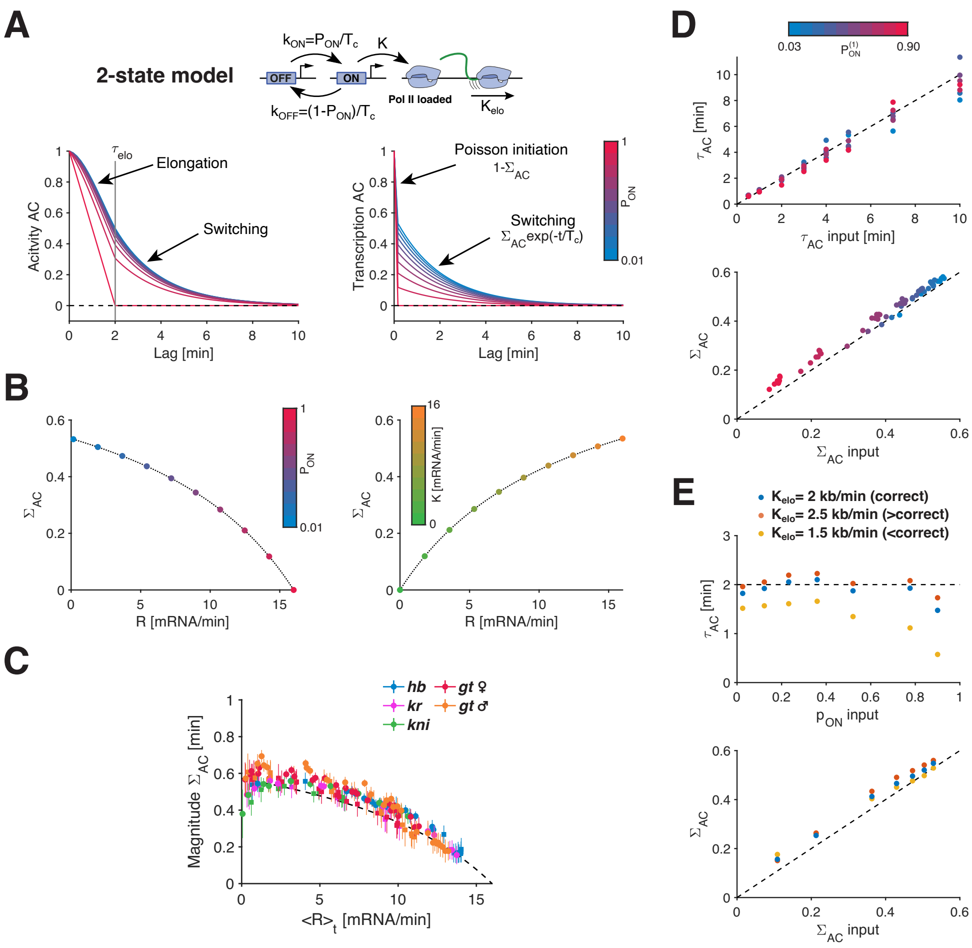

FIG. S5. **Single-allele transcription rate fluctuations reveal key bursting characteristics.** (A) Interpreting auto-correlation function using the 2-state model of transcriptional bursting. In this model for a single gene copy (top), the gene promoter switches stochastically between an OFF and ON state with rates k_{OFF} and k_{ON}, where in the latter Pol II can be loaded at rate $K^{(1)}$ and elongate at rate K_{elo}. The superscript $^{(1)}$ denotes the parameter for a single gene copy. The auto-correlation functions were computed from the model using a switching correlation time $T_{\mathrm{C}}^{(1)} = 1/(k_{\mathrm{OFF}} + k_{\mathrm{ON}}) = 2$ min, a Pol II elongation time $\tau_{\mathrm{elo}} = L_g/K_{\mathrm{elo}} = 2$ min (where L_g is the gene length) and an initiation rate $K^{(1)} = 8$ mRNA/min; the steady state ON-probability $P_{\mathrm{ON}}^{(1)} = k_{\mathrm{ON}}/(k_{\mathrm{ON}} + k_{\mathrm{OFF}})$ varies from 0 to 1 (i.e., blue to red color code, a fraction of nuclei in ON state or fraction of time a nucleus is in ON state). In principle, promoter switching (generating bursts) leads to temporal correlations in the transcriptional activity time series (Activity AC). However, from the raw live measurements, these correlations are hard to distinguish from the ones introduced by elongation (left), specifically when the switching correlation time $T_{\mathrm{C}}^{(1)}$ is close to or smaller than the elongation time τ_{elo}. Instead, performing auto-correlation analysis on deconvolved single allele transcription rates resolves the switching correlations (Transcription AC, right), since correlations due to elongation have been removed. Thus, the switching correlation time $T_{\mathrm{C}}^{(1)}$ can be estimated by fitting an exponential to the decay of the Transcription AC. (B) Expected effect of the ON-probability (left) and Pol II initiation rate (right) on the magnitude of the correlated fluctuations, Σ_{AC}, for $T_{\mathrm{C}}^{(1)} = 2$ min. (Left) As $P_{\mathrm{ON}}^{(1)}$ increases, the transcription rate $R = 2K^{(1)}P_{\mathrm{ON}}^{(1)}$ increases (here for 2 sister chromatids), and Σ_{AC} decreases until it vanishes at $P_{\mathrm{ON}}^{(1)} = 1$ (Poisson regime). This behavior is consistent with our data shown in C. (Right) At fixed $P_{\mathrm{ON}}^{(1)}$ ($P_{\mathrm{ON}}^{(1)} = 0.5$) and varying initiation rate $K^{(1)}$, the Σ_{AC} increases with growing transcription rate R. This behavior is the opposite of what we observed in our data. In both cases (Left and Right) the dotted line corresponds to the exact solution for Σ_{AC}, which is well-approximated by $\Sigma_{\mathrm{AC}} = \Delta t K^{(1)}(1-P_{\mathrm{ON}}^{(1)})/(1+\Delta t K^{(1)}(1-P_{\mathrm{ON}}^{(1)}))$, where $\Delta t = 10$ s corresponds to the data sampling time. (C) Magnitude Σ_{AC} of the correlated fluctuations in single-allele gap data as a function of mean transcription rate R. All gap data (color) collapses showing a universal trend (dashed line, guide to the eye). The fraction of correlated variability decreases as R increases, as expected when approaching a constitutive regime of uncorrelated Poisson initiation (see B). (D) Correlation time and correlated magnitude are properly retrieved after single allele deconvolution. Using the Gillespie algorithm, we generated simulated data ($N = 200$ and 50 min long cell recordings per condition) according to the 2-state model in A. For each input condition ($P_{\mathrm{ON}}^{(1)}$ from 0.03 to 0.9 and $T_{\mathrm{C}}^{(1)}$ from 0.5 to 10 min), we performed single allele deconvolution and computed the auto-correlation on the resulting transcription rates. We estimated the correlation time $T_{\mathrm{C}}^{(1)}$ and the magnitude Σ_{AC} by fitting exponential. Both parameters are properly retrieved with minimal biases. Color code stand for $P_{\mathrm{ON}}^{(1)}$ and dashed line for slope 1. (E) Estimating deconvolution biases due to elongation rate measurement bias. As in D, we generated simulated data ($P_{\mathrm{ON}}^{(1)}$ from 0.03 to 0.9 and $T_{\mathrm{C}}^{(1)} = 2$ min) and aimed to deconvolve the data with elongation rate higher (orange dot) or lower (yellow dot) than the correct value (blue dot, used to generate the data as in F). Overall, the parameters are estimated correctly, with larger biases at large $P_{\mathrm{ON}}^{(1)}$ and when underestimating the elongation rate (yellow).

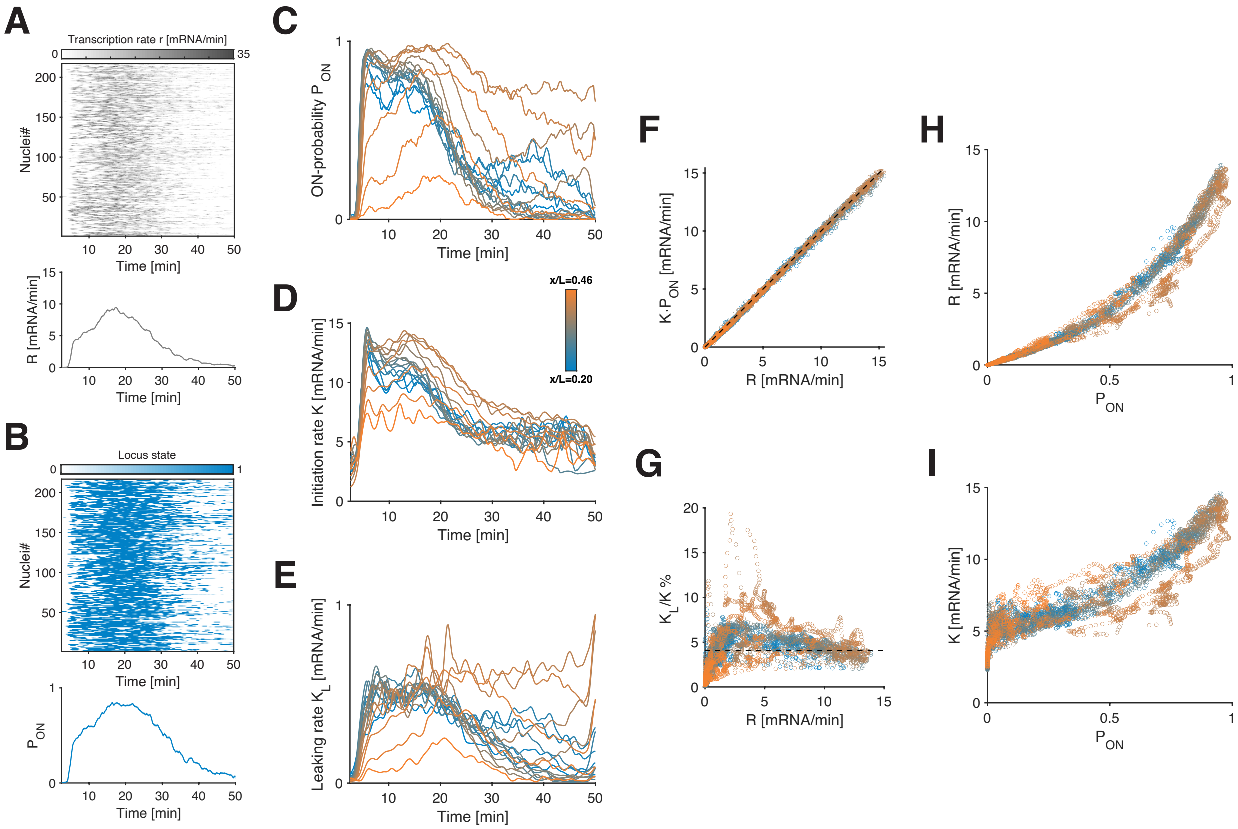

FIG. S6. **Direct estimation of instantaneous mean transcription parameters for *hunchback* in NC14.** (A-B) Heatmaps of deconvolved single allele transcription rates (A) (estimated over 10 s intervals) and of corresponding ON-OFF periods (B) (obtained from burst calling) as a function of time during NC14 for $N = 217$ nuclei expressing *hb*-MS2 at AP position $x/L = 0.43$. Instantaneous mean transcription parameters such as transcription rate R (A, bottom) and ON-probability P_{ON} (B, bottom) are obtained by vertically averaging the heatmaps (top) over all nuclei, respectively. (C-E) *hb* ON-probability P_{ON} (C), initiation rate K (D), and leaking rate K_L (E), as a function of time in NC14 for all AP positions (color coded). P_{ON} is computed as in B, while K and K_L are obtained by averaging over all nuclei in each AP bin the single allele transcription rate (A) conditioned on the locus being ON or OFF (B), respectively (as opposed to R, obtained by averaging regardless of allele state). (F) Transcription rate R versus the product of the initiation rate K and the ON-probability P_{ON} for *hb* in NC14 at all time points and positions. The color code stands for AP position as in Fig. 3 and 4. As it should be by construction, R can be decomposed into the product of K and P_{ON}. (G) Leaking rate K_L over initiation rate K in % as a function of the transcription rate R. The leaking rate K_L never exceed 5% of K on average, supporting our ability to identify well-demarcated bursts over the whole range of transcription rate. (H-I) Transcription rate R (I) and initiation rate K (J) as a function of P_{ON}, for all time points and positions, demonstrating a massive data collapse, suggesting that P_{ON} is the central regulatory parameter for transcriptional bursting.

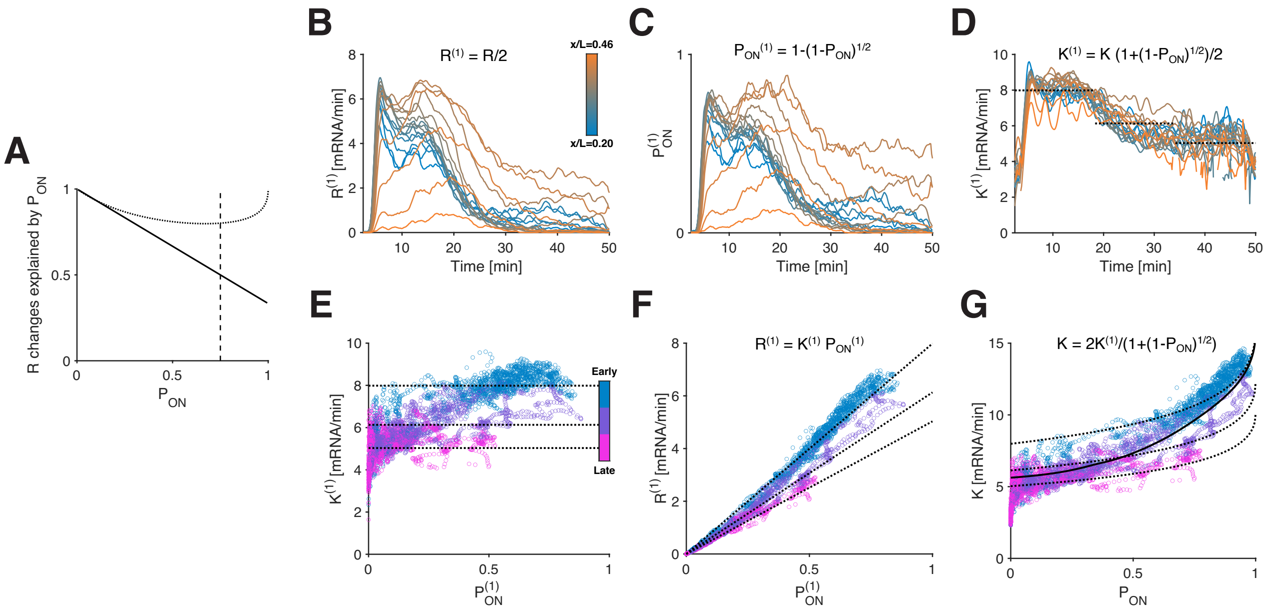

FIG. S7. **Single gene copy parameter for *hunchback*.** (A) Fraction of changes in R explained by $P_{\rm ON}$ as a function of $P_{\rm ON}$. Black line shows $d\log P_{\rm ON}/dP_{\rm ON}$ normalized by the sum $d\log K/dP_{\rm ON} + d\log P_{\rm ON}/dP_{\rm ON}$. The dotted black line is the corrected contribution assuming two independent sister chromatids (see B-G). Most of the changes in R are thus dictated by $P_{\rm ON}$. (B-D) Single gene copy (SGC) parameter computed from the estimated effective parameters for *hb* in NC14, assuming two independent sister chromatids. The color code stands for AP as B. The spatiotemporal regulation of the SGC transcription rate $R^{(1)}$ (B) and the SGC ON-probability $P^{(1)}_{\rm ON}$ (C) are very similar to their corresponding effective parameters, R and $P_{\rm ON}$. On the other hand, the SGC initiation rate $K^{(1)}$ (D) no longer varies across position, but only as a function of time. During NC14, the SGC initiation rate $K^{(1)}$ decreases by 38%, from 8.0 mRNA/min (first dotted line) to 5.0 mRNA/min on average (third dotted line), with most of the decrease happening between the 16$^{\rm th}$ and 34$^{\rm th}$ minute mark (second dotted line at 6.1 mRNA/min). Interestingly, the distribution of $K^{(1)}$ leads to a mean Pol II spacing for a single active chromatid at $K_{\rm elo}/K^{(1)} = 303 \pm 73$ bp, which is consistent with average the Pol II spacing of 330±180 bp in the classic Miller spreads [36]. (E-G) Temporal changes in SGC initiation rate and independent sister chromatid assumption explain the dependence of the effective initiation on the ON-probability. Color code stands for three time windows in NC14: early (cyan, 2.5-16.7 min), mid (purple, 16.7-34.2 min), and late (magenta, 34.2-50 min). (E) Most of the variation in $K^{(1)}$ is explained by time, rather than $P^{(1)}_{\rm ON}$. The dotted lines are drawn at the same $K^{(1)}$ values as in D. (F) The SGC transcription rate $R^{(1)}$ appears almost linearly dependent on $P^{(1)}_{\rm ON}$. The nonlinearity is mostly explained by temporal changes in $K^{(1)}$, as highlighted by dotted lines whose slopes are the $K^{(1)}$ values from D and E. (G) Under the two independent sister chromatids assumption, the effective initiation rate K depends on $P_{\rm ON}$ and on $K^{(1)}$, which varies as a function of time (see D). As $P_{\rm ON}$ increases, the propensity to observe two gene copies initiating transcription at the same time increases, which explains up to a factor of two in the dependence of K on $P_{\rm ON}$. Indeed, the dotted lines correspond to the predicted behavior using the same three constant values of $K^{(1)}$ as in D. In addition, $K^{(1)}$ varies by up to 38% along time during NC14, as can be seen in D. Together, it explains close to a factor of 3.2 in K variation with $P_{\rm ON}$. This reasoning explains the observed relationships very well (black line) and argues for a very weak dependence of K on $P_{\rm ON}$.

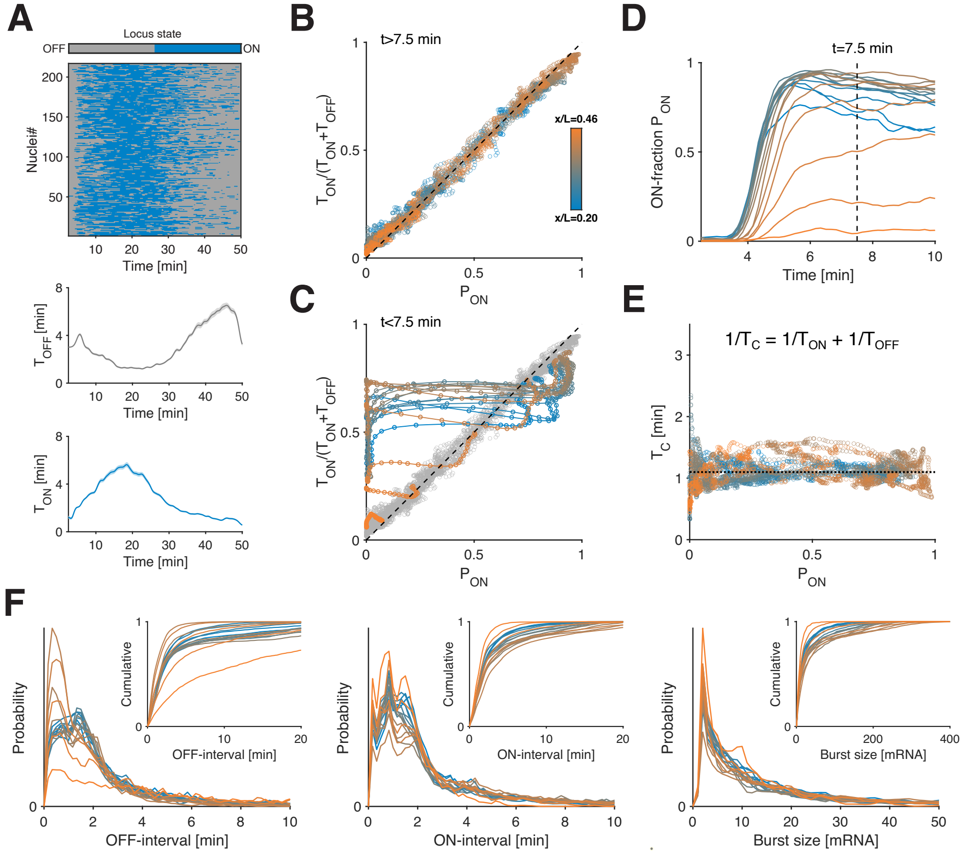

FIG. S8. **Direct estimation of instantaneous mean ON and OFF-time for *hunchback* in NC14.** (A) Binarized heatmap from Fig. S6B. Instantaneous mean OFF-time T_{OFF} (bottom, gray) and mean ON-time T_{ON} (bottom, blue) are obtained by the weighted average of the ON and OFF times over all nuclei (see methods). The weights are given by the inverse of the number of time points within each period. (B) The ratio of T_{ON} over the sum of T_{ON} and T_{OFF} versus ON-probability P_{ON} for all positions and time points beyond the 7.5 min mark in Fig. 2B-C. The 2-state model predicts that near-steady state (after an initial transient) both quantities match (C and D). The agreement between these quantities is a strong indication of the self-consistency of our general approach for extracting these bursting parameters and that the system operates near steady state. Thus, temporal changes in transcription parameters (due to developmental regulation) beyond the 7.5 min mark must be slow enough to allow relaxation. (C) Evidence for initial out-of-steady state transient for *hb* in early NC14. Near the steady-state regime, P_{ON} should be well-approximated by $T_{\mathrm{ON}}/(T_{\mathrm{ON}} + T_{\mathrm{OFF}})$, as it is the case beyond the 7.5 min mark in NC14 (gray circles tracing the diagonal; see B). However, at the post-mitotic onset of transcription (∼3–7.5 min into NC14) we observe strong deviations from the expected near-steady state relationship at all positions (color curves). The system is undergoing a fast transient relaxation that drives it near-steady state within the first 8 min after mitotic exit, see D. (D) Close-up of the first 10 min of Fig. 3D shows a rapid transient in the ON-probability for *hb* at the onset of NC14. The vertical dashed line at 7.5 min marks the transition between the transient and the near-steady-state regime as observed in C. (E) Effective switching correlation time T_{C} (defined as: $1/T_{\mathrm{C}} = 1/T_{\mathrm{ON}} + 1/T_{\mathrm{OFF}}$) as a function of P_{ON}, computed using data points in Fig. 2B-C. T_{C} is mostly conserved across time points and position and is P_{ON} independent. Dotted line corresponds to mean $T_{\mathrm{C}} = 1.1 \pm 0.2$. (F) Distributions and cumulative distributions of OFF-intervals, ON-intervals and burst size for all positions (color coded). These distributions are computed using all alleles and time points at a given position (see heatmpas in Fig. S6A and S8A). As such, they represents non-stationary transcriptional dynamics (see Fig. 2A,C and S6C-D), and are consequently less amendable to direct interpretation (e.g. of the non-exponential nature OFF- and ON-interval distribution) as deviations from the 2-state model stationary expectation.

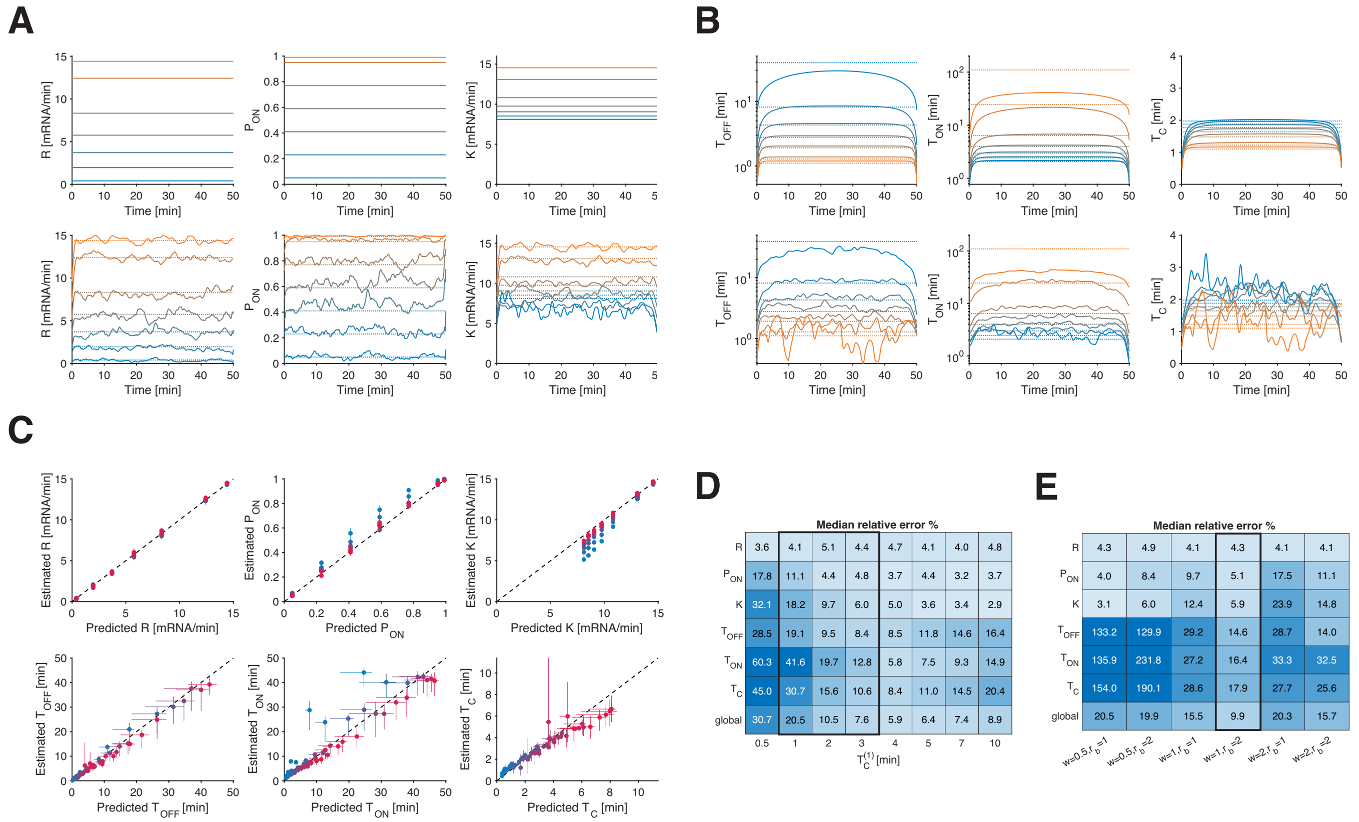

FIG. S9. **Validation of transcription parameter estimation using stationary simulated data.** Burst calling permits effective estimation of transcription parameters. For each set of input parameters ($K^{(1)}$, $T_C^{(1)}$, $P_{\mathrm{ON}}^{(1)}$), we generated simulated data (200 alleles and 50 min long time series at 10 s intervals and with the same imaging noise as measured in real data). Using the 2-state model (Fig. S3D) and the Gillespie algorithm, we generated time series for each individual sister chromatid. We summed the initiation events of both chromatids assuming independence. The resulting initiation events were convolved with the elongation kernel to generate synthetic single allele signal data over which measurement noise was added. We performed single allele deconvolution and burst calling as described (Fig. 3A) to estimate the effective mean transcription parameters (R, K, P_{ON}, T_{ON}, T_{OFF} and T_{C}) for each simulated set of alleles. (A-B) Comparison between the theoretically expected (top row) and the estimated from burst calling effective parameters (bottom row) as a function of time, using stationary (constant in time) input parameters ($T_C^{(1)} = 2$ min, $K^{(1)} = 8$ mRNA/min and $P_{\mathrm{ON}}^{(1)}$ varies from 0.03 to 0.9 that is color coded from blue to orange). Overall, the estimated effective parameters are recovered very well. (A) Despite sampling fluctuations, the constancy of the parameters in time is properly preserved (bottom row), as it should be based on input (top row). (B) Despite the stationary nature of the input parameters $T_C^{(1)}$ and $P_{\mathrm{ON}}^{(1)}$, biases for T_{ON}, T_{OFF} and T_{C} (top row) is expected due to the finite length of the simulated time series (censoring), especially noticeable near the beginning and the end, where the estimations are "bent" (the resulting mean time estimate cannot exceed the width of the time window used to perform the estimate). However, our parameter estimation (bottom row) is very much in line with the expected biases (top row). (C) Global comparison between expected and estimated parameters for the stationary case (input parameters are constant in time). The parameter estimation was performed on a large simulated data set that includes data in A and B ($T_C^{(1)} = 2$ min, $P_{\mathrm{ON}}^{(1)}$ from 0.03 to 0.9) and data for other values of $T_C^{(1)}$ comprised between $T_C^{(1)} = 0.5$ (blue dots) to $T_C^{(1)} = 10$ (red dots). Each dot results from one combination of input parameters ($K^{(1)}$, $T_C^{(1)}$, $P_{\mathrm{ON}}^{(1)}$) and corresponds to the median effective parameter and the error bars to the 68% confidence interval estimated over 50 min. Our deconvolution and burst calling approaches lead to an excellent estimation of the effective parameters over a large range of $T_C^{(1)}$ and $P_{\mathrm{ON}}^{(1)}$ values, albeit with noticeable biases in P_{ON}, K and T_{ON} when $T_C^{(1)}$ approaches 0.5 min (blue dots). Importantly, biases for the effective switching correlation time T_C are small, supporting our ability to detect its constancy in real data. (D) Summary of median relative error for each effective parameter estimated from the data in C as a function of input $T_C^{(1)}$. Parameter estimated from real data (Fig. S12B) suggests that $T_C^{(1)}$ lies within 1 and 3 min (black border). (E) Summary of median relative error for each effective parameter estimated from the whole data set in C as a function of the burst calling parameters. Burst calling depends on two free parameters: the time window w over which the rate is estimated, and the rate threshold r_b applied to call the burst (see Fig. 3A). Our default parameter values are $w = 1u$ and $r_b = 2/u$ (with $u = 5\Delta t = 5/6$ min), which should be close to optimal given the estimated correlation time of $\tau_{\mathrm{AC}} \sim 1$ min (Fig. 2F) and our measurement sensitivity of 1–2 mRNA. When testing the effect of different w and r_b values on the median relative error, our default choice leads to the lowest global relative error.

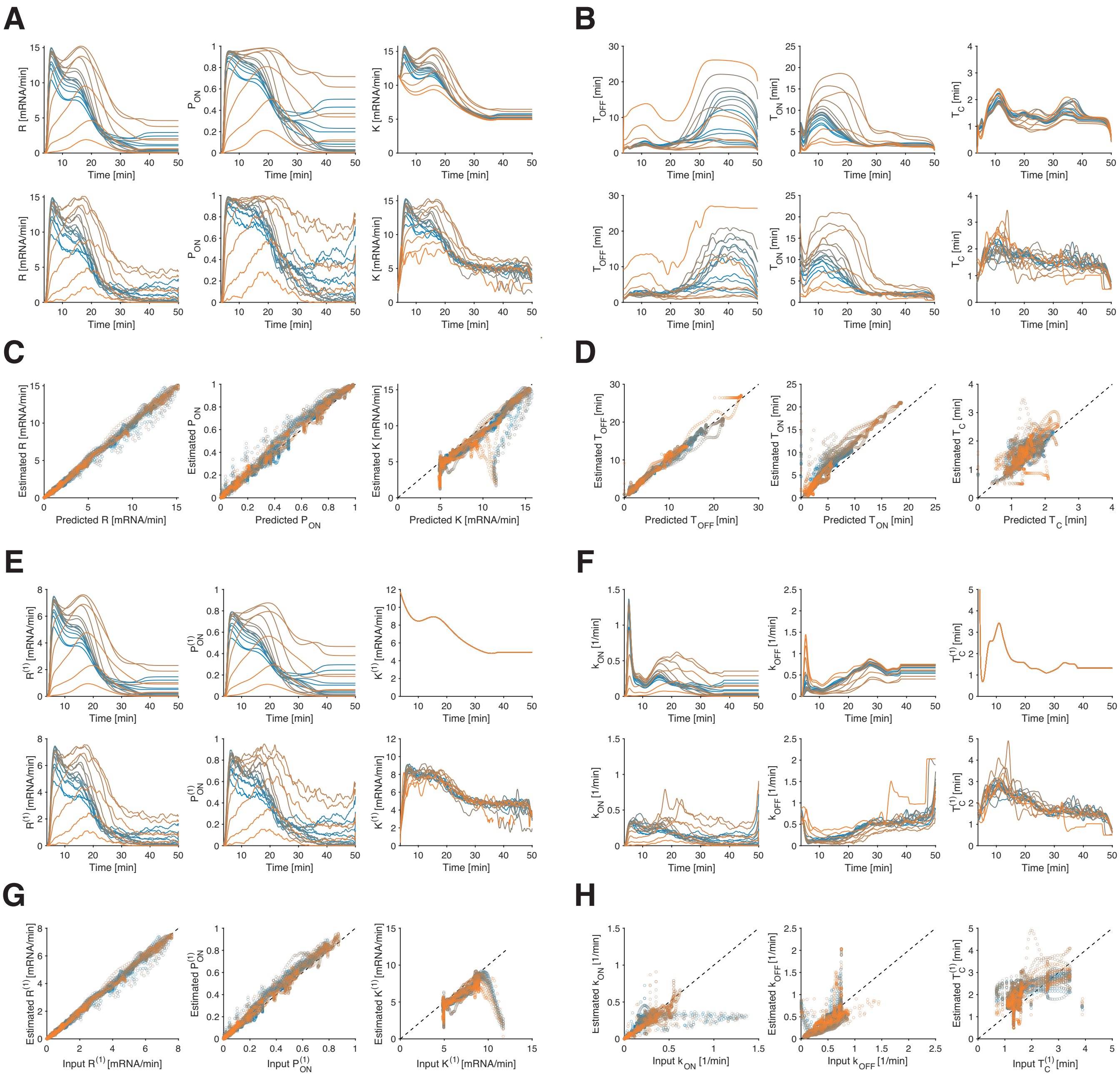

FIG. S10. **Validation of transcription parameter estimation using *hb*-like non-stationary simulated data.** Simulated data is generated as in Fig. S9, except that the input parameters are non-stationary (time-dependent). (A-B) Comparison between expected (A, B, top row) and estimated effective parameters (A, B, bottom row) for time-dependent input parameters (A, B, top row), mimicking the transcriptional output of *hb* in NC14. Color code (blue to orange) stands for virtual AP position. Estimated effective parameters tightly recapitulate the time-dependence of the expected parameters, allowing us to also capture the temporal regulation of the gap genes. (C, D) We show with this realistic test case that our estimated parameters (A, B, bottom row) using burst calling very closely match the expected effective ones (A, B, top row). Indeed, most of the time points (circles) lie on the line of slope one (dashed line), which strongly supports our ability to precisely characterize the transcription parameters from real data. (E–F) Comparison between input (E, F, top row) and burst calling estimated single-gene copy parameters (E, F, bottom row) as a function of time. The time-dependent input parameters (E, F, top row) were used to generate the synthetic *hb* data in A and B. Color code (blue to orange) stands for virtual AP position as in A and B. The estimated single-gene copy parameters were computed from the effective ones (A, B, bottom row) assuming the latter originate from two independent sister chromatids. Namely, we get $R^{(1)} = R/2$, $P_{\mathrm{ON}}^{(1)} = 1-(1-P_{\mathrm{ON}})^{1/2}$, $K^{(1)} = K(1+(1-P_{\mathrm{ON}})^{1/2})/2$, which are exact, and assuming steady state $T_{\mathrm{C}}^{(1)} = 2T_{\mathrm{C}}/(1+(1-P_{\mathrm{ON}})^{1/2})$, $k_{\mathrm{ON}} = P_{\mathrm{ON}}^{(1)}/T_{\mathrm{C}}^{(1)}$ and $k_{\mathrm{OFF}} = (1-P_{\mathrm{ON}}^{(1)})/T_{\mathrm{C}}^{(1)}$. (G-H) Even though the single gene copy parameters are deeply buried in the data, our simple burst calling procedure still manages to recover them correctly. Some discrepancies are observed for k_{ON}, k_{OFF} and $T_{\mathrm{C}}^{(1)}$, mostly near the beginning when the transient after mitosis violates the near-steady-state assumption. But these are expected as the relationships $k_{\mathrm{ON}} = P_{\mathrm{ON}}^{(1)}/T_{\mathrm{C}}^{(1)}$ and $k_{\mathrm{OFF}} = (1-P_{\mathrm{ON}}^{(1)})/T_{\mathrm{C}}^{(1)}$ are only valid for near-steady state.

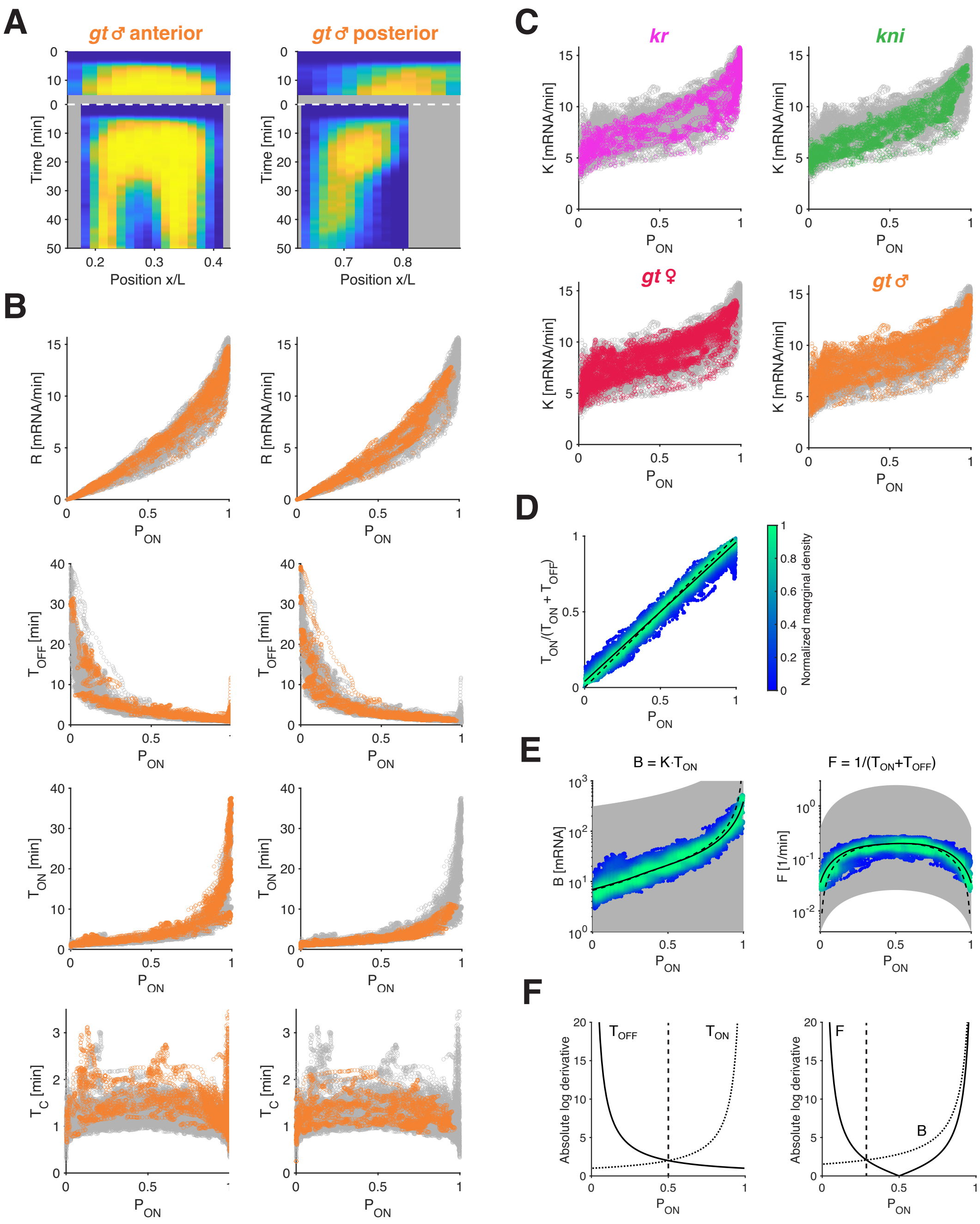

FIG. S11. **Further transcription parameters collapse.** (A) Kymograph of ON-probability for *gt* transcription in male embryos, as a function of position and time. As for the other gap genes, the spatiotemporal transcription patterns arise from complex regulation of the ON-probability. (B) transcription parameters for *gt* in male embryos for NC13 and NC14 as a function of PON (orange data points) and all the other gap gene data sets (gray). (C) Initiation rate K as a function of P_{ON}. K collapses for all gap genes across time and position. Colored data points represent individual gap genes (same color code as in Fig. 5A-E and S11A-B); underlying is the remaining data of all other genes (gray). The K–P_{ON} relationship for *hb* is shown in Fig. 3H. (D) Near-steady state relationship between ON-probability P_{ON} and $T_{\text{ON}}/(T_{\text{ON}}+T_{\text{OFF}})$ for all gap genes in NC13 ($t \geq 6.5$min) and NC14 ($t \geq 7.5$min). Although the data is distributed near the expected relationship (dash line), we observe a slight but clear bias at the extreme ends of the P_{ON} spectrum (solid line), namely $T_{\text{ON}}/(T_{\text{ON}}+T_{\text{OFF}})$ is slightly above zero at $P_{\text{ON}}=0$ and slightly below one at $P_{\text{ON}}=1$. This is a consequence of the finite nature of our recording (50 min in NC14 and 18.4 min in NC13). Thus, our recording time sets an upper limit on the length of measurable ON and OFF intervals. That limit leads to the observed bias. (E) Global scatter of the burst size B and burst frequency F (color) as a function of P_{ON} for all gap genes in both NC13 and NC14 and putative accessible space (gray region). See definitions of B and F at top of respective panels. Color code and accessible space are defined as in Fig. 5F. Solid line stands for the bursting relationships derived with bias (solid line in C) and dashed line without. (F) Absolute derivatives with respect to P_{ON} of $\log T_{\text{ON}}$ and $\log T_{\text{OFF}}$ (left), and $\log F$ and $\log B$ (right), computed from bursting relationships in Fig. 3F and E above. Decomposition in terms of log derivative is convenient since the sources of changes in P_{ON} and R become additive, i.e., $\log(1-P_{\text{ON}})/P_{\text{ON}} = \log T_{\text{OFF}} - \log T_{\text{ON}}$ and $\log R = \log F + \log B$. Thus, transition in the predominant type of expression modulation occurs at the crossing of the derivatives: at $P_{\text{ON}}=0.5$ for ON and OFF-times and $P_{\text{ON}}=1/3$ for burst size and frequency.

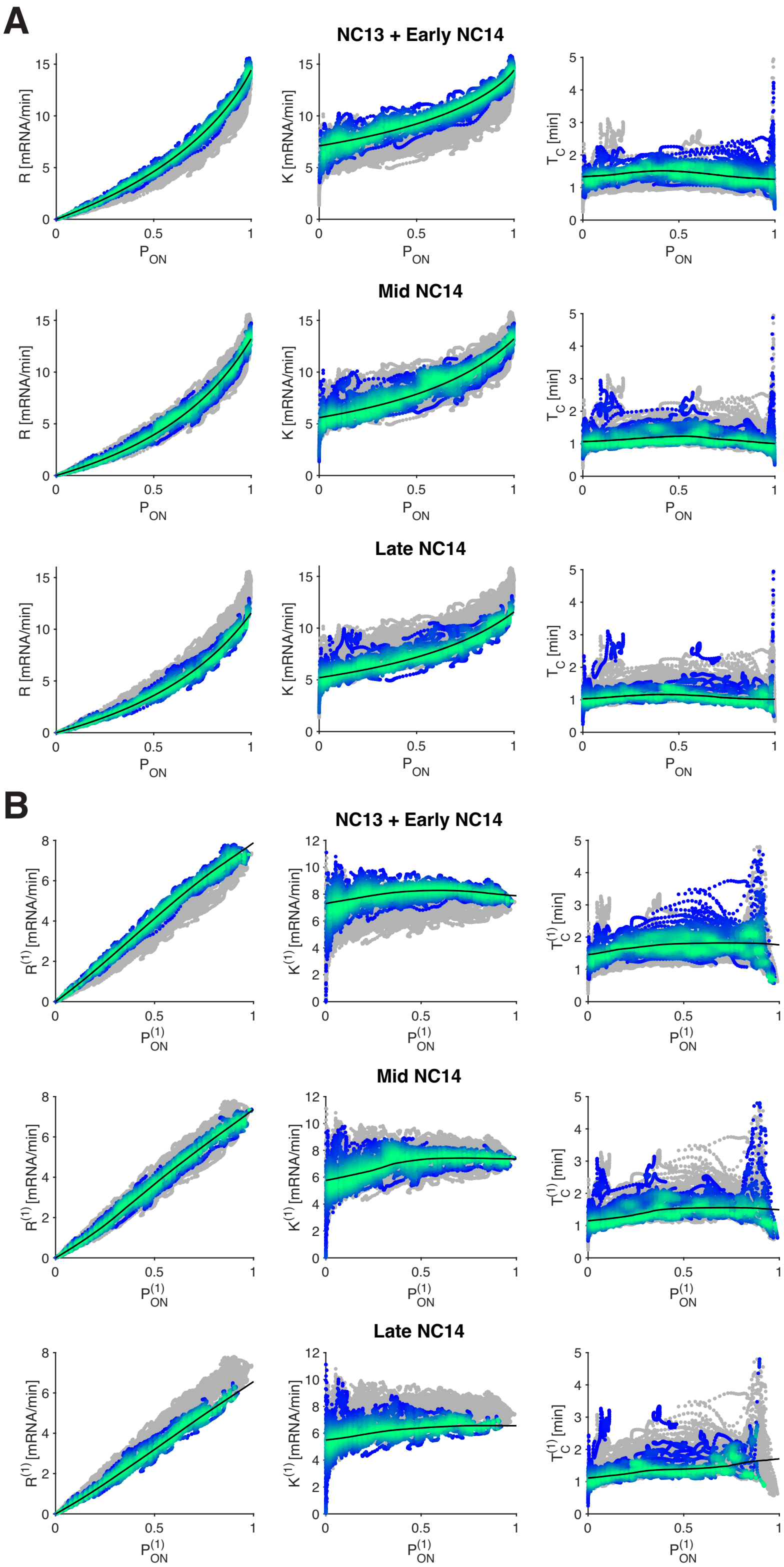

FIG. S12. **Common bursting relationships across developmental time and for single gene copy.** (A) Global scatter of effective transcription parameters as a function of P_{ON} for all gap genes estimated within three time windows, corresponding to NC13 ($6.5 \leq t$ min) plus early NC14 ($7.5 \leq t < 20.5$ min), mid NC14 ($20.5 \leq t < 34.5$ min), and late NC14 ($34.5 \leq t < 48$ min). The observed bursting relationships are further refined when accounting for possible temporal changes (color scatter) compared to all time-pooling (gray scatter, Fig. 5F). Indeed, small changes in K and T_{C} over developmental time ($\sim 40\%$ decrease) explain part of the observed spread in Fig. 5F. (B) As in A, but for single gene copy parameters computed from the effective ones assuming independent sister chromatids. Interestingly, the relationship between SGC transcription rate $R^{(1)}$ and SGC ON-probability $P_{\mathrm{ON}}^{(1)}$ is almost linear, confirming that the SGC initiation rate $K^{(1)}$ does not depend strongly on $P_{\mathrm{ON}}^{(1)}$. Thus, the apparent dependence of $K^{(1)}$ on $P_{\mathrm{ON}}^{(1)}$ is only effective and results from measuring two sister chromatids (two gene copies) together, instead of an isolated single gene copy.

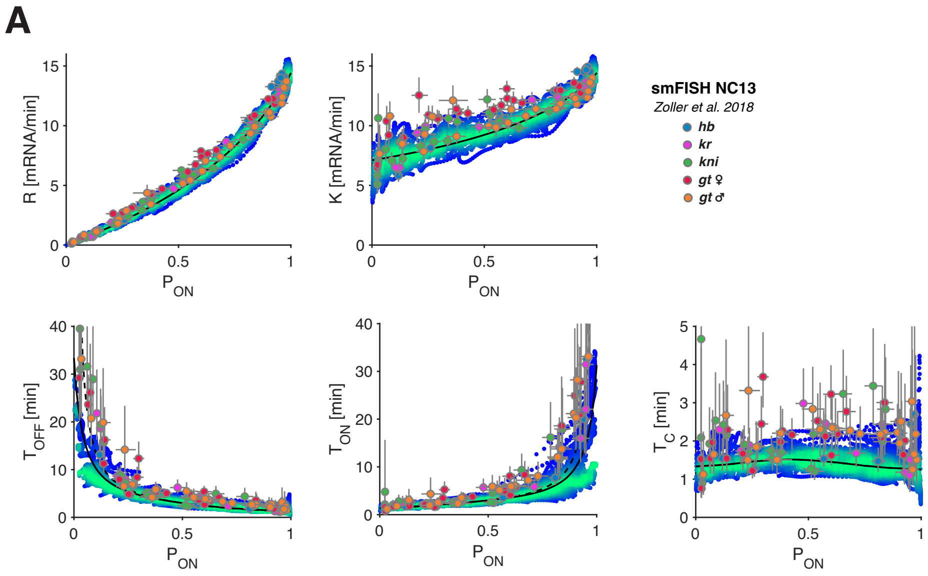

FIG. S13. **Validating bursting relationships with smFISH data.** (A) Bursting relationships in NC13 and early NC14 are consistent with parameters inferred from previous fixed measurements using smFISH. We converted the single gene copy parameters of the gap genes in mid-late NC13 (Zoller et al.) into effective parameters for two sister chromatids (color dots), with error bars corresponding to 68% confidence intervals. The resulting smFISH effective mean correlation time $T_{\mathrm{C}} = 2.06 \pm 0.66$ min is slightly smaller than in the original study ($T_{\mathrm{C}} = 3.0 \pm 1.2$ min). Two reasons explain this difference: 1) we corrected for our updated elongation rate of 1.8kb/min compared to 1.5kb/min used previously, leading to a T_{C} reduction of 17%; 2) the effective correlation time is up to two times smaller for large P_{ON} compared to the SGC correlation time $T_{\mathrm{C}}^{(1)}$ used in the original study ($T_{\mathrm{C}}^{(1)} = 2T_{\mathrm{C}}/(1+(1-P_{\mathrm{ON}})^{1/2})$, see Methods), leading to a further 17% reduction on average. Overall, the smFISH effective parameters closely verify our relationships derived from live measurements ($T_{\mathrm{C}} = 2.06 \pm 0.66$ min fixed versus $T_{\mathrm{C}} = 1.25 \pm 0.37$ min live), albeit with small deviations likely stemming from a technical origin (differences in experimental protocol and microscopy, as well as limitations imposed by fixed measurements on parameter estimation). Thus, most likely, the width of the data clustering around our relationships reflects estimation errors with limited biological variation in parameters, as can also be verified from simulated data (Fig. S5 and Fig. S6).

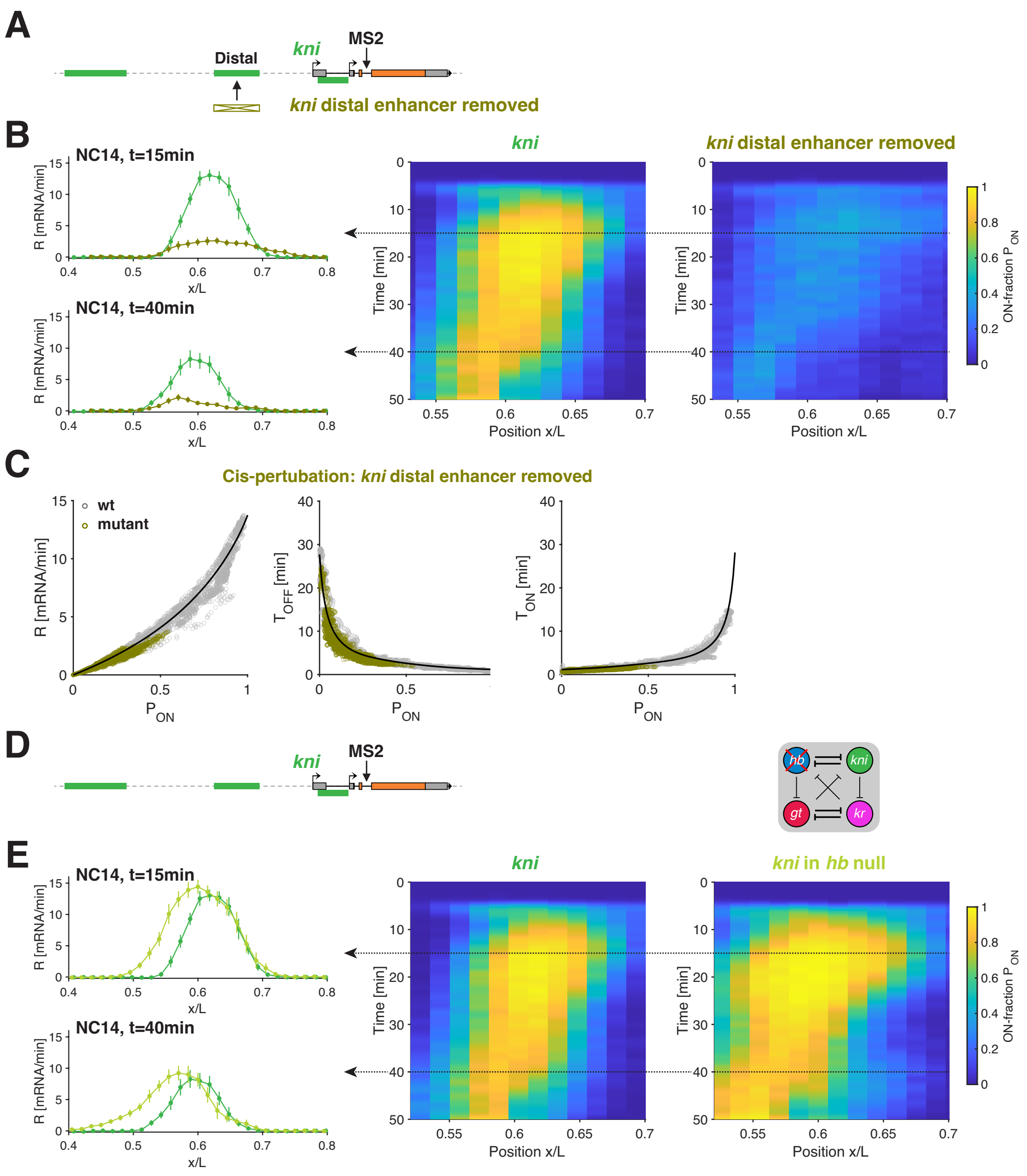

FIG. S14. **Bursting relationships verified by *cis*- and *trans*-perturbations.** (A) Distal *kni* enhancer removal. The MS2-stem loops are inserted at the same location in the mutant (enhancer deletion) and wild-type fly lines. (B) Quantification of *kni* wild-type and mutant (A) phenotypes. Both transcription rate R as a function of x/L (left) and the kymograph for P_{ON} (right) display a significant level decrease underlying the expression patterns of the mutant. Dotted arrow indicates time point in kymograph at which rate profiles (left) are depicted. (C) Transcription parameters for *kni cis*-mutant (olive) collapse on corresponding wild-type parameters (gray), as for *hb cis*-mutant (see Fig. 4C). Solid black lines correspond to the endogenous bursting relationships from Fig. 5F. (D) *kni* measurements in a *hb* null background. The absence of *hb* expression alters the network, namely the concentration of input transcription factors sensed by *kni* in the mutant fly line. (E) Quantification of *kni* wild-type and mutant (D) phenotypes. Both transcription rate R (left) and P_{ON} kymograph (right) display a significant shift of the anterior boundary in the mutant expression patterns. Dotted arrow indicates time point in kymograph at which rate profiles are depicted.

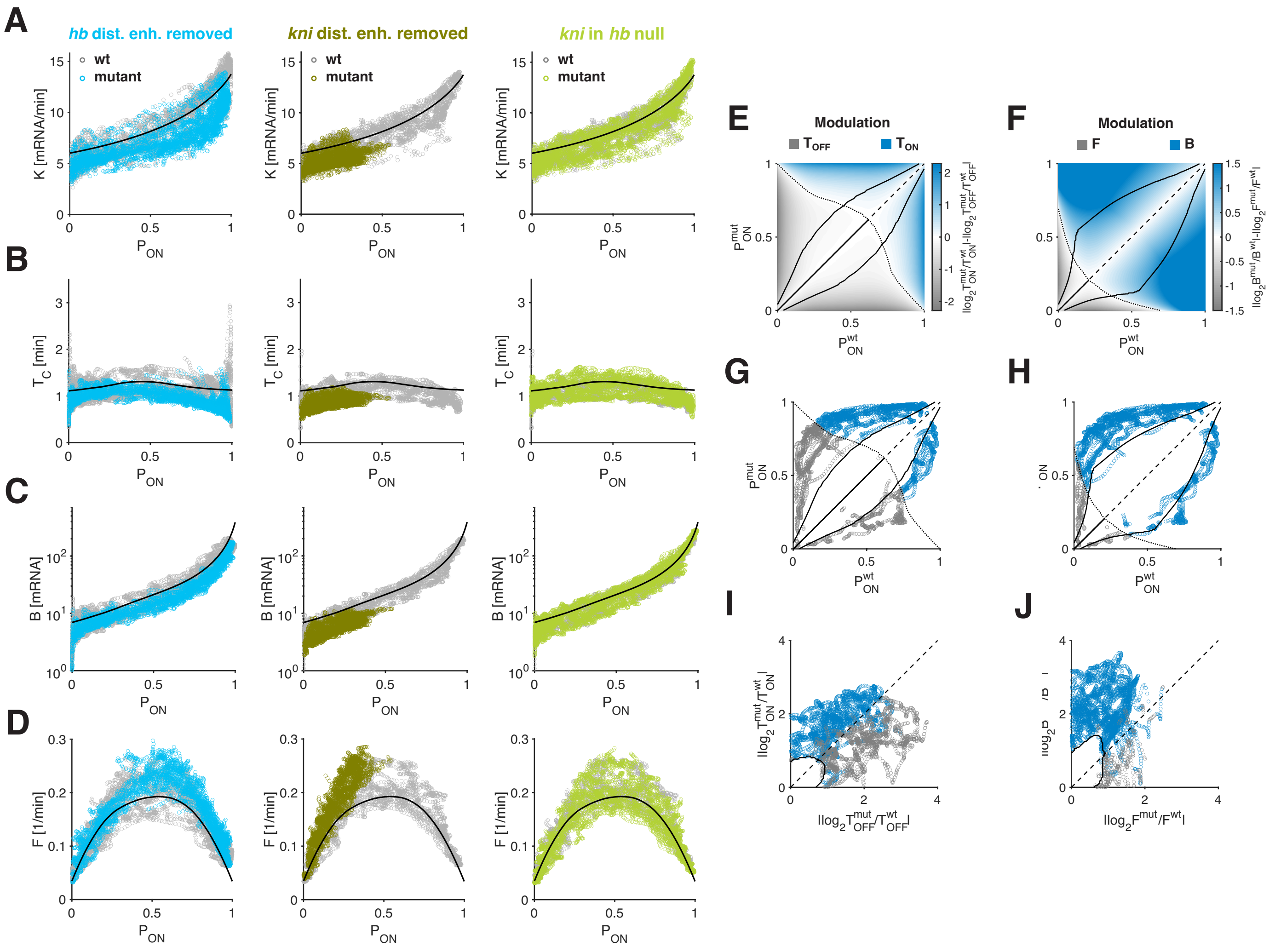

FIG. S15. **Bursting relationships predict ON and OFF modulation by *cis*- and *trans*-perturbations.** (A-D) Initiation rate K (A), correlation time T_C (B), burst size B (C), and burst frequency F (D) for *hb* in *cis*-mutation (cyan), *kni* in *cis*-mutation (olive) and for *kni* in *trans*-mutation (light green) collapse on corresponding wild-type parameters (gray). Solid black lines correspond to the endogenous bursting relationships from Fig. 5F and S11E. (E-J) Predicted T_{OFF} versus T_{ON}, and F versus B bursting modulation for mutant (*hb* in *cis*-mutation and *kni* in *trans*-mutation) based on wild-type-derived relationships (Fig. 5F and S11E, black lines). Color code stands for type of modulation. (E) The type of modulation is predicted by first approximating T_{OFF} and T_{ON} as a function of P_{ON} using the wild-type relationships. The predicted fold change in T_{OFF} ($T_{\mathrm{OFF}}^{\mathrm{mut}}/T_{\mathrm{OFF}}^{\mathrm{wt}}$) and T_{ON} ($T_{\mathrm{ON}}^{\mathrm{mut}}/T_{\mathrm{ON}}^{\mathrm{wt}}$) are then computed for all possible pairs of P_{ON} (i.e. $P_{\mathrm{ON}}^{\mathrm{wt}}$ and $P_{\mathrm{ON}}^{\mathrm{mut}}$). The dotted line delimits the regions where changes in transcription rate are either dominated by changes in T_{OFF} (gray region, $|\log(T_{\mathrm{OFF}}^{\mathrm{mut}}/T_{\mathrm{OFF}}^{\mathrm{wt}})| > |\log(T_{\mathrm{ON}}^{\mathrm{mut}}/T_{\mathrm{ON}}^{\mathrm{wt}})|$) or T_{ON} (blue region, $|\log(T_{\mathrm{OFF}}^{\mathrm{mut}}/T_{\mathrm{OFF}}^{\mathrm{wt}})| < |\log(T_{\mathrm{ON}}^{\mathrm{mut}}/T_{\mathrm{ON}}^{\mathrm{wt}})|$). The solid black lines delimit the region, where changes in T_{OFF} and T_{ON} are not significant given the "thickness" of our relationships (95% confidence intervals, see Methods). Thus, this procedure defined a look-up table enabling prediction of the type of modulation using pairs of P_{ON}. (F) Same as (E), except using F and B instead of T_{OFF} and T_{ON}. (G) Scatter plot of all the P_{ON} pairs from *hb* wt and *cis*-mutant (at same spatiotemporal location). Colors correspond to the predicted modulation (T_{OFF} dominated in gray and T_{ON} dominated in blue) using the look-up table in A. (H) Same as (G), except using F and B instead of T_{OFF} and T_{ON}. (I) Verification of predicted modulation in G. For each P_{ON} pair, we computed the T_{OFF} ($T_{\mathrm{OFF}}^{\mathrm{mut}}/T_{\mathrm{OFF}}^{\mathrm{wt}}$) and T_{ON} ($T_{\mathrm{ON}}^{\mathrm{mut}}/T_{\mathrm{ON}}^{\mathrm{wt}}$) fold change using the estimated T_{OFF} and T_{ON} from data (Fig. 4C). Supporting our ability to predict the modulation, almost all the blue data points (predicted as T_{ON} modulation) are located above the slope 1 diagonal (dashed line), whereas most of the gray ones (predicted as T_{OFF} modulation) are below. Thus, for most data points (> 85%) the prediction is correct (Fig. 4E). (J) Same as (I), except using F and B instead of T_{OFF} and T_{ON}. Supporting our ability to predict the modulation, almost all the blue data points (predicted as B modulation) are located above the slope 1 diagonal (dashed line), whereas most of the gray ones (predicted as F modulation) are below. Thus, for most data points (> 95%) the prediction is correct (Figure 4G).

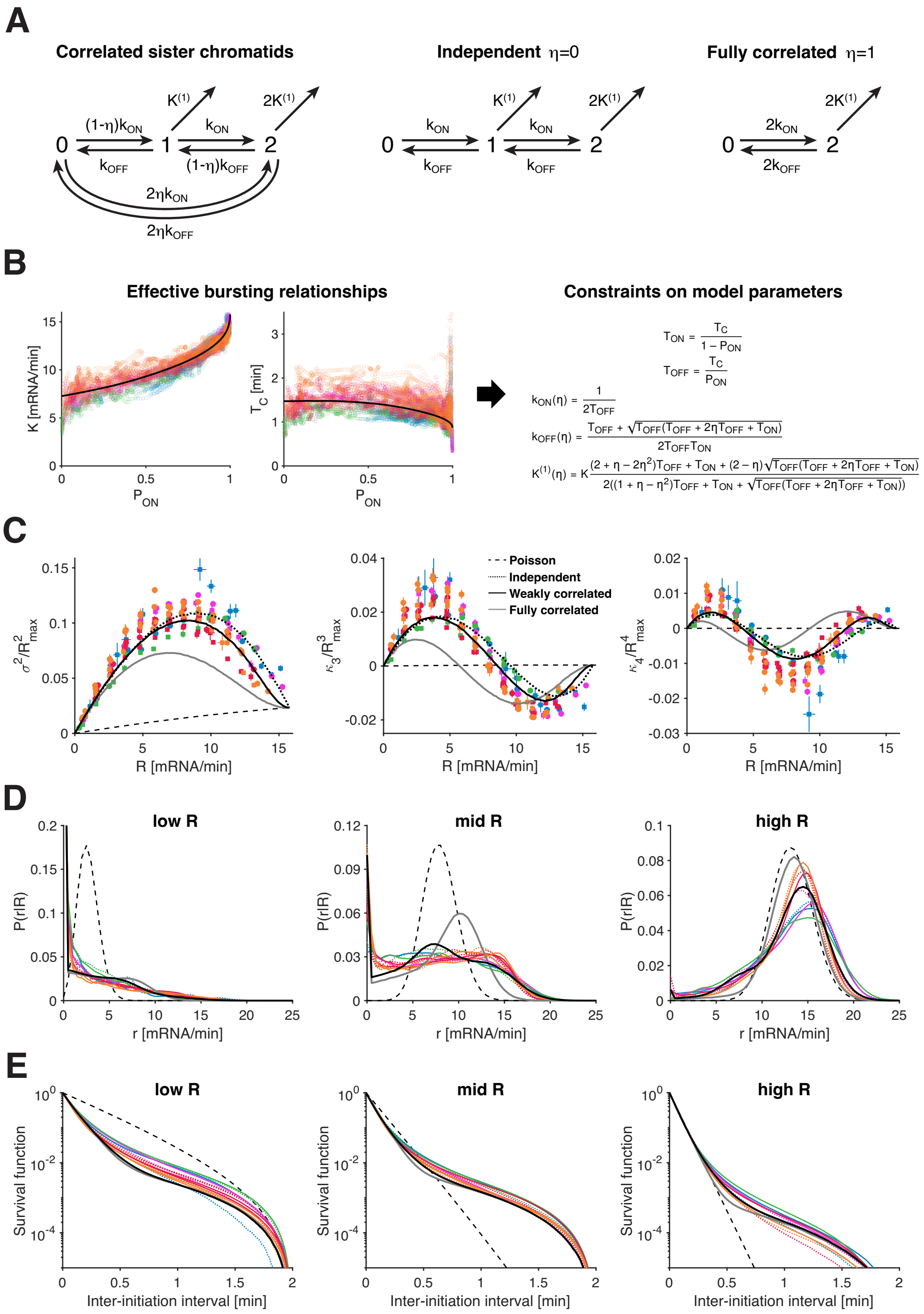

FIG. S16. **Bursting relationships provide strong empirical constraints on transcription models.** [Caption see next page.]

FIG. S16. **Bursting relationships provide strong empirical constraints on transcription models.** This figure demonstrates how the derived bursting relationships help to discriminate between transcription models. Also shown how models that specifically account for two indistinguishable sister chromatids provide a better explanation for the data, suggesting that transcription loci in the embryo are formed by two weakly correlated sister chromatids. (A) Minimal class of models describing two identical and possibly correlated sister chromatids. The gene copy on each sister chromatid behaves as a 2-state model whose transitions between the active and inactive state can be coupled ($\eta > 0$). Assuming two identical gene copies, the model reduces to three effective states corresponding to 0, 1 and 2 active copies. Transition rates between the effective states are function of the single gene copy switching rates k_{ON} and k_{OFF}. Production of mRNAs in the active states ('1' or '2') is determined by the single gene copy initiation rate $K^{(1)}$. The coupling parameter $\eta \in [0, 1]$, together with k_{ON} and k_{OFF}, determine the correlation coefficient $\rho \in [0, 1]$ between the two bursting gene copies, which is given by $\rho = \frac{P(2)-(P(2)+P(1)/2)^2}{(P(2)+P(1)/2)(1-P(2)-P(1)/2)}$ with $P(1)$ and $P(2)$ the occupancy probability of states '1' and '2'. In the limiting case $\eta = 0$ the two gene copies are independent and $\rho = 0$, whereas in the case $\eta = 1$ the two gene copies are fully correlated and $\rho = 1$. (B) The identified bursting relationships (derived without fitting) impose strong constraints on possible models. Any model of transcription (characterized by a set of active and inactive states) must satisfy the relationships between the effective parameters K, T_{C} and P_{ON} (left, data from NC13 + early NC14, see Fig. S12A). Using the class of models in A, we can map the effective parameters onto the single copy parameters by satisfying the following equations: $T_{\mathrm{OFF}} = T_0$, $T_{\mathrm{ON}} = T_{1|2}$ and $KP_{\mathrm{ON}} = K^{(1)}P(1) + 2K^{(1)}P(2)$. Doing so, we find expressions for k_{ON}, k_{OFF} and $K^{(1)}$, as functions of the sole free parameter η. We have thus reduced a four-parameters model into a single-parameter one, which can easily be tested against data (see C,D and E). (C) Variance (2nd cumulant), 3rd cumulant and 4th cumulant of single allele transcription rate r as a function of mean transcription rate R in NC13 (square) and early NC14 ($7.5 \leq t < 20.5$ min; circle). The single allele transcription rates are estimated within 2-min-intervals and the cumulants are normalized by $\max R = R_{\max} = 15.77$ mRNA/min. Data color code as in Fig. S3. The dashed line corresponds to the Poisson limit (i.e., a single gene copy that is constitutively transcribed), whereas the dotted and solid curves are predictions made by the models in A that satisfy the derived bursting relationships in B. The dotted curve corresponds to independent sister chromatids ($\rho = 0$), the solid black line to slightly correlated sister chromatids ($\rho = 0.2$) and the solid gray line to fully correlated sister chromatids ($\rho = 1$). We immediately see that not accounting for pair of sister chromatids, or equivalently only considering highly correlated pair $\rho \sim 1$, provides a poor explanation for the data. On the contrary, models that include small correlations between chromatids $\rho \sim 0.2$ provide a good match to the data. The predicted cumulants are computed by sampling from each model to account for the Pol II footprint estimated around 60bp. (D) Distribution $P(r|R)$ of single allele transcription rates estimated within 2-min-intervals in both NC13 (color dotted lines) and early NC14 (color solid lines). These distributions are computed over all the nuclei from time points and AP bins whose mean transcription rate R corresponds either to a low $[2.1, 3.2]$, mid $[7.5, 8.5]$ or high transcription level $[12.8, 13.9]$ (as in Fig. S3). Black dashed line corresponds to the Poisson distribution for a single constitutive gene. Solid black and gray lines are predicted distributions by the models as in C (slightly correlated and fully correlated sister chromatids, respectively). While the model with fully correlated sister chromatids fails to account for the empirical distributions at mid and high R, a small amount of correlation ρ leads to a good match. (E) Survival function of elapsed time between successive Pol II initiation events (inter-initiation intervals) estimated within 2-min-intervals in both NC13 (color dotted lines) and early NC14 (color solid lines). These survival functions (1-"the cumulative distribution") are computed over the same nuclei, time points and AP bins as in D. The black dashed line corresponds to the Poisson for a single constitutive gene. Solid black and gray lines are predicted survival functions by the models as in C and D. A model describing a pair of slightly correlated bursting sister chromatids generate survival functions that mimic closely the data on a 2-min-interval (over which the system can be considered roughly stationary, i.e. $T_{\mathrm{C}} \sim 1.5$ min)

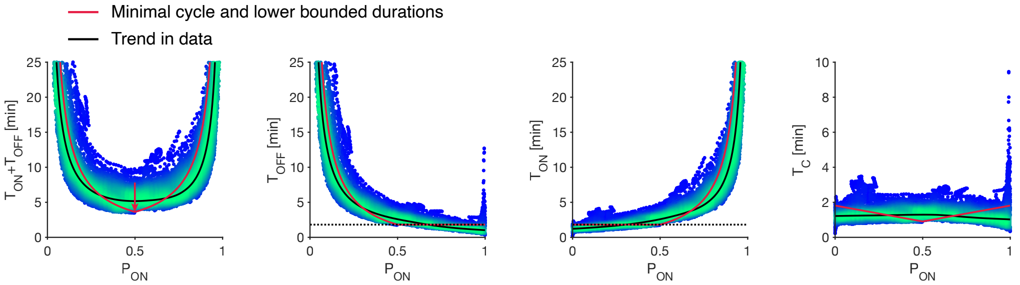

FIG. S17. **A bursting regime with a minimal ON-OFF cycle recapitulated the data.** An ON-OFF transcriptional regime, with a lower limit on these period durations and a minimal mean cycle duration $T_{\mathrm{ON}} + T_{\mathrm{OFF}} = 1/F$ shown in red, closely recapitulates the observed bursting relationships in black. The horizontal dotted line corresponds to a lower bound of 1.8 min providing the best-fit to the data. An allele at mid-activity level ($P_{\mathrm{ON}} = 0.5$) minimizes $T_{\mathrm{ON}} + T_{\mathrm{OFF}}$. Various ON-OFF combinations can yield $P_{\mathrm{ON}} > 0.5$, but increasing OFF periods prolong encoding P_{ON} compared to solely increasing ON durations.

SUPPLEMENTAL VIDEOS

Video V1-V4. Representative videos of 4 gap gene transcription rate measurements: for *hb*, *Kr*, *kni*, and *gt* (female), respectively. Anterior is on the left. We measured the dorsal side of the embryos. Red channel shows nuclei marked by Histone-RFP label; green channel shows the MCP-GFP signal, in particular highlighting one site of nascent transcription in each nucleus when it binds to MS2 stem-loops (see Methods). Each video was mean projected in the axial (z) direction. Projected image size is about 210x120 μm^2. Timestamp is in units of minutes. The video display frame rate is 15 Hz, which is equivalent to 2.5 min of imaging time per display second. Video contrast was adjusted for better visualization.

Video V5. The temporal progression of transcriptional activity along developmental time. X-axis: normalized embryo length (0-1). (Top) The mean activity directly from the MS2 signal. (Bottom) Calculated mean transcription rate (Methods Section 5.2) that is no longer gene length dependent.

Video V6. The temporal progression of relative protein abundance along developmental time. (Top) Protein accumulation predicted from measured mean transcription rate based on a simple model. (Bottom) Previously measured protein patterns from carefully staged gap gene antibody staining [31]. Major differences observed for *hb* are likely related to maternal mRNAs, whose contribution is not observed in our live measurements reflecting only zygotic gene expression.

Video V7. The mean transcription rate for wildtype *hb*-MS2 (dark blue) and its distal enhancer deletion mutant (light blue).

Video V8. The mean transcription rate for wildtype *kni*-MS2 (green) and its distal enhancer deletion mutant (dark yellow-green).

Video V9. The mean transcription rate for wildtype *kni*-MS2 (green) and its expression in the background of *hb* null mutant (light yellow-green).

Gene	Annotated length [bp]	Physical length L_g [bp]	Effective length $\tilde{L}$ [bp]
hb-intron	4464	4464	3851
hb-3utr	3516	3516	2903
Kr-intron	3765	4565	3952
Kr-3utr	1915	2715	2102
kni	3049	3849	3236
gt	2940	3740	3127

TABLE S1. Gene length of the measured gap genes. The annotated gene length was obtained from UCSC Genome Browser (BDGP Release 6 + ISO1 MT/dm6) Assembly and it includes the length of the inserted MS2 cassette. The physical gene length is obtained by further adding 800bp to all genes except *hb*, and it accounts for the retention of elongated nascent transcripts at the sites. This extra length was estimated from dual color smFISH measurements [23]. The effective gene length is calculated from the physical gene length according to Equ. 4.

Gene	$\sigma^2_{\text{img}}/\sigma^2$	T_d [min]
TFF1	0.21 ± 0.02	10.5 ± 0.8
CANX	0.53 ± 0.04	7.4 ± 1.5
DNAJC5	0.29 ± 0.02	10.4 ± 0.9
ERRFI1	0.49 ± 0.06	15.7 ± 2.6
KPNB1	0.26 ± 0.03	11.8 ± 1.4
MYH9	0.24 ± 0.03	6.2 ± 0.8
RAB7A	0.25 ± 0.02	12.4 ± 1.3
RHOA	0.32 ± 0.03	9.0 ± 1.2
RPAP3	0.62 ± 0.04	7.9 ± 1.7
SEC16A	0.39 ± 0.04	15.5 ± 2.1
SLC2A1	0.15 ± 0.03	14.9 ± 2.0

TABLE S2. Estimated fractional imaging noise and dwell-time for the 11 human genes from [9, 45].

Gene	R [1/min]	K [1/min]	P_ON	T_OFF [min]	T_ON [min]	T_C [min]
TFF1	0.027 ± 0.001	0.54 ± 0.02	0.050 ± 0.016	71.3 ± 11.7	4.4 ± 0.7	4.1 ± 0.6
CANX	0.037 ± 0.017	0.55 ± 0.03	0.065 ± 0.035	37.7 ± 11.2	2.7 ± 0.6	2.5 ± 0.5
DNAJC5	0.084 ± 0.016	0.52 ± 0.01	0.163 ± 0.029	17.0 ± 1.9	3.5 ± 0.4	2.9 ± 0.3
ERRFI1	0.008 ± 0.004	0.57 ± 0.03	0.009 ± 0.007	213.6 ± 44.7	2.8 ± 1.2	2.8 ± 1.2
KPNB1	0.101 ± 0.041	0.55 ± 0.04	0.182 ± 0.067	19.9 ± 4.4	4.7 ± 0.8	3.8 ± 0.5
MYH9	0.081 ± 0.033	0.51 ± 0.02	0.157 ± 0.056	24.9 ± 4.6	5.5 ± 1.2	4.5 ± 0.8
RAB7A	0.052 ± 0.017	0.53 ± 0.02	0.097 ± 0.032	25.3 ± 5.7	3.0 ± 0.5	2.6 ± 0.4
RHOA	0.117 ± 0.032	0.51 ± 0.01	0.231 ± 0.060	12.1 ± 2.4	3.8 ± 0.6	2.8 ± 0.3
RPAP3	0.011 ± 0.005	0.57 ± 0.03	0.018 ± 0.012	117.2 ± 31.3	2.4 ± 0.6	2.3 ± 0.5
SEC16A	0.036 ± 0.015	0.55 ± 0.02	0.065 ± 0.026	37.1 ± 9.7	2.9 ± 0.5	2.6 ± 0.4
SLC2A1	0.067 ± 0.029	0.53 ± 0.03	0.122 ± 0.049	32.9 ± 8.0	5.5 ± 1.6	4.5 ± 1.2

TABLE S3. Estimated bursting parameters using our deconvolution approach for the 11 human genes from [9, 45].

Gene	F [1/min]	B
TFF1	0.014 ± 0.002	2.33 ± 0.39
CANX	0.026 ± 0.007	1.50 ± 0.28
DNAJC5	0.049 ± 0.005	1.79 ± 0.19
ERRFI1	0.005 ± 0.001	1.59 ± 0.58
KPNB1	0.042 ± 0.007	2.62 ± 0.53
MYH9	0.034 ± 0.005	2.83 ± 0.66
RAB7A	0.037 ± 0.007	1.58 ± 0.25
RHOA	0.064 ± 0.009	1.91 ± 0.27
RPAP3	0.009 ± 0.002	1.33 ± 0.26
SEC16A	0.026 ± 0.007	1.58 ± 0.30
SLC2A1	0.027 ± 0.005	2.94 ± 0.87

TABLE S4. Estimated burst frequency and size using our deconvolution approach for the 11 human genes from [9, 45].
