# Supplementary material for: Common bursting relationships underlie eukaryotic transcription dynamics
Source: ArXiv. 2023 Apr 18:arXiv:2304.08770v1. Preprint. [Version 1] (PMC10153294)
Supplement: Supplementary file 1 [file NIHPP2304.08770v1-supplement-1.pdf]

SUPPLEMENTAL FIGURES

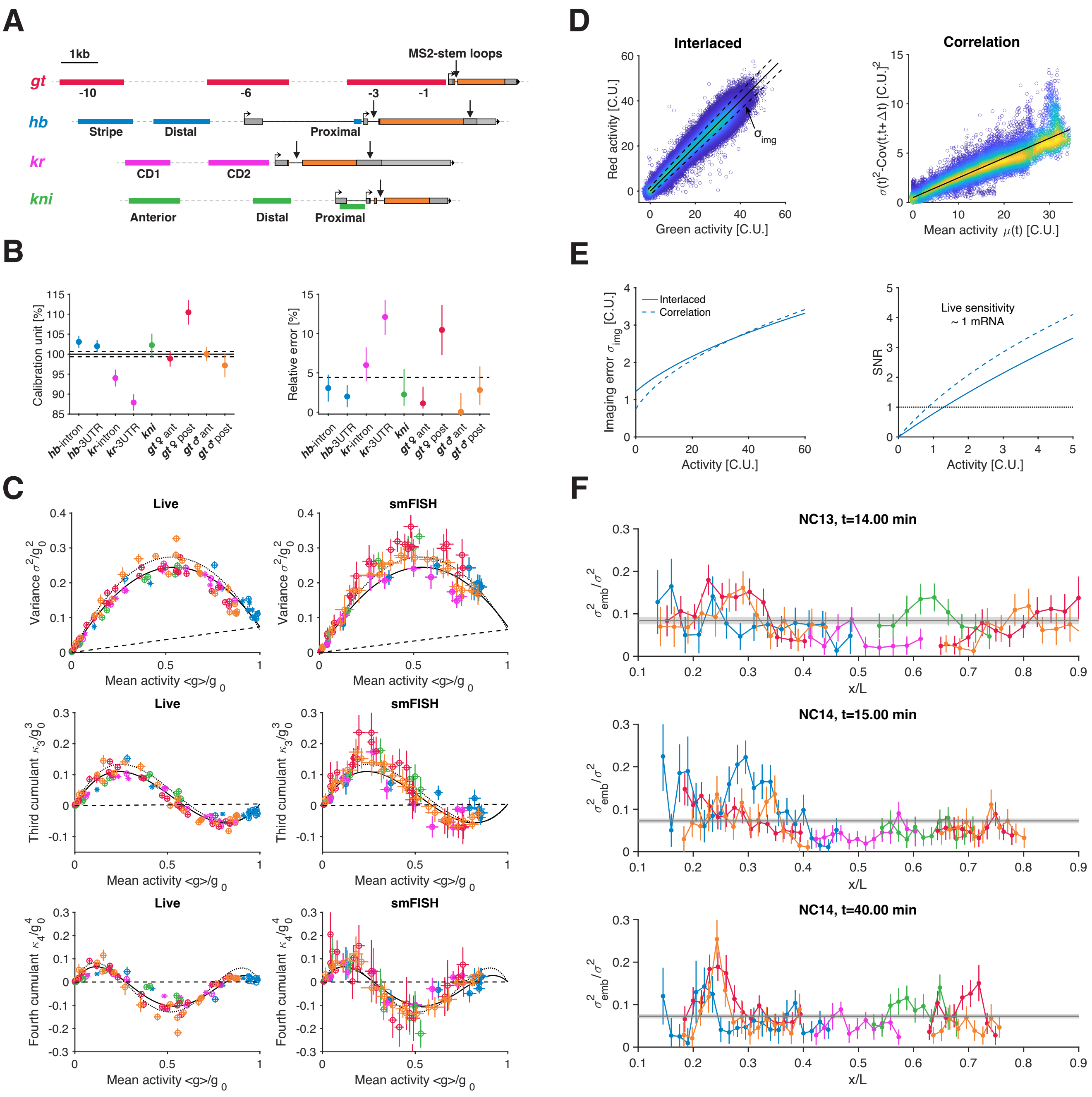

FIG. S1. Signal calibration, measurement error and embryo-embryo variability. [Caption see next page.]

FIG. S1. **Signal calibration, measurement error and embryo-embryo variability.** (A) The four trunk gap genes, *giant* (*gt*), *hunchback* (*hb*), *Kruppel* (*Kr*) and *knirps* (*kni*) were imaged using the MS2/PP7 stem-loop labeling systems. Stem-loop cassettes (vertical black arrow) were inserted either in the first/second intron or in the 3'UTR of each gene. Gap genes harbor different *cis*-architectures as characterized by the number of promoters, number of enhancers (color boxes), and composition of these *cis*-regulatory elements (TF binding motifs, core promoter elements, etc.). (B) Relative calibration unit (left) and relative error (right) for each gap gene construct related to Fig. 1C. The conversion of the live signal to absolute units is performed by comparison to a smFISH-based measurement. Calibration was performed by matching the mean full embryo length transcriptional activity profiles (reconstructed by averaging over all nuclei in 2.5% AP bins, within a 5 min time window in NC13) measured by live imaging to those previously measured with smFISH (Zoller et al., 2018). (Left) The procedure was performed using all measured gap profiles at once, leading to a final calibration unit (horizontal back line, dashed lines are plus/minus one standard error). We then repeated the procedure for each individual construct separately (color circles), and the derived units are expressed in percent of the global fit. (Right) Relative error for the calibration unit of each individual gene construct with respect to the global unit and mean relative error (dashed line), which is below 5%. Error bars are 68% confidence intervals. (C) Comparison of higher cumulants versus mean activity relationships obtained by live imaging and smFISH measurements; right column panels are reproduced from Figure 3B-D in Zoller et al. (2018), left column panels are from live data analyzed equivalently. Live cumulants of transcriptional activity (mean, variance, 3rd and 4th cumulant) are estimated over all nuclei in 2.5% AP bins, within a 5 min time window in NC13. Cumulants are converted from equivalent cytoplasmic mRNA units (C.U.) to Pol II counts for a single gene copy of average length (3.3 kb). The cumulants are normalized with respect to g_0 defined as the intercept of the Poisson background (dashed line) and the polynomial fit to the data (black solid line for live and doted for smFISH). The number g_0 can be interpreted as the mean number of Pol II on a 3.3 kb long gap gene at maximal activity. We get $g_0 = 13.6$ for live and $g_0 = 15.2$ with smFISH measurements, a difference of 12%. Overall, the higher cumulants versus mean relationships obtained from live (left column) and from smFISH (right column) are extremely close (black solid versus dotted line), confirming the quantitative nature and the proper calibration of our live assay. Two independent methods leading to the same quantitative conclusions validate each other reciprocally. It strongly suggests that our synthetic modifications of the endogenous gap gene loci have no currently measurable effect on the transcriptional output of the system. (D) Two independent methods to assess the imaging error. (Left) An interlaced cassette of alternating MS2 and PP7 stem-loops, labeled with two differently colored coat proteins (MCP-GFP and PCP-mCherry), is inserted in the first intron of *Kr*. In absence of imaging error, the transcriptional activity in the green and red channels when calibrated to C.U. should perfectly correlate (on the diagonal). We fitted the spread σ_{img} orthogonal to the diagonal (black line, slope one) to characterize the imaging error; assuming σ^2_{img} scales as $\sigma^2_b + \alpha I$ with mean intensity I, where σ^2_b is the background noise and αI a Poisson shot noise term. The resulting fit for σ_{img} is highlighted by the dashed lines (plus minus one std around the diagonal). (Right) Imaging error estimation from the single allele transcriptional time series (with the assumption that the measured transcriptional fluctuations result from the sum of uncorrelated imaging noise and correlated noise due to the elongation of tagged nascent transcripts). We computed the time-dependent mean activity $\mu(t)$, variance $\sigma^2(t)$ and covariance between consecutive time points $\text{Cov}(t, t + \Delta t)$ (where Δt is 10 s), over all nuclei within 1.5–2.5% AP bins for all measured genes. The uncorrelated imaging variability σ^2_{img} is then approximated by $\sigma^2_{\text{img}} - \text{Cov}(t, t + \Delta t)$, which is plotted as a function of $\mu(t)$ for all time points. We characterized σ^2_{img} by fitting the data with a line $\sigma^2_b + \alpha\mu$. Fitting results are shown in E. (E) Our two estimates for the imaging error (interlaced dual-color construct (solid line) and correlation-based approach (dashed line)) are consistent. The signal-to-noise-ratio (SNR), defined as μ/σ_{img}, is close to one (dotted line) when $\mu \approx 1$, indicating that the sensitivity of our live measurements is close to one mRNA molecule. (F) Fractional embryo variability profiles as a function of AP position and developmental time, for all gap genes. We define embryo variability σ^2_{emb} as the variance of the mean activity across embryos, and report the fractional embryo variability as the ratio $\sigma^2_{\text{emb}}/\sigma^2$, where $\sigma^2 = \sigma^2_{\text{emb}} + \sigma^2_{\text{nuc}}$ is the total variance, and σ^2_{nuc} corresponds to the transcriptional allele-to-allele noise across nuclei. Overall, the fractional embryo variability is $\sim 10\%$, meaning that most of the variability arises from σ^2_{nuc}. Thus, together D, E, and F show that $\sigma^2_{\text{tot}} = \sigma^2_{\text{img}} + \sigma_{\text{emb}} + \sigma^2_{\text{nuc}}$ is a good proxy for σ^2_{nuc}, which is the relevant noise contribution that contains all the bursting phenomenology.

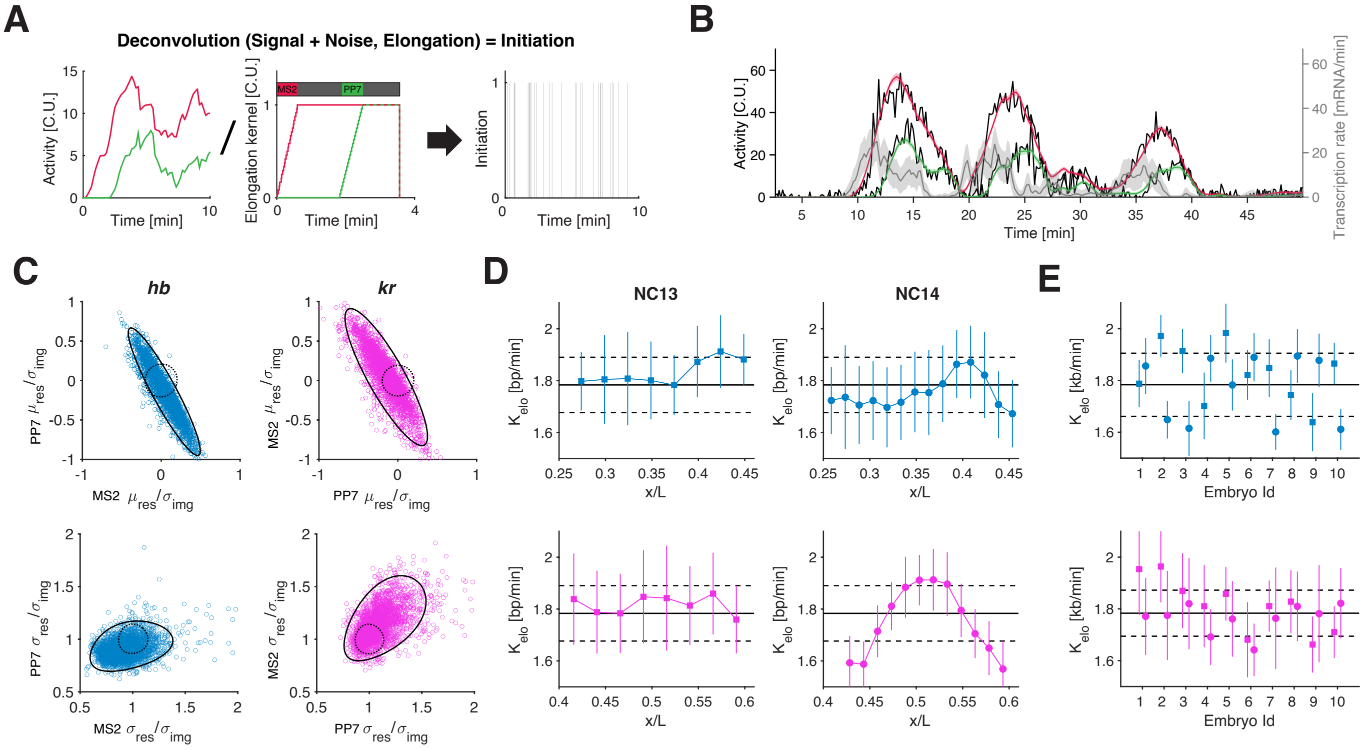

FIG. S2. **Dual color measurements to validate single-cell deconvolution and measure elongantion rate.** (A) Validation of the kernel assumption for the deconvolution of initiation events from single allele transcription time series using a dual-color (confocal) imaging approach for *hb* and *Kr*. For *hb* (*Kr*), we generated fly lines with dual-insertions of a MS2 (PP7) stem-loop cassette in the respective first intron and a PP7 (MS2) stem-loop cassette in the 3'UTR. In both cases, the two cassettes were labelled using two different colors (MCP-GFP green and PCP-mCherry red). Since the two signals are correlated through the elongation process, the simultaneously measured pair of time series has a further constrained set of underlying initiation configurations and represents thus a good test for the approach. To deconvolve single allele dual color time series together (i.e., a single train of polymerases needs to match two signals), using two kernels modeling each loop-cassette location and satisfying our key assumptions (i. constant and deterministic elongation rate; ii. no Pol II pausing/dropping in gene body; iii. fast termination). In addition, the dual-color strategy allows estimation of the average elongation rate from the overall delay between the two signals (using the known genomic distance between the MS2 and PP7 insertion sites). (B) Dual-color signal reconstructed from deconvolved single allele transcription time series (black lines for raw measured data). single allele transcription rate (gray line with one std envelope) is deconvolved from the single depicted pair of measured time series (black lines). The signal (red and green lines with one std envelope) is devoid of imaging noise (as it was modeled from Fig. S1D during the deconvolution process) and is reconstructed by convolving back the resulting transcription rate with the kernel of each channel. Qualitatively, the signal (color) matches well (see C) the measured time series (black) in strong support of our kernel assumptions. (C) Distribution of residuals from the dual-color reconstruction. We quantified the mean and standard deviation of the normalized residuals, i.e., of the difference between the measured signal (black in H) and the reconstructed signal (color in B) divided by the standard deviation of the imaging noise, for each recorded individual allele (for *hb* $N = 2666$ (blue) and for *Kr* $N = 2594$ (pink)). Overall, the dispersion of the means and standard deviations of normalized residuals (black line, 95% confidence ellipse) is close to the expected dispersion of a perfect model (dotted line, 95% confidence ellipse). (D-E) Estimated elongation rate K_{elo} from dual-color measurements. (D) Average elongation rate computed over nuclei across 10 embryos as a function AP position (both *hb* (blue) and *Kr* (pink)) in NC13 (square) and NC14 (circle), with error bars representing one standard deviation across the embryo means. (E) Average elongation rate computed for individual embryos (color code and symbols as D), with error bars representing the standard deviation across the means over positions. The elongation rate is globally conserved across genes and nuclear cycles, with $K_{\mathrm{elo}} = 1.8 \pm 0.1$ kb/min (corresponding to the mean across embryos (black line) plus/minus one standard deviation (dashed line).

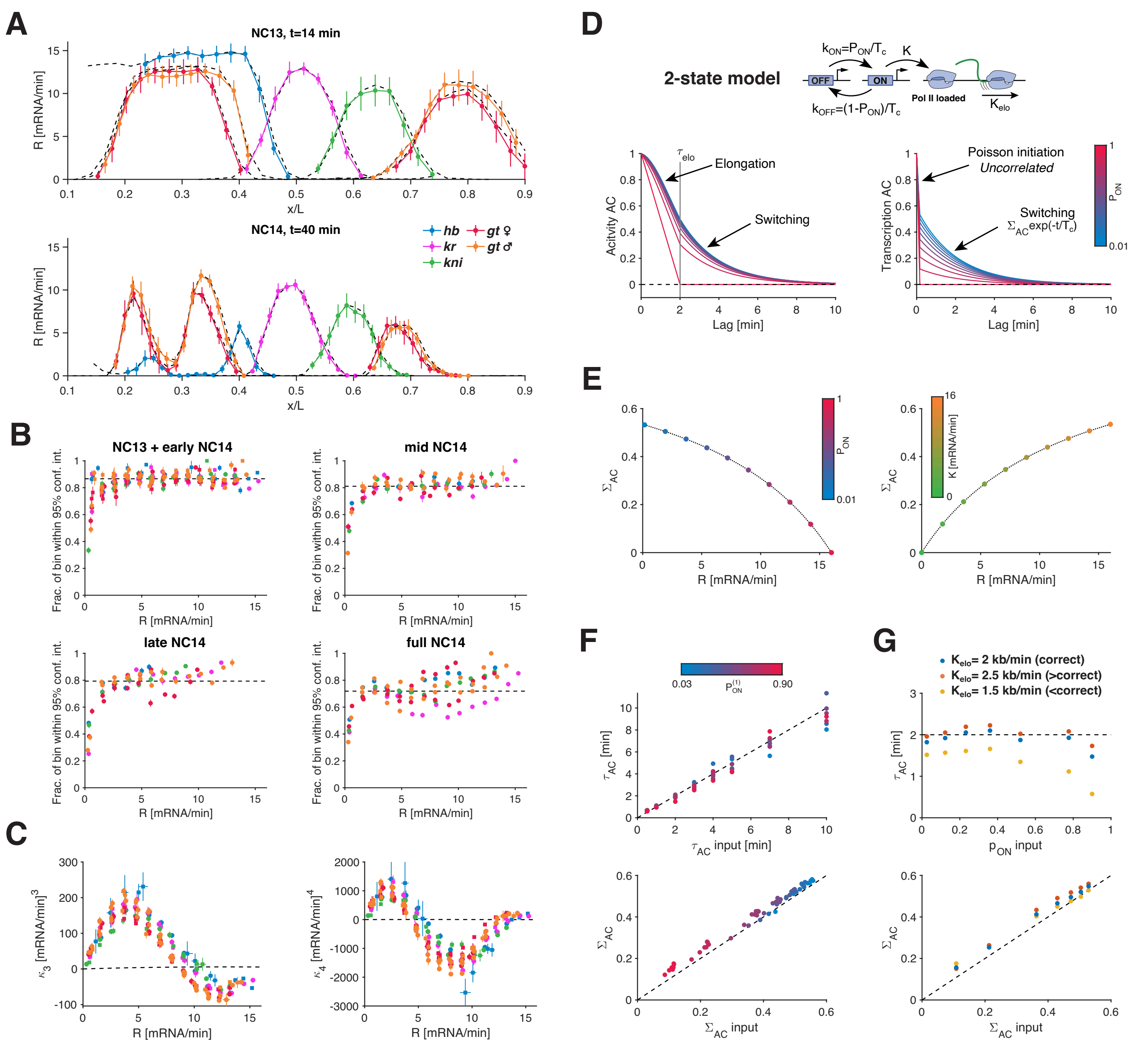

FIG. S3. Transcription rate fluctuations reveal key bursting characteristics. [Caption see next page.]

FIG. S3. **Transcription rate fluctuations reveal key bursting characteristics.** (A) Snapshots of the gap gene mean transcription rate R as a function of AP position in late NC13 and at 40 min in NC14. Gap gene profiles (color) are obtained by averaging the deconvolved single allele transcription rate over all nuclei within each AP bin (width of 2.5% and 1.5% embryo egg length in NC13 and NC14 respectively) and at each time point (10 s temporal resolution). The black dashed lines correspond to the mean activity of each gap gene at the same position and time normalized by the effective elongation time. Both the colored and dashed profiles agree, justifying our deconvolution approach. Error bars are one standard deviation across embryo means. Overall, we have effectively deconvolved $N_g = 7$ "genes" (4+gt male and female and anterior and posterior regions), over $N_t = 362$ time points (NC13+NC14), across N_x =9–18 positions, leading to a total of $33'214$ bins, each averaging ~ 200 nuclei (with a single allele per nucleus). (B) Fraction of spatial and temporal bins whose single allele transcription rate distribution $P(r)$ is consistent with the conditional transcription rate distribution $P(r|R)$ determined by pooling nuclei over multiple bins at a given mean transcription rate R. We computed the 95% confidence interval on the cumulative distribution of $P(r|R)$ and checked for all the underlying bins at a given R whether their individual cumulative distribution was within the overall confidence interval. We repeated this process for four distinct developmental time windows: NC13 ($6.5 \leq t$ min after mitosis) plus early NC14 ($7.5 \leq t < 20.5$ min), mid NC14 ($20.5 \leq t < 34.5$ min), late NC14 ($34.5 \leq t < 48$ min), and a wider NC14 window ($7.5 \leq t < 48$ min). Overall, bins that share similar R within the same time window have very similar $P(r)$ distribution (median given by dashed line over 80%), which justifies the pooling of these bins. On the other hand, when pooling bins over the whole NC14 we observe further dissimilarities between bins, suggesting that $P(r|R)$ might moderately change over time. (C) 3rd cumulant and 4th cumulant of single allele transcription rate as a function of mean transcription rate R in NC13 (square) and early NC14 ($7.5 \leq t < 20.5$ min; circle). The single allele transcription rates are estimated within 1-min-intervals, highlighting a strong departure of the variance from a constitutive Poisson regime (dashed line, κ_3 and $\kappa_4 = R$). All gap genes follow the same trend suggesting a common bursting regime. (D) Interpreting auto-correlation function using the 2-state model of transcriptional bursting. In this model for a single gene copy (top), the gene promoter switches stochastically between an OFF and ON state with rates k_{OFF} and k_{ON}, where in the latter Pol II can be loaded at rate $K^{(1)}$ and elongate at rate K_{elo}. Of note, the superscript (1) specifies the parameter for a single gene copy. The auto-correlation functions displayed here are computed from the model using a switching correlation time $T_{\mathrm{C}}^{(1)} = 1/(k_{\mathrm{OFF}} + k_{\mathrm{ON}}) = 2$ min, a Pol II elongation time $\tau_{\mathrm{elo}} = L_g/K_{\mathrm{elo}} = 2$ min (where L_g is the gene length) and an initiation rate $K^{(1)} = 8$ mRNA/min; the steady state ON-probability $P_{\mathrm{ON}}^{(1)} = k_{\mathrm{ON}}/(k_{\mathrm{ON}} + k_{\mathrm{OFF}})$ varies from 0 to 1 (i.e., blue to red color code, fraction of nuclei in ON state or fraction of time a nucleus is in ON state). In principle, promoter switching (generating bursts) leads to temporal correlations in the transcriptional activity time series (Activity AC). However, from the raw live measurements, these correlations are hard to distinguish from the ones introduced by elongation (left), specifically when the switching correlation time $T_{\mathrm{C}}^{(1)}$ is close to or smaller than the elongation time τ_{elo}. Instead, performing auto-correlation analysis on deconvolved single allele transcription rates resolves the switching correlations (Transcription AC, right), since correlations due to elongation have been removed. Thus, the switching correlation time $T_{\mathrm{C}}^{(1)}$ can be estimated by fitting an exponential to the decay of the Transcription AC. (E) Expected effect of the ON-probability (left) and Pol II initiation rate (right) on the magnitude of the correlated noise, Σ_{AC}. Computation of Σ_{AC} using same parameters as in D. (Left) As $P_{\mathrm{ON}}^{(1)}$ increases, the transcription rate $R = 2K^{(1)}P_{\mathrm{ON}}^{(1)}$ increases (here for 2 sister chromatids), and Σ_{AC} decreases until it vanishes at $P_{\mathrm{ON}}^{(1)} = 1$ (Poisson regime). This behavior is consistent with our data, see Fig. 2E. (Right) At fixed $P_{\mathrm{ON}}^{(1)}$ ($P_{\mathrm{ON}}^{(1)} = 0.5$) and varying initiation rate $K^{(1)}$, the Σ_{AC} increases with growing transcription rate R. This behavior is the opposite of what we observed in our data (Fig. 2E). In both cases (Left and Right) the dotted line corresponds to the exact solution for Σ_{AC}, which is well-approximated by $\Sigma_{\mathrm{AC}} = \Delta t K^{(1)}(1 - P_{\mathrm{ON}}^{(1)})/(1 + \Delta t K^{(1)}(1 - P_{\mathrm{ON}}^{(1)}))$, where $\Delta t = 10$ s corresponds to the data sampling time. (F) Correlation time and correlated noise magnitude are properly retrieved after single allele deconvolution. Using the Gillespie algorithm, we generated simulated data ($N = 200$ and 50 min long cell recordings per condition) according to the two-state model in D. For each input condition ($P_{\mathrm{ON}}^{(1)}$ from 0.03 to 0.9 and $T_{\mathrm{C}}^{(1)}$ from 0.5 to 10 min), we performed single allele deconvolution and computed the auto-correlation on the resulting transcription rates. We estimated the correlation time $T_{\mathrm{C}}^{(1)}$ and the magnitude Σ_{AC} by fitting exponential. Both parameters are properly retrieved with minimal biases. Color code stand for $P_{\mathrm{ON}}^{(1)}$ and dashed line for slope 1. (G) Estimating deconvolution biases due to elongation rate measurement bias. As in F, we generated simulated data ($P_{\mathrm{ON}}^{(1)}$ from 0.03 to 0.9 and $T_{\mathrm{C}}^{(1)} = 2$ min) and aimed to deconvolve the data with elongation rate higher (orange dot) or lower (yellow dot) than the correct value (blue dot, used to generate the data as in F). Overall, the estimated parameters are estimated correctly, with larger biases at large $P_{\mathrm{ON}}^{(1)}$ and when underestimating the elongation rate (yellow).

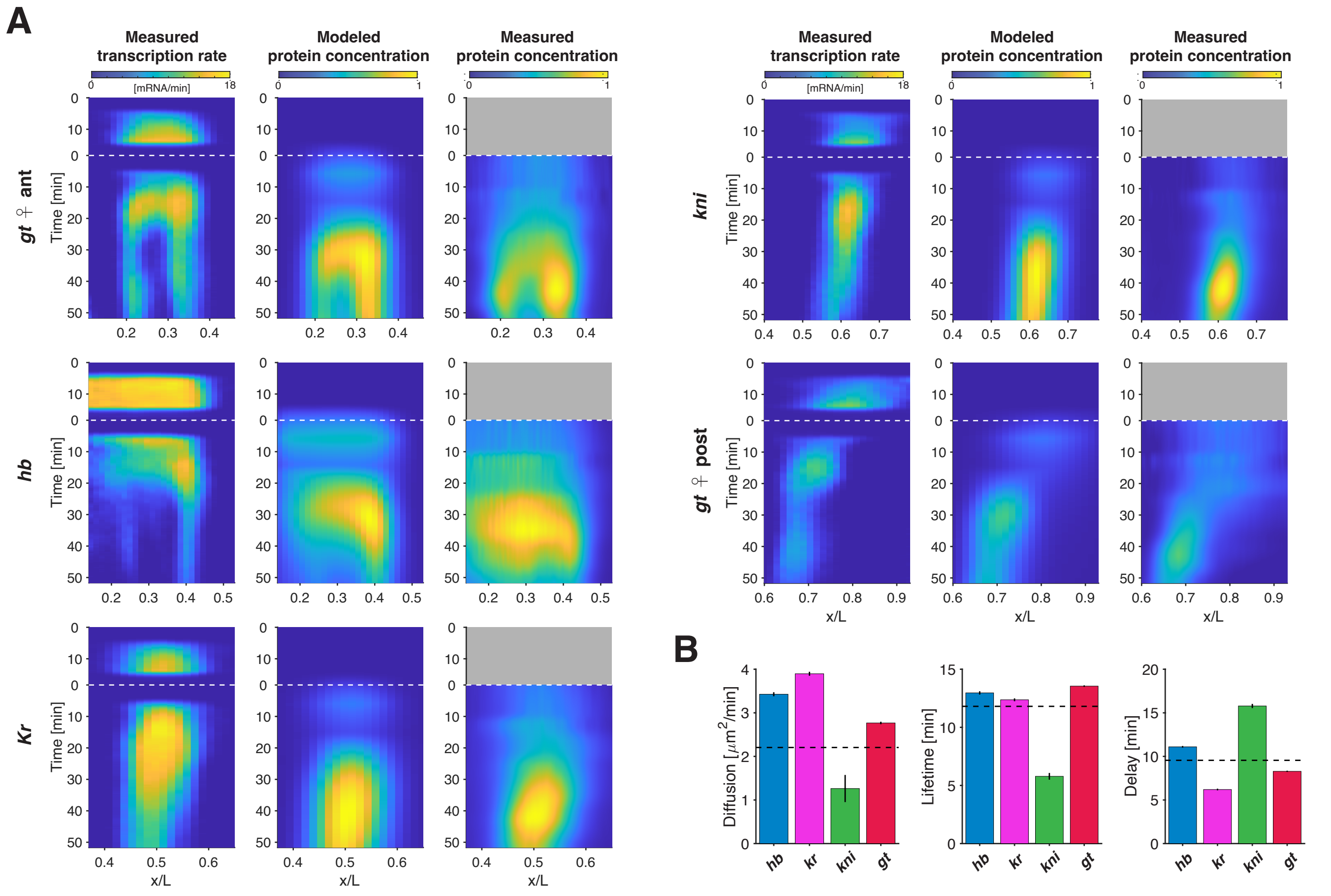

FIG. S4. **Transcription rate explains dynamic pattern establishment.** (A) A simple modeling attempt for protein accumulation from mean transcription rate measurements. The mean transcription rate (left column) across space and time is estimated by normalizing the measured mean activity by the elongation time and applying a minor correction for the delay ($<$ 1 min) resulting from the loop insertion location. Horizontal white dashed lines correspond to the transition (mitosis) from NC13 to NC14. Protein accumulation (middle column) is computed from the mean transcription rate as the convolution of the latter with a kernel modeling protein decay, diffusion, and delay due to mRNA export, translation, and nuclear import. This simple model introduces three free parameters, a protein lifetime, a diffusion constant, and a time-delay (see B). These three parameters were set by minimizing the mean squared error with previously measured protein patterns (right column; Dubuis et al., 2013). Small residual deviations between middle and right columns might be due to post-transcriptional regulatory processes that our simple model does not account for. (B) Parameters estimated for the modeled accumulation of effective proteins as described in A. The three parameters were either estimated for each gene separately (color bars) or all genes together (dashed lines, used for middle column in A). Overall, the effective parameters are mostly in line with previous estimates [64].

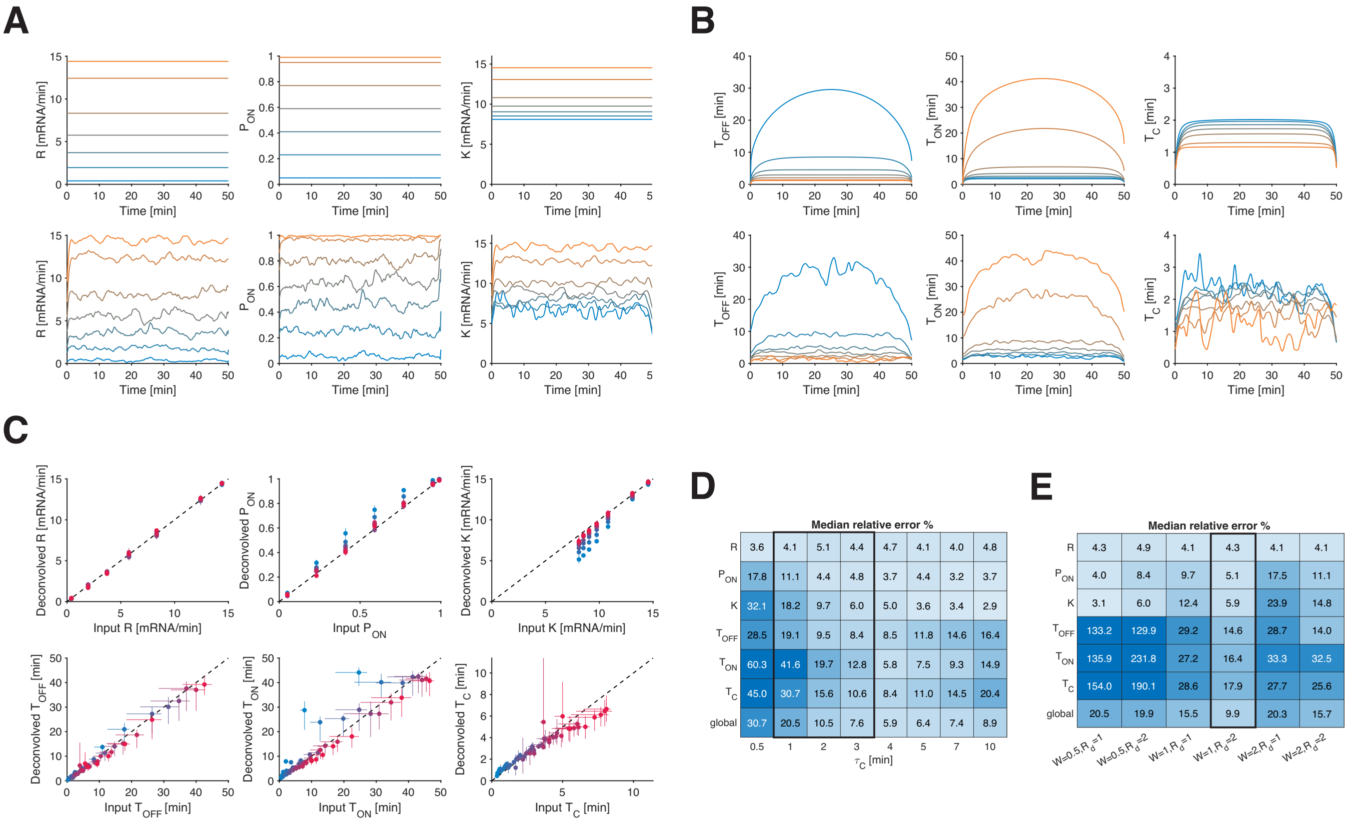

FIG. S5. **Validation of transcriptional parameter estimation using stationary simulated data.** Burst calling permits effective estimation of transcriptional parameters. For each set of input parameters ($K^{(1)}$, $T_C^{(1)}$, $P_{\rm ON}^{(1)}$), we generated simulated data (200 alleles and 50 min long time series at 10 s intervals and with the same imaging noise as measured in real data). Using the two-state model (Fig. S3D) and the Gillespie algorithm, we generated time series for each individual sister chromatid. We summed the initiation events of both chromatids assuming independence. The resulting initiation events were convolved with the elongation kernel to generate synthetic single allele signal data over which measurement noise was added. We performed single allele deconvolution and burst calling as described (Fig. 3A) to estimate the effective mean transcription parameters (R, K, $P_{\rm ON}$, $T_{\rm ON}$, $T_{\rm OFF}$ and $T_{\rm C}$) for each simulated set of alleles. (A-B) Comparison between the theoretically expected (top row) and the estimated from burst calling effective parameters (bottom row) as a function of time, using stationary (constant in time) input parameters ($T_C^{(1)} = 2$ min, $K^{(1)} = 8$ mRNA/min and $P_{\rm ON}^{(1)}$ varies from 0.03 to 0.9 that is color coded from blue to orange). Overall, the estimated effective parameters are recovered very well. (A) Despite sampling fluctuations, the constancy of the parameters in time is properly preserved (bottom row), as it should be based on input (top row). (B) Despite the stationary nature of the input parameters $T_C^{(1)}$ and $P_{\rm ON}^{(1)}$, biases for $T_{\rm ON}$, $T_{\rm OFF}$ and $T_{\rm C}$ (top row) are expected due to the finite length of the simulated time series (censoring), especially noticeable near the beginning and the end, where the estimations are "bent" (the resulting mean time estimate cannot exceed the width of the time window used to perform the estimate). However, our parameter estimation (bottom row) is very much in line with the expected biases (top row). (C) Global comparison between expected and estimated parameters for the stationary case (input parameters are constant in time). The parameter estimation was performed on a large simulated data set that includes data in A and B ($T_C^{(1)} = 2$ min, $P_{\rm ON}^{(1)}$ from 0.03 to 0.9) and data for other values of $T_C^{(1)}$ comprised between $T_C^{(1)} = 0.5$ (blue dots) to $T_C^{(1)} = 10$ (red dots). Each dot results from one combination of input parameters ($K^{(1)}$, $T_C^{(1)}$, $P_{\rm ON}^{(1)}$) and corresponds to the median effective parameter and the error bars to the 68% confidence interval estimated over 50 min. Our deconvolution and burst calling approach leads to an excellent estimation of the effective parameters over a large range of $T_C^{(1)}$ and $P_{\rm ON}^{(1)}$ values, albeit with noticeable biases in $P_{\rm ON}$, K and $T_{\rm ON}$ when $T_C^{(1)}$ approaches 0.5 min (blue dots). Importantly, biases for the effective switching correlation time T_C are small, supporting our ability to detect its constancy in real data. (D) Summary of median relative error for each effective parameter estimated from the data in C as a function of input $T_C^{(1)}$. Parameter estimated from real data (Fig. S9B) suggests that $T_C^{(1)}$ lies within 1 and 3 min (black border). (E) Summary of median relative error for each effective parameter estimated from the whole data set in C as a function of the burst calling parameters. Burst calling depends on two free parameters: the time window W over which the rate is estimated, and the rate threshold R_d applied to call the burst (see Fig. 3A). Our default parameter values are $W = 1$ and $R_d = 2$, which should be close to optimal given the estimated correlation time of $\tau_{\rm AC} \sim 1$ min (Fig. 2F) and our measurement sensitivity of 1–2 mRNA. When testing the effect of different W and R_d values on the median relative error, our default choice leads to the lowest global relative error.

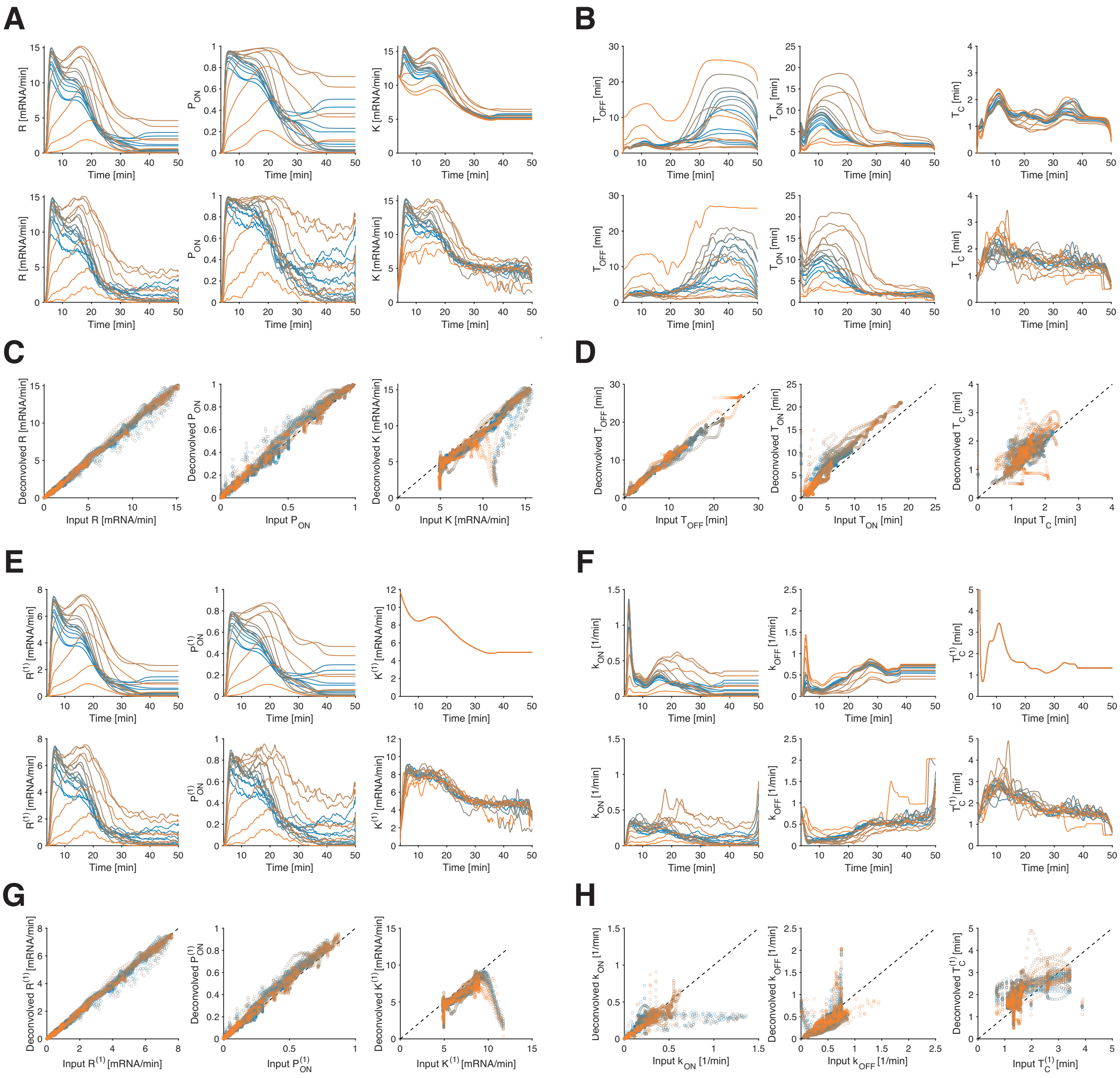

FIG. S6. **Validation of transcriptional parameter estimation using *hb*-like non-stationary simulated data.** Simulated data is generated as in Fig. S5, except that the input parameters are non-stationary (time-dependent). (A-B) Comparison between expected (A, B, top row) and estimated effective parameters (A, B, bottom row) for time-dependent input parameters (A, B, top row), mimicking the transcriptional output of *hb* in NC14. Color code (blue to orange) stands for virtual AP position. Estimated effective parameters tightly recapitulate the time-dependence of the expected parameters, allowing us to also capture the temporal regulation of the gap genes. (C, D) We show with this realistic test case that our estimated parameters (A, B, bottom row) using burst calling very closely match the expected effective ones (A, B, top row). Indeed, most of the time points (circles) lie on the line of slope one (dashed line), which strongly supports our ability to precisely characterize the transcriptional parameters from real data. (E–F) Comparison between input (E, F, top row) and burst calling estimated single-gene copy parameters (E, F, bottom row) as a function of time. The time-dependent input parameters (E, F, top row) were used to generate the synthetic *hb* data in A and B. Color code (blue to orange) stands for virtual AP position as in A and B. The estimated single-gene copy parameters were computed from the effective ones (A, B, bottom row) assuming the latter originate from two independent sister chromatids. Namely, we get $R^{(1)} = R/2$, $P_{\mathrm{ON}}^{(1)} = 1 - (1 - P_{\mathrm{ON}})^{1/2}$, $K^{(1)} = K(1+(1-P_{\mathrm{ON}})^{1/2})/2$, which are exact, and assuming steady state $T_{\mathrm{C}}^{(1)} = 2T_{\mathrm{C}}/(1+(1-P_{\mathrm{ON}})^{1/2})$, $k_{\mathrm{ON}} = P_{\mathrm{ON}}^{(1)}/T_{\mathrm{C}}^{(1)}$ and $k_{\mathrm{OFF}} = (1 - P_{\mathrm{ON}}^{(1)})/T_{\mathrm{C}}^{(1)}$. (G-H) Even though the single gene copy parameters are deeply buried in the data, our simple burst calling procedure still manages to recover them correctly. Some discrepancies are observed for k_{ON}, k_{OFF} and $T_{\mathrm{C}}^{(1)}$, mostly near the beginning when the transient after mitosis violates the near-steady-state assumption. But these are expected as the relationships $k_{\mathrm{ON}} = P_{\mathrm{ON}}^{(1)}/T_{\mathrm{C}}^{(1)}$ and $k_{\mathrm{OFF}} = (1 - P_{\mathrm{ON}}^{(1)})/T_{\mathrm{C}}^{(1)}$ are only valid for near-steady state.

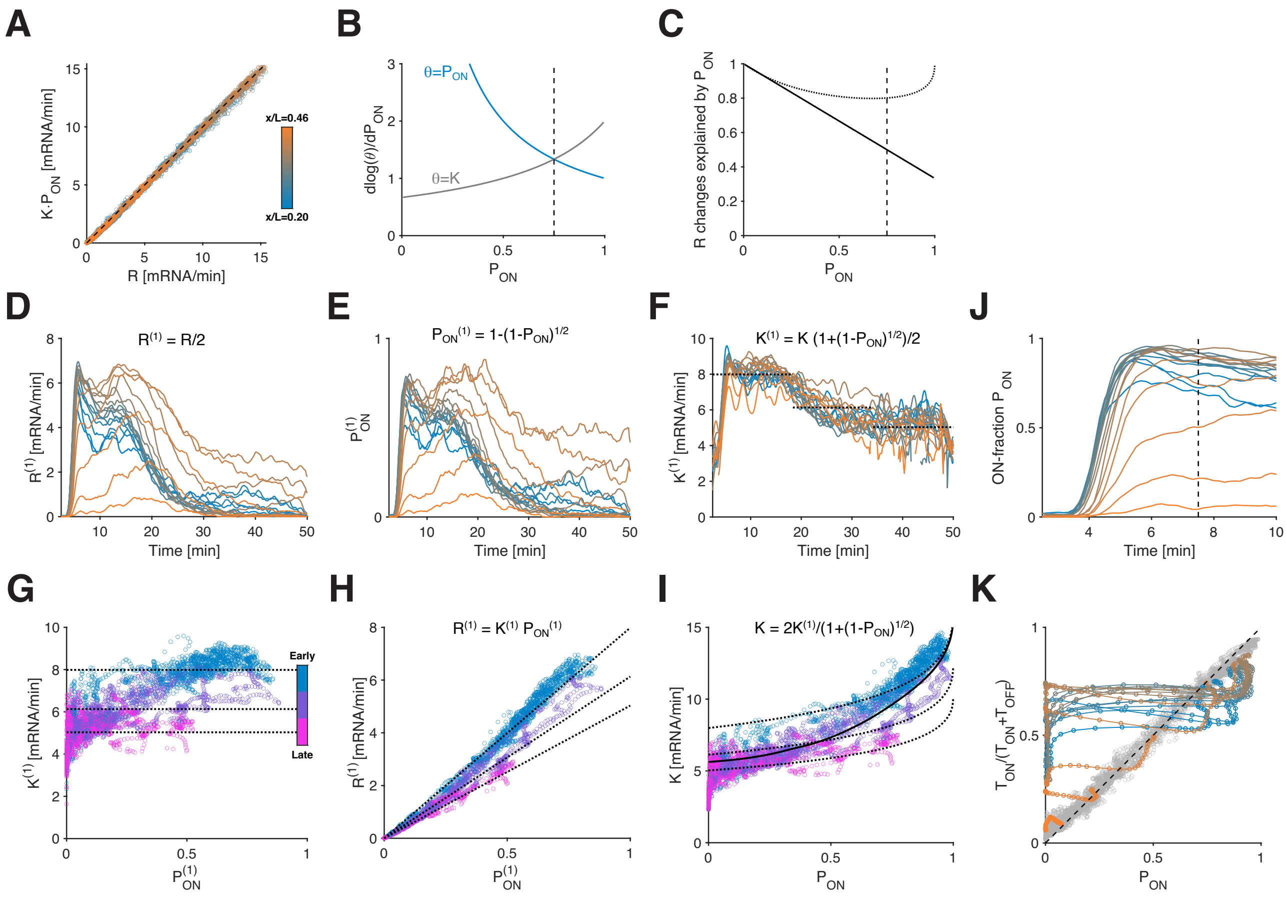

FIG. S7. Single gene copy parameter and transient for *hunchback* in NC14. [Caption see next page.]

FIG. S7. **Single gene copy parameter and transient for *hunchback* in NC14.** (A) Transcription rate R versus the product of the initiation rate K and the ON-probability $P_{\rm ON}$ for *hb* in NC14 at all time points and positions. The color code stands for AP position as in Fig. 3 and 4. As it should be by construction, R can be decomposed into the product of K and $P_{\rm ON}$. (B) Log-derivative with respect to $P_{\rm ON}$ of the controlling parameter K (gray) and P$P_{\rm ON}$ (blue) as a function of $P_{\rm ON}$. Since $R = K \cdot P_{\rm ON}$, $d\log K/dP_{\rm ON} + d\log P_{\rm ON}/dP_{\rm ON} = d\log R/dP_{\rm ON}$ and changes in R are thus dominated by changes in P_{ON} when $d\log P_{\rm ON}/dP_{\rm ON} = 1/P_{\rm ON} > d\log K/dP_{\rm ON}$. The log-derivative is computed from the mean trend in data (Fig. 3I) and the transition is at $P_{\rm ON} = 0.75$ (black dashed line). (C) Fraction of changes in R explained by $P_{\rm ON}$ as a function of $P_{\rm ON}$. Black line shows $d\log P_{\rm ON}/dP_{\rm ON}$ normalized by the sum $d\log K/dP_{\rm ON} + d\log P_{\rm ON}/dP_{\rm ON}$ (see B). The solid black line is computed as in B, whereas the dotted black line is the corrected contribution assuming two independent sister chromatids (see G and H). Most of the changes in R are thus mostly dictated by $P_{\rm ON}$. (D-F) Single gene copy (sgc) parameter computed from the estimated effective parameters for *hb* in NC14, assuming two independent sister chromatids. The color code stands for AP as A. The spatiotemporal regulation of the sgc transcription rate $R^{(1)}$ (D) and the sgc ON-probability $P_{\rm ON}^{(1)}$ (E) are very similar to their corresponding effective parameters, R and $P_{\rm ON}$. On the other hand, the sgc initiation rate $K^{(1)}$ (F) no longer varies across position, but only as a function of time. During NC14, the sgc initiation rate $K^{(1)}$ decreases by 38%, from 8.0 mRNA/min (first dotted line) to 5.0 mRNA/min on average (third dotted line), with most of the decrease happening between the 16$^{\rm th}$ and 34$^{\rm th}$ minute mark (second dotted line at 6.1 mRNA/min). (G-I) Temporal changes in sgc initiation rate and independent sister chromatid assumption explain the dependence of the effective initiation on the ON-probability. Color code stands for three time-windows in NC14: early (cyan, 2.5-16.7 min), mid (purple, 16.7-34.2 min), and late (magenta, 34.2-50 min). (G) Most of the variation in $K^{(1)}$ is explained by time, rather than $P_{\rm ON}^{(1)}$. The dotted lines are drawn at the same $K^{(1)}$ values as in C. (H) The sgc transcription rate $R^{(1)}$ appears almost linearly dependent on $P_{\rm ON}^{(1)}$. The nonlinearity is mostly explained by temporal changes in $K^{(1)}$, as highlighted by dotted lines whose slopes are the $K^{(1)}$ values from F and G. (I) Under the two independent sister chromatids assumption, the effective initiation rate K depends on $P_{\rm ON}$ and on $K^{(1)}$, which varies as a function of time (see F). As $P_{\rm ON}$ increases, the propensity to observe two gene copies initiating transcription at the same time increases, which explains up to a factor of two in the dependence of K on $P_{\rm ON}$. Indeed, the dotted lines correspond to the predicted behavior using the same three constant values of $K^{(1)}$ as in F. In addition, $K^{(1)}$ varies by up to 38% along time during NC14, as can be seen in F. Together, it explains close to a factor of 3.2 in K variation with $P_{\rm ON}$. This reasoning explains the observed relationships very well (black line) and argues for a very weak dependence of K on $P_{\rm ON}$. (J) Close-up of the first 10 min of Fig. 3D shows a rapid transient in the ON-probability for *hb* at the onset of NC14. The vertical dashed line at 7.5 min marks the transition between the transient and the near-steady-state regime as observed in H. (K) Evidence for out-of-steady state transient for *hb* in NC14. Near the steady-state regime, $P_{\rm ON}$ should be well-approximated by $T_{\rm ON}/(T_{\rm ON} + T_{\rm OFF})$, as it is the case beyond the 7.5 min mark in NC14 (gray circles tracing the diagonal; see also Fig. 4D). However, at the post-mitotic onset of transcription ($\sim$3–7.5 min into NC14) we observe strong deviations from the expected near-steady state relationship at all positions (color curves). The system is undergoing a fast transient relaxation that drives it near-steady state within the first 8 min after mitotic exit.

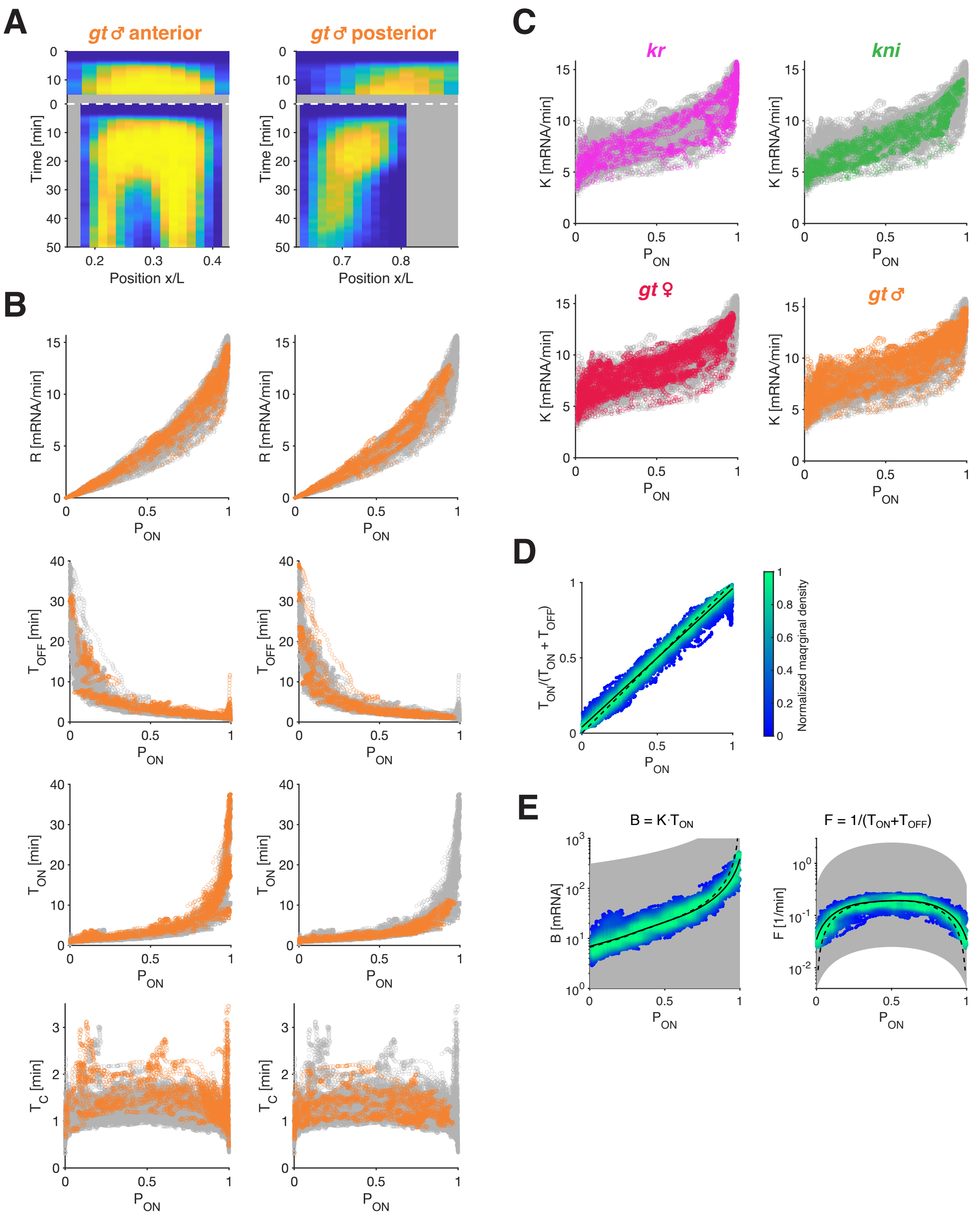

FIG. S8. **Further transcriptional parameters collapse.** (A) Kymograph of ON-probability for *gt* transcription in male embryos, as a function of position and time. As for the other gap genes, the spatiotemporal transcription patterns arise from complex regulation of the ON-probability. (B) Transcriptional parameters for *gt* in male embryos for NC13 and NC14 as a function of PON (orange data points) and all the other gap gene data sets (gray). (C) Initiation rate K as a function of $P_{\rm ON}$. K collapses for all gap genes across time and position. Colored data points represent individual gap genes (same color code as in Fig. 5A-E and S8A-B); underlying is the remaining data of all other genes (gray). The K–$P_{\rm ON}$ relationship for *hb* is shown in Fig. 3H. (D) Near-steady state relationship between ON-probability $P_{\rm ON}$ and $T_{\rm ON}/(T_{\rm ON}+T_{\rm OFF})$ for all gap genes in NC13 ($t \geq 6.5$min) and NC14 ($t \geq 7.5$min). Although the data is distributed near the expected relationship (dash line), we observe a slight but clear bias at the extreme ends of the $P_{\rm ON}$ spectrum (solid line), namely $T_{\rm ON}/(T_{\rm ON}+T_{\rm OFF})$ is slightly above zero at $P_{\rm ON}=0$ and slightly below one at $P_{\rm ON}=1$. This is a consequence of the finite nature of our recording (50 min in NC14 and 18.4 min in NC13). Thus, our recording time sets an upper limit on the length of measurable ON and OFF intervals. That limit leads to the observed bias. (E) Global scatter of the burst size B and burst frequency F (color) as a function of $P_{\rm ON}$ for all gap genes in both NC13 and NC14 and putative accessible space (gray region). See definitions of B and F at top of respective panels. Color code and accessible space defined as in Fig. 5F. Solid line stands for the bursting rules derived with bias (solid line in C) and dashed line without.

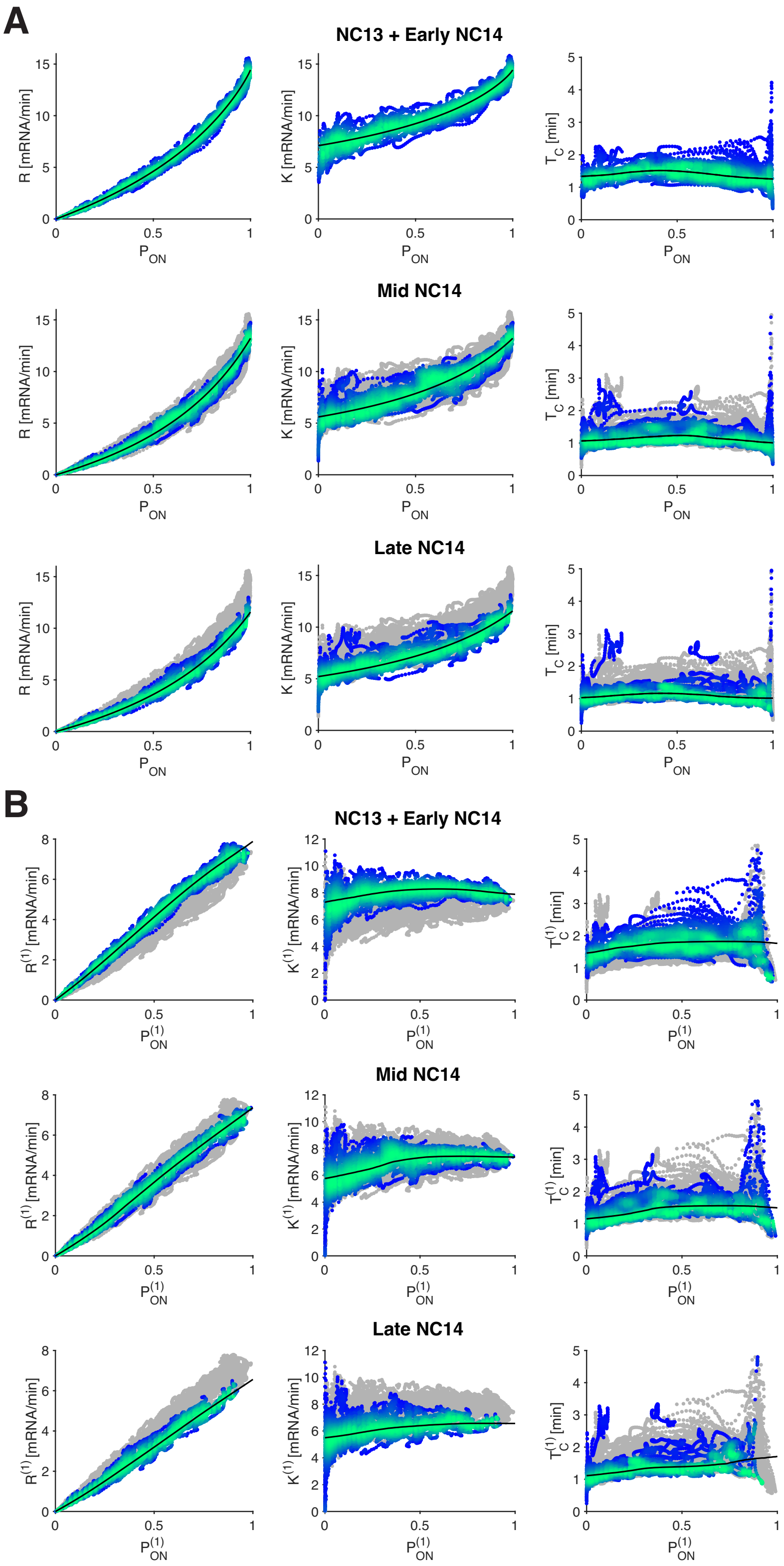

FIG. S9. **Common bursting rules across developmental time and for single gene copy.** (A) Global scatter of effective transcriptional parameters as a function of P_{ON} for all gap genes estimated within three time windows, corresponding to NC13 ($6.5 \leq t$ min) plus early NC14 ($7.5 \leq t < 20.5$ min), mid NC14 ($20.5 \leq t < 34.5$ min), and late NC14 ($34.5 \leq t < 48$ min). The observed bursting relationships are further refined when accounting for possible temporal changes (color scatter) compared to all time-pooling (gray scatter, Fig. 5F). Indeed, small changes in K and T_{C} over developmental time ($\sim 40\%$ decrease) explain part of the observed spread in Fig. 5F. (B) As in A, but for single gene copy parameters computed from the effective ones assuming independent sister chromatids. Interestingly, the relationship between sgc transcription rate $R^{(1)}$ and sgc ON-probability $P_{\mathrm{ON}}^{(1)}$ is almost linear, confirming that the sgc initiation rate $K^{(1)}$ does not depend strongly on $P_{\mathrm{ON}}^{(}(1))$. Thus, the apparent dependence of $K^{(1)}$ on $P_{\mathrm{ON}}^{(}(1))$ is only effective and results from measuring two sister chromatids (two gene copies) together, instead of an isolated single gene copy.

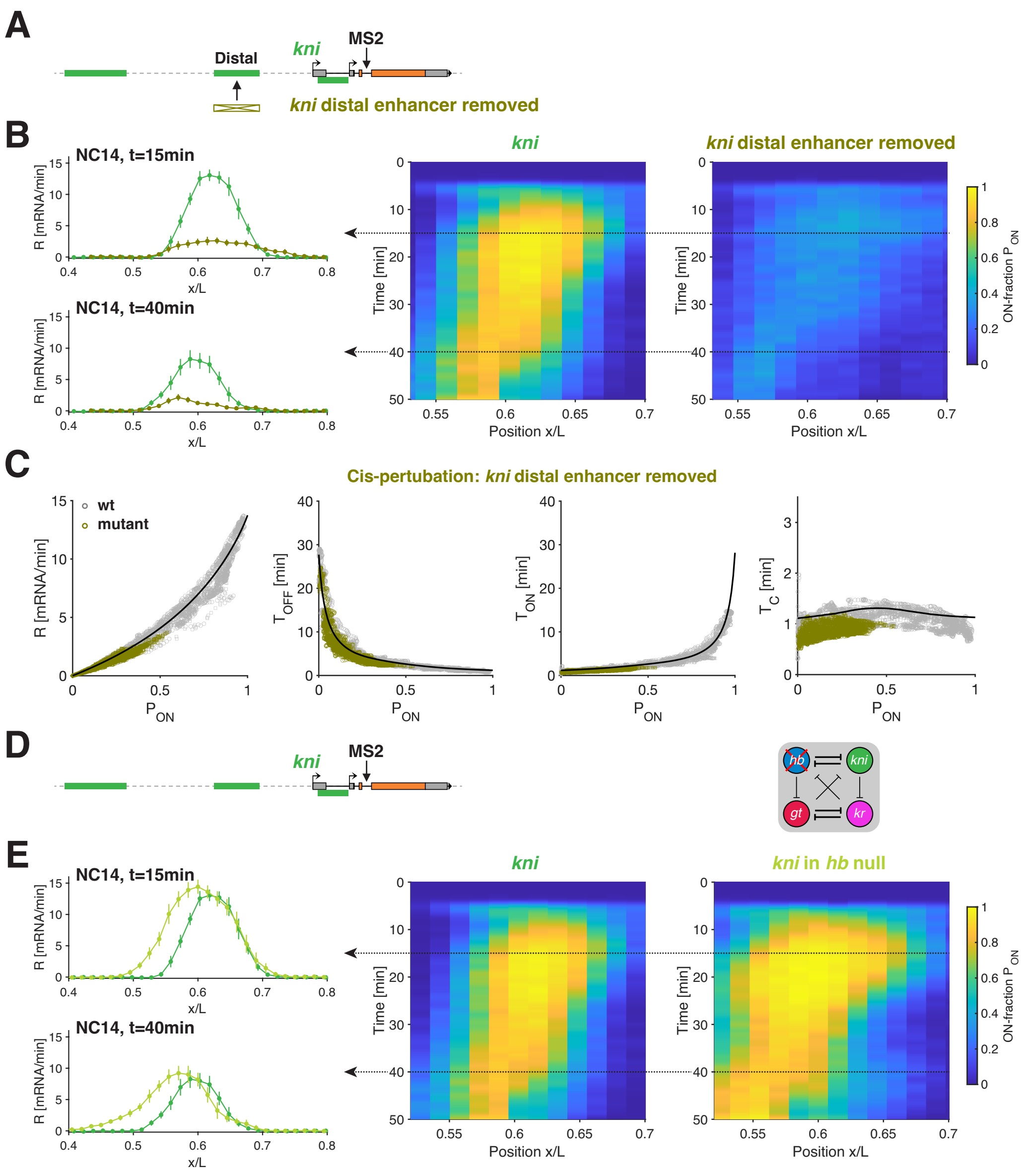

FIG. S10. **Bursting rules verified by *cis*- and *trans*-perturbations.** (A) Distal *kni* enhancer removal. The MS2-stem loops are inserted at the same location in the mutant (enhancer deletion) and wild-type fly lines. (B) Quantification of *kni* wild-type and mutant (A) phenotypes. Both transcription rate R as a function of x/L (left) and the kymograph for P_{ON} (right) display a significant level decrease underlying the expression patterns of the mutant. Dotted arrow indicates time point in kymograph at which rate profiles (left) are depicted. (C) Transcription parameters for *kni cis*-mutant (olive) collapse on corresponding wild-type parameters (gray), as for *hb cis*-mutant (see Fig. 6C). Solid black lines correspond to the endogenous bursting rules from Fig. 5F. (D) *kni* measurements in a *hb* null background. The absence of *hb* expression alters the network, namely the concentration of input transcription factors sensed by *kni* in the mutant fly line. (E) Quantification of *kni* wild-type and mutant (D) phenotypes. Both transcriptional rate R (left) and P_{ON} kymograph (right) display a significant shift of the anterior boundary in the mutant expression patterns. Dotted arrow indicates time point in kymograph at which rate profiles are depicted.

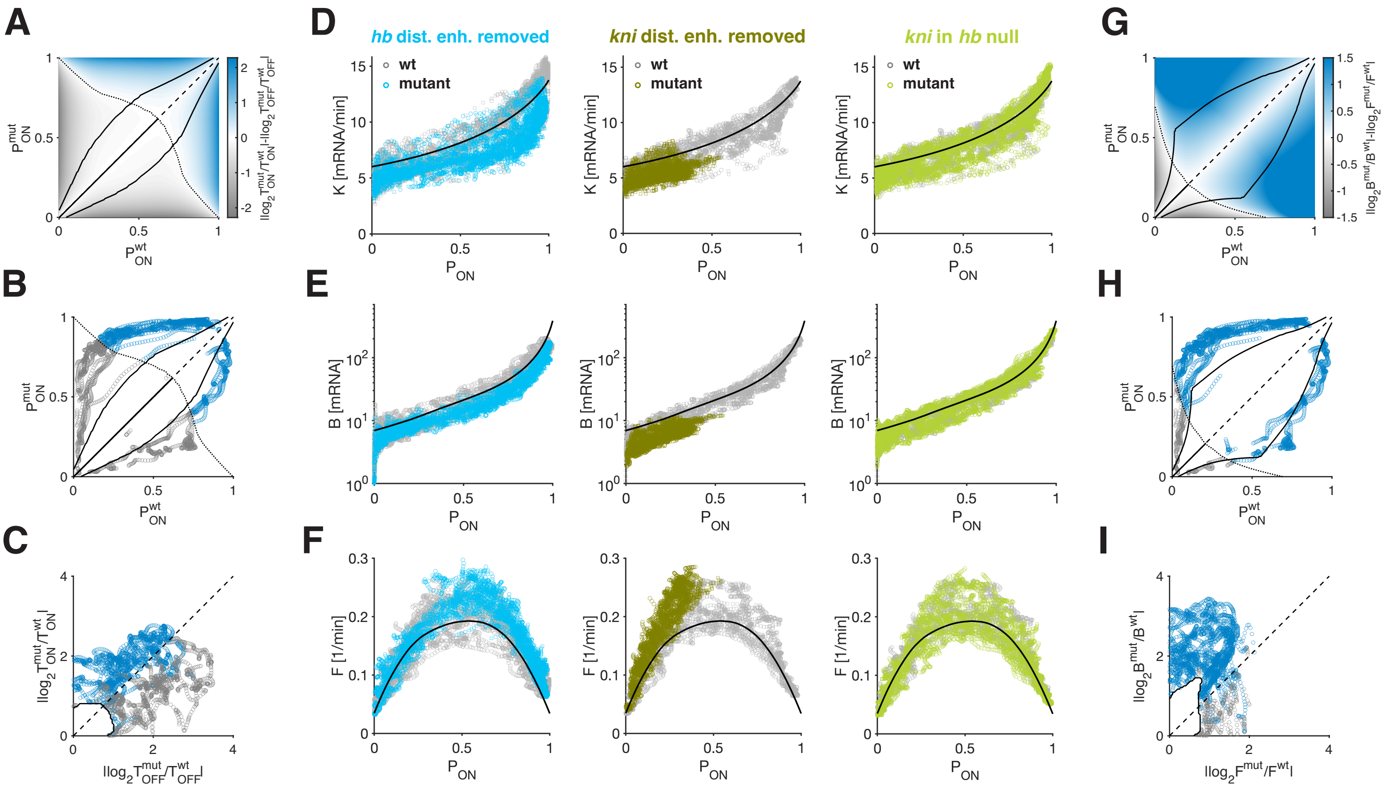

FIG. S11. **Bursting rules predict ON and OFF modulation by *cis*- and *trans*-perturbations.** (A-C) Predicted $T_{\rm OFF}$ versus $T_{\rm ON}$ bursting modulation for mutant based on wild-type-derived rules (Fig. 5F, black lines). (A) The type of modulation is predicted by first approximating $T_{\rm OFF}$ and $T_{\rm ON}$ as a function of $P_{\rm ON}$ using the wild-type rules. The predicted fold change in $T_{\rm OFF}$ ($T_{\rm OFF}^{\rm mut}/T_{\rm OFF}^{\rm wt}$) and $T_{\rm ON}$ ($T_{\rm ON}^{\rm mut}/T_{\rm ON}^{\rm wt}$) are then computed for all possible pairs of $P_{\rm ON}$ (i.e. $P_{\rm ON}^{\rm wt}$ and $P_{\rm ON}^{\rm mut}$). The dotted line delimits the regions where changes in transcription rate are either dominated by changes in $T_{\rm OFF}$ (gray region, $|\log(T_{\rm OFF}^{\rm mut}/T_{\rm OFF}^{\rm wt})| > |\log(T_{\rm ON}^{\rm mut}/T_{\rm ON}^{\rm wt})|$) or $T_{\rm ON}$ (blue region, $|\log(T_{\rm OFF}^{\rm mut}/T_{\rm OFF}^{\rm wt})| < |\log(T_{\rm ON}^{\rm mut}/T_{\rm ON}^{\rm wt})|$). The solid black lines delimit the region, where changes in $T_{\rm OFF}$ and $T_{\rm ON}$ are not significant given the "thickness" of our relationships (95% confidence intervals, see Methods). Thus, this procedure defined a look-up table enabling prediction of the type of modulation using pairs of $P_{\rm ON}$. (B) Scatter plot of all the $P_{\rm ON}$ pairs from *hb* wt and *cis*-mutant (at same spatiotemporal location). Colors correspond to the predicted modulation ($T_{\rm OFF}$ dominated in gray and $T_{\rm ON}$ dominated in blue) using the look-up table in A. (C) Verification of predicted modulation in B (color code as in B). For each $P_{\rm ON}$ pair, we computed the $T_{\rm OFF}$ ($T_{\rm OFF}^{\rm mut}/T_{\rm OFF}^{\rm wt}$) and $T_{\rm ON}$ ($T_{\rm ON}^{\rm mut}/T_{\rm ON}^{\rm wt}$) fold change using the estimated $T_{\rm OFF}$ and $T_{\rm ON}$ from data (Fig. 6C). Supporting our ability to predict the modulation, almost all the blue data points (predicted as $T_{\rm ON}$ modulation) are located above the slope 1 diagonal (dashed line), whereas most of the gray ones (predicted as $T_{\rm OFF}$ modulation) are below. Thus, for most data points (> 85%) the prediction is correct (Fig. 6F). (D-F) Initiation rate K, burst size B, and burst frequency F for *hb* in *cis*-mutation (cyan), *kni* in *cis*-mutation (olive) and for *kni* in *trans*-mutation (light green) collapse on corresponding wild-type parameters (gray). Solid black lines correspond to the endogenous bursting rules from Fig. 5F and S8E. (G-I) Predicted F versus B modulation for mutant based on wild-type-derived rules (Figure S8E, black lines). (G) F and B are first approximated as function of $P_{\rm ON}$ using the wild-type rules. The predicted fold change in F ($F^{\rm mut}/F^{\rm wt}$) and B ($B^{\rm mut}/B^{\rm wt}$) are then computed for all possible pairs of $P_{\rm ON}$ (i.e. $P_{\rm ON}^{\rm wt}$ and $P_{\rm ON}^{\rm mut}$). The dotted line delimits the regions where changes in transcription rate are either dominated by changes in F (gray region, $|\log(F^{\rm mut}/F^{\rm wt})| > |\log(B^{\rm mut}/B^{\rm wt})|$) or B (blue region, $|\log(F^{\rm mut}/F^{\rm wt})| < |\log(B^{\rm mut}/B^{\rm wt})|$). As for $T_{\rm OFF}$ and $T_{\rm ON}$ (in A), the solid black lines delimit the region of significance and we have thus defined a look-up table enabling prediction of F vs B modulation using pairs of $P_{\rm ON}$. (H) Scatter plot of all the $P_{\rm ON}$ pairs from *hb* wt and *cis*-mutant (at same spatiotemporal location). Colors correspond to the predicted modulation (F dominated in gray and B dominated in blue) using the look-up table in G. (I) Verification of predicted modulation in H (color code as in H). For each $P_{\rm ON}$ pair, we computed the F ($F^{\rm mut}/F^{\rm wt}$) and B ($B^{\rm mut}/B^{\rm wt}$) fold change using the estimated F and B from data (E and F). Supporting our ability to predict the modulation, almost all the blue data points (predicted as B modulation) are located above the slope 1 diagonal (dashed line), whereas most of the gray ones (predicted as F modulation) are below. Thus, for most data points (> 95%) the prediction is correct (Figure 6G).

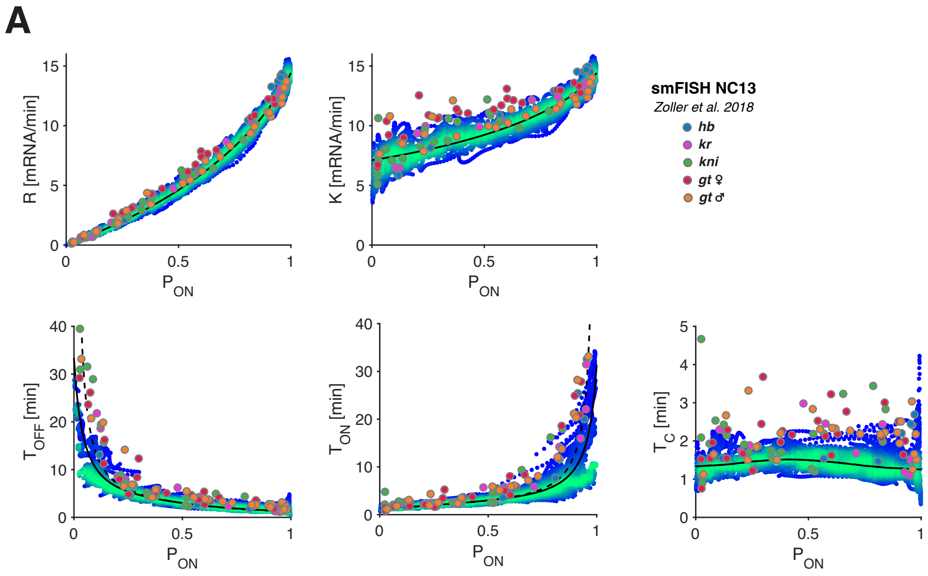

FIG. S12. **Validating bursting rules with smFISH data.** (A) Bursting relationships in NC13 and early NC14 are consistent with parameters inferred from previous smFISH measurements. We converted the single gene copy parameters of the gap genes in mid-late NC13 (Zoller et al.) into effective parameters for two sister chromatids (color dots). Overall, these parameters closely verify our relationships derived from live measurements.
